# Ambiphilic Ligand Stabilization of Aluminum(I) Fragments: Halides, Cations, and a Hydride

**DOI:** 10.1002/anie.6333732

**Published:** 2026-08-05

**Authors:** Charlotte S. V. Weiß, Philipp Dabringhaus

**Affiliations:** ^1^ Anorganisch‐Chemisches Institut Ruprechts‐Karls‐Universität Heidelberg Heidelberg Germany

**Keywords:** ambiphilic ligands, bonding analysis, boron‐aluminum cooperativity, density‐functional theory, low‐valent aluminum

## Abstract

Z‐type ligands have been highly successful in stabilizing transition‐metal fragments in unique coordination geometries and in enabling novel reactivities. However, examples of the use of these ligands in stabilizing elusive main‐group metal fragments are scarce. In this study, we present the stabilization of aluminum (I) halides by an ambiphilic tris(phosphine)borane ligand. Unlocking unprecedented coordination chemistry at aluminum, the unique electronic structure of the novel compounds is analyzed in detail experimentally and computationally. Successful post‐functionalization via coordination of Lewis bases to the Al atom enables isolation of elusive Al cations with the metal in a low redox state. In addition, the Al(I)Br transfer onto 2,2′‐bipyridine ligands is presented.

## Graphical abstract

Stabilization of elusive aluminum(I) halides in the framework of an ambiphilic ligand is reported. The unique bonding situation at the trigonal‐bipyramidal aluminum center is analyzed, and the coordination chemistry and Al(I)Br transfer reactivity of the complexes are presented.

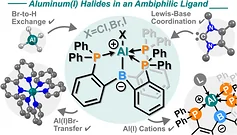

## 1 Introduction

Ambiphilic ligands, which are composed of L‐type donor and Z‐type acceptor groups, emerged in the last decade as a highly promising class of ligands in transition‐metal (TM) chemistry [1]. Owing to their unique coordination abilities, these versatile ligands not only allowed for the isolation of novel, unusual coordination geometries, especially at electron‐rich, late transition metals, but also enabled novel reactivities and catalytic applications of TM complexes [2]. Phosphine‐boranes evolved into one of the most widely used classes of ambiphilic ligands (Figure 1A). Early studies in the field focused on understanding the bonding situation in the respective group 10 and 11 metal complexes (e.g. Pd, Pt, AuCl) in great detail [3, 4, 5, 6, 7]. In the last decade, it has been shown that the unusual coordination geometries enforced by the ambiphilic ligands can translate into unique reactivity. In particular, the trigonal‐bipyramidal coordination induced by the tetradentate tris(phosphine)borane (TPB) ligand has been shown to enable unprecedented reactivities of Fe and Co complexes in low redox states [8, 9, 10, 11, 12, 13, 14, 15]. In a milestone work, the iron complex of the tetradentate TPB ligand was discovered to catalyze the conversion of nitrogen into ammonia [16]. Detailed studies on this reactivity have since been reported [17, 18, 19, 20, 21]. Closely related to TPB, ligands incorporating three phosphine donors and heavier group 13 Z‐type ligands have been used for the isolation of trigonal bipyramidal TM complexes [22, 23, 24, 25]. Overall, the research on TM‐complexes with Z‐type ligands has been extremely active in recent years, resulting in the expansion of the acceptor atoms, for example, to silicon, phosphorus, or antimony [26, 27, 28, 29, 30, 31, 32]. Despite this great success of ambiphilic ligands in the coordination sphere of late transition metals, examples for the stabilization of unique main‐group metal complexes with ambiphilic ligands are extremely scarce [33]. Using a phosphine‐borane ligand of type **I**, elusive SiH_2_ and GeH_2_, as well as arsenic fragments in low redox states, could be trapped successfully [34, 35].

**FIGURE 1:**
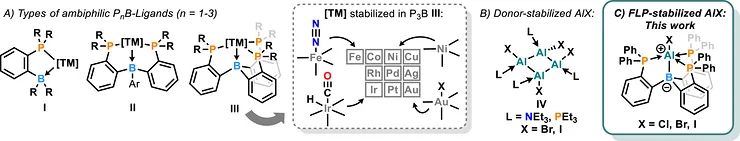
A) Types of ambiphilic phosphine‐borane ligands and metals for which tris(phosphine)borane (TPB) complexes have been reported [7, 9, 13, 53]. B) Structurally‐characterized, donor‐stabilized Al(I)X tetramers [40, 41]. C) AlX salts stabilized in ambiphilic TPB ligand.

Inspired by the stabilization of TM fragments with ambiphilic ligands, we were interested in expanding the use of TPB ligands in the coordination of elusive main‐group metal fragments, in particular of aluminum. Aluminum complexes in their low‐redox states are extremely reactive, and isolation requires precise control of steric and electronic properties [36]. In contrast to the heavier analogs gallium, indium, and thallium, for which univalent starting materials of type M(I)X (X = halides or weakly‐coordinating anion) are easily accessible or commercially available, aluminum(I) halides are only accessible by co‐condensation of gaseous AlX at low temperatures in a highly specialized apparatus (e.g. **IV** in Figure 1B) [37, 38]. Structural characterization of elusive tetramers of AlCl, AlBr, and AlI as their amine or phosphine adducts could be performed upon co‐condensation of AlX with the respective ligands [39, 40, 41]. Since the milestone works by the group of Schnöckel on metastabile AlX compounds, a large variety of neutral and anionic Al(I) complexes have been developed, which show a diverse reactivity in the activation of small‐molecules [42, 43, 44, 45, 46, 47, 48, 49]. In these compounds, the Al(I) center is stabilized by bulky anionic ligands, which are irreversibly bonded to aluminum, limiting the possibilities for post‐functionalization of the reactive species or transfer of the Al(I) atom. Hence, the stabilization of Al(I)X fragments by a neutral ligand framework is expected to enable post‐functionalization at the aluminum center, the straightforward preparation of novel Al(I) complexes via aluminum atom transfer, or access to hitherto unknown species, such as monomeric Al cations in low redox states. Notably, the only other examples of stabilized formal Al(I)X fragments have been reported as complexes with the TM fragments Fe(CO)_4_ or Cr(CO)_5_, formed by reduction of the AlX_3_ complexes with the respective TM anions [50, 51, 52].

In this manuscript, we report the isolation and characterization of Al(I)X fragments (X═Cl, Br, I) stabilized in a simple ambiphilic TPB ligand (Figure 1C). An extensive DFT analysis of the bonding situation in the complexes, incorporating a uniquely trigonal‐bipyramidal‐coordinated aluminum atom in a low redox state, indicates pronounced boron‐aluminum covalency. Moreover, the reactivity of the novel compounds towards Lewis bases is investigated, revealing the flexibility of the TPB ligand's coordination and the accessibility of elusive Al cations in a low redox state. Moreover, the successful transfer of an Al(I)Br fragment is reported.

## 2 Results and Discussion

### 2.1 Preparation and Characterization of [(TPB)AlX] (X═Cl, Br, I) Complexes

First, the reduction of TPB ligand **V** [53] was attempted. While the addition of KC_8_ to a THF‐solution of **V** resulted in decomposition, no reaction was observed with [(thf)_3_Mg(Anthracene)]. Adding 2 equivalents of KC_8_ to a mixture of AlBr_3_ and **V** in toluene resulted in a color change from yellow to dark red. In the NMR spectra, the formation of a novel species was observed, appearing as quartets in the ^11^B NMR spectrum at −7.8 ppm (^3^ *J*_B,p_ = 41.0 Hz) and in the ^11^P{^1^H} NMR spectrum at −23.3 ppm (^3^ *J*_B,p_ = 41.0 Hz). Moreover, the characteristic singlet of [AlBr_4_]^−^ was observed at +81.0 ppm in the ^27^Al NMR spectrum. Yet only one‐third of **V** has reacted. Rationalizing these observations, we postulated the [(TPB)AlBr]^2+^ dication as a possible intermediate in the reaction (Scheme 1), resulting from reaction of the ligand with excess AlBr_3_. Mixing **V** with 3 equivalents of AlBr_3_ in 1,2‐difluorobenzene (1,2‐DFB) yielded the nearly quantitative formation of a novel species with a broad ^31^P{^1^H} NMR resonance at −3.9 ppm and a novel ^27^Al NMR resonance at 125.5 ppm, which we attributed to [(TPB)AlBr]^2+^([AlBr_4_]^−^)_2_ **2** (see Supporting Information). Unfortunately, partial decomposition was observed upon isolation, and unambiguous confirmation of the structure by single‐crystal x‐ray diffraction (scXRD), IR spectroscopy, or mass spectrometry was unsuccessful (see Supporting Information). Addition of cobaltocene resulted in a color change of the solution to dark‐red, accompanied by the clean formation of the previously observed novel species. Structural analysis by scXRD identified the product as the formal Al(I)Br complex of TPB **5** (Figure 2). Repeating the studies for AlCl_3_ and AlI_3,_ respectively, signals attributed to the postulated dications [(TPB)AlCl]^2+^([AlCl_4_]^−^)_2_ **1** and [(TPB)All]^2+^([All_4_]^−^)_2_ **3** were observed (see Supporting Information). The addition of cobaltocene to the reaction solutions yielded red solutions of the respective [(TPB)AlCl] **4** and [(TPB)AlI] **6**, which showed NMR spectra similar to those of **5** (see Supporting Information). Both compounds could be unambiguously characterized by single‐crystal x‐ray diffraction. After work‐up, the novel compounds **4–6** could be isolated in yields of 26%, 69%, and 41%. Purity was confirmed by NMR spectroscopy, IR spectroscopy, and UV–vis spectroscopy (vide infra, see Supporting Information).

**SCHEME 1:**
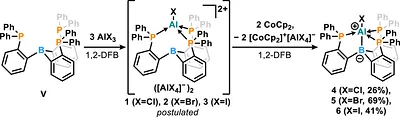
Synthesis of AlX fragments incorporated into TPB ligands.

**FIGURE 2:**
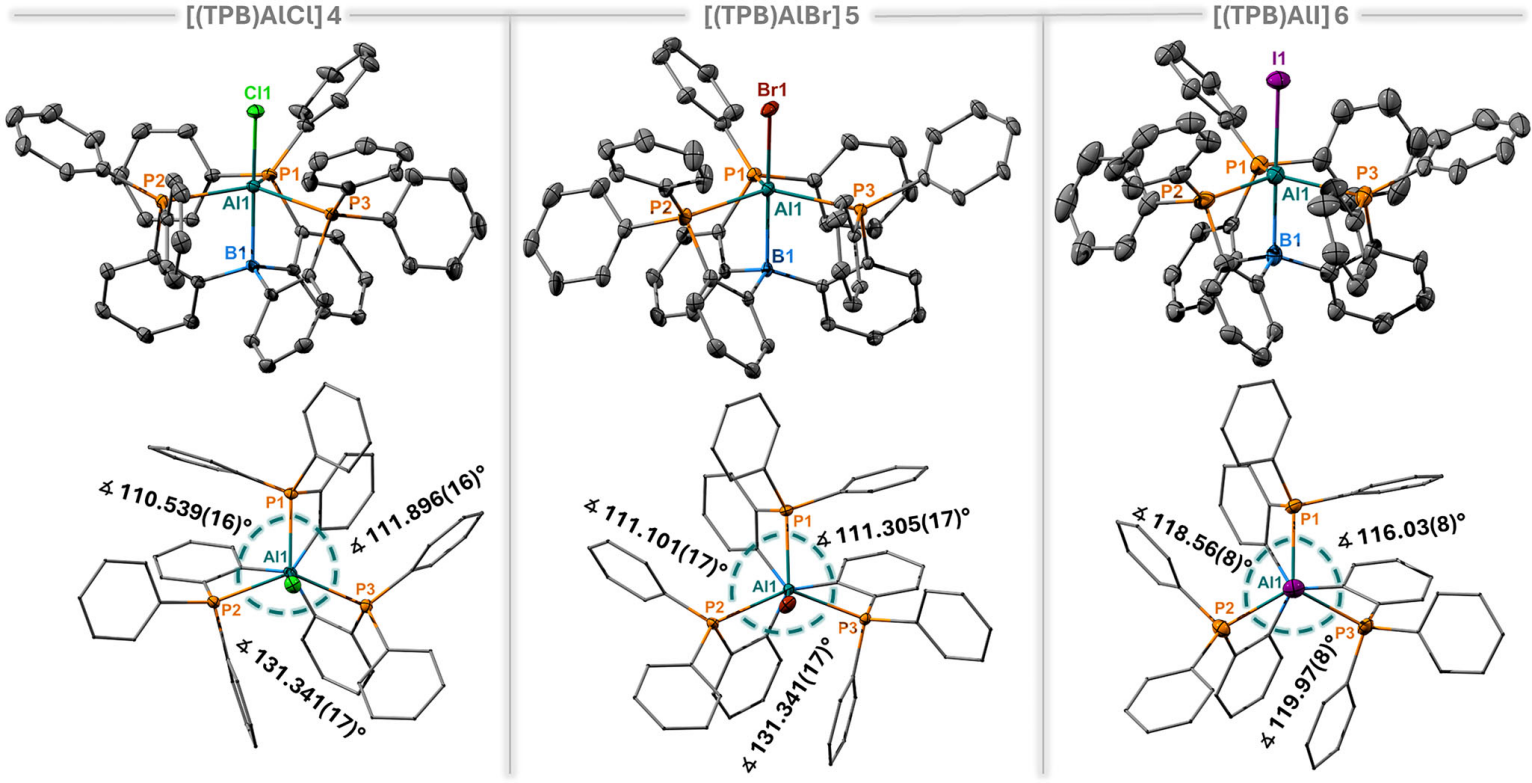
Molecular structures of **4**, **5**, and **6**. Thermal displacement of the ellipsoids was set at 50% probability. Hydrogen atoms are omitted for clarity. Selected distances (in Å) and angles (in °): **4**: Al1−Cl1 2.1913(4), Al1−B1 2.2112(11), Al1−P1 2.4447(5), Al1−P2 2.5418(4), Al1−P3 2.5863(4), Cl1−Al1−B1 170.66(4), P1−Al1−P2 110.539(16), P2−Al1−P3 132.098(19), P3−Al1−P1 111.896(16); **5**: Al1−Br1 2.3500(4), Al1−B1 2.1897(13), Al1−P1 2.4433(5), Al1−P2 2.6004(4), Al1−P3 2.5601(5), Br1−Al1−B1 168.06(4), P1−Al1−P2 111.101(17), P2−Al1−P3 131.341(17), P3−Al1−P1 111.305(17); **6**: Al1−l1 2.623(2), Al1−B1 2.213(7), Al1−P1 2.484(2), Al1−P2 2.529(2), Al1−P3 2.532(2), l1−Al1−B1 178.3(2), P1−Al1−P2 118.56(8), P2−Al1−P3 119.97(8), P3−Al1−P1 116.03(8).

The molecular structures of compounds **3–5** display a trigonal bipyramidal (tbp) coordination geometry, in which the aluminum atoms are coordinated by three phosphines in the equatorial positions while the halide and boron ligands occupy the apical positions. While tbp Al(III) atoms are widely known [54, 55], this coordination geometry of an Al compound in a low redox state, formally described as an [ML_3_XZ] complex by the CBC (covalent bond classification) method, is unprecedented in main‐group chemistry.

The chlorine‐ and bromine‐derivatives show a slight distortion from the ideal tbp geometry, resulting in one shorter Al−P1 bond and a widened P2−Al−P3 angle. Moreover, the B−Al−X bond angles are observed to deviate from the ideal 180°. In **6**, the distortion of the coordination geometry is significantly reduced. As observable in the molecular orbitals (see Supporting Information), the observed distortions in **4** and **5** are a result of the minimization of repulsive interactions between the P−Al bonds and the lone pairs at the halide. The B−Al bonds in **4–6** (**4**: 2.2115(16) Å, **5**: 2.1886(16) Å, **6**: 2.213(7) Å) are slightly longer compared to literature‐known B−Al bonds, typically observed in the range from 2.10 to 2.18 Å [56, 57, 58, 59, 60]. Notably, one other B−Al bond length above 2.20 Å has been reported previously in the aluminaborane [R_2_FAl−B(Mes)_2_]^−^ (2.281(2) Å). Here, the elongation was attributed to steric repulsion between bulky substituents at Al and B [58]. Compared to B−C[Ar] bond lengths in the free ligand of 1.571(3), possessing a Lewis‐acidic, trigonal‐planar boron atom, the respective B−C[Ar] bond lengths are increased in **4–6** (**4**: 1.618(2) Å‐1.636(2) Å; **5**: 1.616(2) Å‐1.630(2) Å; **6**: 1.607(9) Å‐1.637(9) Å), indicative of the more electron‐rich nature of the tetrahedral boron atom. While the shortest Al−P bond lengths in **4** (2.4446(5) Å) and **5** (2.4432(6) Å) fit well with bond lengths in PPh_3_ adducts of strong Lewis acids AlCl_3_ (2.4296(15) Å) [61] and Al{OC(CF_3_)_3_} (2.441(1) Å) [62], the longer Al−P in **5** compare well to the PPh_3_ adduct of the weak Lewis acid AlMe_3_ (2.5329(5) Å) [63]. This indicates significant differences in the Al−P bond strengths in [(TPB)AlX] compounds.

The majority of literature‐known compounds with Al−B bonds are colorless or slightly yellow [59, 60, 64]. The compounds **4–6**, however, are dark red in the solid state and solution. For **5**, the UV–Vis spectrum reveals an intense UV–Vis band at 356 nm and a broad shoulder at 458 nm.

The experimental values are in agreement with excitations computed by time‐dependent density functional theory (CAM‐B3LYP‐D3(BJ)/def2‐TZVP//r^2^SCAN‐3c). Similar experimental and computational results are obtained for **4** and **6** (see Supporting Information). The lowest energy absorptions correspond to transitions from the Al−B bond (HOMO = highest occupied molecular orbital) into empty orbitals, showing contributions of *p*_x_ and *p*_y−_ orbitals at Al (LUMO = lowest unoccupied molecular orbital, LUMO+1, see Figure 3). These insights are consistent with the bonding situation expected for formal Al(I)X fragments stabilized in the ambiphilic TPB ligand. Here, the HOMO can be formally described as an Al lone pair with high s‐orbital character stabilized by the Lewis‐acidic boron‐atom in a B−Al bond. The LUMO/+1 orbitals are characterized as empty p_x_ and p_y_ orbitals stabilized by interactions with the phosphine moieties.

**FIGURE 3:**
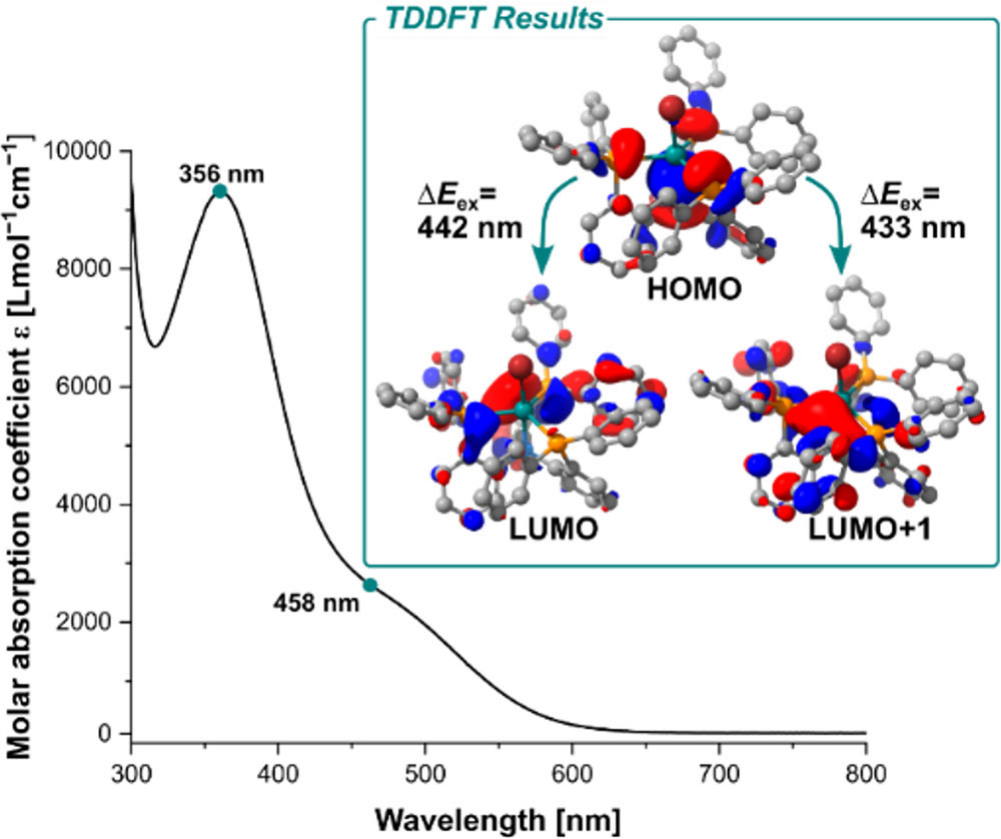
UV–Vis spectrum (1,2‐DFB, rt) of **5,** along with a representation of the molecular orbitals (isovalue 0.03), which participate in the dominant electronic transitions. Computed at CAM‐B3LYP‐D3(BJ)/def2‐TZVP//r2SCAN‐3c (CPCM, 1,2‐DFB) level of DFT.

### 2.2 Bonding Analysis

To gain further insight into the bonding mode of compounds **4–6**, DFT calculations were performed, which are presented in Figure 4 for **5**. In the NBO analysis (= natural bonding orbitals), the B−Al interaction is described as a polarized, covalent bond, as indicated by the NBO coefficients and reduced Wiberg bond index (Figure 4). Importantly, minor stabilization energies E(2) are computed for the NBO associated with the s Al−B bond (see Supporting Information), indicating minor relevance of other Lewis formulations of the bond. This supports the description as a covalent bond with a minor contribution of the donor‐acceptor interaction. In accordance with the asymmetry observed in the molecular structure, one Al−P is defined as a covalent bond in the NBO picture, while two Al−P bonds are characterized as phosphorous lone pairs delocalized onto aluminum in the form of a dative bond. The QTAIM picture (= quantum theory of atoms in molecules) supports a mixture of covalent and dative bonding in the form of a polarized B−Al bond, with a small positive value for the Laplacian of the electron density, shifted towards the less electronegative Al atom (Figure 4b) [65]. Notably, the QTAIM analyses based on the DFT‐optimized structures are in good agreement with QTAIM analyses based on the crystallographic model refined with Hirshfeld atom refinement (HAR) non‐spherical structure refinement method (see Supporting Information) [66, 67]. In the EDA‐NOCV analysis (= energy decomposition analysis with natural orbitals for chemical valence), the lowest computed orbital energy interaction Δ*E*_Orb._ energies can indicate the most reasonable fragmentation of a chemical bond [68]. For **5**, the Δ*E*_Orb_ value for the homolytic bond splitting into TPB^−^ (doublet) and AlBr^+^ (doublet) fragments is favored by 10.8 kcal mol^−1^ compared to the heterolytic bond splitting into TPB (singlet) and AlBr (singlet) fragments. The NOCV orbitals (Figure 4c) further support the polar covalent character of the B−Al bond. However, the P−Al bonds are revealed as predominantly dative in nature, since electron density is transferred from phosphorus to aluminum in both NOCV orbitals. Consequently, while the B−Al bond in **5** is best described as polarized covalent bond, the P−Al bonds are best described as dative bonds. While NBO and QTAIM calculations show similar results for **4** and **6** compared to **5**, the energy differences (ΔΔ*E*_Orb._ (Homo‐Het)) between the Δ*E*_Orb_ values computed for heterolytic and homolytic bond fragmentation decrease significantly in the order of **4**> **5**> **6** (+17 kcal mol^−1^> +10.8 kcal mol^−1^> +1.9 kcal mol^−1^). This indicates an increase in the dative bonding character upon increasing the size of the halide from AlCl to AlI.

**FIGURE 4:**
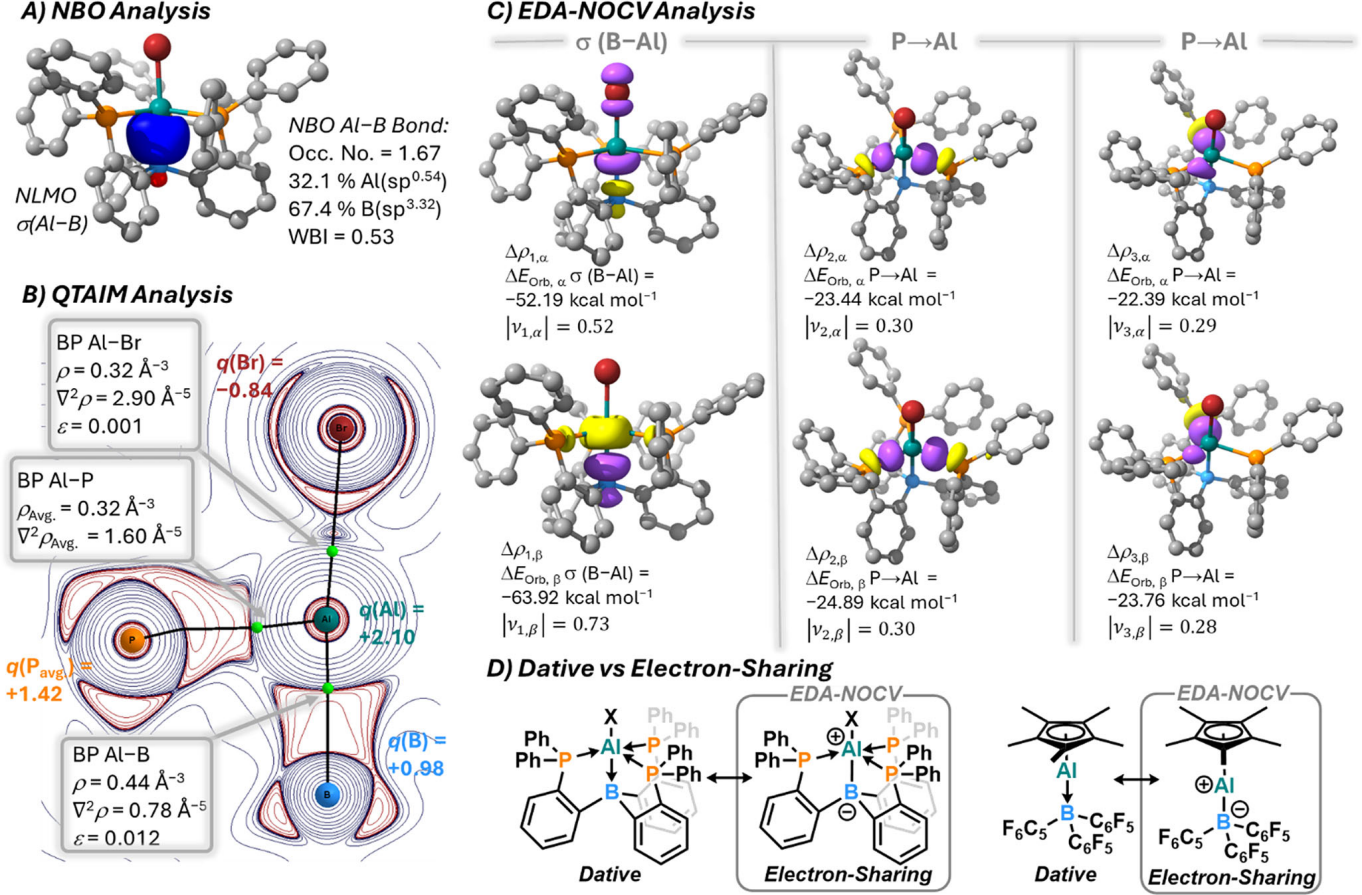
A) Results of NBO analysis for **4** (isovalue 0.07). B) Contour plot for Laplacian of electron density **5** (red = negative, blue = positive; *ρ* = electron density; ∇^2^ *ρ* = Laplacian of electron density; *ε* = ellipticity; *q* = atomic charge). C) Plots of deformation densities Δ*ρ*_(1)‐(3)_ of the pairwise orbital interaction for homolytic fragmentation of **5** in AlBr^+^ and TPB^−^ (isovalue 0.004 for B−Al bond, 0.002 for Al−P bonds). Charge flows from yellow to purple. D) Resonance structures for **3–5** and known Cp*Al−B(C_6_F_5_)_3_.

To place the bonding motives in **4–6** in the context of known B−Al bonds, we performed DFT studies on isolated examples. Cp*Al→B(C_6_F_5_)_3_ [64] is regarded as a prototypical example of an Al→B donor‐acceptor bond. QTAIM, NBO, and EDA‐NOCV analysis, however, indicate that the bond in this compound is better described as a covalent bond (see Supporting Information). The EDA‐NOCV reveals a positive *ΔΔE*_Orb_.(Homo‐Het) value of +20.64 kcal mol^−1^, which is even larger than values determined for **4**−**6**. Analysis of the adduct Cp*Al→BF(Ph) [69], which incorporates the less Lewis‐acidic 9‐phenyl‐borafluorene, yields a negative *ΔΔE*_Orb._(Homo‐Het) of −6.00 kcal mol^−1^, indicating a description as a donor‐acceptor bond. Notably, the commonly described covalent bonding interaction in compounds of type R_2_B−AlR_2_ (example: *ΔΔE*_Orb._(Homo‐Het) = +66.41 kcal mol^−1^ for (C_4_H_2_(SiMe_3_)_4_)Al−B(Mes)_2_ [58], see Supporting Information) is unambiguously confirmed by EDA‐NOCV. In summary, the B−Al bonds in **4–6** should be regarded as polarized, covalent bonds, which compare well to known Al−B bond compounds, which were previously defined as donor‐acceptor bonds. In general, identification of dative Al→B bonds is not trivial and does not correlate with the mode of bond formation. The Al−P bonds in **4–6** should be regarded as predominantly dative in nature.

### 2.3 Substitution, Coordination, and Halide Abstraction

Following up on the detailed analysis of the electronic structure of the AlX compounds, we sought to explore their coordination chemistry and potential for structural modification. First, we reacted [(TPB)AlBr] **5** with LiAlH_4_ (Scheme 2), resulting in the precipitation of an orange solid. The novel species could be identified as [(TPB)AlH] **7** by scXRD (Figure 5). In NMR spectroscopy, ^11^B and ^31^P{^1^H} signals of **7**, which show as quartets at −6.0 ppm and −17.6 ppm, are shifted downfield compared to the AlX complexes **4–6**. The hydride is detected as a quartet at +5.82 ppm (^2^ *J*_H,p_ = 50.7). Furthermore, an Al−H stretching vibration at 1791 cm^−1^ was detected by IR spectroscopy.

**SCHEME 2:**
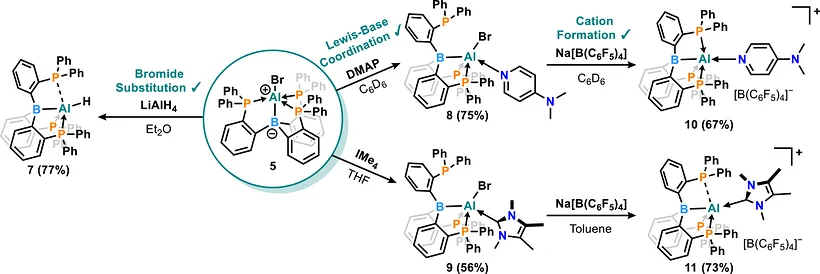
Reactivity of [(TPB)AlBr] towards LiAlH_4_ and Lewis bases. DMAP = 4‐(dimethylamino)pyridine; IMe_4_ = 1,2,3,4‐tetramethylimidazol‐2‐ylidene.

**FIGURE 5:**
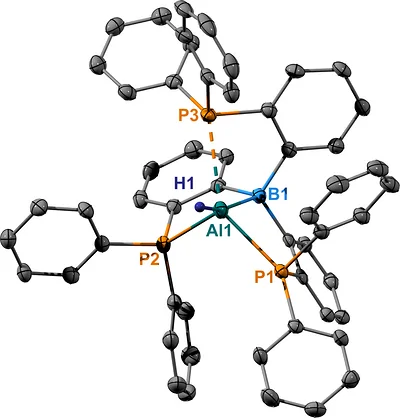
Molecular structures of **7**. Thermal displacement of the ellipsoids was set at 50% probability. Hydrogen atoms are omitted for clarity. Selected distances (in Å) and angles (in °): **7**: Al1−H1 1.73(2), Al1−B1 2.184(8), Al1−P1 2.523(1), Al1−P2 2.436(1), Al1−P3 2.8164(9), H1−Al1−B1 162.4(8), P1−Al1−P2 104.68(4), P2−Al1−P3 102.46(3), P3−Al1−P1 142.99(4).

Intriguingly, the structure of [(TPB)AlH] **7** (Figure 5) deviates from the structures of **4**−**6**. In **7**, two short Al−P bonds are observed, while the third Al−P3 distance is significantly elongated to 2.8164(9) Å. The structural changes are preserved upon optimizing the scXRD structure using DFT. In the QTAIM and NBO analyses, respectively, lower values are computed for the electron density at the BCP of the Al−P3 interaction (*ρ*(Al−P3) = 0.20 Å^−3^; *ρ*(Al−P1) = 0.33 Å^−3^, *ρ*(Al−P2) = 0.27 Å^−3^) and the WBI value (= Wiberg bond index; WBI(Al−P3) = 0.29; WBI(Al−P1) = 0.51; WBI(Al−P2) = 0.42). Hence, the structure can be described as a seesaw‐like coordination at Al. Notably, the structural asymmetry is expected to be highly fluxional in solution, since only one signal is observed in the ^31^P NMR spectrum. The structural change can be attributed to the decreased electronegativity of hydrogen compared to the halogen substituents, rendering the aluminum in **7** less Lewis‐acidic compared to complexes **4–6**. These results reveal promising flexibility in the coordination of the multidentate ligand to the AlX units, indicating increased accessibility of the Al atom due to the hemilability of a phosphorus donor moiety.

Aiming to explore this proposed substitution reaction, we added small Lewis bases 4‐(dimethylamino)pyridine (DMAP) and 1,2,3,4‐tetramethylimidazol‐2‐ylidene (IMe_4_) to solutions of **5**. Here, yellow crystals of the respective DMAP‐ and IMe_4_‐adducts could be isolated (Scheme 2). In both structures (Figure 6), one phosphine donor has been substituted by the added Lewis base, and one very long Al−P3 distance is measured (3.5268(6) Å in **8**, 3.7184(3) Å in **9**). This results in a bipyramidal coordination geometry at Al with two axial phosphorus‐donor moieties. While in **8** both Al−P bond lengths are similar, the Al−P2 bond length is, at 2.7212(8) Å, significantly elongated compared to the Al−P1 bond, measuring 2.5757(8) Å. The asymmetry of structures of **8** and **9** is retained in solution. Here, a broad ^31^P NMR resonance of the non‐coordinating −PPh_2_ unit is observed at −10.6 ppm and −6.8 ppm, respectively, while broad resonances of the Al‐bonded phosphorus atoms show broad singlets at −23.9/−24.9 ppm for **8** and −27.8/−33.3 ppm for **9** (see Supporting Information). These bond changes can be attributed to the higher donor ability of the carbene compared to DMAP. Intriguingly, a similar trend is observed for the Al−Br bond lengths, which are elongated in **8** (2.3552(4) Å) and **9** (2.3562(3) Å) compared to **5** (2.3500(4) Å), indicating a labilization of the respective bonds upon Lewis‐base coordination. Hence, we attempted to abstract a bromide from the Lewis base complexes **8** and **9**. While addition of Na[B(C_6_F_5_)_4_] to a solution of the DMAP adduct resulted in an instantaneous color change of the yellow solution to red, no color change was observed in the case of the carbene adduct. In both reactions, NMR spectroscopy revealed a clean conversion to novel species, which show as ^31^P NMR resonances at −23.4 ppm for the DMAP reaction and at −12.2 ppm for the IMe_4_ reaction. The successful bromide abstraction and formation of a cationic species could be verified by scXRD analysis, yielding the molecular structures of [(TPB)Al(DMAP)]^+^[B(C_6_F_5_)_4_]^−^ **10** and [(TPB)Al(IMe_4_)]^+^[B(C_6_F_5_)_4_]^−^ **11**. Both crystals decomposed rapidly (within a few minutes) upon exposure to air, even when protected by an inert oil. Intriguingly, the structure of **10** reveals a structure similar to the AlX compounds **4–6**, in which the aluminum atom is coordinated by three phosphine donors. The structure of **10**, however, is reminiscent of the hydride **7**, revealing a seesaw‐like coordination with two shorter Al−P interactions (Al1−P1 2.4140(14) Å, Al1−P2 2.5033(16) Å) and one long Al−P3 distance (Al1−P3 2.8431(16) Å). The structural differences and analogies to AlX compounds rationalize the differences in color and the NMR spectra of **10** and **11**. Importantly, aluminum cations in low redox states are highly elusive. Only in 2022, a first example of an Al(I) cation was reported by Krossing and coworkers [47, 70]. Moreover, cationic dialanes and the radical cation of a dialumene have been isolated recently [71, 72]. Overall, the results demonstrate the structural variability and flexibility in the coordination environment accessible with the novel Al complexes introduced in this study. Moreover, multiple charge states can be accessed, likely leading to the access of novel bonding motifs and reactivity at aluminum in the future.

**FIGURE 6:**
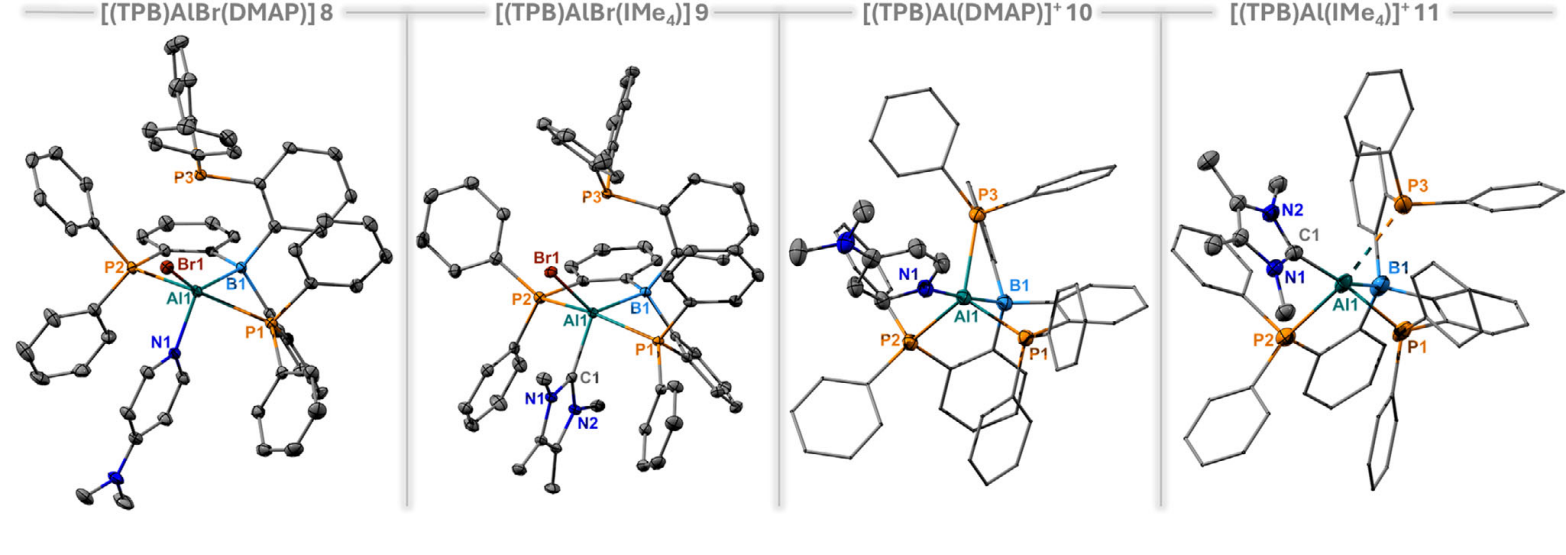
Molecular structures of **8**, **9**, **10**, and **11**. Thermal displacement of the ellipsoids was set at 50% probability. Weakly‐coordinating [B(C_6_F_5_)_4_]^−^ anion omitted for **10** and **11**. Hydrogen atoms are omitted for clarity. Since the unit cell of **10** shows two distinct molecules, the respective bond lengths are both listed. Selected distances (in Å) and angles (in °): **8**: Al1−Br1 2.3552(4), Al1−B1 2.1857(16), Al1−N1 1.9860(12), Al1−P1 2.5944(5), Al1−P2 2.5681(5), Al1−P3 3.5268(6), Br1−Al1−B1 140.08(4), N1−Al1−B1 117.59(6), P1−Al1−P2 163.63(2), P1−Al1−Br1 97.756(17); **9**: Al1−Br1 2.3563(6), Al1−B1 2.187(2), Al1−C1 2.084(2), Al1−P1 2.5757(8), Al1−P2 2.7212(8), Al1−P3 3.7184(8), Br1−Al1−B1 130.20(6), C1−Al1−B1 123.23(8), P1−Al1−P2 160.13(3), P1−Al1−Br1 104.56(2); **10**: Al1−B1 2.203(3)/2.188(2), Al1−N1 1.985(2)/1.975(2), Al1−P1 2.4617(9)/2.4788(9), Al1−P2 2.5290(8)/2.4818(9), Al1−P3 2.5559(9)/2.6324(9), N1−Al1−B1 175.98(10)/176.60(10), P1−Al1−P2 117.12(3)/109.83(3); **11**: Al1−C1 2.019(4), Al1−B1 2.144(4), Al1−P1 2.4140(14), Al1−P2 2.5033(16), Al1−P3 2.8431(16), C1−Al1−B1 147.84(16), P1−Al1−P2 106.93(5), P2−Al1−P3 154.59(5), P3−Al1−P1 91.53(5).

### 2.4 Al(I)Br‐Transfer

While previous experiments focused on exploring the coordination chemistry at the TPB‐Al platform, we aimed to investigate the use of **5** as an Al(I)Br transfer reagent. As discussed in the introduction, readily accessible and storable starting materials for Al(I) complexes are hitherto unknown, necessitating multi‐step procedures to prepare the corresponding Al complexes in low redox states, which often result in low yields. Addition of 3 equivalents of 2,2′‐bipyridine (bipy) to **5** resulted in an instantaneous color change of the solution from red to brown. Intriguingly, NMR spectroscopy revealed the quantitative formation of free TPB ligand (Scheme 3). Moreover, an intense EPR signal was detected. Crystallization of the solution yielded colorless crystals of the free TPB ligand next to black‐brown crystals of the [Al(bipy)_3_]^+^Br^−^ **12**. Unfortunately, separation of the ligand from **12** was not successful due to similar solubilities in common organic solvents. Moreover, the brown solution readily decolorizes and forms Al metal within one day.

**SCHEME 3:**
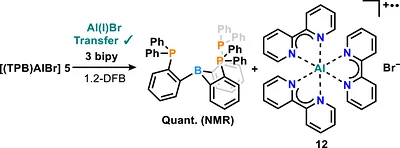
Reactivity of [(TPB)AlBr] towards bipy (= 2,2′‐bipyridine).

Previously, Wieghardt and co‐workers had performed a detailed structural analysis of 2,2′‐bipyridine metal complexes in various redox states. They discovered that the structural changes in the intrachelate C−C bond can be used as indicator of the redox state of the ligand. The respective C−C bond lengths measure to ∼1.49 Å for the neutral (bipy)^0^ ligand, to ∼1.43 Å for the monoanionic (bipy^•^)^−^ ligand and ∼1.38 Å for the dianionic (bipy)^2−^ [73]. In the molecular structure of **12** (Figure 7), two intrachelate C−C bond lengths clearly fit a single‐reduced (bipy^•^)^−^ ligand, while the third bond length is close to bond lengths observed neutral bipyridine ligands. Hence, based on the structural analysis, **12** is expected to represent a biradical formally bearing two single‐reduced bipy and one neutral bipyridine ligand. In solution, however, the fluxionality of the structure is expected to allow delocalization of electron density across all three 2,2′‐bipyridine ligands. Unfortunately, no half‐field signal could be observed at room temperature or at 100 K in a frozen 1,2‐DFB solution. Preliminary broken symmetry DFT‐studies at the B3LYP‐D4/def2‐TZVP//r2SCAN‐3c level of theory show that **12** exhibits a triplet ground‐state with a singlet‐triplet gap of 7.6 kJ mol^−1^. The closed‐shell singlet lies significantly higher in energy (Δ*E*_T‐CSS_ = +30.0 kJ mol^−1^). Previously, only neutral and anionic derivatives [Al(bipy^R^)_3_] (bipy^R^ = 4,4′‐Dimethyl‐2,2′‐bipyridine, 4,4′‐Di‐*tert*‐butyl‐2,2′‐bipyridine) [74] and [Al(bipy)_2_]^−^ [75]] have been reported. Intriguingly, [Al(bipy^R^)_3_] could only been accessed previously by Schnöckel starting from metastabile AlBr·(NEt_3_)*_n_* solutions in a disproportionation reaction [74]. The disproportionation reactions potentially occur since the metastable AlBr·(NEt_3_)*_n_* are composed of Al metal clusters rather than monomeric Al(I)Br units [41]. Hence, this reaction acts as proof‐of‐principle that our room‐temperature stable complexes can allow for transfer of a monomeric Al(I)Br (Figure 7).

**FIGURE 7:**
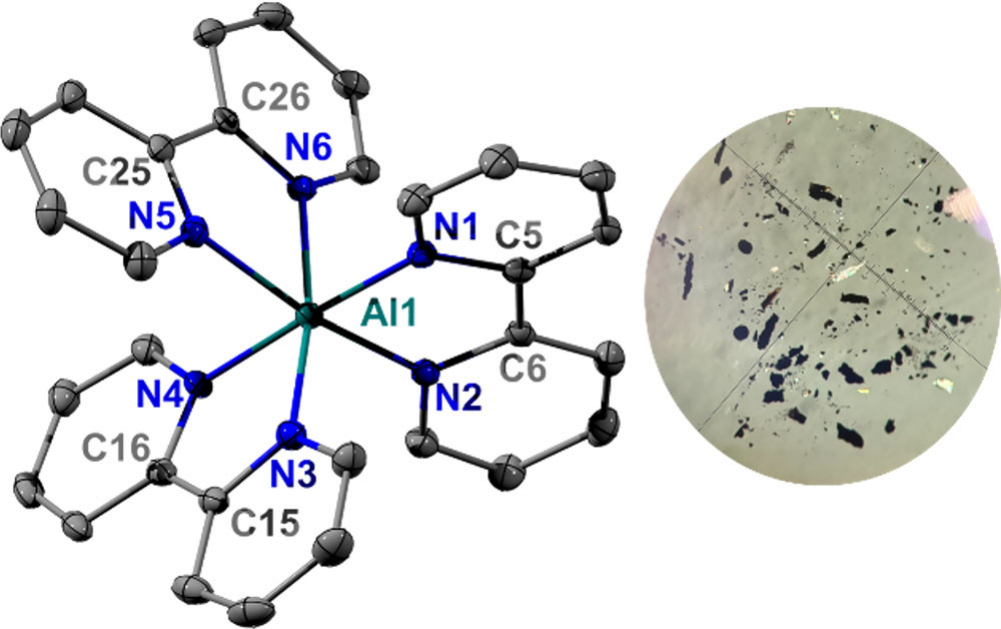
Left: Molecular structure of the cation in [Al(bipy)_3_]Br **12**. Thermal displacement of the ellipsoids was set at 50% probability. Hydrogen atoms and bromide counter‐ion are omitted for clarity. Selected distances (in Å) and angles (in °): Al1−N1 1.9838(14), Al1−N2 1.9778(14), Al1−N3 1.9642(14), Al1−N4 1.9940(14), Al1−N5 2.0723(14), Al1−N6 2.0500(14), C5−C6 1.433(2), C15−C16 1.424(2), C25−C26 1.471(2). Right: Picture of the crystals of **12**.

## 3 Conclusion

In summary, we present the straightforward preparation of aluminum(I) halide complexes of a tris(phosphine)borane ligand. The experimental and computational analyses reveal a unique electronic coordination environment at the penta‐coordinated aluminum atom, in which the lone pair on Al is stabilized in a covalent, polarized Al−B bond. The flexibility of the TPB‐Al platform is further shown by the preparation of an aluminum‐hydride complex via Br−H exchange and by successful coordination of the Al atom by small Lewis bases. Moreover, the successful bromide abstraction from the carbene‐adduct allowed for the successful isolation of elusive, monomeric Al cations in low redox states. Finally, the ability of the unique compounds to act as transfer agents of a monomeric Al(I)X fragments is evidenced in the reactivity of the bromide derivative towards redox‐active 2,2′‐bipyridine. Previously largely limited to transition‐metal chemistry, the present study highlights the potential of ambiphilic ligands in stabilizing elusive main‐group metal fragments in low redox states and in unlocking unprecedented coordination environments at aluminum. In the future, we aim to further explore the structure variability as well as Al(I)X transfer reactivity, targeting the discovery of novel bonding motifs and reactivity at abundant aluminum [76].

## Author Contributions

**Charlotte S. V. Weiß**: Data curation, investigation, visualization, writing – review and editing, writing – original draft. **Philipp Dabringhaus**: Conceptualization, methodology, investigation, supervision, writing – review and editing, writing – original draft, funding acquisition, data curation, visualization.

## Conflicts of Interest

The authors declare no conflicts of interest.

## Supporting information



The authors have cited additional references within the Supporting Information [66, 67, 77, 78, 79, 80, 81, 82, 83, 84, 85, 86, 87, 88, 89, 90, 91, 92, 93, 94, 95, 96, 97, 98, 99, 100, 101, 102, 103, 104, 105, 106, 107].**Supporting File**: anie73966‐sup‐0001‐SuppMat.pdf.

## Data Availability

The data that supports the findings of this study are available in the supplementary material of this article.
