# Supplementary material for: Ambiphilic Ligand Stabilization of Aluminum(I) Fragments: Halides, Cations, and a Hydride
Source: Angew Chem Int Ed Engl. 2026 Aug 5;65(40):e6333732. doi: 10.1002/anie.6333732 (PMC13618292; doi:10.1002/anie.6333732)
Supplement: Supplementary file 1 [file ANIE-65-e6333732-s001.pdf]

Supporting information for the article:

Ambiphilic Ligand Stabilization of Aluminum(I) Fragments: Halides, Cations and a Hydride

Charlotte S. V. Weiß and Philipp Dabringhaus*

Contents

S-1 Supporting experimental data 4

S-1.1 General experimental details and analytical methods 4

S-1.2 Synthesis of TPB-Ligand 6

S-1.3 Preparation of [(TPB)AlCl] 4 7

S-1.4 Preparation of [(TPB)AlBr] 5 13

S-1.5 Preparation of [(TPB)AlI] 6 19

S-1.7 NMR reaction: Preparation of [(TPB)AlCl] 4 via $[(TPB)AlCl]^{2+}([AlCl_4]^-)_2$ 1 25

S-1.8 NMR reaction: Preparation of [(TPB)AlBr] 5 from $[(TPB)AlBr]^{2+}([AlBr_4]^-)_2$ 2 28

S-1.9 NMR reaction: Preparation of [(TPB)AlI] 6 via $[(TPB)AlI]^{2+}([AlI_4]^-)_2$ 3 31

S-1.10 Attempted isolation of $[(TPB)AlCl]^{2+}([AlCl_4]^-)_2$ 1 34

S-1.11 Attempted isolation of $[(TPB)AlBr]^{2+}([AlBr_4]^-)_2$ 2 37

S-1.12 Attempted isolation of $[(TPB)AlI]^{2+}([AlI_4]^-)_2$ 3 40

S-1.13 Preparation of [(TPB)AlH] 7 43

S-1.14 Preparation of [(TPB)AlBr(DMAP)] 8 48

S-1.15 Preparation of $[(TPB)AlBr(IMe_4)]$ 9 52

S-1.16 Preparation of $[(TPB)Al(DMAP)]^+$ 10 55

S-1.17 NMR reaction: Preparation of $[(TPB)Al(IMe_4)]^+$ 11 via $[(TPB)AlBr(IMe_4)]$ 9 59

S-1.18 Preparation of $[(TPB)Al(IMe_4)]^+$ 11 62

S-1.19 Preparation of $[Al(bipy)_3]Br$ 12 65

S-2 Supporting quantum-chemical calculations 68

S-2.1 Computational details 68

S-2.2 RMSD analysis of experimental and optimized structures 69

S-2.3 Frontier Orbitals of [(TPB)AlX] complexes 71

S-2.4 QTAIM analysis of [(TPB)AlX] complexes 73

S-2.5 QTAIM analysis of [(TPB)AlH] complexes 78

S-2.6 NBO analysis of [(TPB)AlX] complexes 79

S-2.7 EDA-NOCV analysis of [(TPB)AlX] complexes 82

S-2.8 EDA-NOCV analysis of [(TPB)AlH] 88

S-2.9 EDA-NOCV analysis for hypothetical Ph_3B-AlBr 90

S-2.10 QTAIM, NBO and EDA-NOCV analysis for Cp*Al−B(C_6F_5)$_3$ I 92

S-2.11 QTAIM, NBO and EDA-NOCV analysis for Cp*Al−BFPh II 95

S-2.12 QTAIM, NBO and EDA-NOCV analyses of literature known R_2B−AlR_2 III 98

S-3 scXRD Tables 101

S-4 Literature 105

S-1 Supporting experimental data

S-1.1 General experimental details and analytical methods

All manipulations of air and/or water sensitive compounds were performed under an inert argon atmosphere using standard Schlenk or glovebox techniques. All glassware used in reactions has been stored overnight in an oven at 180 °C and was thoroughly flame-dried prior to usage.

Chemicals:

Bought and used as received: $AlCl_3$ (Sigma Aldrich), $AlBr_3$ (Fisher Scientific), AlI_3 (Sigma Aldrich), (2-Bromophenyl)diphenylphosphine (BLDPharm), *n*-Butyllithium (Sigma-Aldrich), BCl_3 (Fisher Scientific), dmap (Sigma Aldrich).

Prepared by literature procedures: Cobaltocene was prepared by students from the “Molekülchemie 2” lab course at Heidelberg University according to a modified literature procedure.[1] $Na[B(C_6F_5)_4]$[2] and IMe_4[3] were prepared according to literature procedures.

Solvents: Hexane, Et_2O, THF, and toluene were taken from the solvent purification system, degassed, and stored over activated 3 Å molecular sieves. 1,2-Difluorobenzene (Fluorochem, 1,2-DFB) distilled from CaH_2, and stored over activated 3 Å molecular sieves. *n*-Pentane and deuterated solvents were degassed and dried over activated 3 Å molecular sieves.

NMR spectroscopy: NMR samples were prepared in an inert-atmosphere glovebox in NMR tubes equipped with a gas-tight J. Young valve. ^{1}H, ^{7}Li, ^{11}B, ^{13}C, ^{19}F, ^{27}Al, and ^{31}P-NMR spectra were acquired either on a Bruker Avance II 400 MHz, Bruker Avance III 600 MHz, or a Bruker UltraShield 600 MHz. ^{1}H and ^{13}C NMR spectra are reported relative to TMS and were calibrated to residual solvent resonances.[4] Data analysis was performed using MestReNova 15.1.0 software.

EPR Spectroscopy: X-band EPR measurements were conducted at room temperature on a MiniScope MS400 (magenettech) spectrometer or on a Bruker ESP 300 spectrometer at 100 K.

IR spectroscopy: FT-IR spectra were recorded inside a glovebox with Cary 630 FTIR Spectrometer. The data were processed with the Opus software package. All IR spectra were normalized to 1 and the intensities are reported as follows: ≥ 0.8 = very strong (vs), ≥ 0.6 = strong (s), ≥ 0.4 = medium (m), ≥ 0.2 = weak (w), < 0.2 = very weak (vw).

UV-Vis spectroscopy: Solution UV-VIS spectra were recorded on a Cary 5000 UV-Vis spectrophotometer in quartz cuvettes (2 mm thickness) in toluene. The graphical representation in the manuscript was created with OriginPro 2021.

Single crystal X-ray analysis: Single crystal X-ray diffraction. Single crystal X-ray diffraction data were collected using a Bruker D8 VENTURE with PHOTONIII detector, four-circle diffractometer and INCOATEC Mo/Cu microsource. Crystals were selected under perfluoropolyether oil, mounted on 0.1 to 0.3 mm diameter CryoLoops and quench-cooled using an Oxford Cryostream 800 open flow N_2 cooling device.[4] Data were collected at 100 K using monochromated Mo Kα radiation (λ = 0.71073 Å). Data processing was done with Bruker APEX5 including SAINT for integration SADABS for multi-scan absorption correction.[5] Structure solution was performed by direct methods with SHELXT and refined by full-matrix least squares methods against F^2 using SHELXL.[6,7] ShelXle was used as visualization tool during refinement.[8] Disordering of fragments was done with the help of the implemented DSR tool.[9,10] Graphical representations have been prepared using Diamond. Finalization of gathered data was done using the final CIF tool.[11] HAR refinement was performed using the NoSphereA2[12,13] procedure in Olex2.

S-1.2 Synthesis of TPB-Ligand

The ligand was prepared according to a modified literature procedure.[14]

Step 1: (2-Bromophenyl)diphenylphosphine (3.00 g, 8.79 mmol) was dissolved in Et_2O (approx. 30 mL). *n*BuLi (2.5 M in Hexanes, 3.7 mL, 9.2 mmol, 1.05 equiv) was added at room temperature. The mixture was stirred for 2 h at room temperature and solvent was evaporated under vacuum. The residue was washed with pentane (10 mL) yielding the ether-adduct of (2-Lithiophenyl)diphenylphosphine as off-white solid (2.72 g, 7.95 mmol, 90 %).

Step 2: (2-Lithiophenyl)diphenylphosphine (2.72 g, 7.95 mmol) was weighed into a large pressure vessel (120 mL), and toluene (25 mL) was added. The mixture was cooled to −78 °C, followed by dropwise addition of BCl_3 (1 M in heptane, 2.6 mL, 2.6 mmol, 0.3 equiv). The mixture was stirred for 15 min at −78 °C and for 1 h at room temperature. Then, the reaction mixture was refluxed overnight, yielding a yellow solution and a colorless precipitate. The solvent was evaporated under vacuum. The residue was dissolved in DCM and filtered. Subsequently, the solvent was removed under vacuum, and the residue was washed with cold DCM (6 mL), Et_2O (6 mL), and pentane (10 mL). The TPB ligand was isolated as a slightly yellow solid (750 mg, 944 μmol, 36 %). Another batch (480 mg, 604 μmol, 23 %) could be obtained by drying the filtrate under vacuum, dissolving the solid in minimal amounts of DCM, and layering the solution with pentane at −36 °C.

The observed NMR signals match the literature.[14] scXRD-quality crystals of the ligand could be obtained by layering a benzene solution with pentane at room temperature.

S-1.3 Preparation of [(TPB)AlCl] 4

Procedure: TPB (242 mg, 305 μmol) and $AlCl_3$ (124 mg, 929 μmol, 3.05 equiv.) were charged into a vial, into which 1,2-DFB (2 mL) was added. The mixture was stirred overnight. Cobaltocene (118 mg, 624 μmol, 2.05 equiv.) was added, whereby the yellow solution immediately turned dark red. The mixture was stirred for 30 min. Then, the mixture was filtered, and the solid was washed with benzene (5 mL) and toluene (3 mL). The solvent of the combined filtrates was removed in vacuo. The obtained residue was washed with acetonitrile (3 mL) and pentane (10 mL). Crystals of the title compound **4** were obtained by layering a solution of the residue in approximately 3 mL THF with 5 mL pentane (67.0 mg, 78.2 μmol, 26 %).

^{1}H NMR spectrum (400.20 MHz, C_6D_6, 298 K): δ = 6.68-6.77 (m, 3H, −P(C_6H_4)B), 6.77-6.86 (m, 8H, $-PPh_2$), 6.92-6.95 (m, 3H, −P(C_6H_4)B), 6.95-7.07 (m, 10H, $-PPh_2$), 7.07-7.11 (m, 3H, −P(C_6H_4)B), 7.20-7.38 (m, 12H, $-PPh_2$), 7.58-7.60 (m, 3H, −P(C_6H_4)B) ppm.

^{13}C NMR spectrum (100.63 MHz, C_6D_6, 298 K): δ = 123.8 (−P(C_6H_4)B), 128.0 (−P(C_6H_4)B), 128.8 ($-PPh_2$), 132.5 (−P(C_6H_4)B), 134.2 ($-PPh_2$), 136.2 (−P(C_6H_4)B) ppm.

^{11}B NMR spectrum (128.40 MHz, C_6D_6, 298 K): δ = -7.5 (q, $^{3}J_{B,P}$ = 40.2 Hz) ppm.

^{31}P NMR spectrum (162.00, C_6D_6, 298 K): δ = -23.0 (q, $^{3}J_{B,P}$ = 35.0 Hz) ppm.

IR spectrum (128 scans, ATR, Diamond): ν = 691 (vs), 738 (vs), 742 (vs), 755 (vw), 765 (vw), 844 (vw), 875 (vw), 916 (vw), 998 (vw), 1027 (vw), 1069 (vw), 1093 (w), 1158 (vw), 1182 (vw), 1240 (vw), 1262 (vw), 1307 (vw), 1420 (vw), 1434 (m), 1479 (vw), 1540 (vw), 1569 (vw), 1585 (vw), 3033 (vw), 3053 (vw) cm^{-1}.

UV-Vis (toluene, 500 µM, RT): λ_{max} = 356 (ε = 8934 $Lmol^{-1}cm^{-1}$), 453 (ε = 2550 $Lmol^{-1}cm^{-1}$) nm.

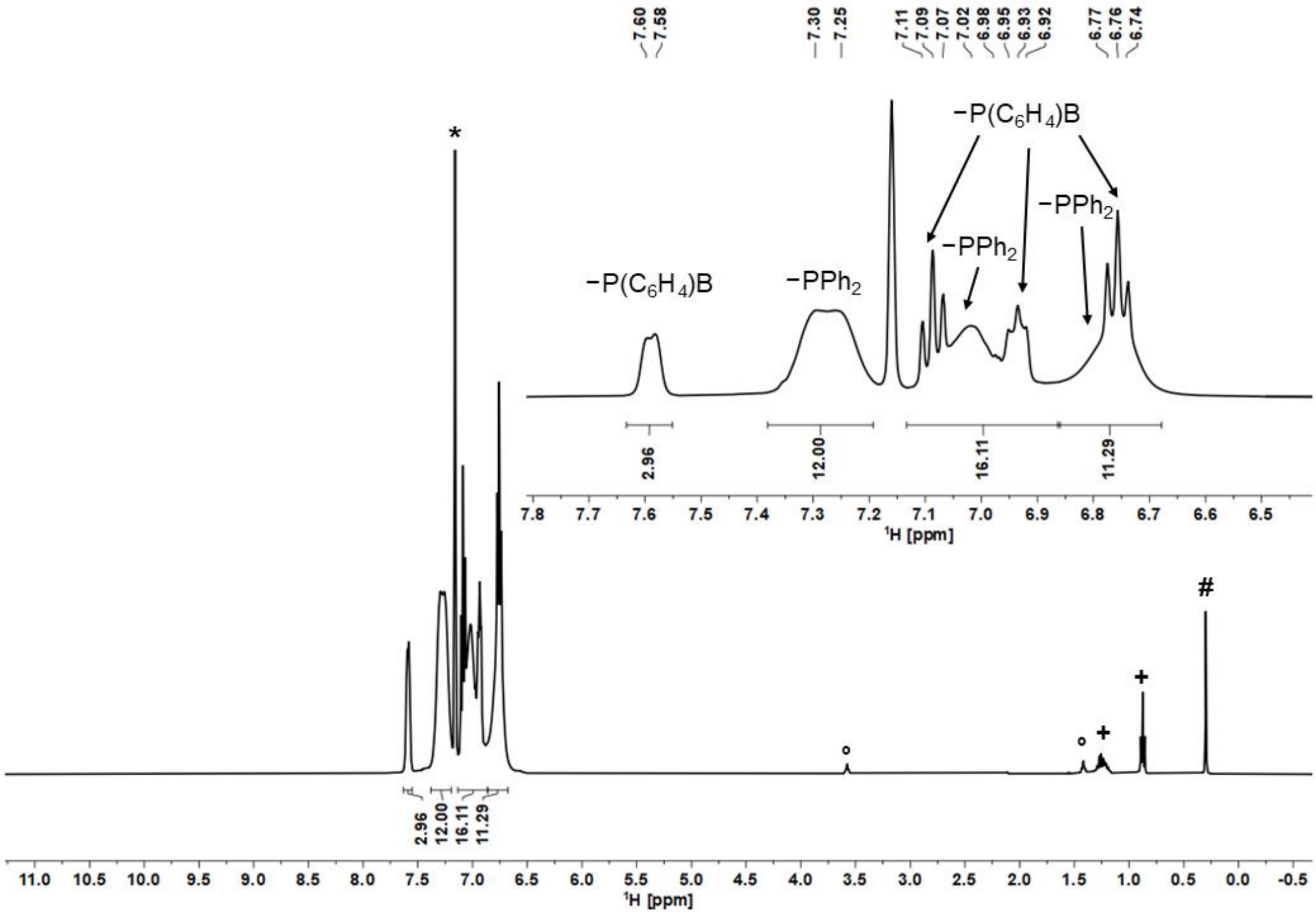

S-Figure 1: ^{1}H NMR spectrum (400.20 MHz, C_6D_6, 298 K) of [(TPB)AlCl] **4**. C_6D_6 is marked with *, silicon grease with #, residual pentane with +, and residual thf with °.

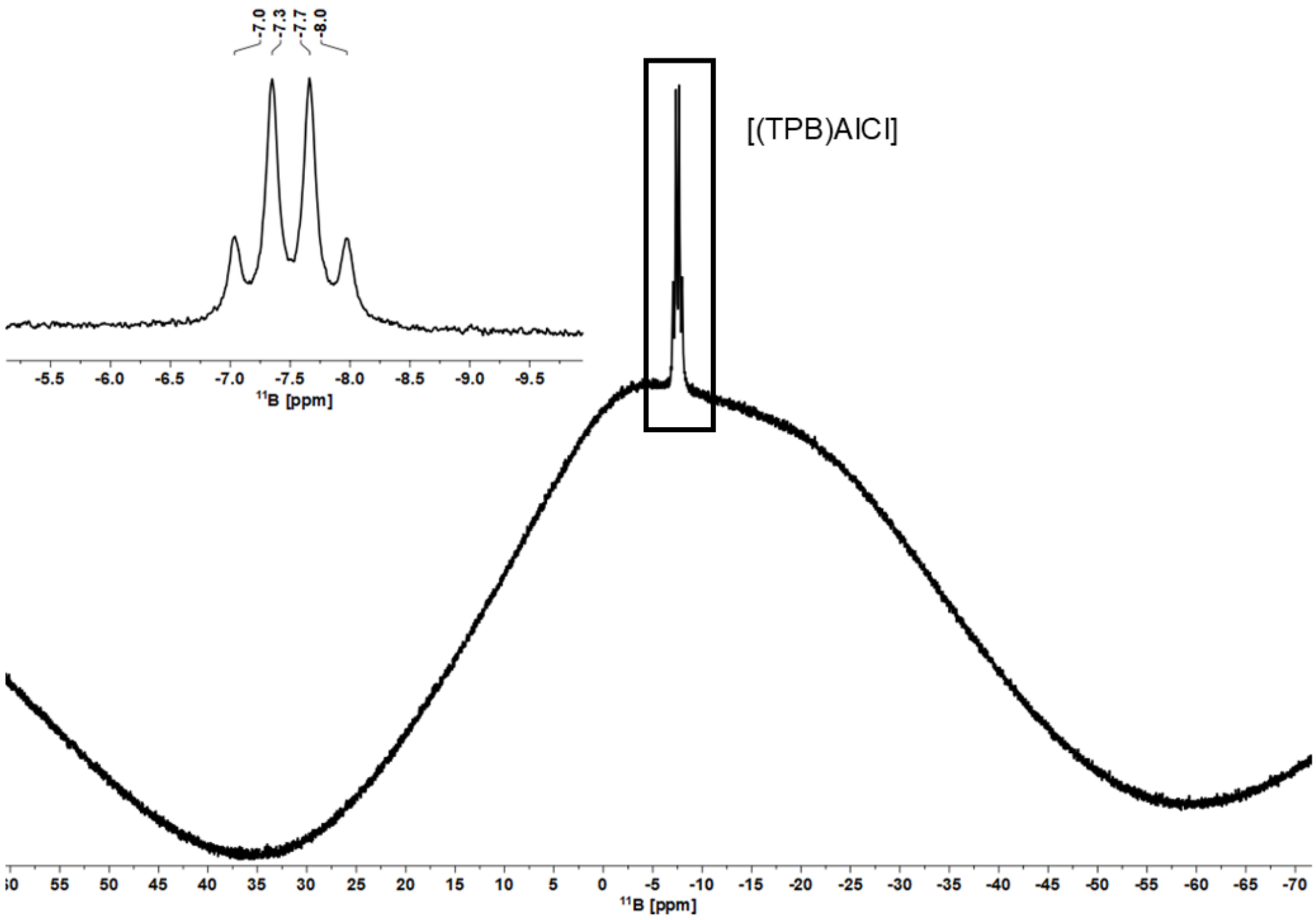

S-Figure 2: ^{11}B NMR spectrum (128.40 MHz, C_6D_6, 298 K) of [(TPB)AlCl] **4**.

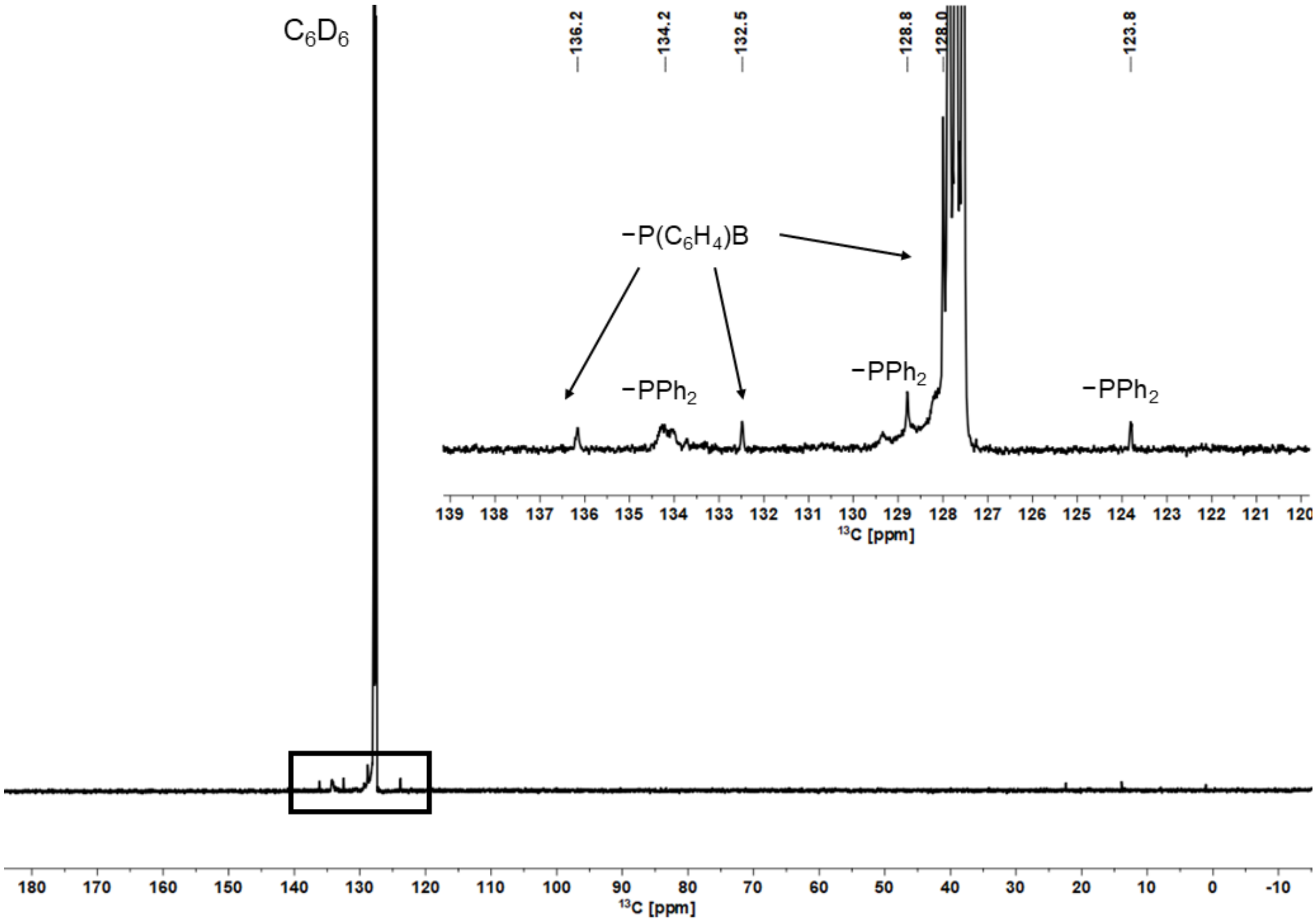

S-Figure 3: ^{13}C NMR spectrum (150.93 MHz, C_6D_6, 298 K) of [(TPB)AlCl] **4**.

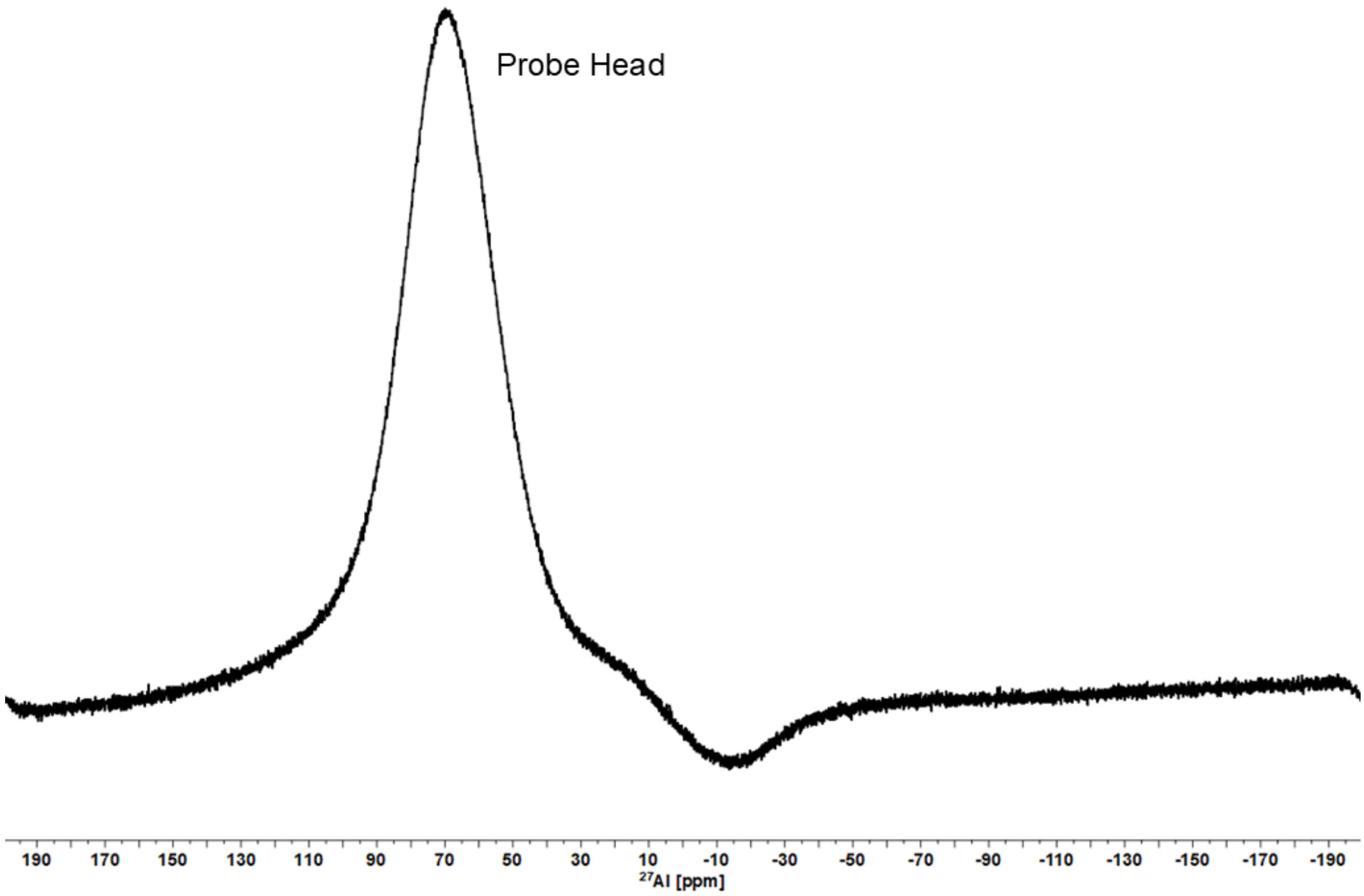

S-Figure 4: ^{27}Al NMR spectrum (104.28 MHz, C_6D_6, 298 K) of [(TPB)AlCl] **4**.

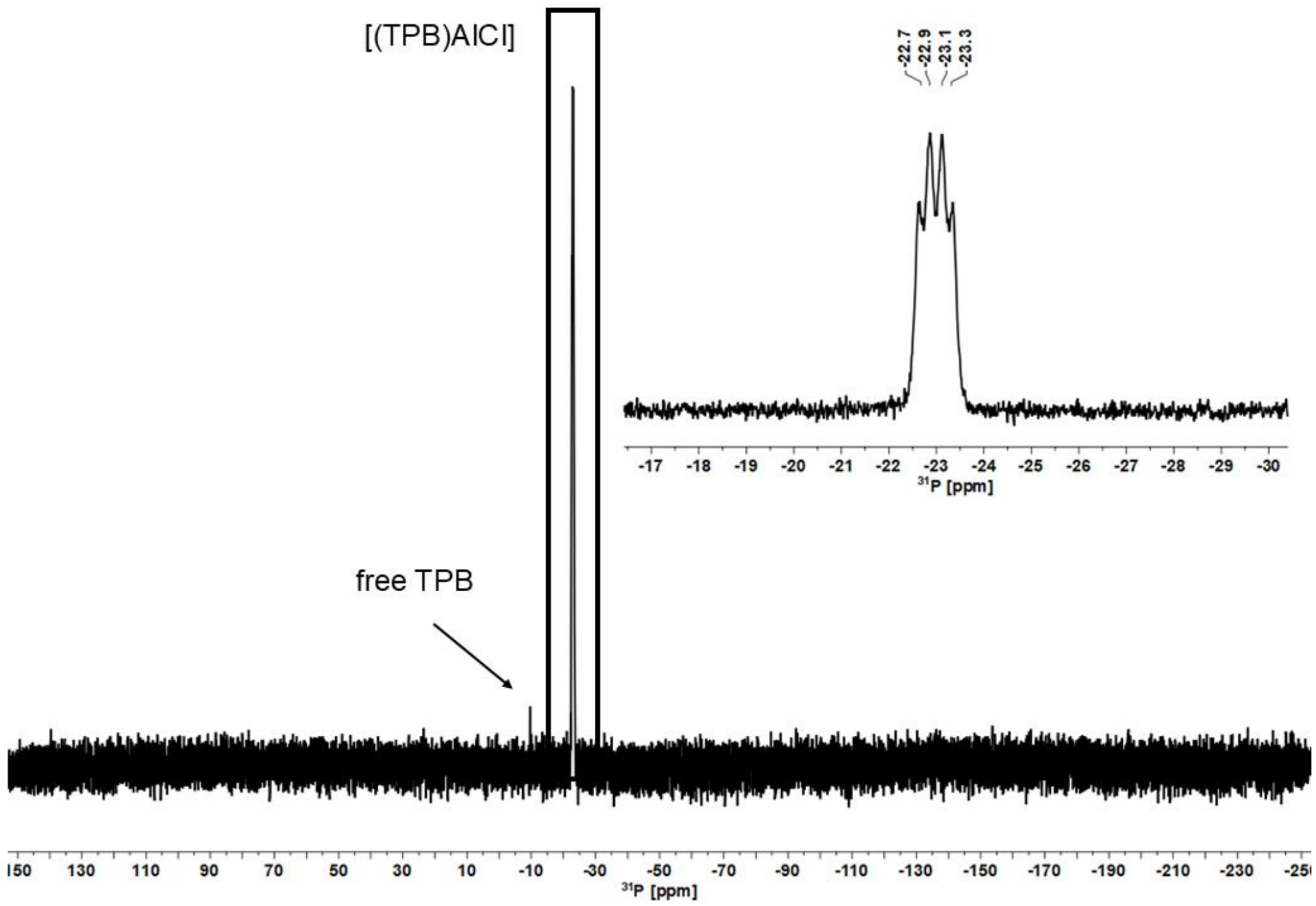

S-Figure 5: ^{31}P NMR spectrum (162.00 MHz, C_6D_6, 298 K) of [(TPB)AlCl] **4**.

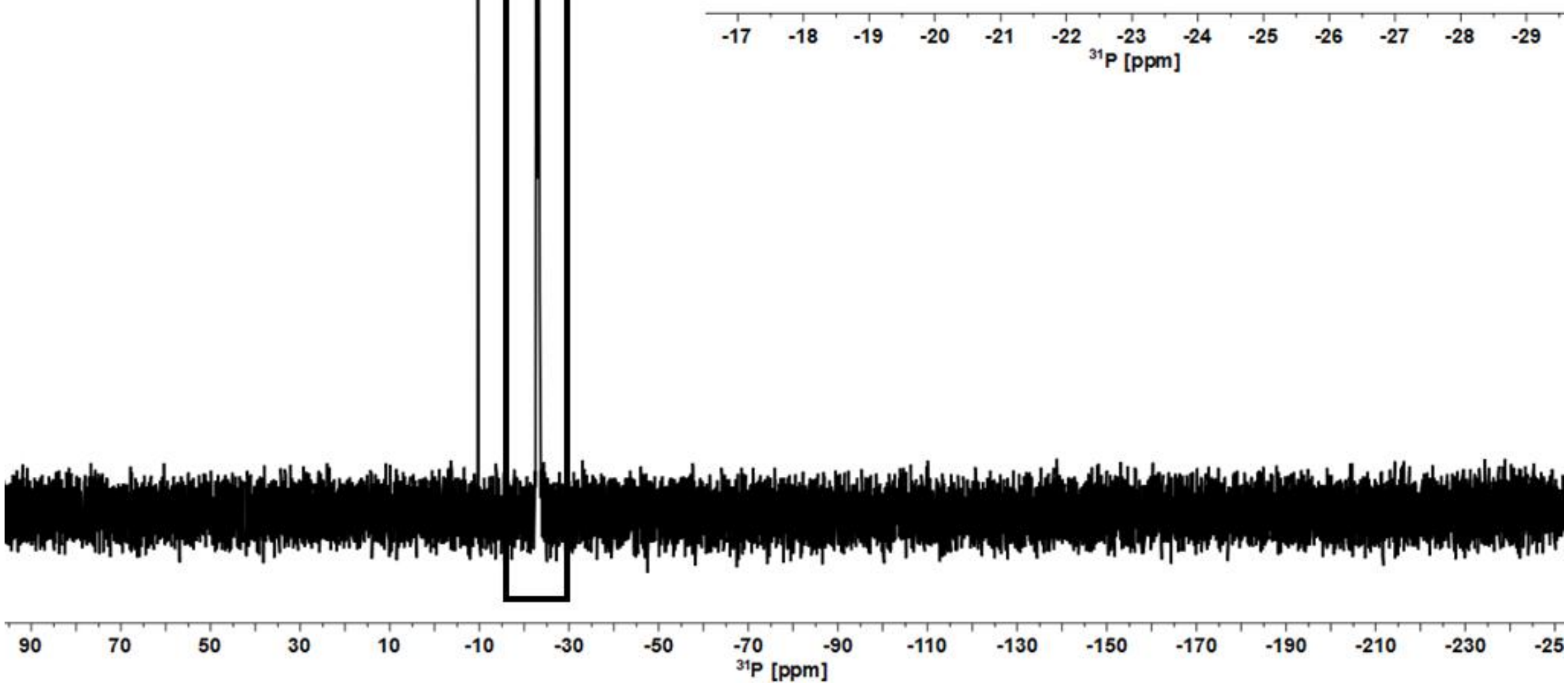

S-Figure 6: $^{31}P\{^1H\}$ NMR spectrum (162.00 MHz, C_6D_6, 298 K) of [(TPB)AlCl] **4**.

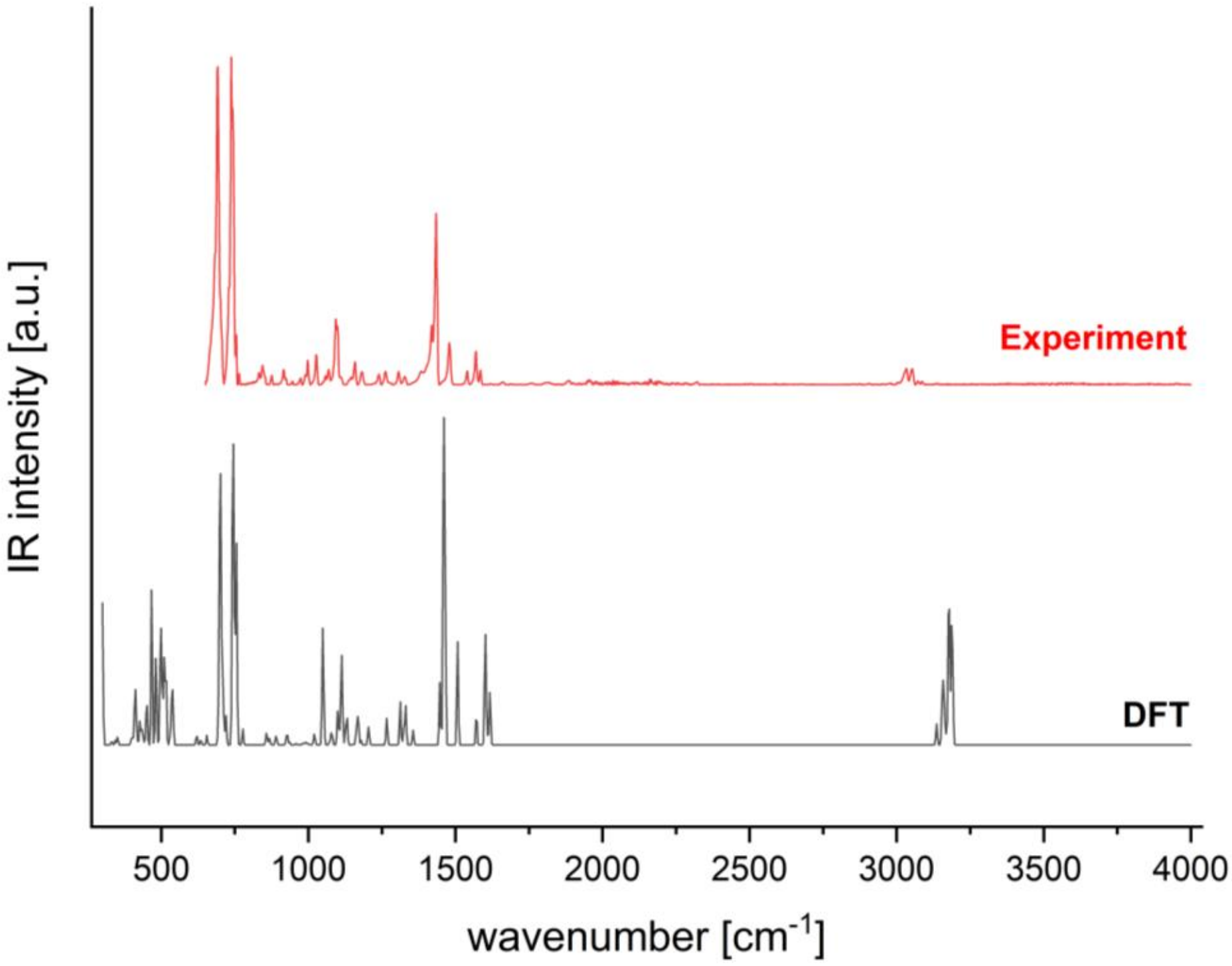

S-Figure 7: Comparison of the experimental IR spectrum (ATR, red, top) of [(TPB)AlCl] **4** with the computed IR spectra of **4** (black, bottom). Structures optimized at r^{2}SCAN-3c level of DFT with CPCM(fluorobenzene).

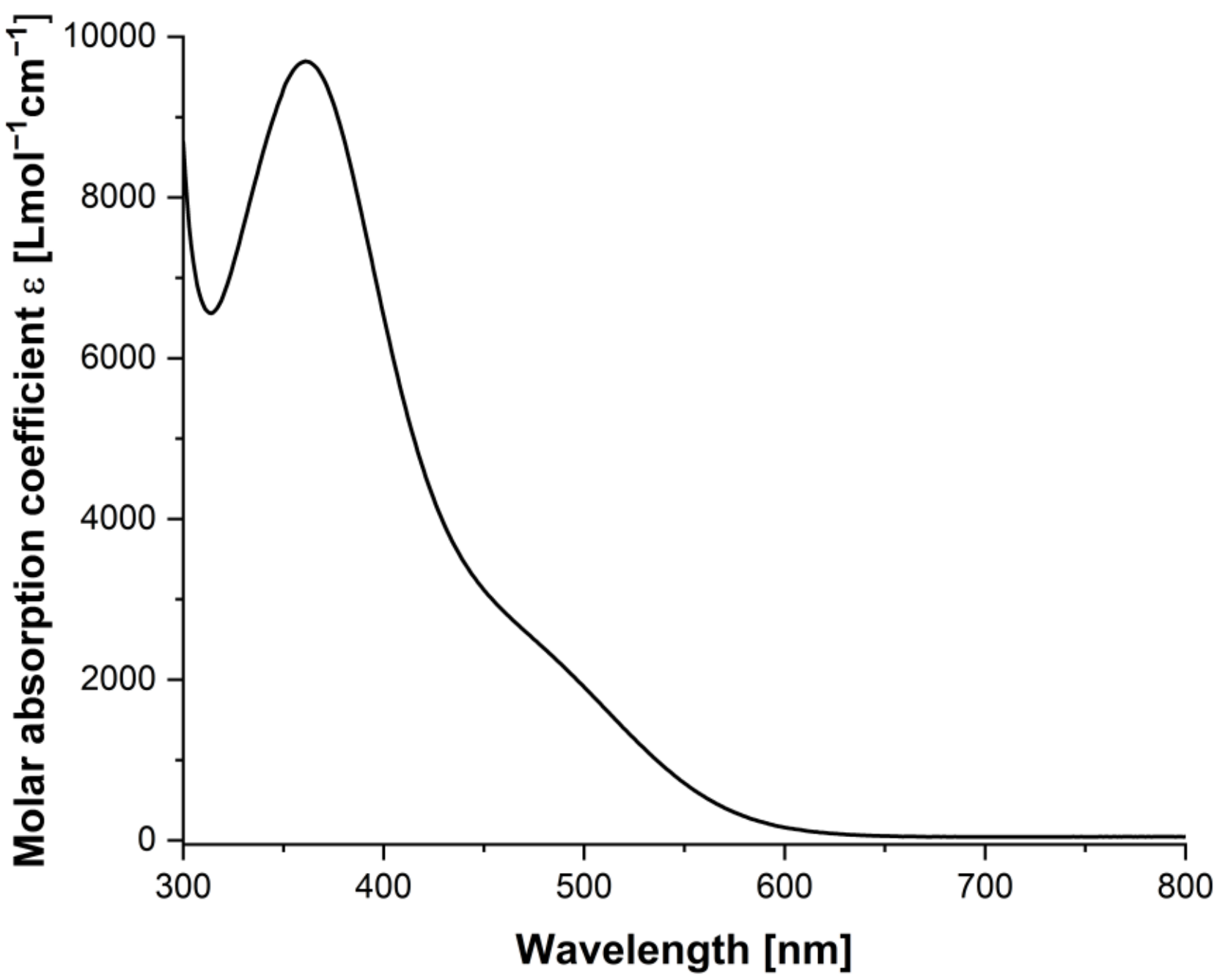

S-Figure 8: UV-Vis spectrum (toluene, 500 µM, RT) of [(TPB)AlCl] **4**.

Peak Analysis

Data Set:[A500mM1]500mM_1!F Date:01/05/2026

BaseLine:Constant

Chi^2=6.18396E+02 Adj. R-Square=9.99935E-01 # of Data Points=471

SS=2.86936E+05 Degrees of Freedom=464

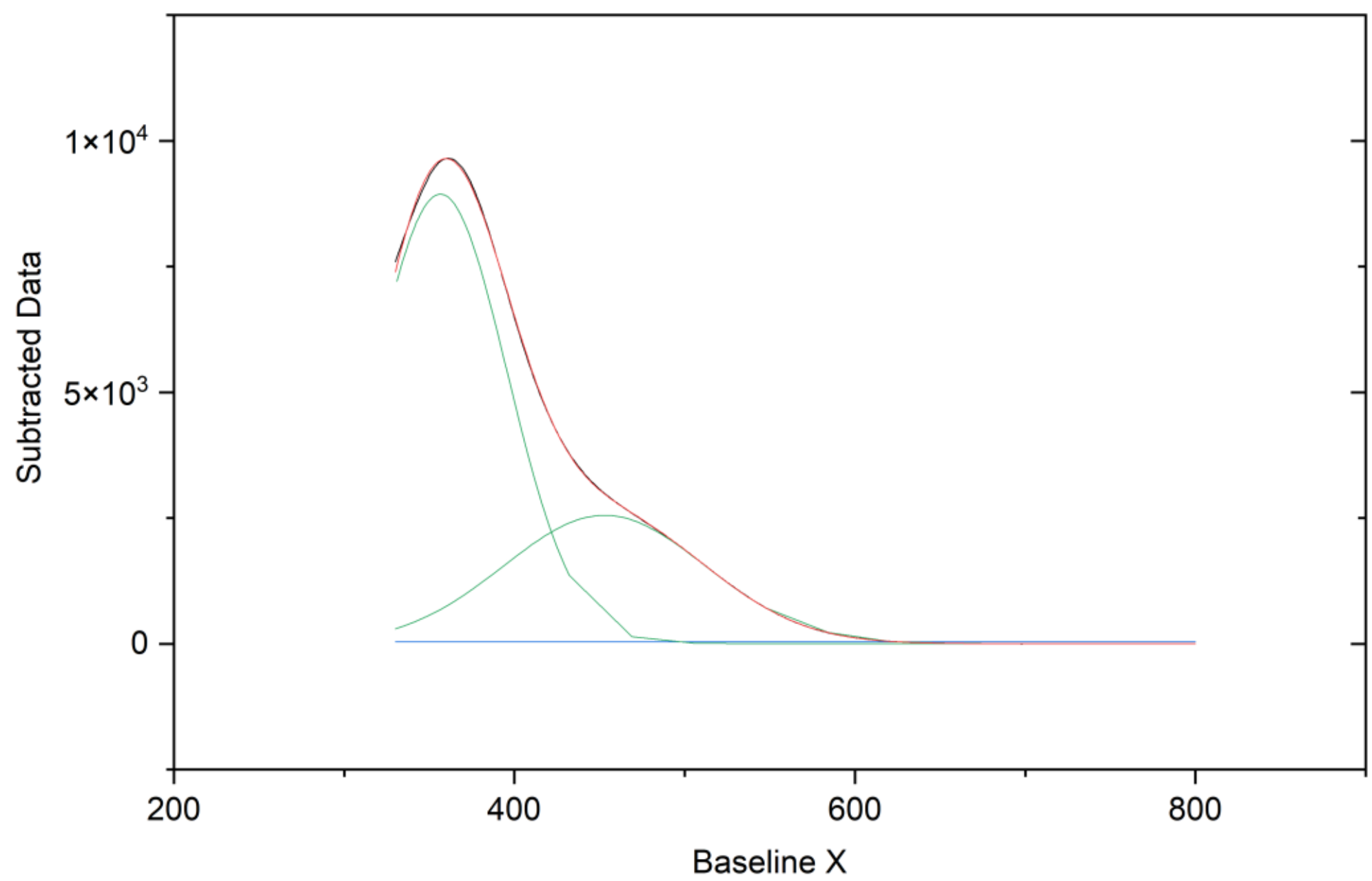

Fitting Results

Peak Index	Peak Type	Area Intg	FWHM	Max Height	Center Grvty	Area IntgP
1	GaussAmp	656830.68937	91.90696	8934.22438	356.5102	63.88089
2	GaussAmp	371380.91239	139.45621	2550.21935	452.89212	36.11911

S-Figure 9: Fit of UV-Vis spectrum (toluene, 500 µM, RT) of [(TPB)AlCl] **4**.

S-1.4 Preparation of [(TPB)AlBr] 5

Procedure 1: TPB (100 mg, 126 μmol) and $AlBr_3$ (102 mg, 384 μmol, 3.05 equiv.) were weighed in a vial and 1,2-DFB (2 mL) was added. The mixture was stirred for 15 min yielding a yellow solution. Cobaltocene (48.8 mg, 258 μmol, 2.05 equiv.) was added, resulting in a color change of the solution to dark red. The solution was stirred for 15 min, and the solvent was then evaporated under vacuum. The residue was dissolved in benzene (5 mL) and filtered through a frit. The filter cake was washed thoroughly with benzene. The filtrate and combined washing solutions were dried in vaccum. The residue was washed with acetonitrile (5 mL) and pentane (5 mL) and dried thoroughly. Compound **5** was isolated as a red powder (50.0 mg, 55.5 μmol, 44 %).

Procedure 2: TPB (250 mg, 315 μmol) and $AlBr_3$ (252 mg, 944 μmol, 3.00 equiv.) were weighed in a vial and 1,2-DFB (3 mL) was added. The mixture was stirred for 15 min, yielding a yellow solution. Cobaltocene (119 mg, 629 μmol, 2.00 equiv.) was added, resulting in a color change of the solution to dark red. The solution was stirred for 1 h, and the solvent was then removed under reduced pressure. The residue was dissolved in benzene (5 mL) and filtered through a frit. The filtrate was dried in vaccum. The red residue dissolved in THF (5 mL) and layered with pentane (8 mL) at −40 °C in the glovebox freezer. After 2 days, compound **5** was isolated as a red crystals (197 mg, 129 μmol, 69 %).

^{1}H NMR spectrum (400.17 MHz, C_6D_6, 298 K): δ = 6.65-6.78 (m, 3H, $-PPh_2$), 6.78-6.84 (m, 8H, $-P(C_6H_4)B$), 6.87-6.94 (m, 3H, $-P(C_6H_4)B$), 6.95-7.07 (m, 10H, $-PPh_2$), 7.07-7.14 (m, 3H, $-P(C_6H_4)B$), 7.22-7.41 (m, 12H, $-PPh_2$), 7.57-7.66 (m, 3H, $-P(C_6H_4)B$) ppm.

^{13}C NMR spectrum (150.93 MHz, C_6D_6, 298 K): δ = 123.8 ($-P(C_6H_4)B$), 128.0 ($-PPh_2$), 128.1 ($-P(C_6H_4)B$), 128.8 ($-PPh_2$), 132.6 ($-P(C_6H_4)B$), 134.2 ($-PPh_2$), 136.0 ($-P(C_6H_4)B$) ppm.

^{11}B{^{1}H} NMR spectrum (128.40 MHz, C_6D_6, 298 K): δ = -7.8 (q, $^3J_{B,P}$ = 41.0 Hz) ppm.

^{31}P{^{1}H} NMR spectrum (161.99, C_6D_6, 298 K): δ = -23.3 (q, $^3J_{B,P}$ = 41.0 Hz) ppm.

IR spectrum (128 scans, ATR, Diamond): ν = 689 (m), 726 (vs), 742 (m), 798 (m), 829 (vw), 969 (vs), 997 (vw), 1025 (vw), 1047 (w), 1095 (m), 1163 (m), 1213 (vs), 1238 (s), 1273 (m), 1296 (w), 1350 (vw), 1438 (vw), 1482 (vw), 1506 (vw), 1587 (vw) cm^{-1}.

UV-Vis (toluene, 500 µM, RT): λ_{max} = 356 (ε = 8608 $Lmol^{-1}cm^{-1}$), 458 (ε = 2342 $Lmol^{-1}cm^{-1}$) nm.

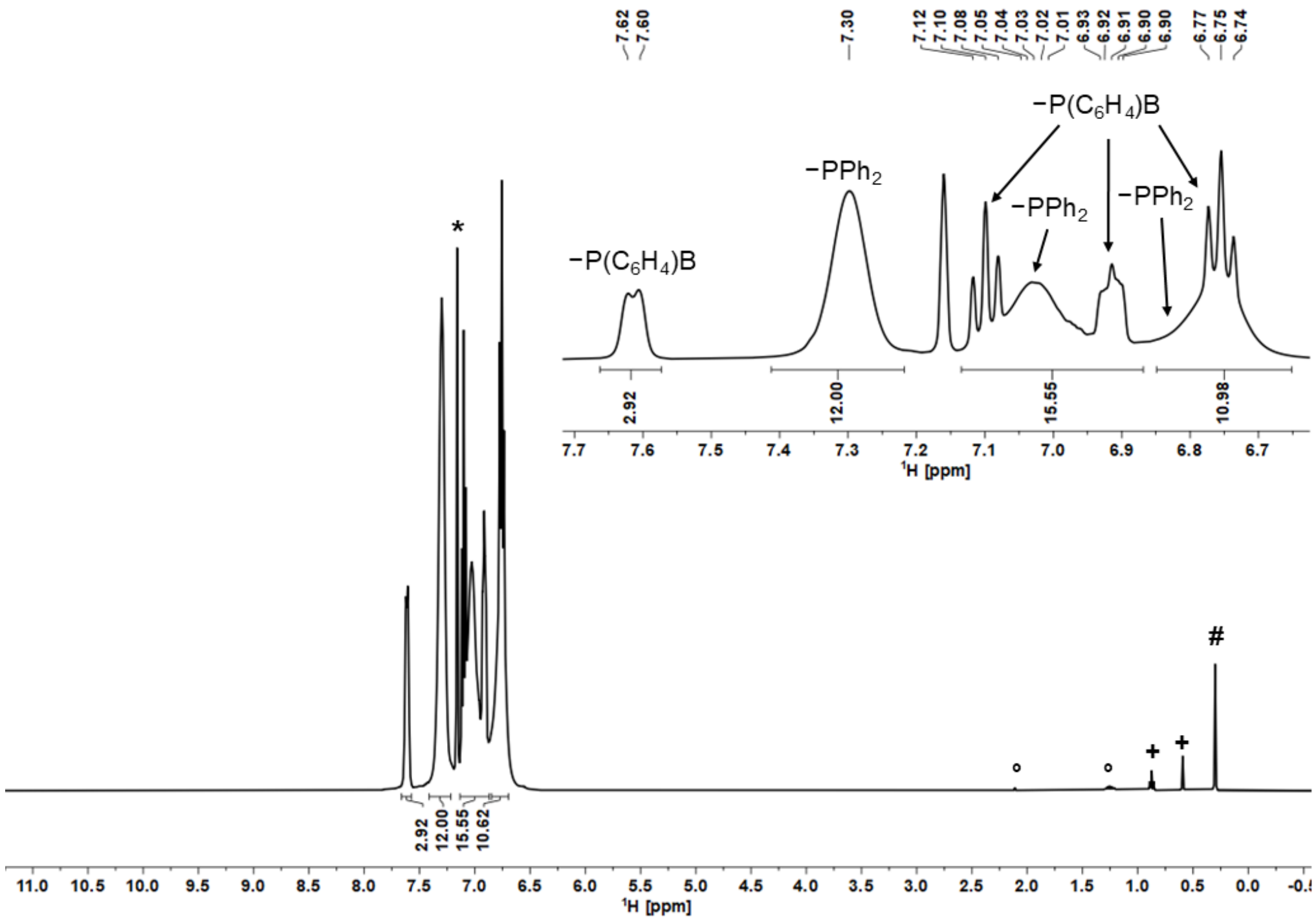

S-Figure 10: ^{1}H NMR spectrum (400.20 MHz, C_6D_6, 298 K) of [(TPB)AlBr] **5**. C_6D_6 is marked with *, silicon grease with #, residual pentane with +, and residual thf with °.

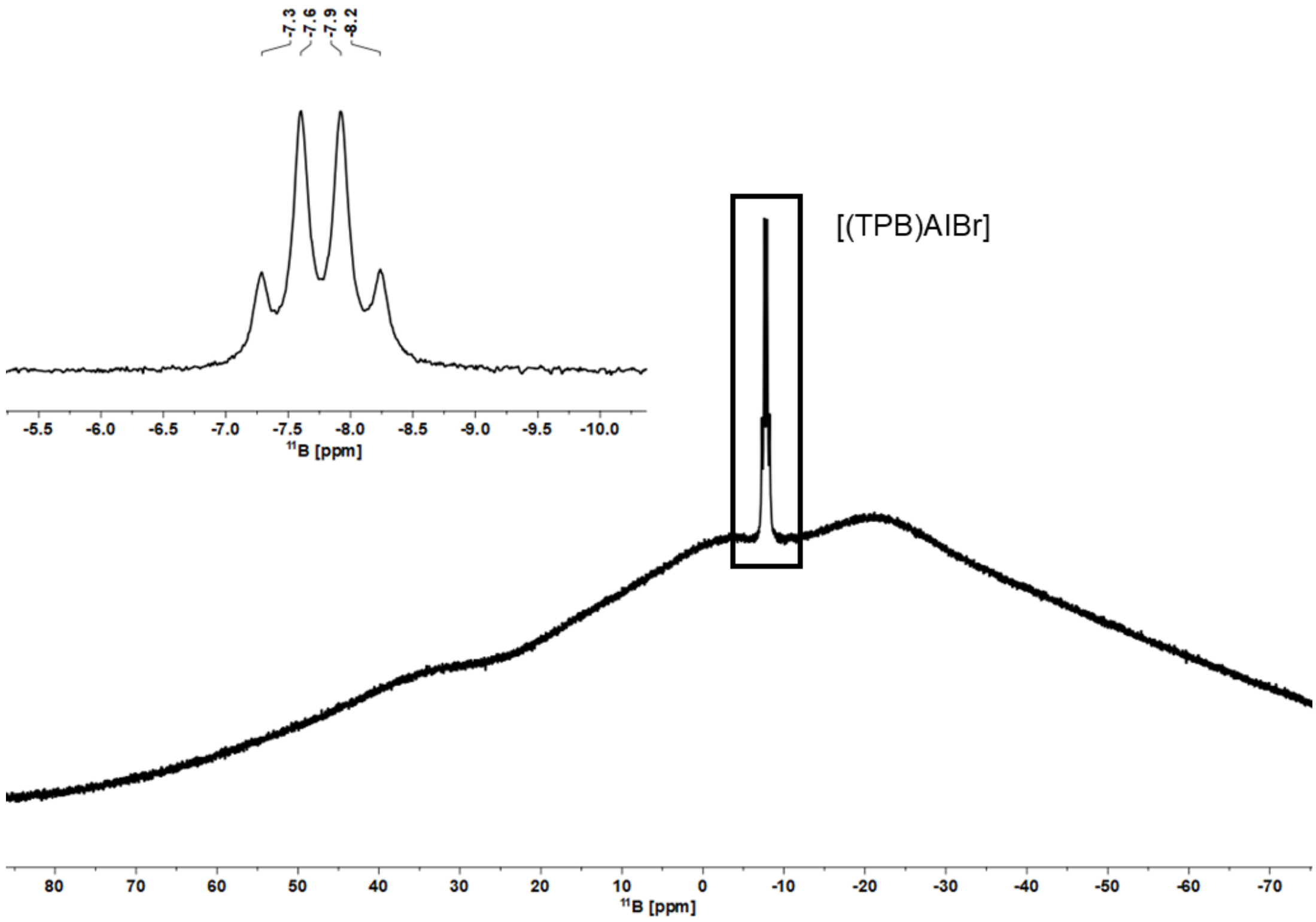

S-Figure 11: ^{11}B NMR spectrum (128.30 MHz, C_6D_6, 298 K) of [(TPB)AlBr] **5**.

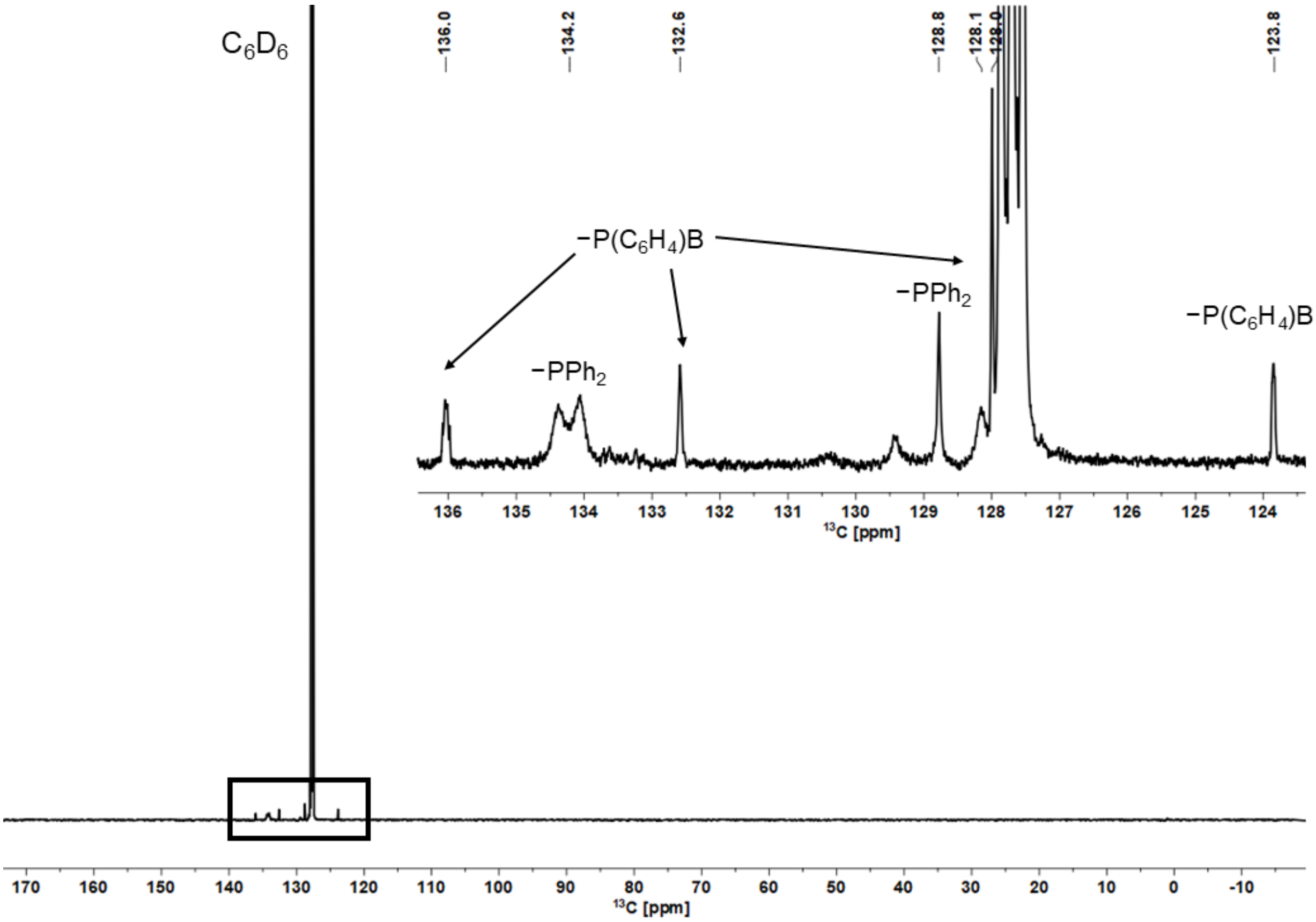

S-Figure 12: ^{13}C NMR spectrum (150.93 MHz, C_6D_6, 298 K) of [(TPB)AlBr] **5**.

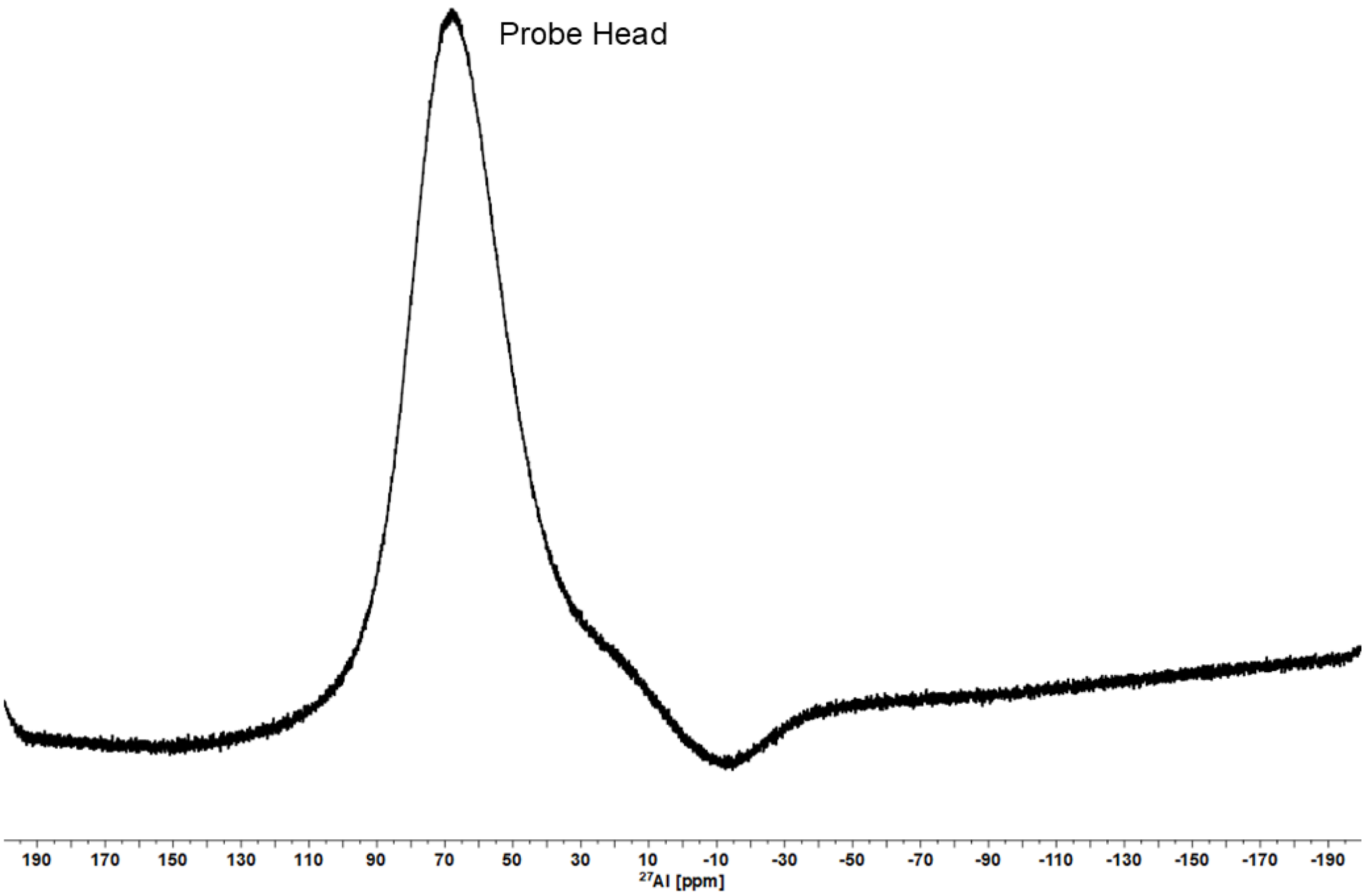

S-Figure 13: ^{27}Al NMR spectrum (104.20 MHz, C_6D_6, 298 K) of [(TPB)AlBr] **5**.

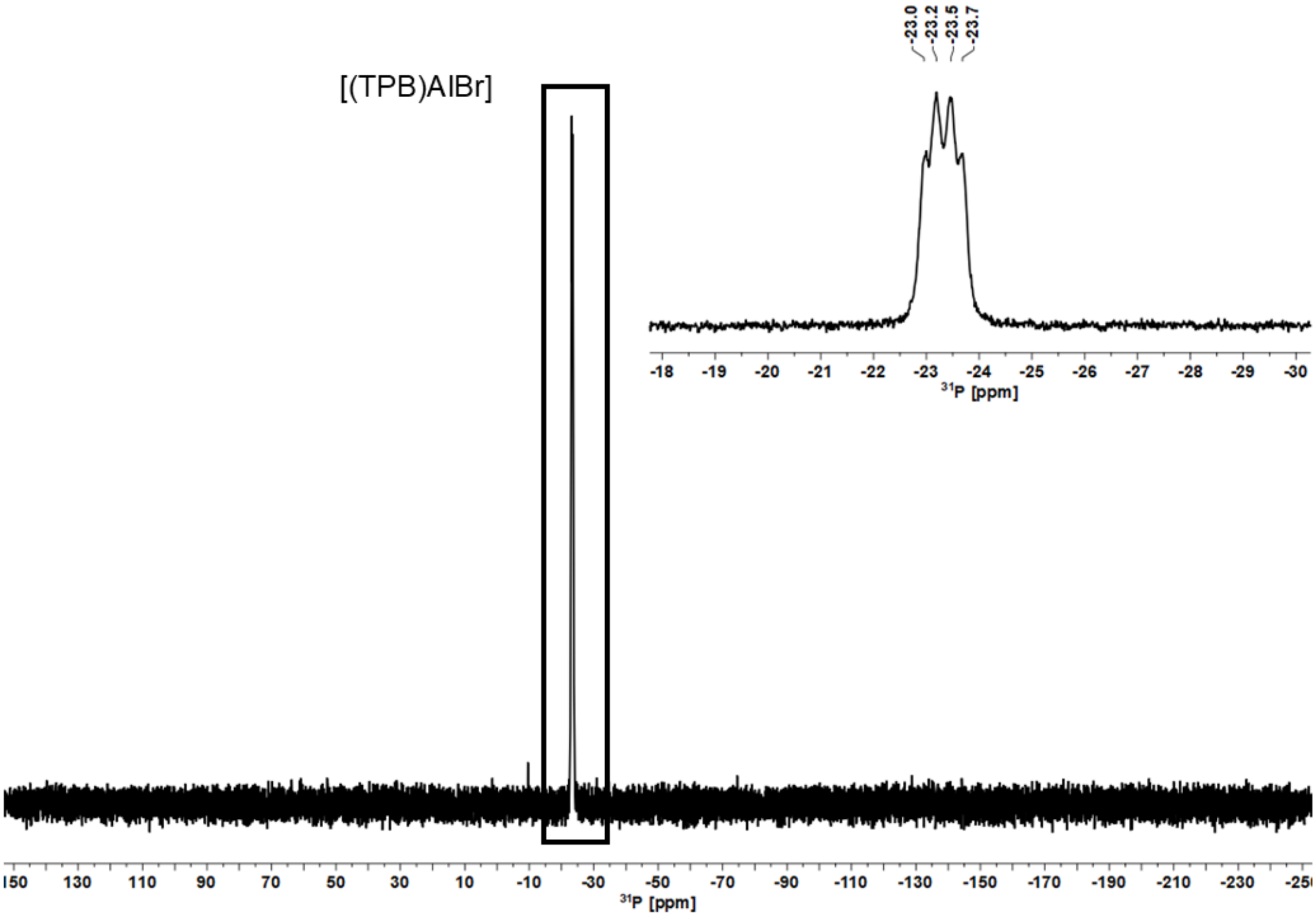

S-Figure 14: ^{31}P NMR spectrum (162.00 MHz, C_6H_6, 298 K) of [(TPB)AlBr] **5**.

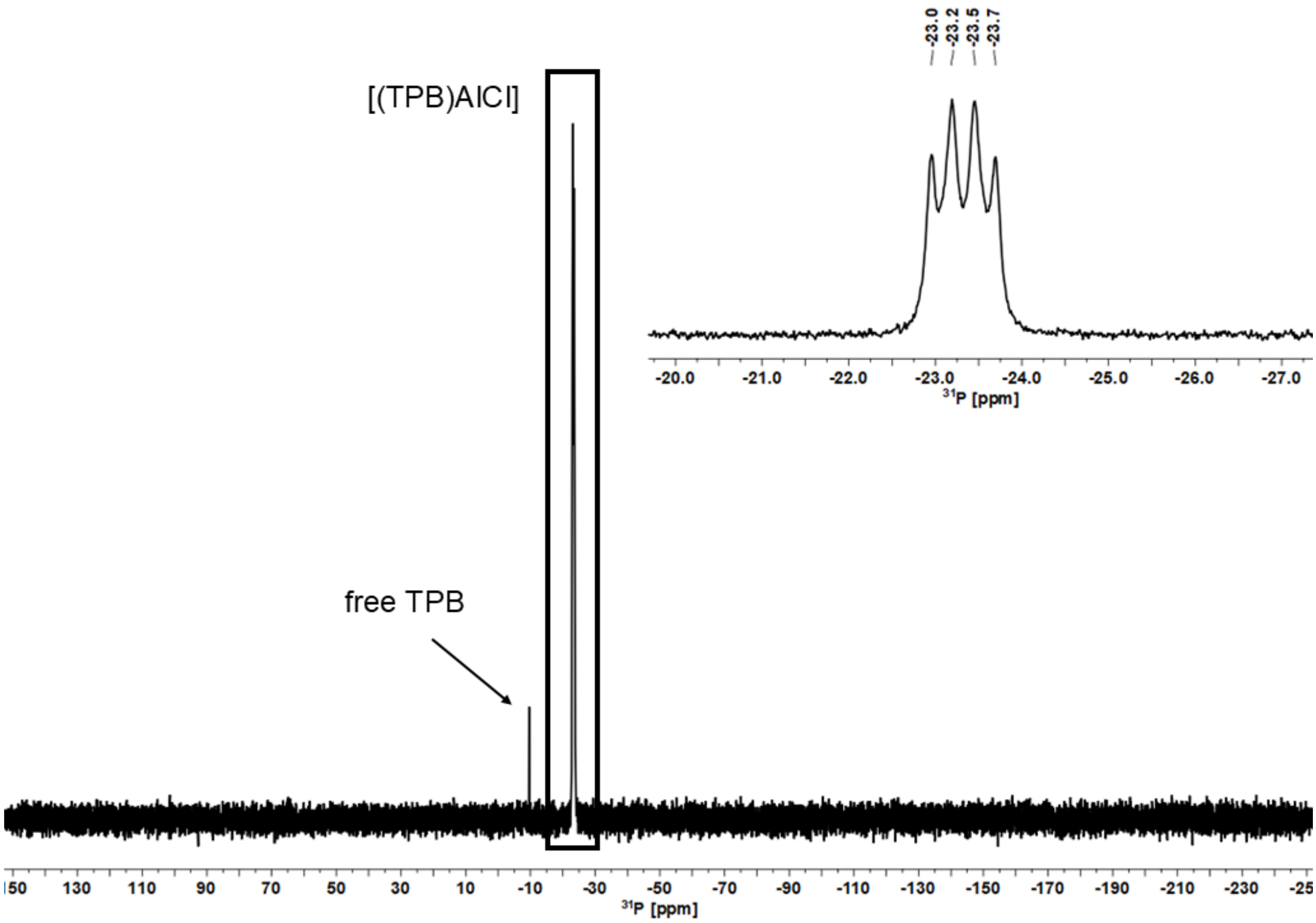

S-Figure 15: ^{31}P{^{1}H} NMR spectrum (162.00 MHz, C_6H_6, 298 K) of [(TPB)AlBr] **5**.

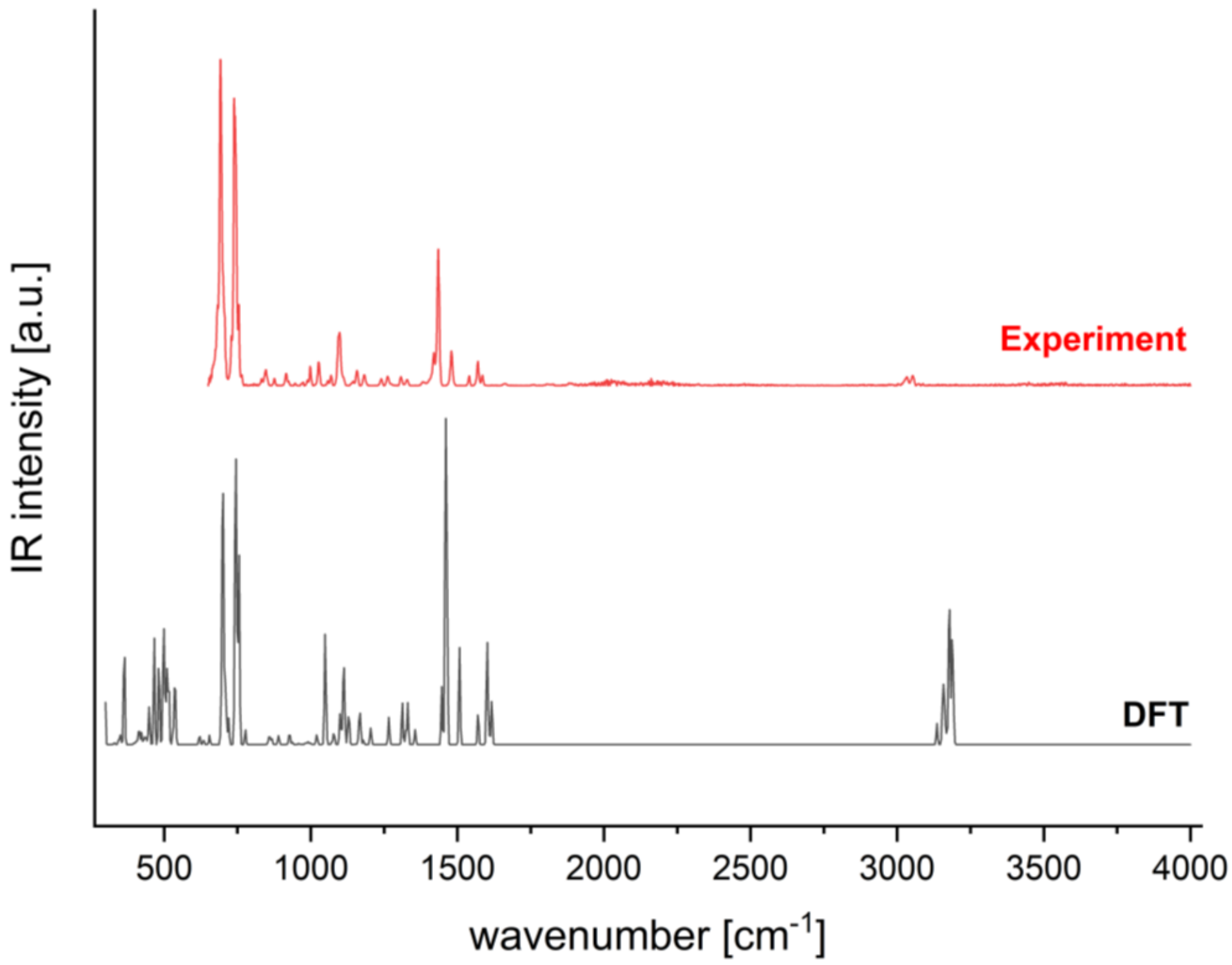

S-Figure 16: Comparison of the experimental IR spectrum (ATR, red, top) of [(TPB)AlBr] **5** with the computed IR spectra of **5** (black, bottom). Structures optimized at r^{2}SCAN-3c level of DFT with CPCM(fluorobenzene).

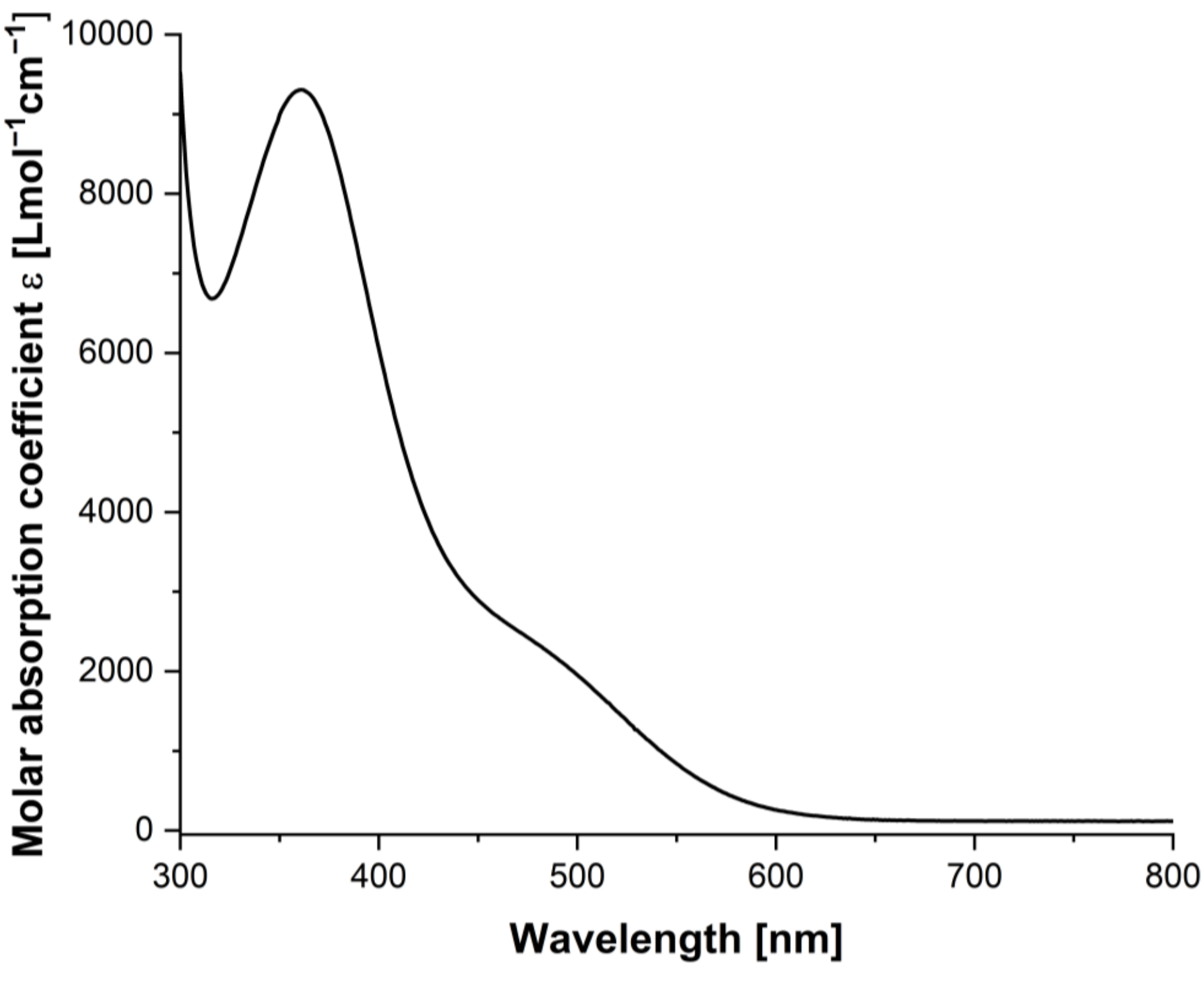

S-Figure 17: UV-Vis spectrum (toluene, 500 µM, RT) of [(TPB)AlBr] **5**.

Peak Analysis

Data Set:[A500mM1]500mM_1!E   Date:01/05/2026

BaseLine:Constant

Chi^2=8.18064E+02   Adj. R-Square=9.99902E-01   # of Data Points=470

SS=3.78764E+05   Degrees of Freedom=463

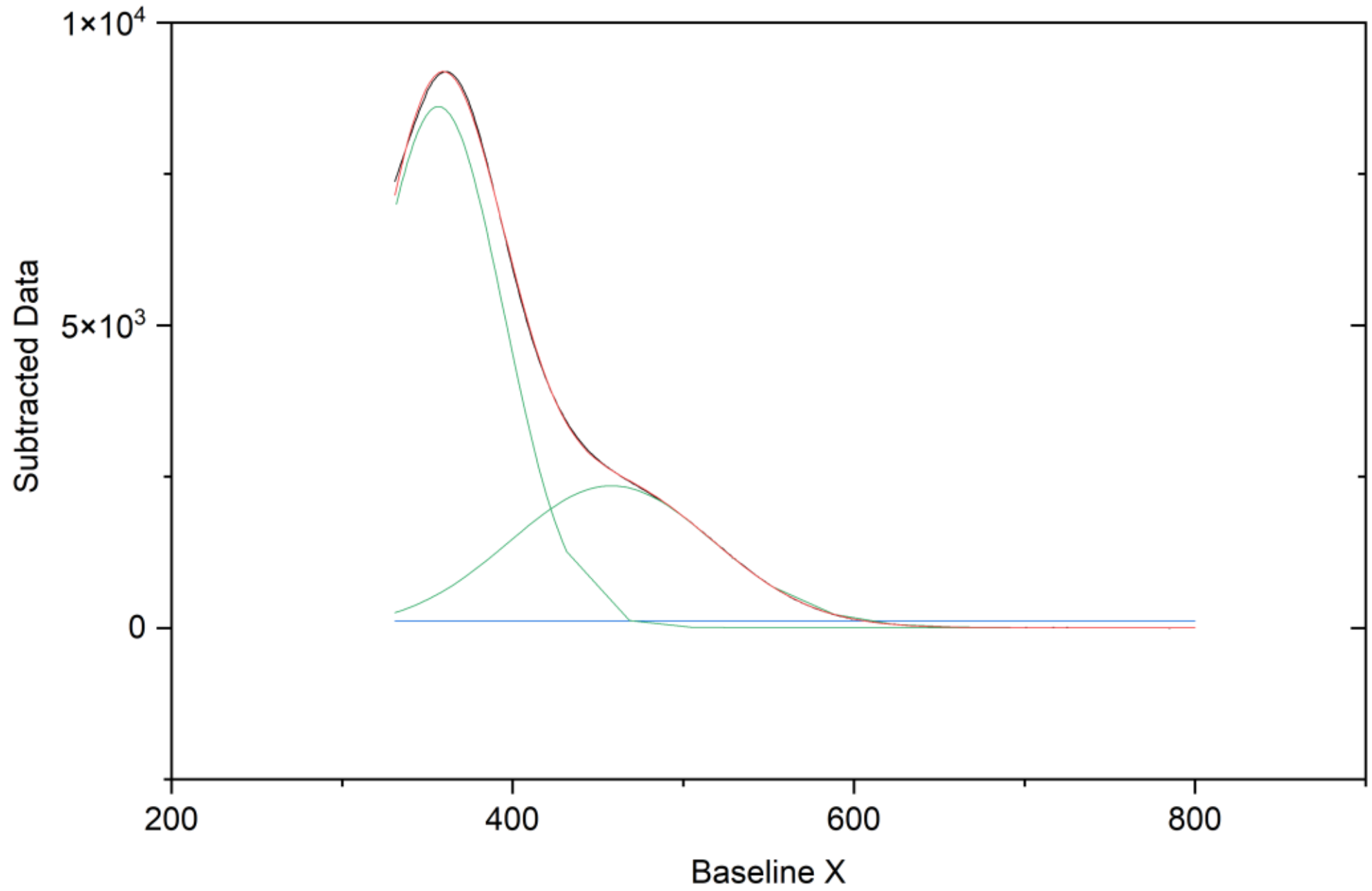

Fitting Results

Peak Index	Peak Type	Area Intg	FWHM	Max Height	Center Grvty	Area IntgP
1	GaussAmp	616822.38563	90.08346	8608.3012	356.48179	64.08905
2	GaussAmp	345623.4415	141.02231	2342.32661	457.92613	35.91095

S-Figure 18: Fit of UV-Vis spectrum (toluene, 500 µM, RT) of [(TPB)AlBr] **5**.

S-1.5 Preparation of [(TPB)AlI] 6

Procedure: TPB (100 mg, 126 μmol) and AlI_3 (156 mg, 384 μmol, 3.05 equiv.) were charged into a vial, into which 1,2-DFB (2 mL) was added. The mixture was stirred for 15 min until dissolved. Cobaltocene (48.8 mg, 258 μmol, 2.05 equiv.) was added whereby the yellow solution immediately turned dark red. The mixture was stirred for 30 min, and the solvent was removed in vacuo. The red residue was suspended in benzene (10 mL), filtered off, and the filter cake was washed thoroughly with benzene. The combined filtrate and washing solutions were dried in vacuo. The residual solids were washed with acetonitrile (3 mL) and pentane (10 mL), then dried thoroughly in vacuo to yield compound **6** as a red powder (49.0 mg, 51.7 μmol, 41%).

^{1}H NMR spectrum (399.89 MHz, C_6D_6, 298 K): δ = 6.72-6.78 (m, 3H, −P(C_6H_4)B), 6.78-6.84 (m, 8H, −PPh_2), 6.88-6.91 (m, 3H, -P(C_6H_4)B)), 6.98-7.07 (m, 10H, −PPh_2), 7.09-7.13 (m, 3H, −P(C_6H_4)B), 7.30-7.34 (m, 12H, −PPh_2), 7.63-7.65 (m, 3H, −P(C_6H_4)B) ppm.

^{13}C NMR spectrum (100.63 MHz, C_6D_6, 298 K): δ = 123.9 (−P(C_6H_4)B), 128.3 (−P(C_6H_4)B), 128.8 (−PPh_2), 132.8 ((−P(C_6H_4)B), 134.2 (−PPh_2), 135.8 ((−P(C_6H_4)B) ppm.

^{11}B NMR spectrum (128.30 MHz, C_6D_6, 298 K): δ = -8.1 (q, $^{3}J_{B,P}$ = 41.0 Hz) ppm.

$^{31}P\{^{1}H\}$ NMR spectrum (161.99, C_6D_6, 298 K): δ = -23.5 (q, $^{3}J_{B,P}$ = 43.0 Hz) ppm.

IR spectrum (128 scans, ATR, Diamond): ν = 689 (m), 726 (vs), 742 (m), 798 (m), 829 (vw), 969 (vs), 997 (vw), 1025 (vw), 1047 (w), 1095 (m), 1163 (m), 1213 (vs), 1238 (s), 1273 (m), 1296 (w), 1350 (vw), 1438 (vw), 1482 (vw), 1506 (vw), 1587 (vw) cm^{-1}.

UV-Vis (toluene, 500 μM, RT): λ_{max} = 360 (ε = 9648 $Lmol^{-1}cm^{-1}$), 459 (ε = 2771 $Lmol^{-1}cm^{-1}$) nm.

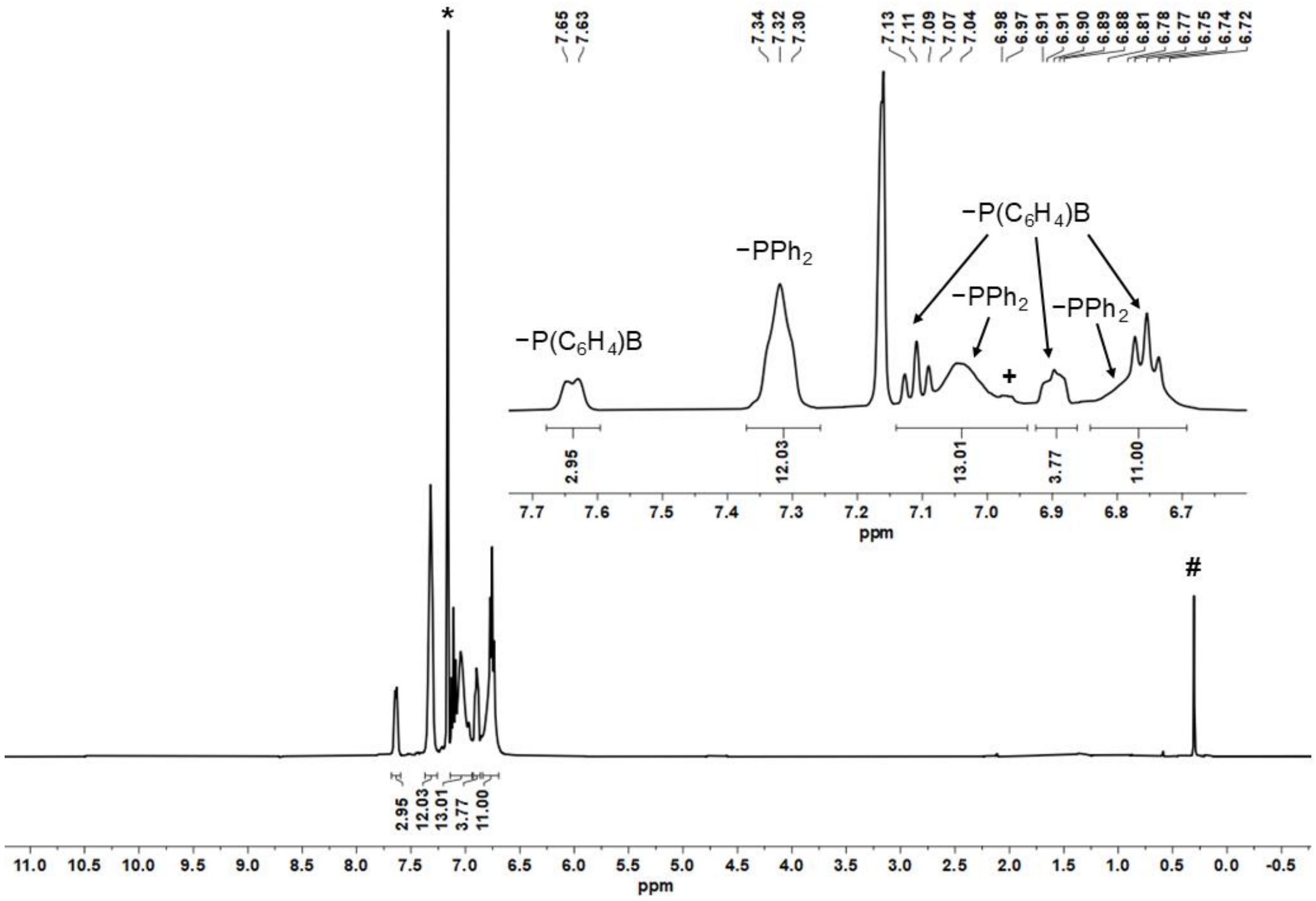

S-Figure 19: ^{1}H NMR spectrum (399.89 MHz, C_6D_6, 298 K) of [(TPB)AlI] **6**. C_6D_6 is marked with *, silicon grease with #, and residual starting material with +.

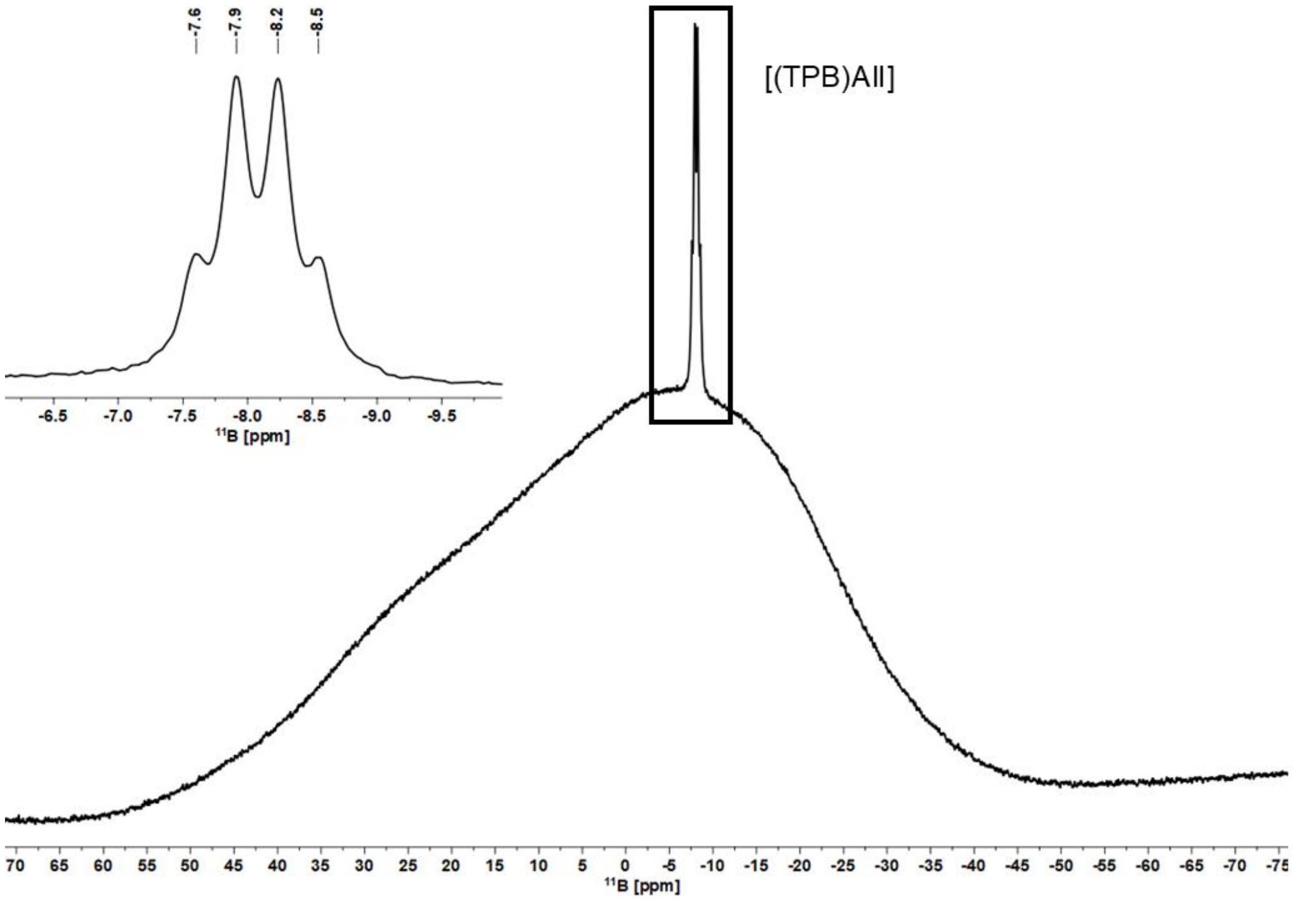

S-Figure 20: ^{11}B NMR spectrum (128.30 MHz, C_6D_6, 298 K) of [(TPB)AlI] **6**.

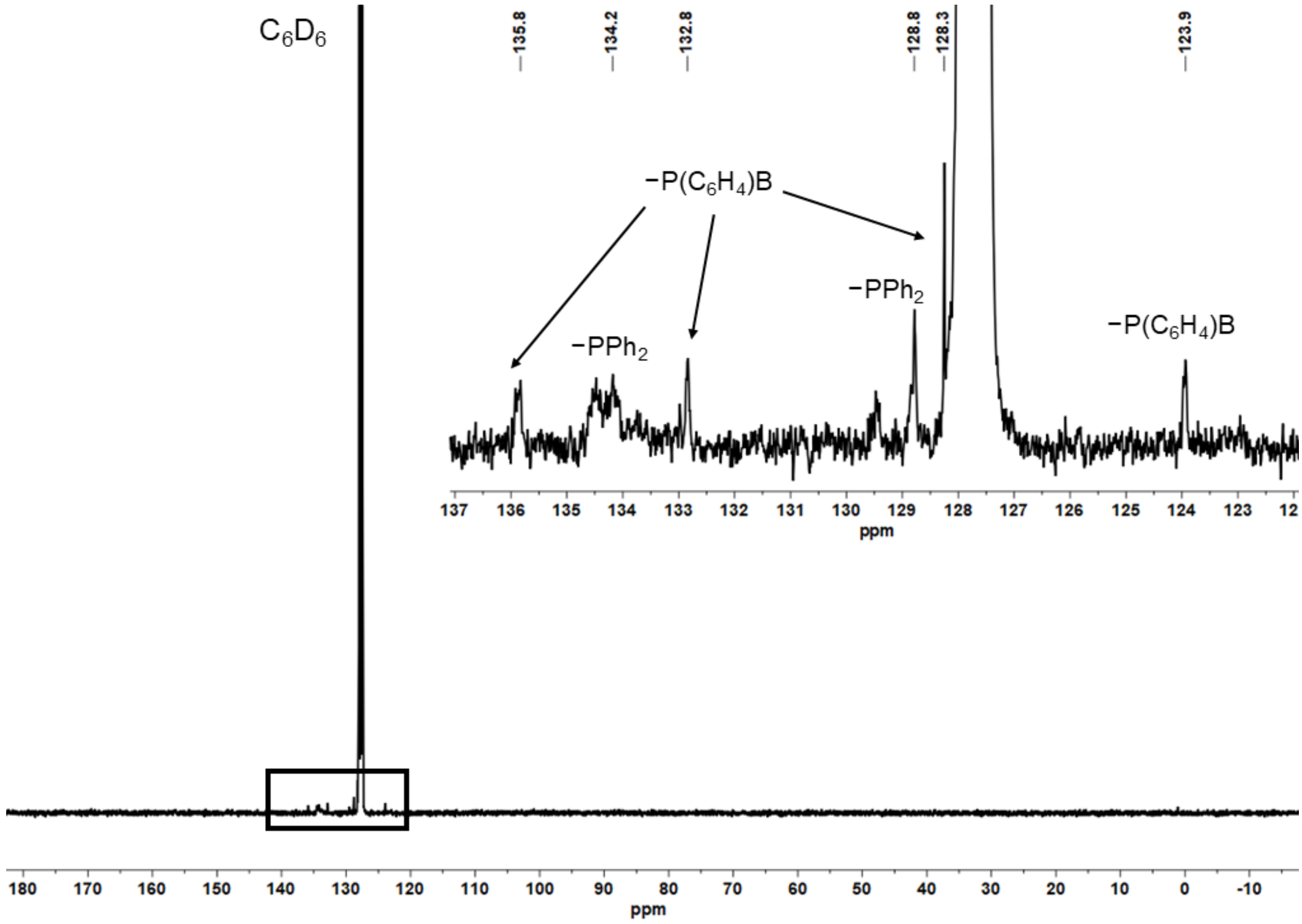

S-Figure 21: ^{13}C NMR spectrum (150.93 MHz, C_6D_6, 298 K) of [(TPB)AlI] **6**.

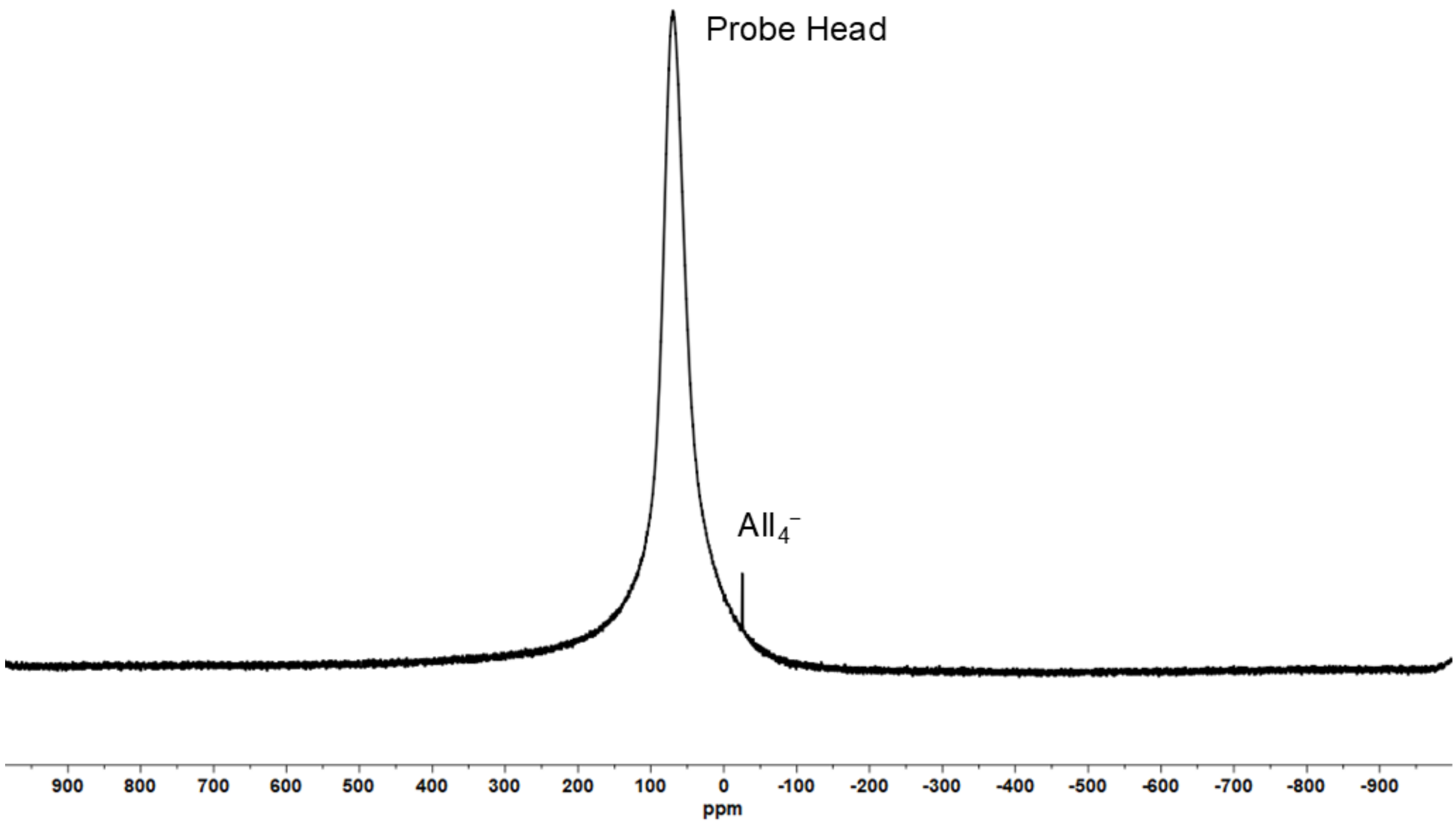

S-Figure 22: ^{27}Al NMR spectrum (104.20 MHz, C_6D_6, 298 K) of [(TPB)AlI] **6**.

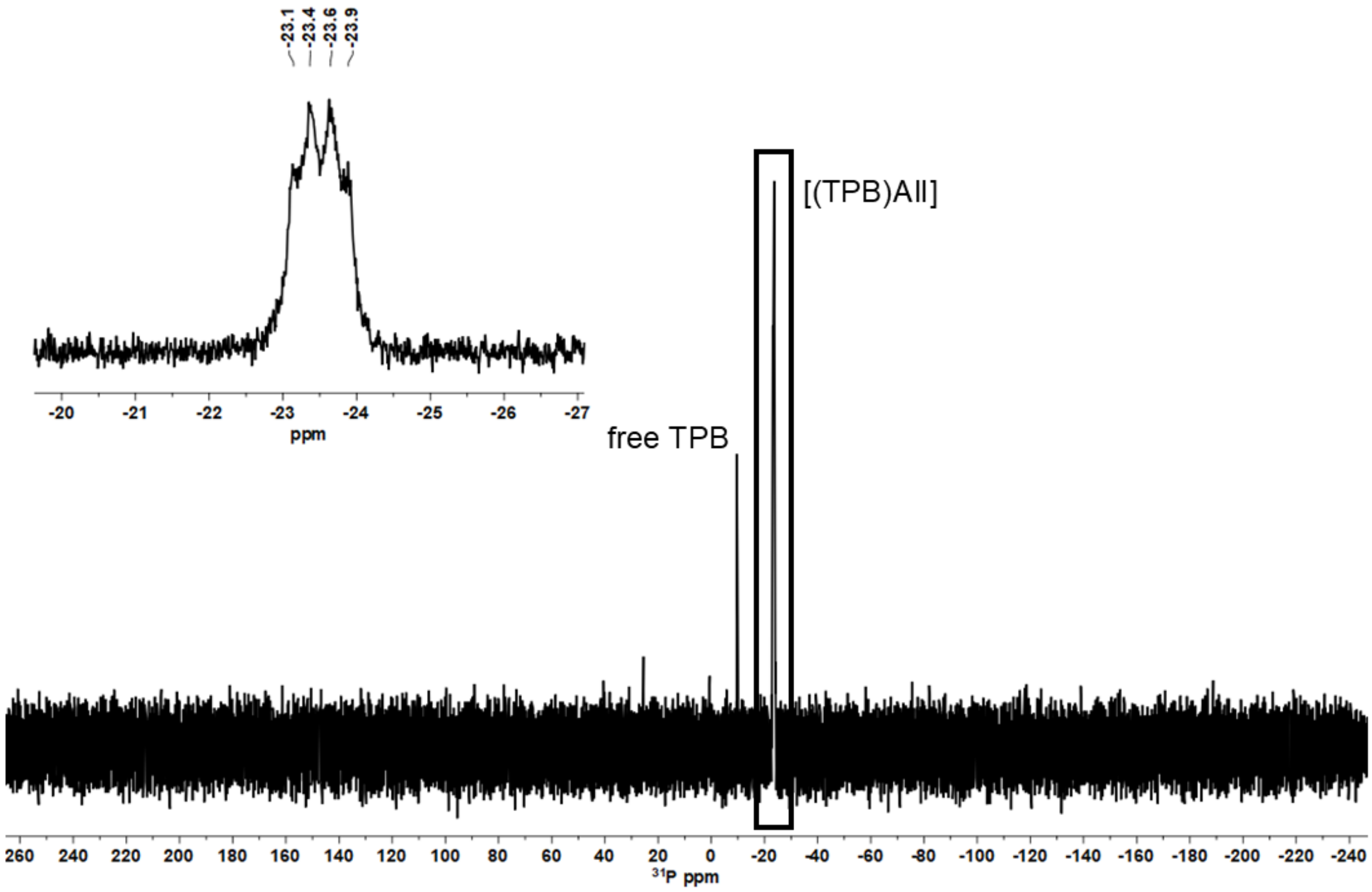

S-Figure 23: $^{31}P\{^{1}H\}$ NMR spectrum (161.89 MHz, C_6D_6, 298 K) of [(TPB)AlI] **6**.

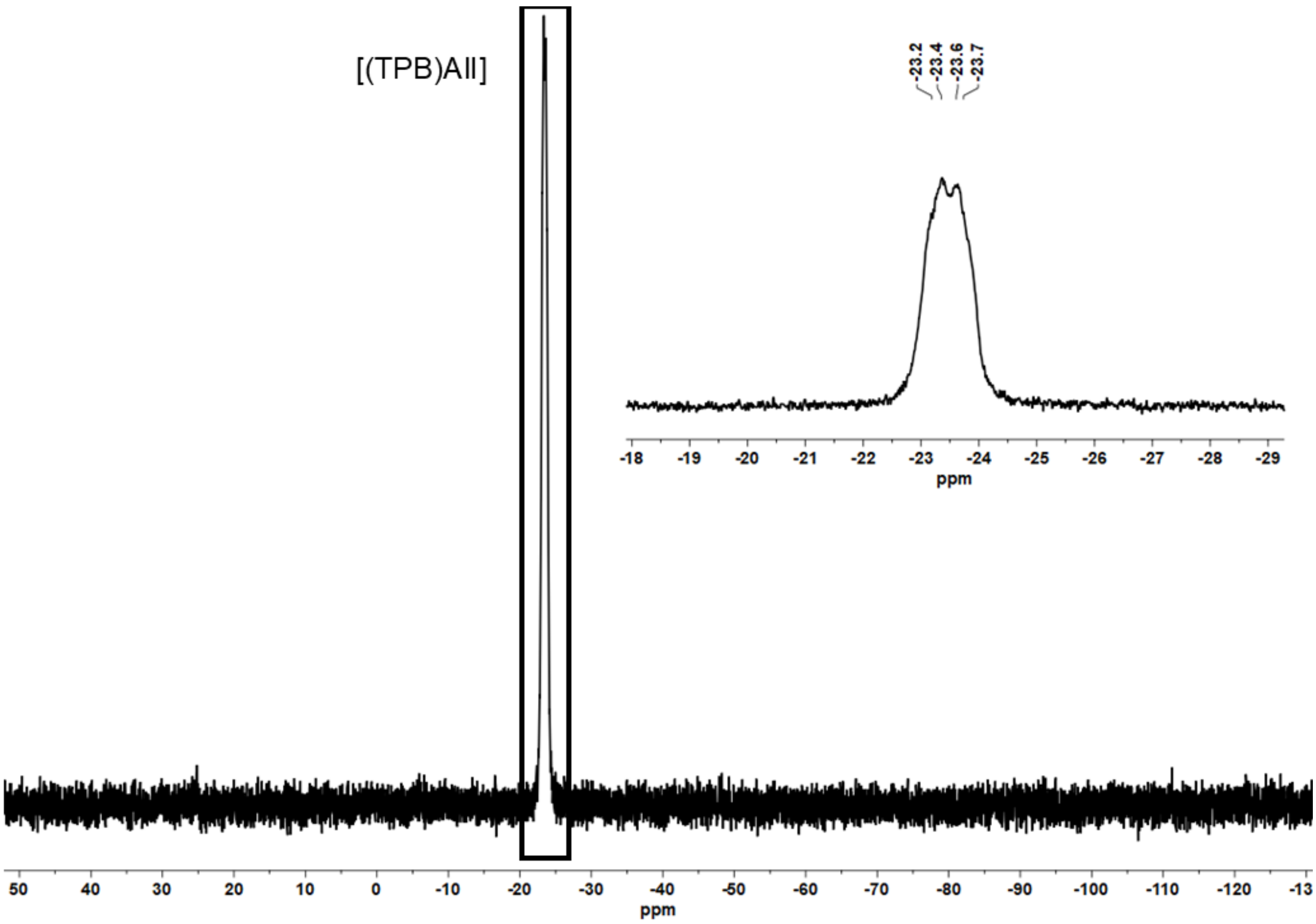

S-Figure 24: ^{31}P NMR spectrum (162.0 MHz, C_6H_6, 298 K) of [(TPB)AlI] **6**.

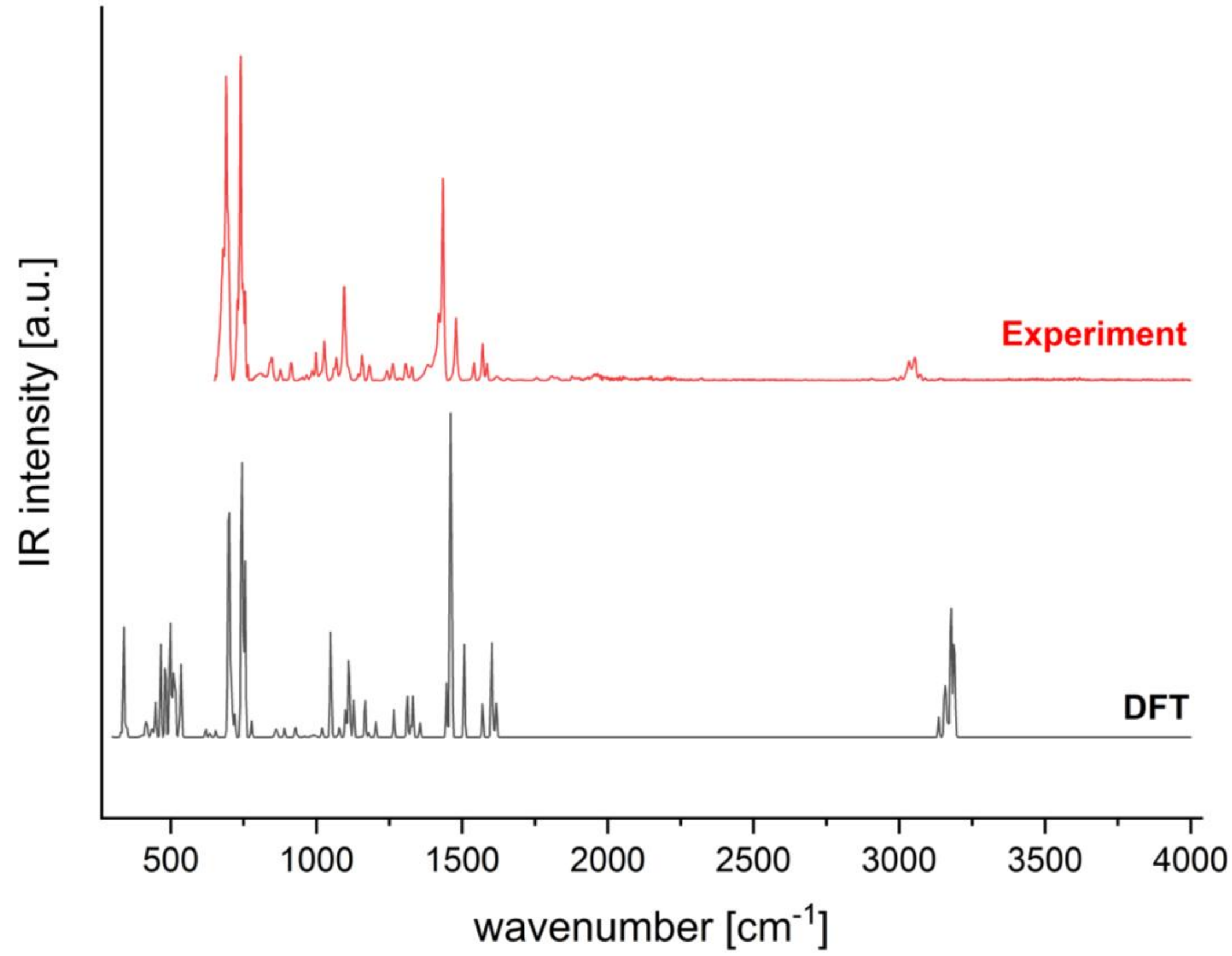

S-Figure 25: Comparison of the experimental IR spectrum (ATR, red, top) of [(TPB)AlI] **6** with the computed IR spectra of **6** (black, bottom). Structures optimized at r^{2}SCAN-3c level of DFT with CPCM(fluorobenzene).

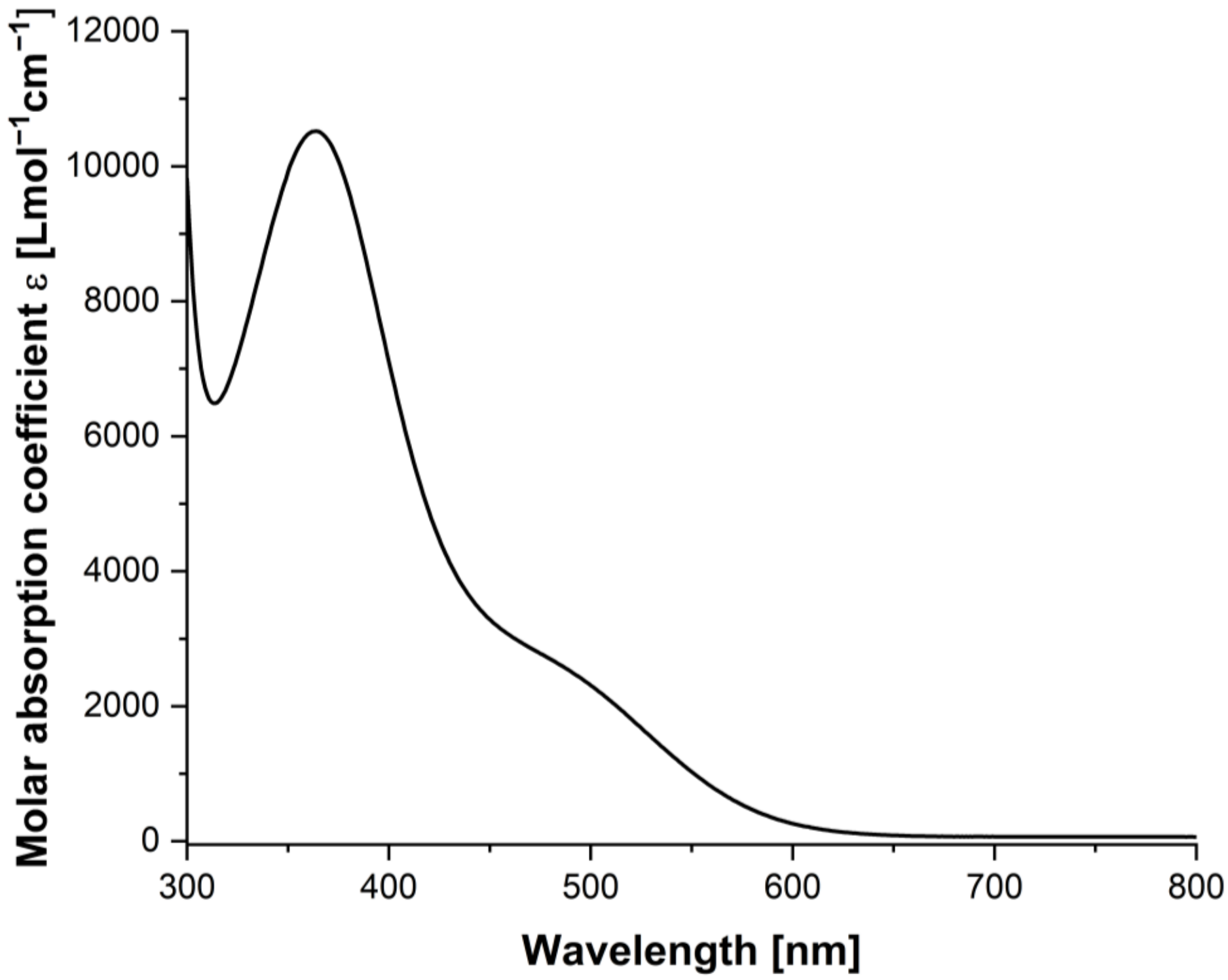

S-Figure 26: UV-Vis spectrum (toluene, 500 µM, RT) of [(TPB)AlI] **6**.

Peak Analysis

Data Set:[A500mM1]500mM_1!G | Date:01/05/2026

BaseLine:Constant

Chi^2=3.38854E+02 | Adj. R-Square=9.99967E-01 | # of Data Points=461

SS=1.53840E+05 | Degrees of Freedom=454

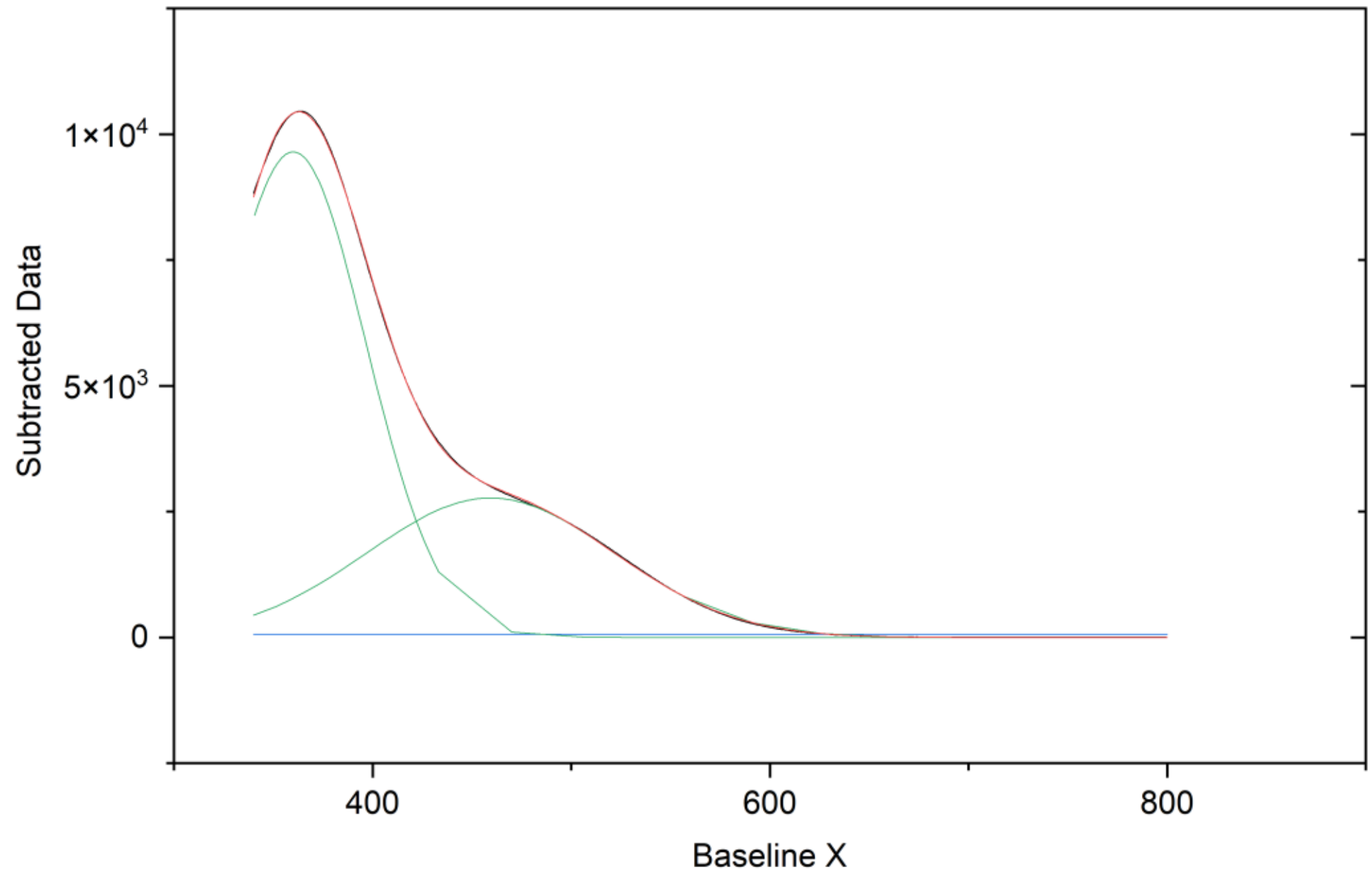

Fitting Results

Peak Index	Peak Type	Area Intg	FWHM	Max Height	Center Grvty	Area IntgP
1	GaussAmp	627052.26982	86.29032	9648.11049	360.02535	59.85197
2	GaussAmp	420619.69129	146.67596	2770.67174	459.36918	40.14803

S-Figure 27: Fit of UV-Vis spectrum (toluene, 500 µM, RT) of [(TPB)AlI] **6**.

S-1.7 NMR reaction: Preparation of [(TPB)AlCl] 4 via $[(TPB)AlCl]^{2+}([AlCl_4]^-)_2$ 1

Procedure: TPB (10.0 mg, 12.6 μmol) and $AlCl_3$ (5.00 mg, 37.8 μmol, 3.00 equiv.) were charged into a vial, into which 1,2-DFB (0.5 mL) was added. The mixture was stirred at room temperature. ^{31}P NMR spectra were measured after 15 min and 16 h of stirring. Cobaltocene (4.72 mg, 25.0 μmol, 2.00 equiv.) was added, whereby the yellow solution immediately turned dark red. The reaction mixture was stirred for 15 min at room temperature and then investigated by NMR spectroscopy. Crystals of **4** were obtained by layering a solution of compound **4** in C_6D_6 with pentane at room temperature.

Analysis: The ^{31}P NMR spectrum of the reaction mixture after 15 min of stirring showed signals assigned to the free TPB (**S-Figure 28**). After stirring overnight, these signals vanished, and a new broad signal appeared at −7.4 ppm, which was assigned the proposed dication $[(TPB)AlCl]^{2+}$ **1** (**S-Figure 29**). After the addition of cobaltocene, ^{11}B and ^{31}P NMR spectra show signals which can be assigned to the product [(TPB)AlCl] **4** (**S-Figure 30**, **S-Figure 31**). Detailed analysis of compound **4** is shown in chapter S-1.3.

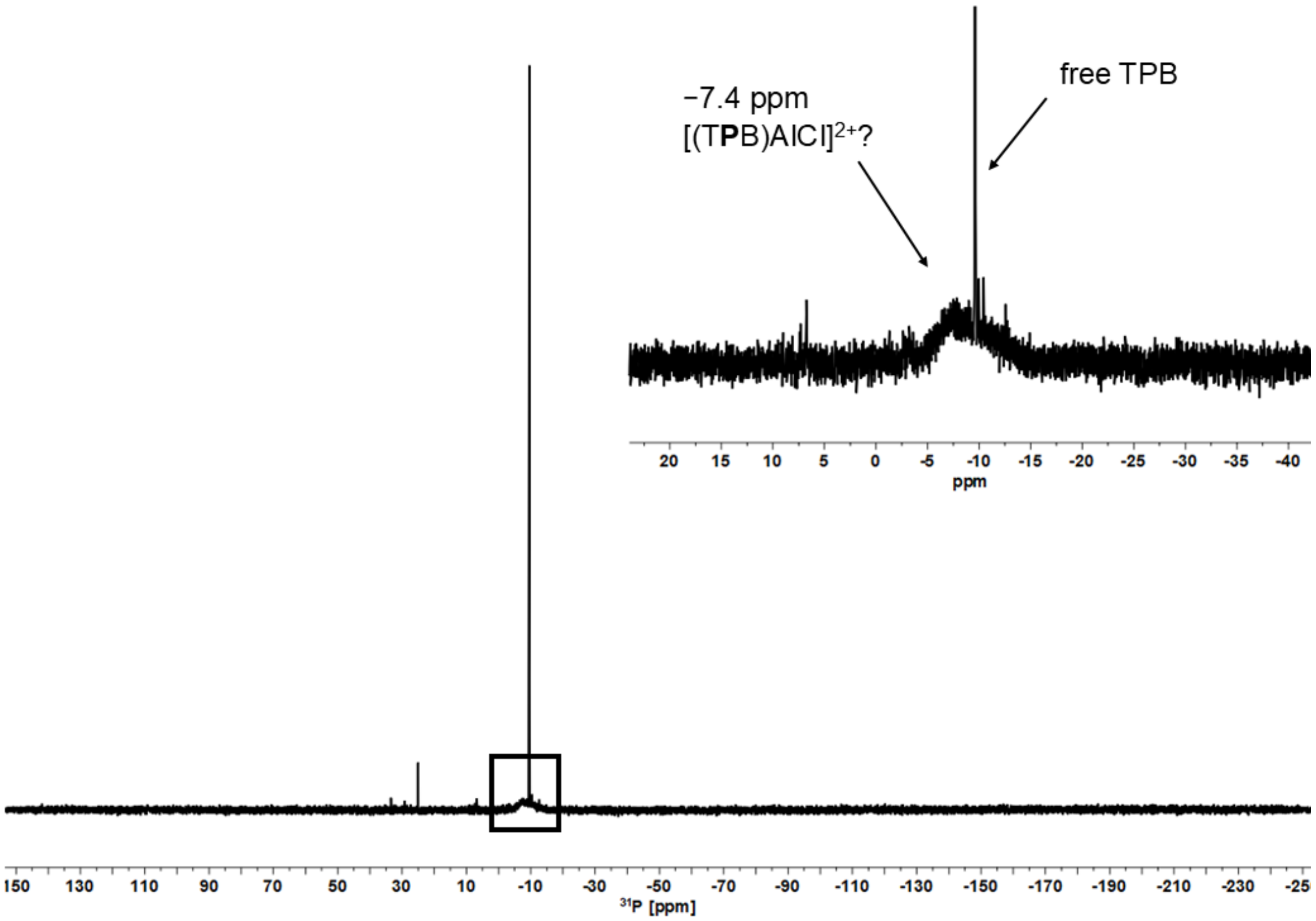

S-Figure 28: ^{31}P{^{1}H} NMR spectrum (162.00 MHz, 1,2-DFB, 298 K) of the mixture of TPB and 3 equiv. $AlCl_3$ after 15 min.

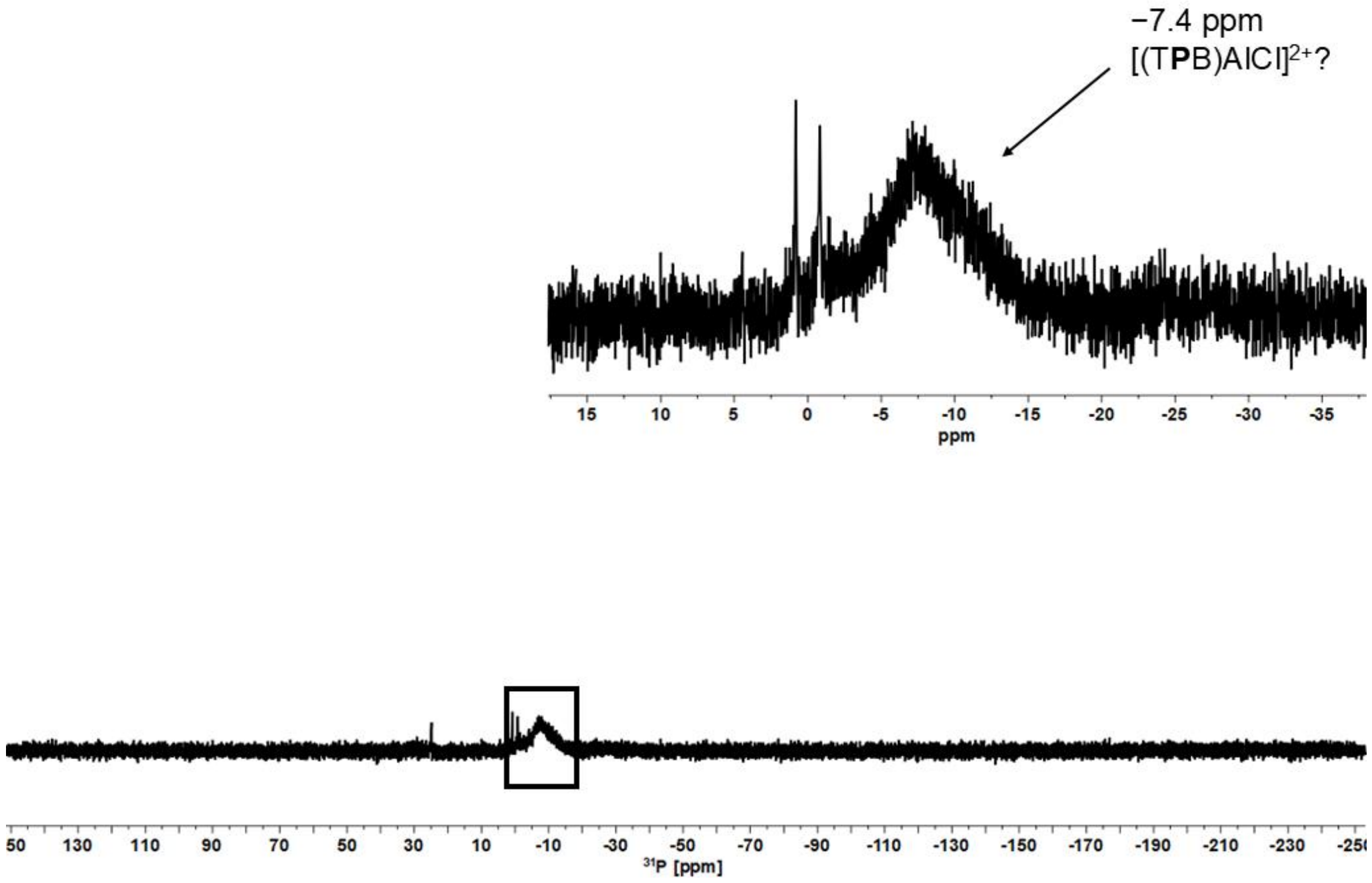

S-Figure 29: $^{31}P\{^{1}H\}$ NMR spectrum (162.00 MHz, 1,2-DFB, 298 K) of the mixture of TPB and 3 equiv. $AlCl_3$ after 15 min.

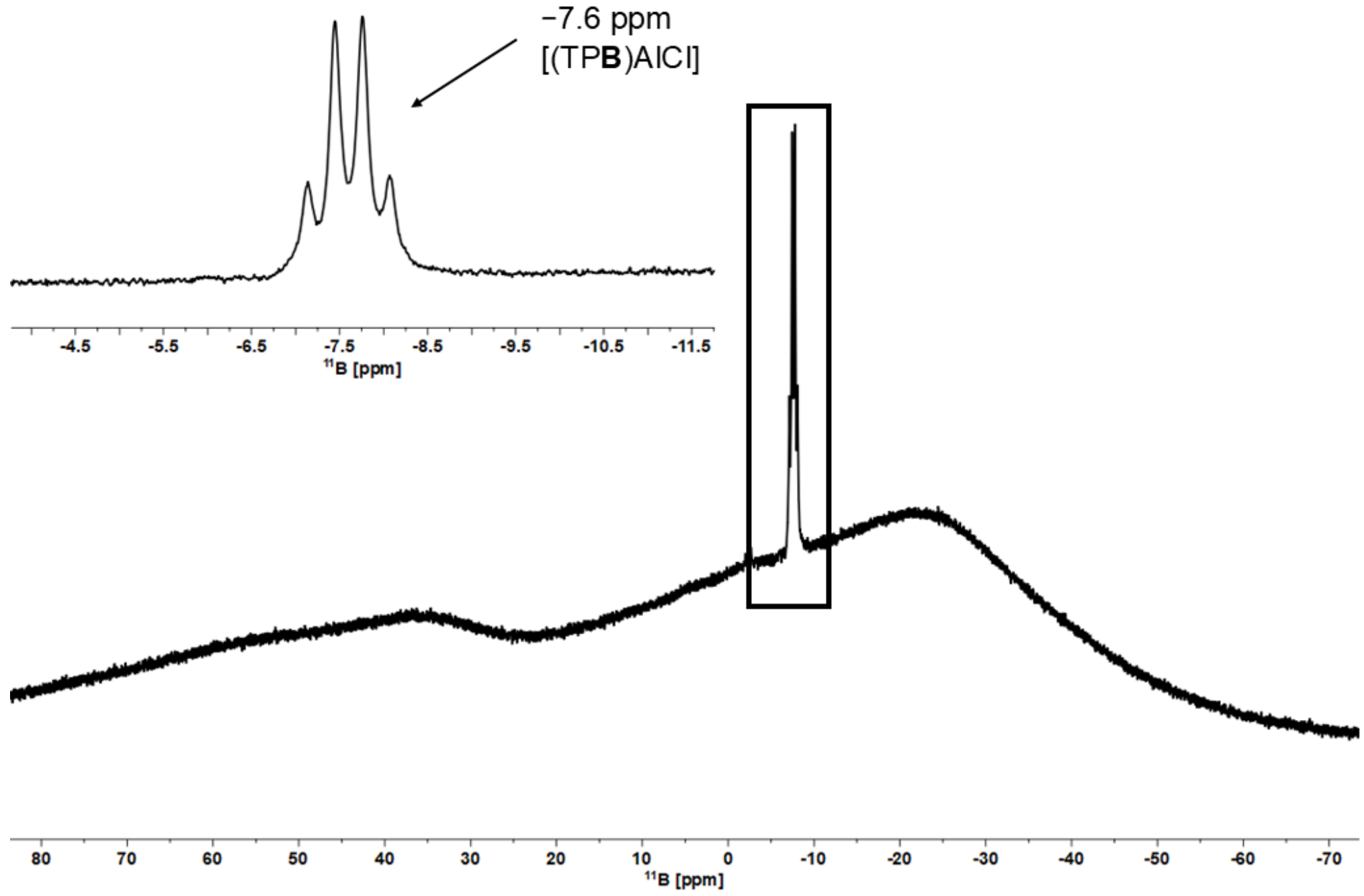

S-Figure 30: ^{11}B NMR spectrum (128.40 MHz, 1,2-DFB, 298 K) of in situ formed [(TPB)AlCl] **4** after addition of cobaltocene**.**

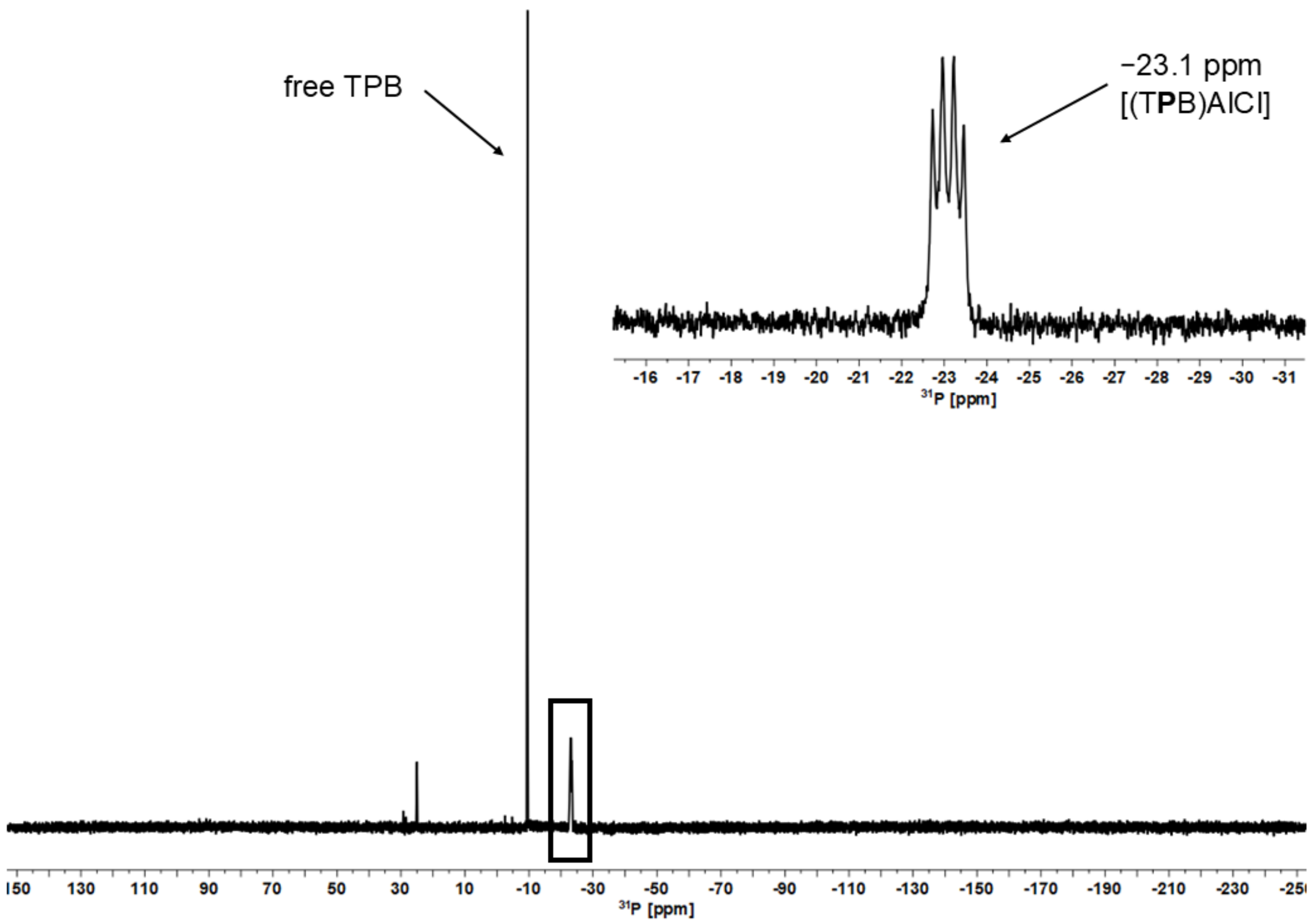

S-Figure 31: $^{31}P\{^{1}H\}$ NMR spectrum (162.00 MHz, 1,2-DFB, 298 K) of in situ formed [(TPB)AlCl] **4** after addition of cobaltocene**.**

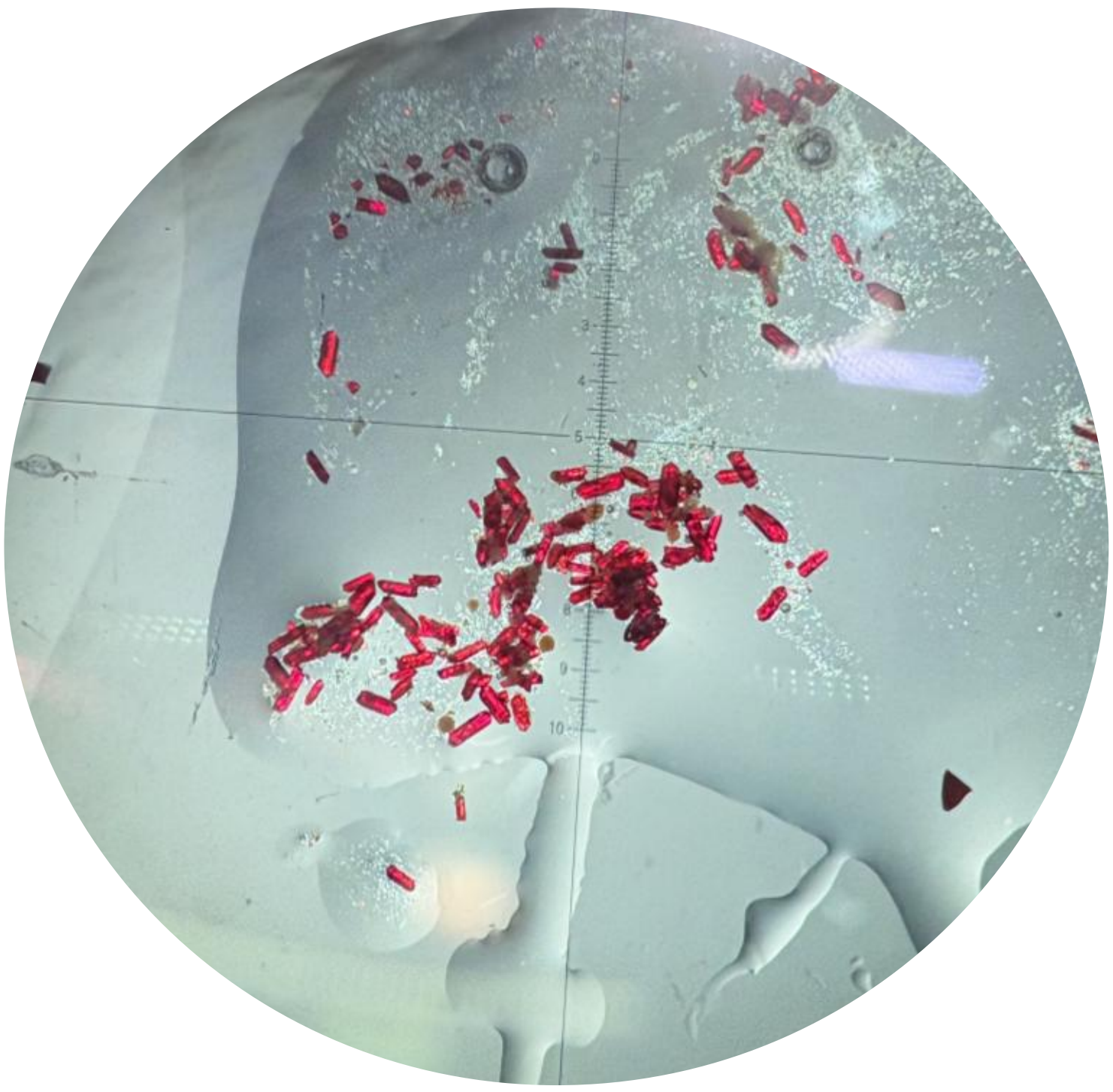

S-Figure 32: Picture of crystals of [(TPB)AlCl] **4** obtained from crystallization of the reaction mixture.

S-1.8 NMR reaction: Preparation of [(TPB)AlBr] 5 from $[(TPB)AlBr]^{2+}([AlBr_4]^-)_2$ 2

Procedure: TPB (10.0 mg, 12.6 μmol) and $AlBr_3$ (10.0 mg, 37.8 μmol, 3.00 equiv.) were charged into a vial, into which 1,2-DFB (0.5 mL) was added. The mixture was stirred at room temperature for 15 min. The obtained solution was analyzed by ^{31}P NMR spectroscopy. Cobaltocene (4.72 mg, 25.0 μmol, 2.00 equiv.) was added, whereby the yellow solution immediately turned dark red. The reaction mixture was stirred for 30 min at room temperature and then investigated by NMR spectroscopy. Crystals were obtained by layering a solution of compound **5** in C_6D_6 with pentane.

Analysis: The ^{31}P NMR spectrum shows a new broad signal at −4.0 ppm, which was assigned the proposed dication $[(TPB)AlBr]^{2+}$ **2** (**S-Figure 33**). After the addition of cobaltocene, ^{11}B and ^{31}P NMR spectra show signals which can be assigned to the product [(TPB)AlBr] **5** (**S-Figure 34**, **S-Figure 35**). Detailed analysis of compound **5** is shown in chapter S-1.4.

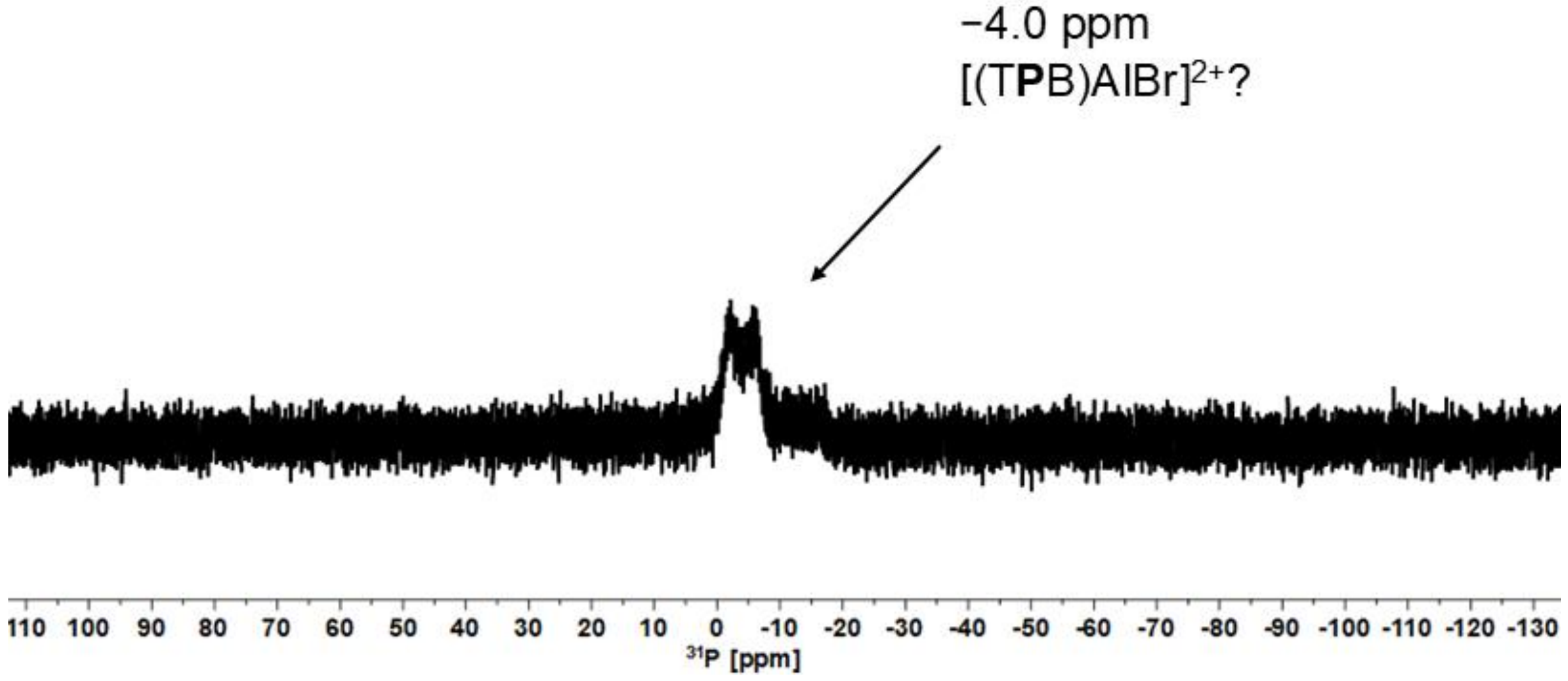

S-Figure 33: ^{31}P NMR spectrum (162.00 MHz, 1,2-DFB, 298 K) of the mixture of TPB and 3 equiv. $AlBr_3$ after 15 min.

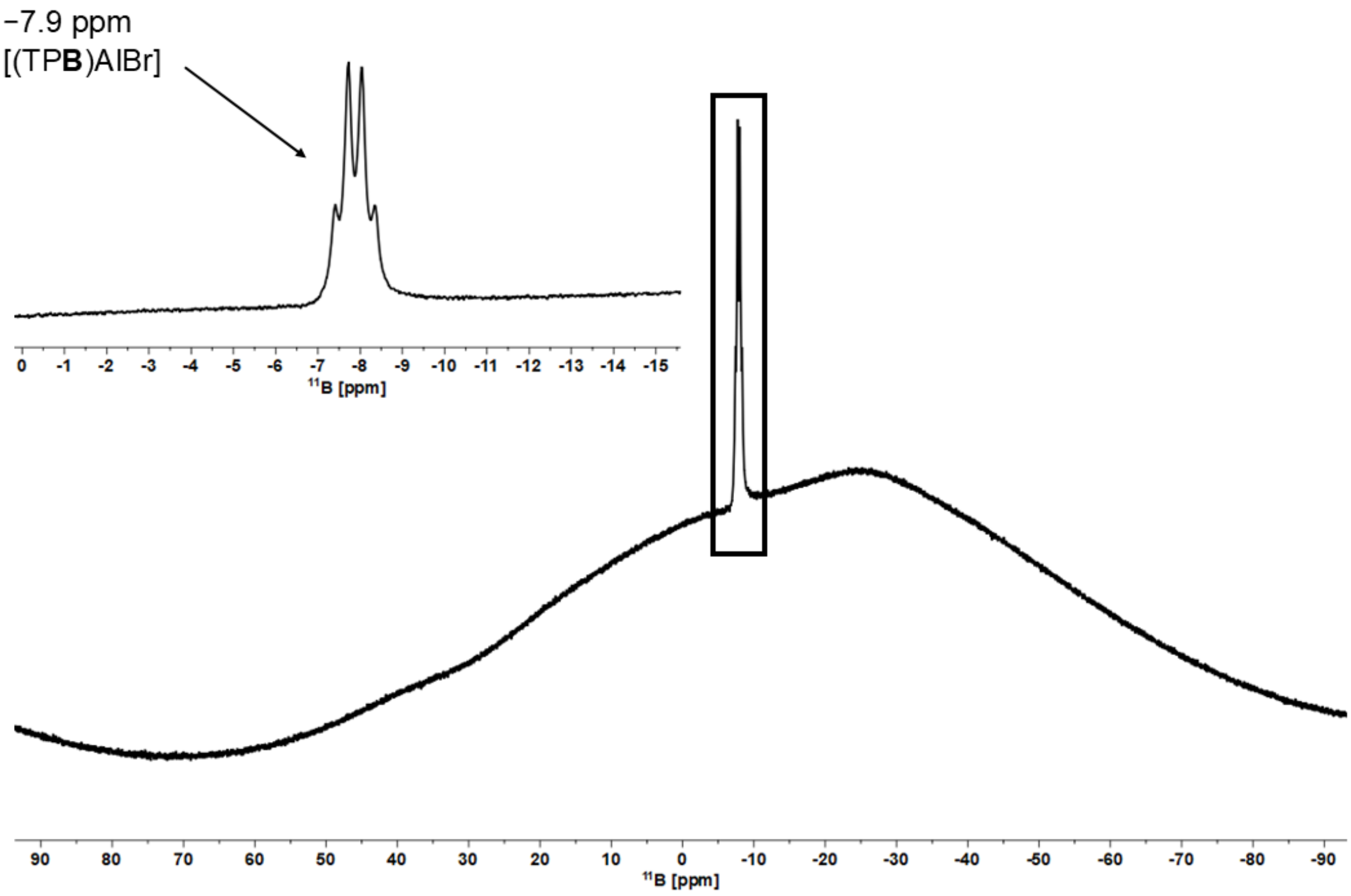

S-Figure 34: ^{11}B NMR spectrum (128.40 MHz, 1,2-DFB, 298 K) of [(TPB)AlBr] **5** after addition of cobaltocene.

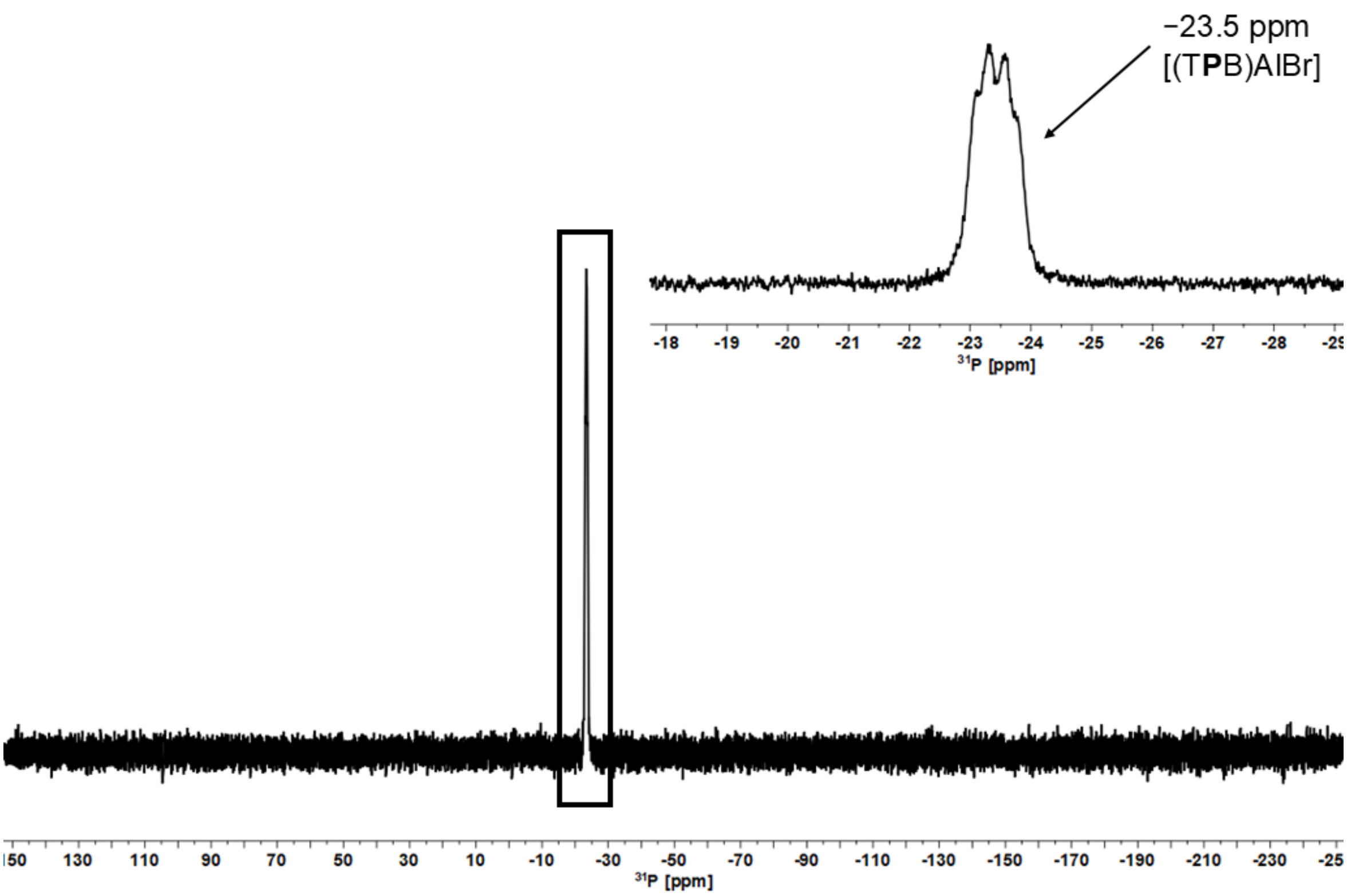

S-Figure 35: ^{31}P NMR spectrum (162.00 MHz, 1,2-DFB, 298 K) of [(TPB)AlBr] **5** after addition of cobaltocene.

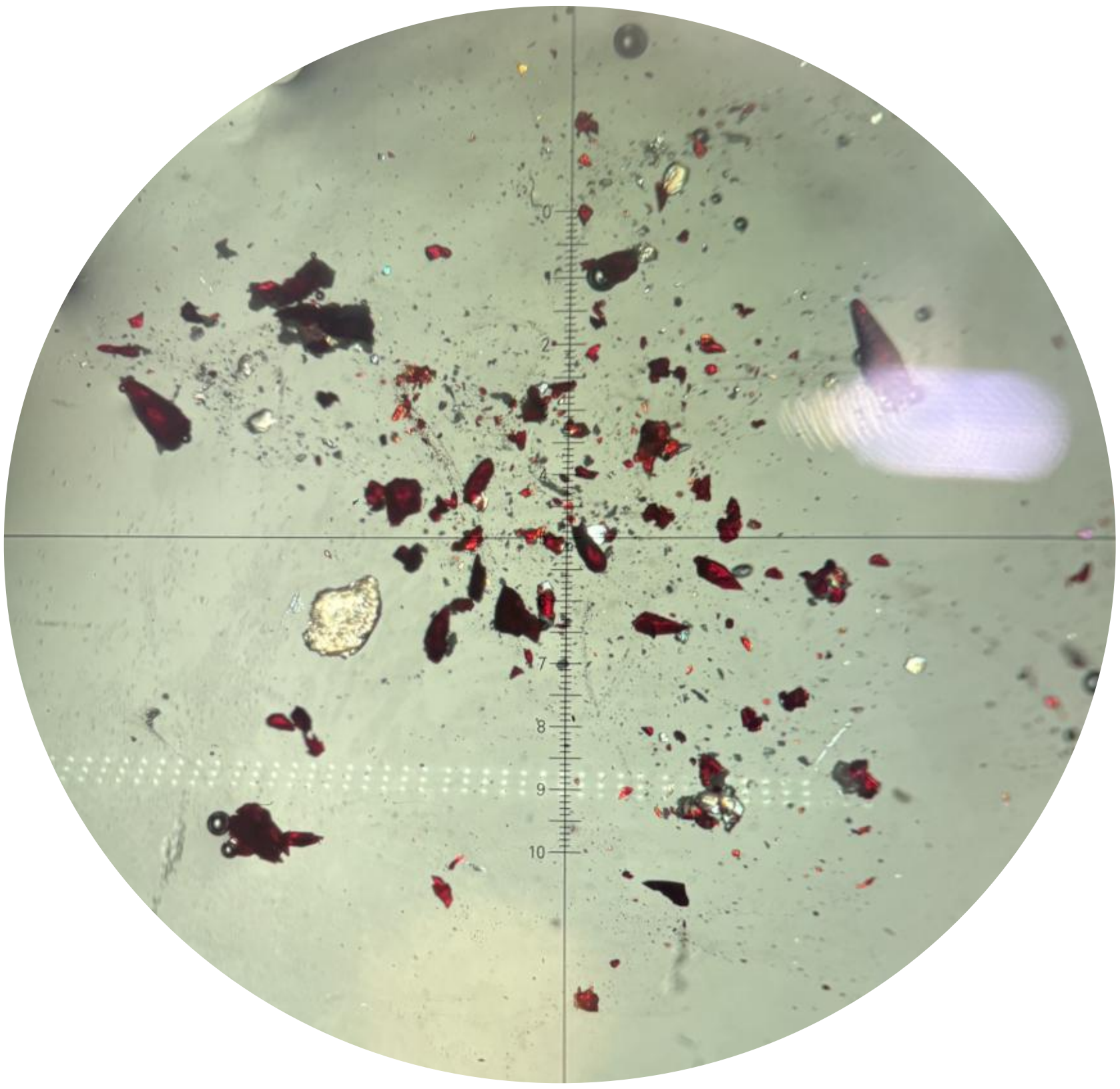

S-Figure 36: Picture of crystals of [(TPB)AlBr] **5** obtained from crystallization of the reaction mixture.

S-1.9 NMR reaction: Preparation of [(TPB)AlI] 6 via [(TPB)AlI]$^{2+}$([AlI$_4$]$^-$)$_2$ 3

Procedure: TPB (10.0 mg, 12.6 μmol) and AlI_3 (15.4 mg, 37.8 μmol, 3.00 equiv.) were charged into a vial, into which 1,2-DFB (0.5 mL) was added. The mixture was stirred at room temperature for 15 min. The obtained solution was analyzed by ^{31}P NMR spectroscopy. Cobaltocene (4.72 mg, 25.0 μmol, 2.00 equiv.) was added, whereby the yellow solution immediately turned dark red. The reaction mixture was stirred for 30 min at room temperature and then investigated by NMR spectroscopy. Crystals were obtained by layering a solution of compound **6** in C_6D_6 with pentane.

Analysis: A ^{31}P NMR spectrum shows a new broad signal at −5.8 ppm, which was assigned the proposed dication [(TPB)AlI]$^{2+}$ **3** (**S-Figure 37**). After addition of cobaltocene, ^{11}B and ^{31}P NMR spectra show signals which can be assigned to the product [(TPB)AlI] **6** (**S-Figure 38**, **S-Figure 39**). Detailed analysis of compound **6** is shown in chapter S-1.5.

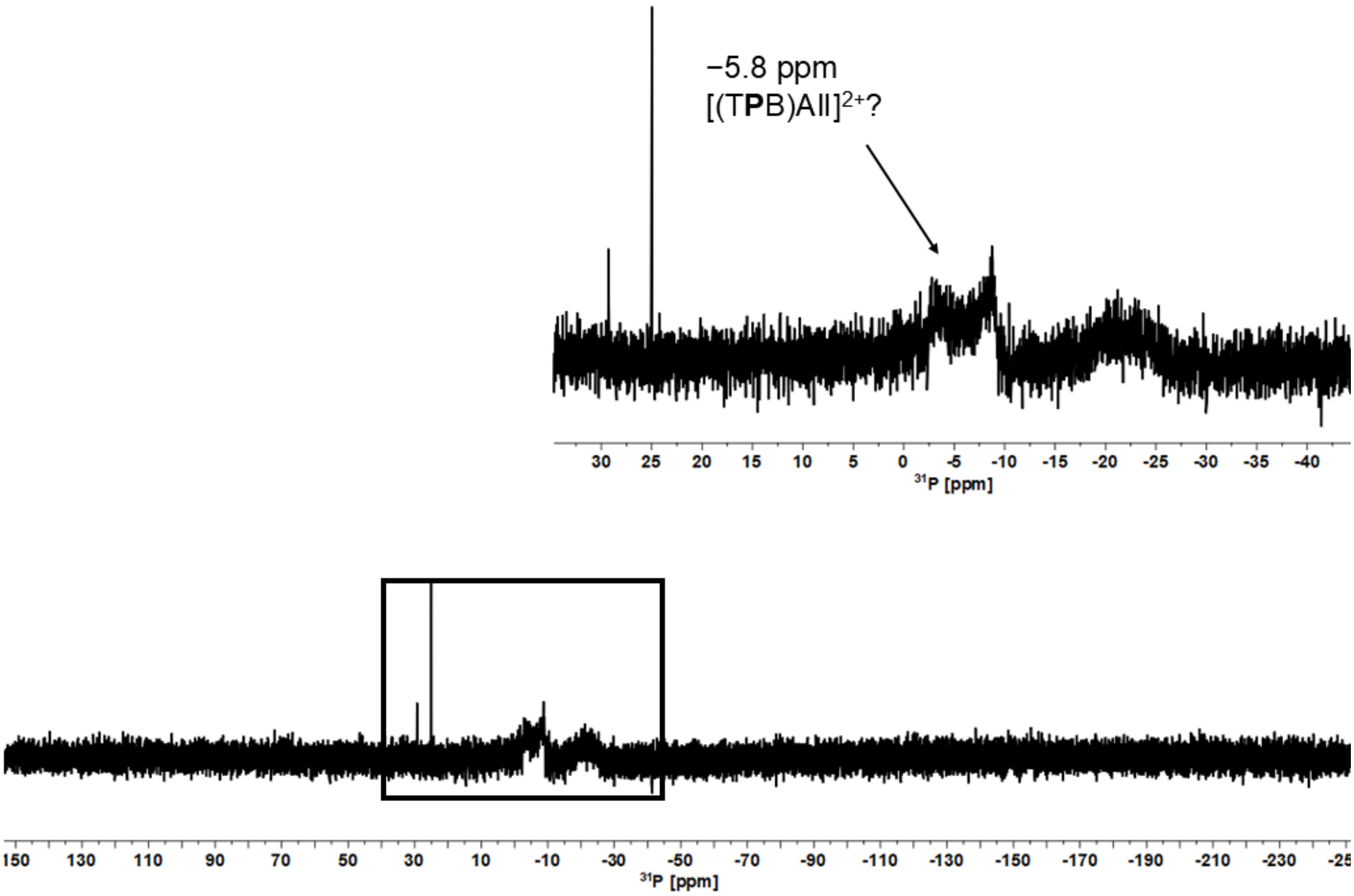

S-Figure 37: ^{31}P{^{1}H} NMR spectrum (162.00 MHz, 1,2-DFB, 298 K) of the mixture of TPB and 3 equiv. AlI_3 after 15 min.

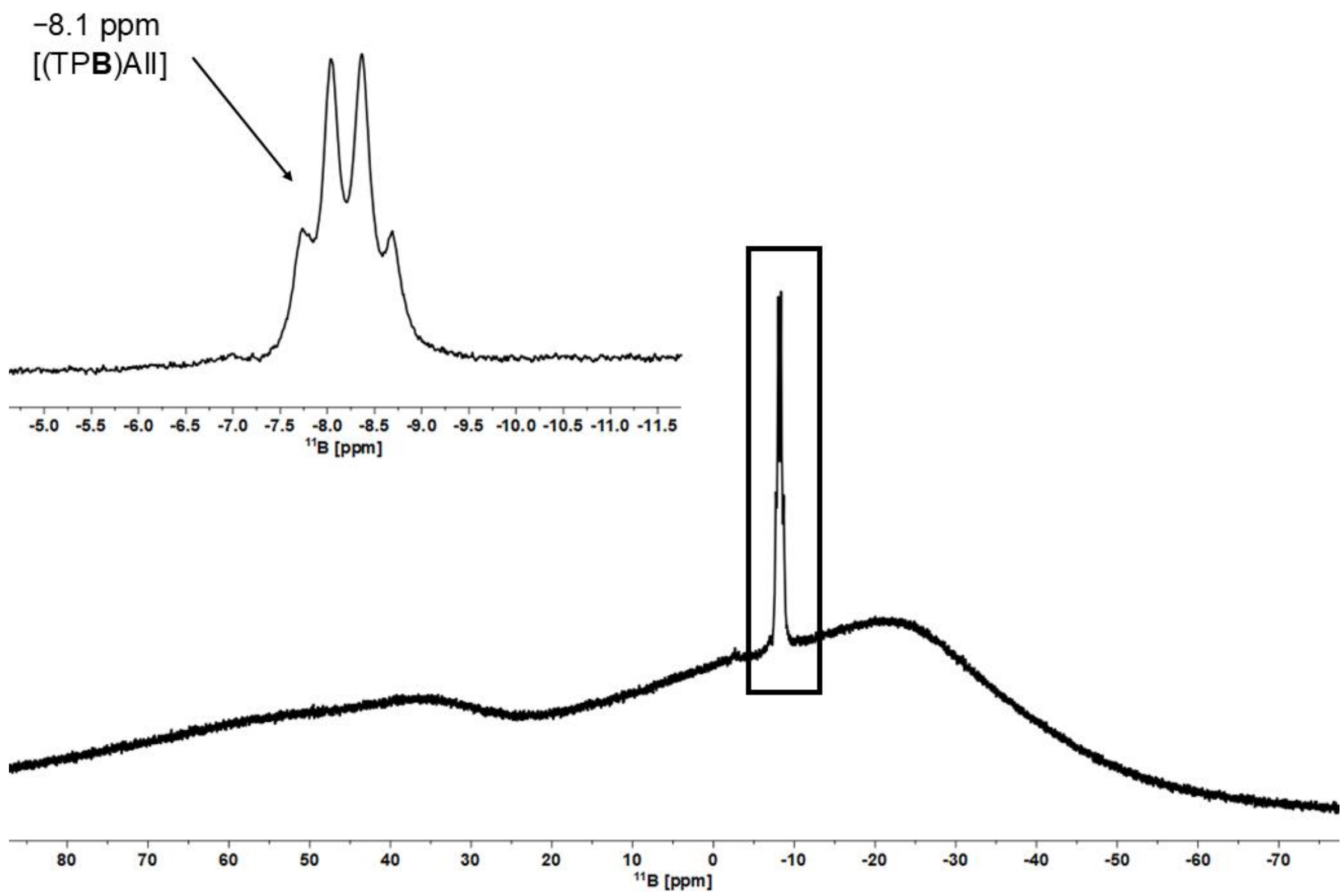

S-Figure 38: ^{11}B NMR spectrum (128.40 MHz, 1,2-DFB, 298 K) of [(TPB)AlI] **6** after addition of cobaltocene.

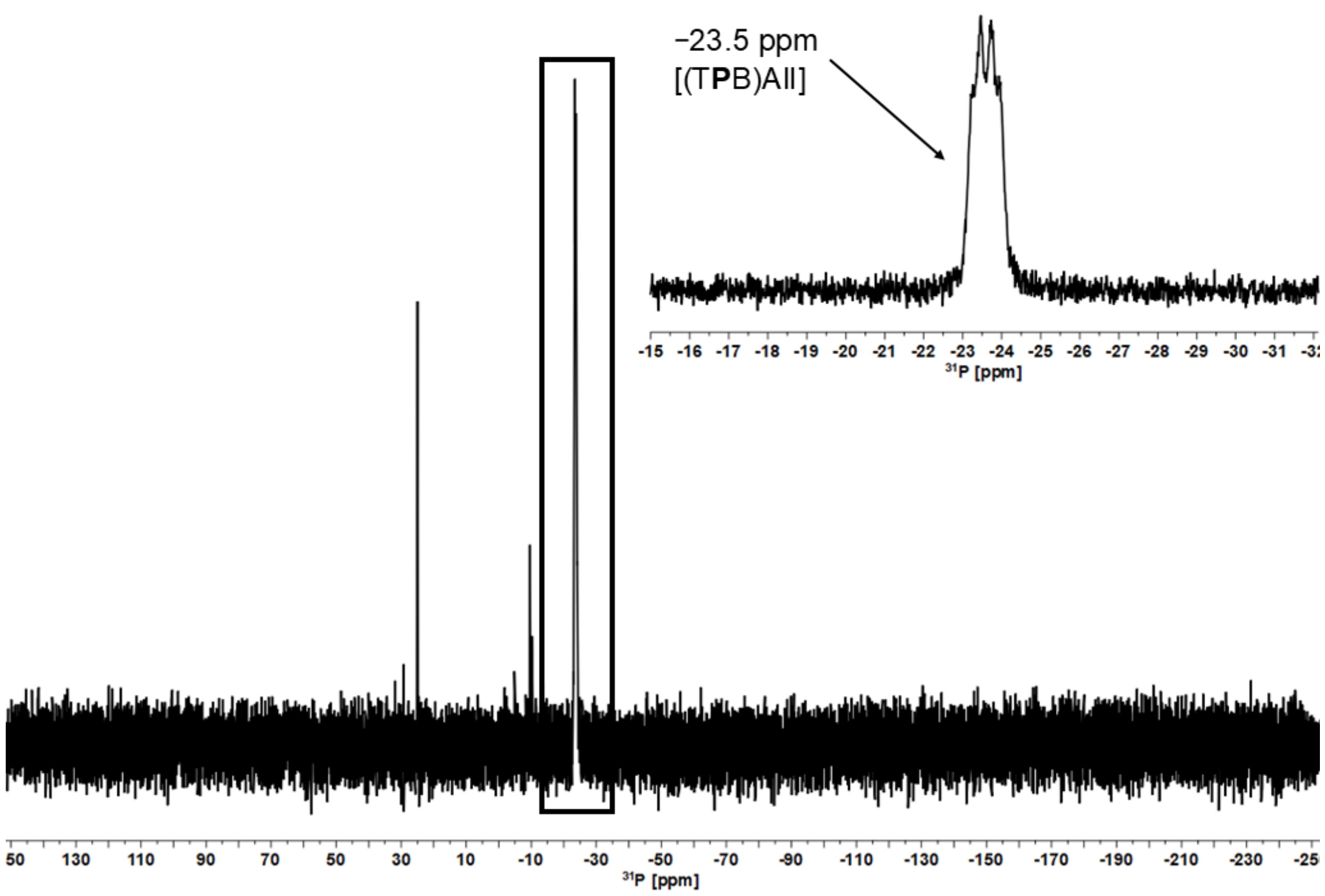

S-Figure 39: $^{31}P\{^1H\}$ NMR spectrum (162.00 MHz, 1,2-DFB, 298 K) of of [(TPB)AlI] **6** after addition of cobaltocene.

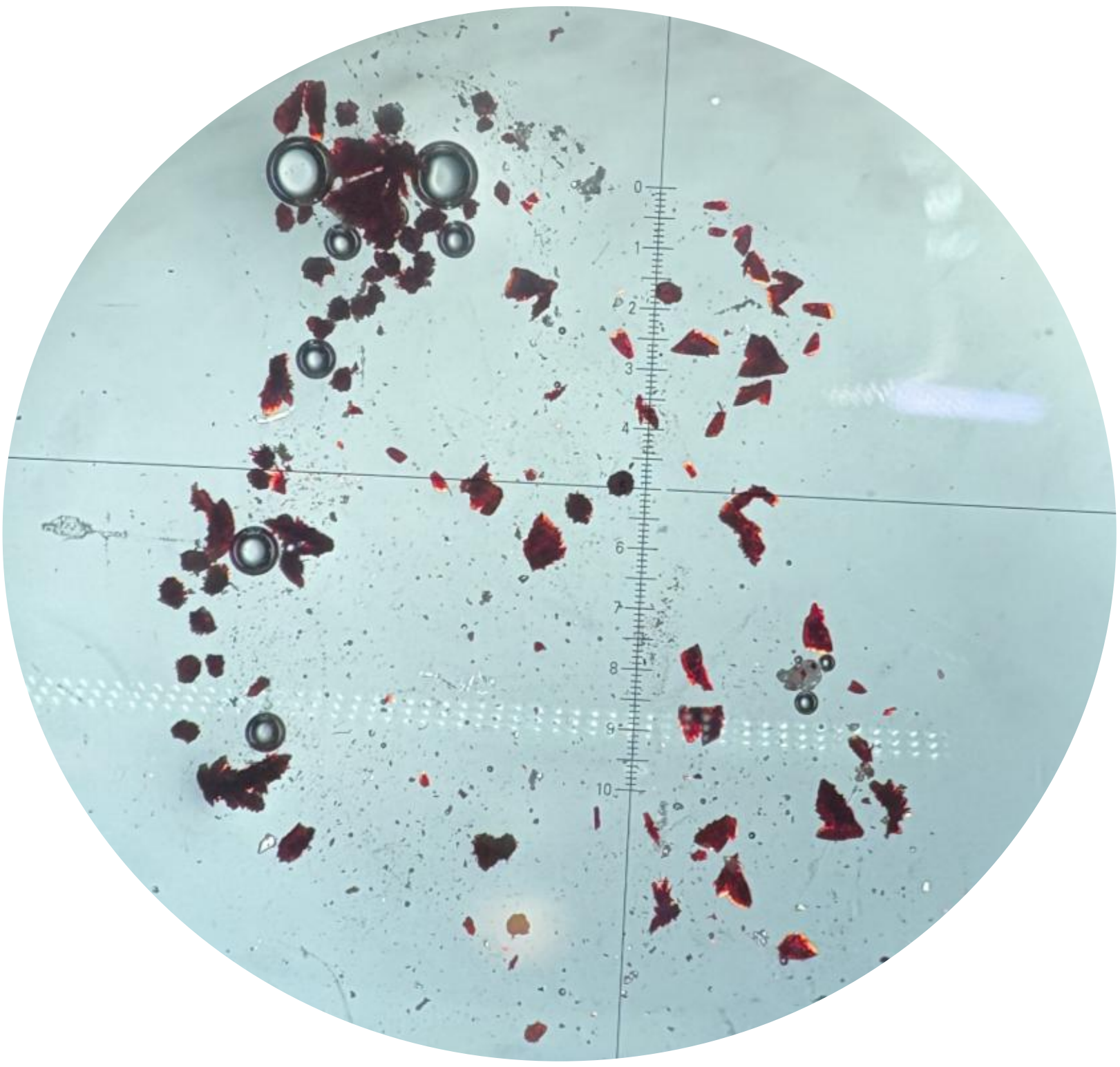

S-Figure 40: Picture of crystals of [(TPB)AlI] **6** obtained from crystallization of the reaction mixture.

S-1.10 Attempted isolation of $[(TPB)AlCl]^{2+}([AlCl_4]^{-})_2$ 1

Procedure: TPB (10.0 mg, 12.6 µmol) and $AlCl_3$ (15.4 mg, 37.8 µmol, 3.00 equiv.) were charged into a vial, into which 1,2-DFB (0.5 mL) was added. The mixture was stirred at room temperature for 16 h. The solvent was removed in vacuo, and the remaining solid was washed with pentane. The obtained yellow powder was analyzed by NMR and IR spectroscopy.

Analysis: ^{11}B, ^{27}Al, and ^{31}P NMR spectra of compound **1** were measured with a high scan number (**S-Figure 41**, **S-Figure 42**,

S-Figure 43). The ^{11}B and ^{31}P NMR spectra each reveal an additional signal at −8.2 ppm and -7.4 ppm which were assigned to the proposed dication $[(TPB)AlCl]^{2+}$. The ^{27}Al NMR spectrum shows a broad signal at approximately 104 ppm, which was assigned to $AlCl_4^{-}$. The peak broadening is most likely due to coordination of $AlCl_4^{-}$, resulting in hindered rotation. The presumed dicationic species is not stable in solution, as measurements with a high scan number show decomposition products in all NMR spectra.

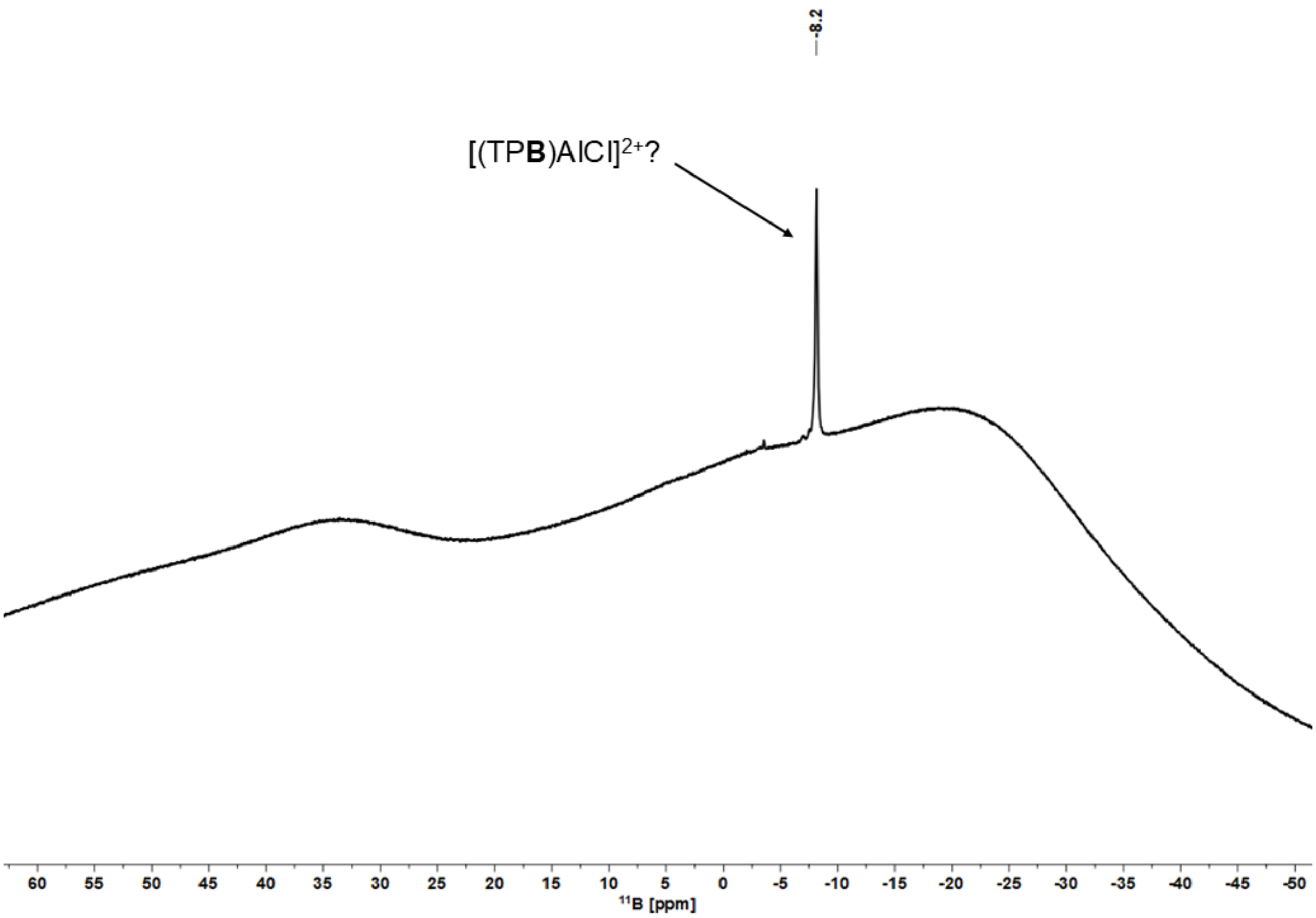

S-Figure 41: ^{11}B NMR spectrum (128.40 MHz, 1,2-DFB, 298 K, 3000 scans) of the isolated solid in the reaction of TPB with 3 equiv. $AlCl_3$.

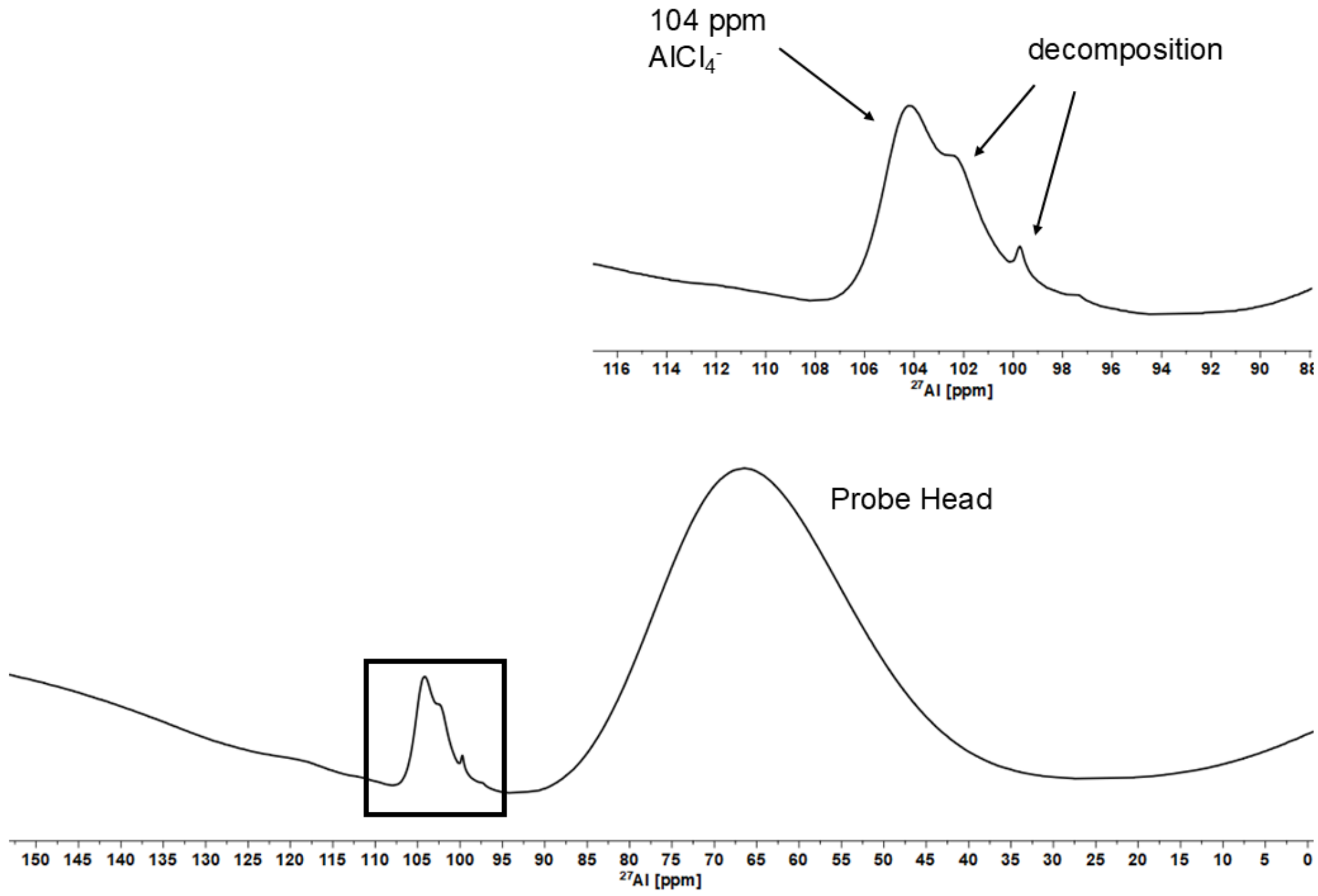

S-Figure 42: ^{27}Al NMR spectrum (104.28 MHz, 1,2-DFB, 298 K, 3000 scans) of the isolated solid in the reaction of TPB with 3 equiv. $AlCl_3$.

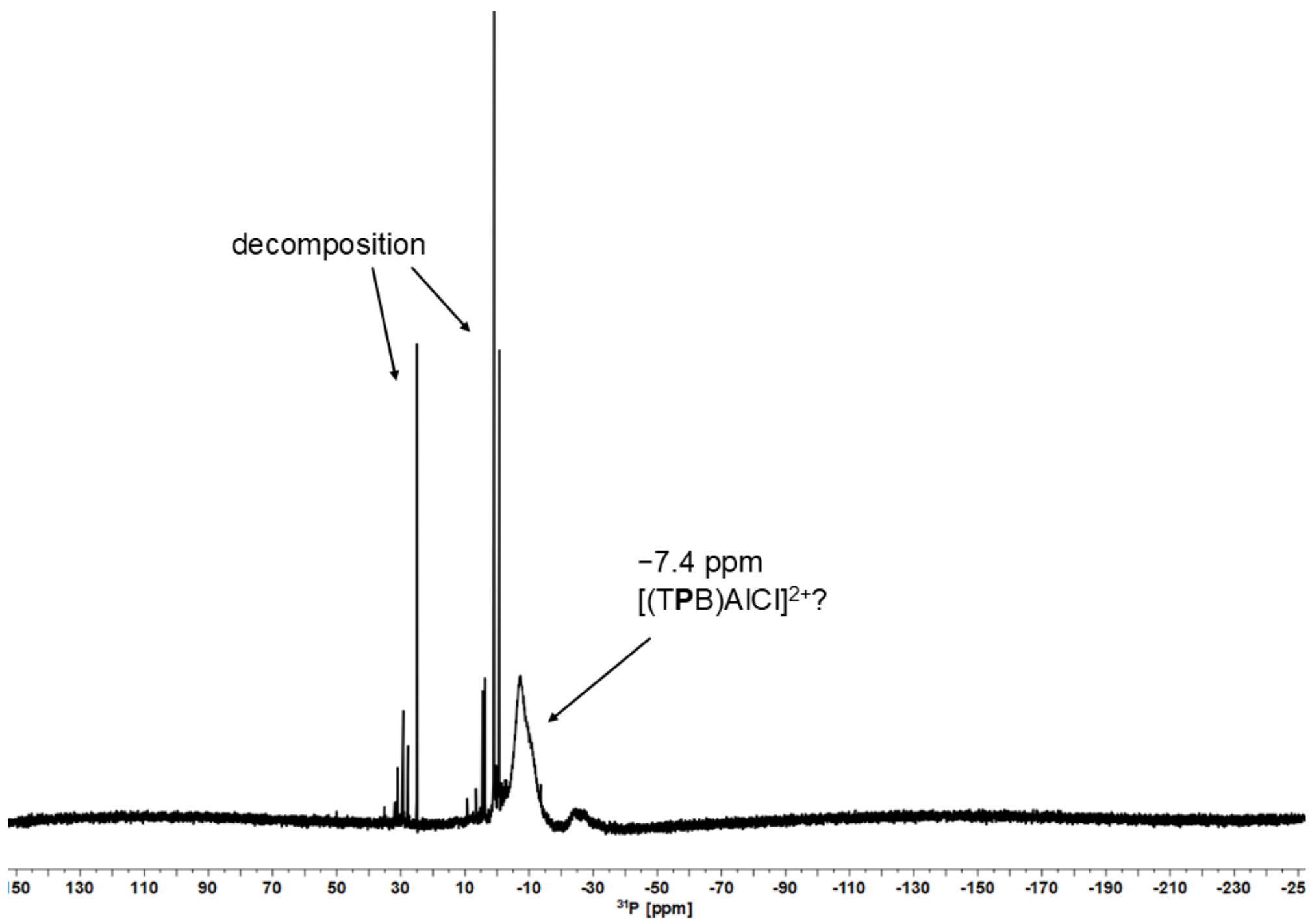

S-Figure 43: ^{31}P{^{1}H} NMR spectrum (162.00 MHz, 1,2-DFB, 298 K) of the isolated solid in the reaction of TPB with 3 equiv. $AlCl_3$.

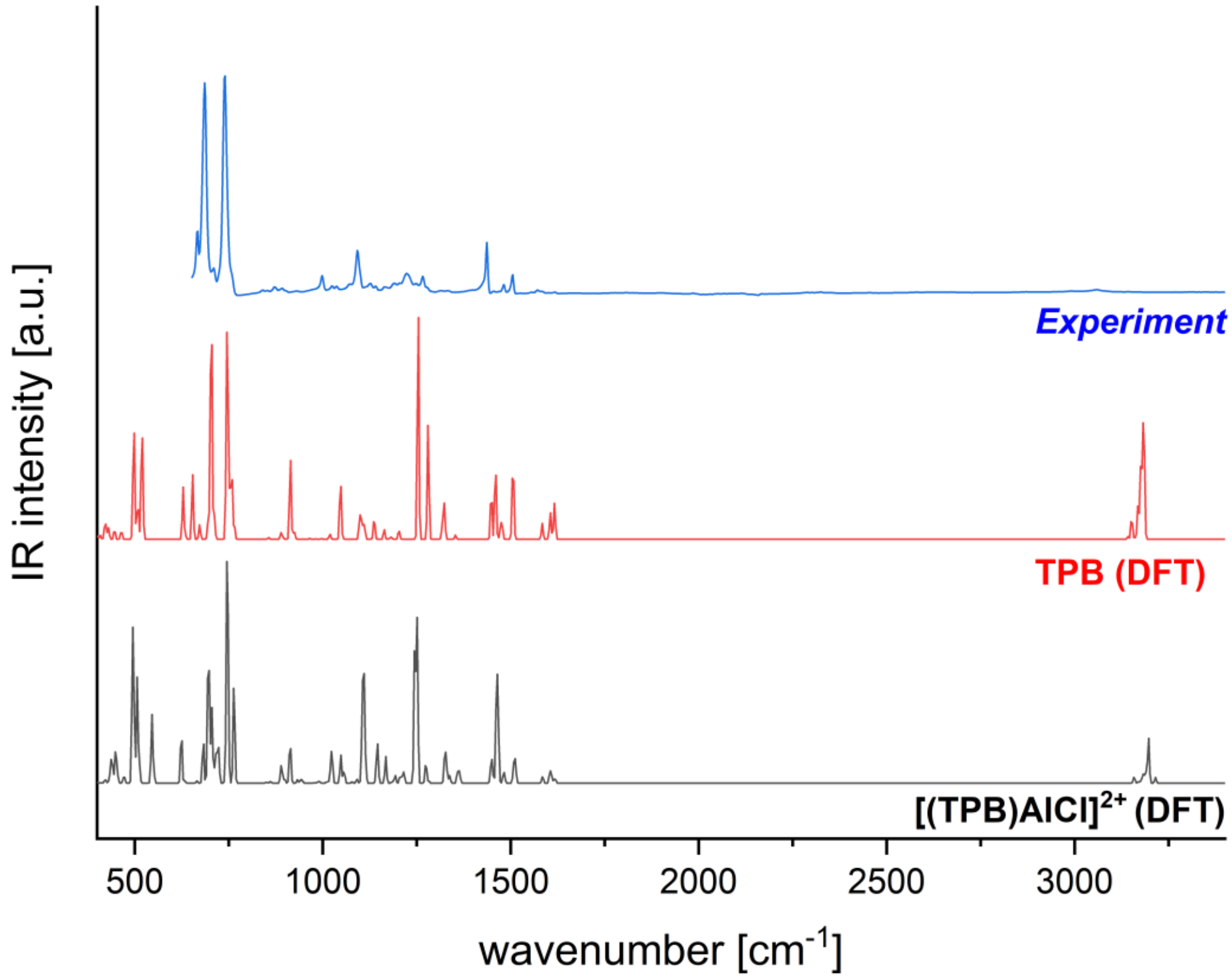

S-Figure 44: Comparison of the experimental IR spectrum (ATR, blue, top) of the isolated solid in the reaction of TPB with 3 equiv. $AlCl_3$ with the computed IR spectra of the free TPB ligand (red, middle) and $[(TPB)AlCl]^{2+}$ dication (black, bottom). Structures optimized at r^2SCAN-3c level of DFT with CPCM(fluorobenzene).

S-1.11 Attempted isolation of $[(TPB)AlBr]^{2+}([AlBr_4]^-)_2$ 2

Procedure: TPB (68.4 mg, 85.6 μmol) and $AlBr_3$ (68.4 mg, 257 μmol, 3.00 equiv.) were charged into a vial, into which 1,2-DFB (2 mL) was added. The mixture was stirred at room temperature for 15 min. The solvent was removed in vacuo, and the remaining solid was washed with pentane. The obtained yellow powder was analyzed by NMR spectroscopy, IR spectroscopy, and mass spectrometry. Crystallization attempts were unsuccessful. Crystallization from THF/pentane yielded the free ligand, indicating decomposition of the complex salt in donor solvents.

Analysis: ^{11}B, ^{27}Al, and ^{31}P NMR spectra of compound **2** were measured with a high scan number (**S-Figure 45**, **S-Figure 46**, **S-Figure 47S-Figure 46**). The ^{11}B NMR reveals an additional signal at −8.2 ppm, the ^{27}Al NMR spectrum shows a signal at −125.9 ppm, and the ^{31}P NMR spectrum a new signal at −4.0 ppm, which were assigned to the proposed dication $[(TPB)AlCl]^{2+}$. The presumed dicationic species is not stable in solution, as measurements with a high scan number show decomposition products in the NMR spectra. The mass spectrum only showed signals assignable to the protonated free ligand.

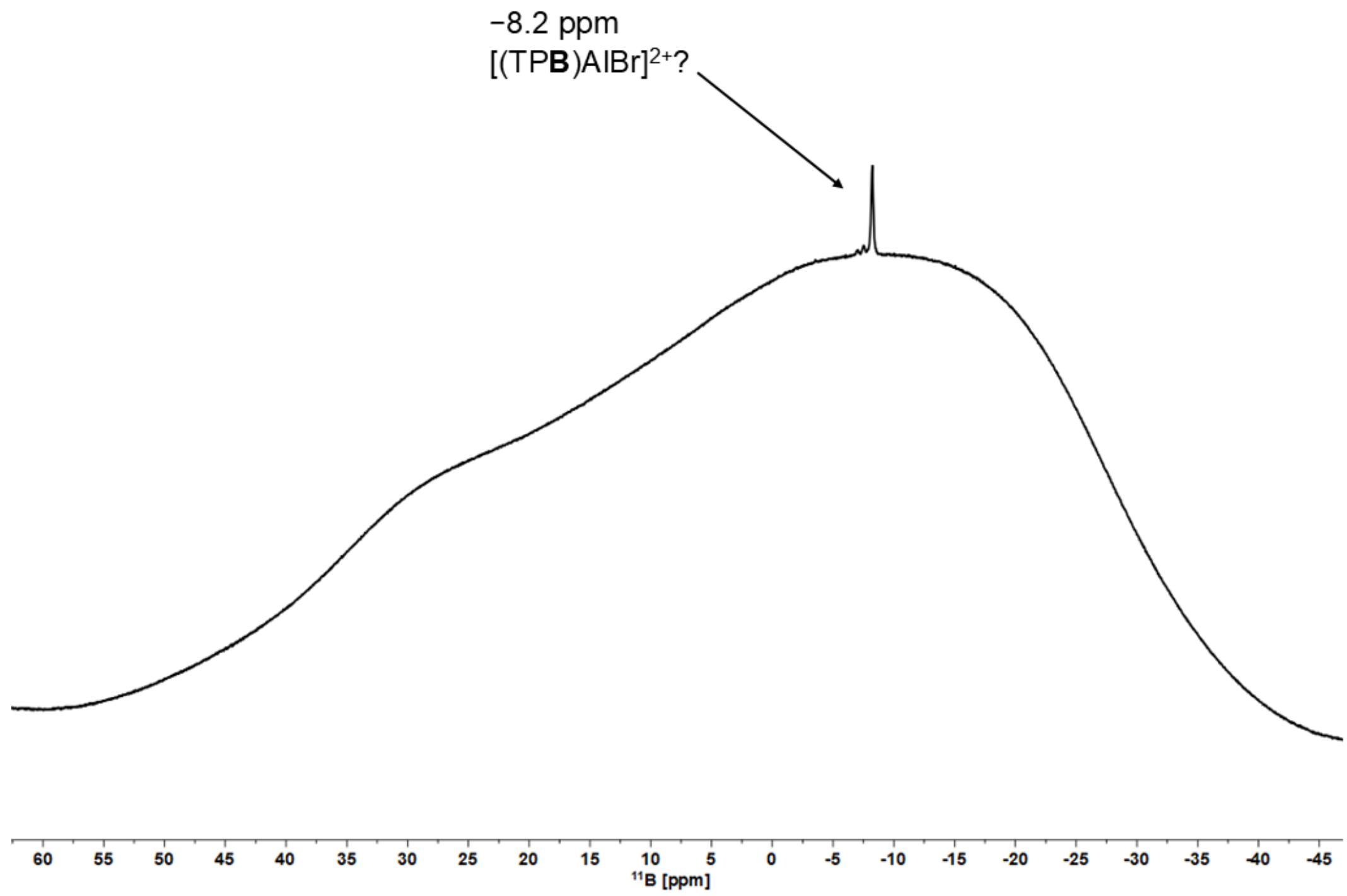

S-Figure 45: ^{11}B NMR spectrum (128.40 MHz, 1,2-DFB, 298 K, 3000 scans) of the isolated solid in the reaction of TPB with 3 equiv. $AlBr_3$.

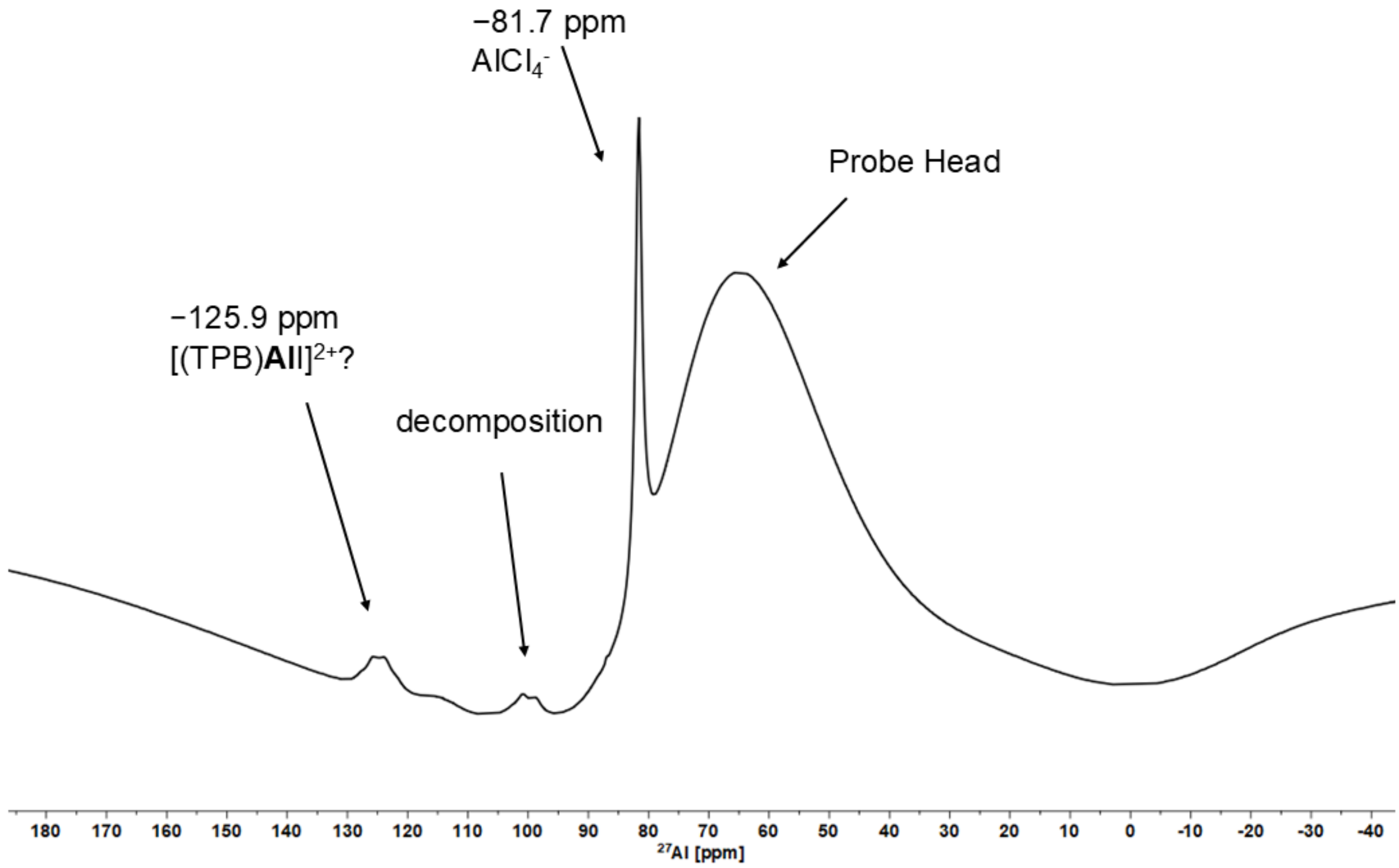

S-Figure 46: ^{27}Al NMR spectrum (104.28 MHz, 1,2-DFB, 298 K, 3000 scans) of the isolated solid in the reaction of TPB with 3 equiv. $AlBr_3$.

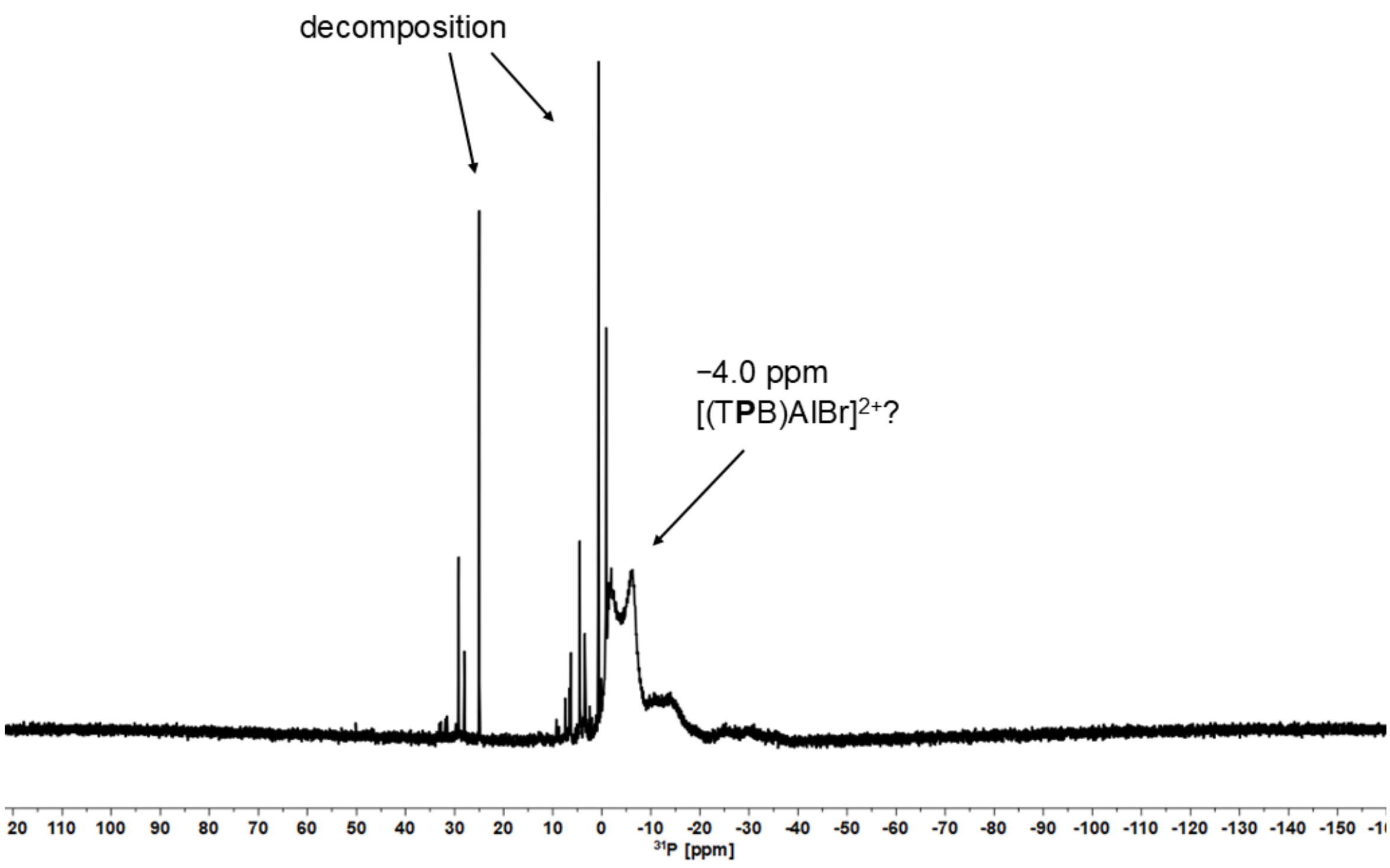

S-Figure 47: ^{31}P{^{1}H} NMR spectrum (162.00 MHz, 1,2-DFB, 298 K, 2000 scans) of the isolated solid in the reaction of TPB with 3 equiv. $AlBr_3$.

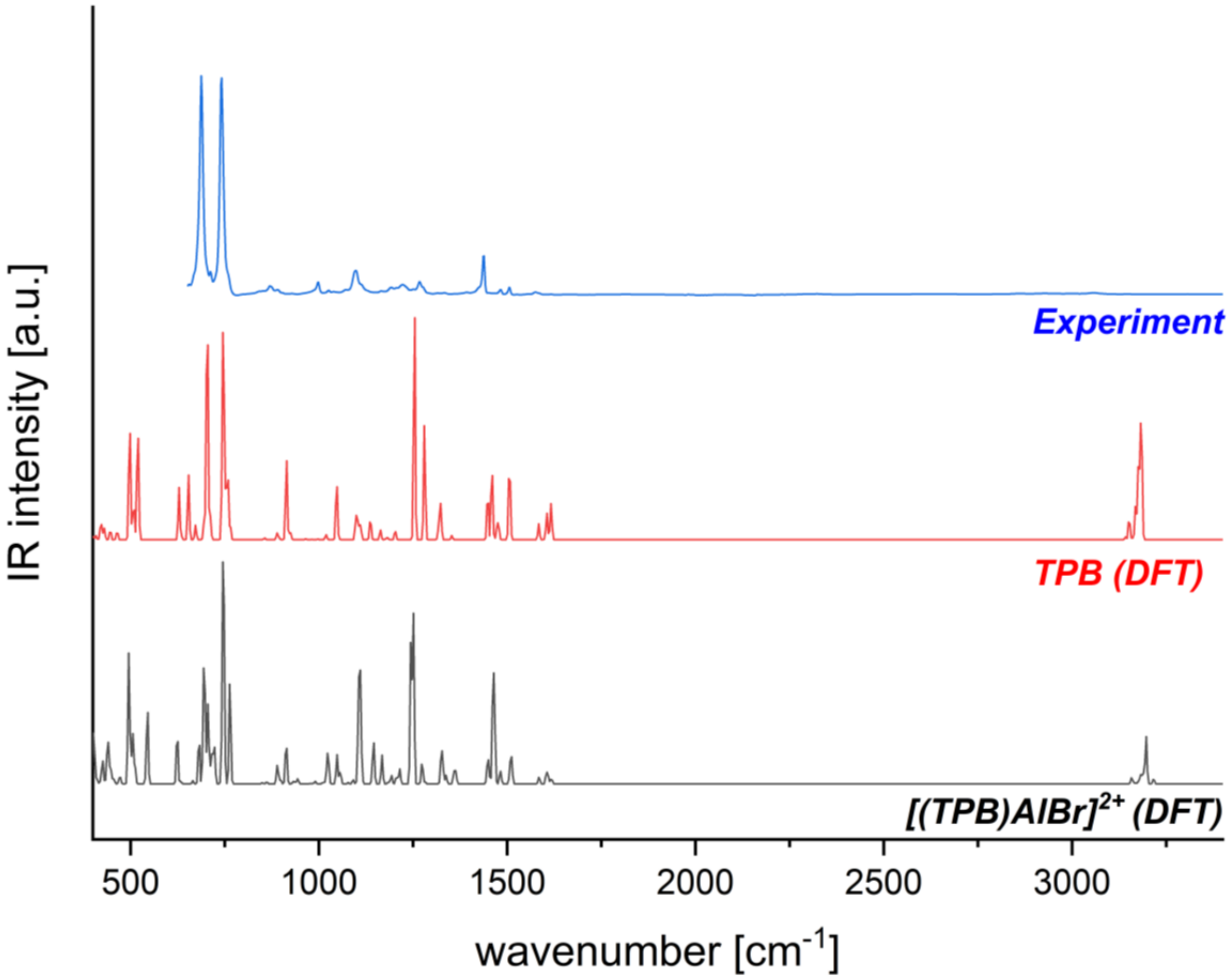

S-Figure 48: Comparison of the experimental IR spectrum (ATR, blue, top) of the isolated solid in the reaction of TPB with 3 equiv. $AlBr_3$ with the computed IR spectra of the free TPB ligand (red, middle) and $[(TPB)AlBr]^{2+}$ dication (black, bottom). Structures optimized at r^2SCAN-3c level of DFT with CPCM(fluorobenzene).

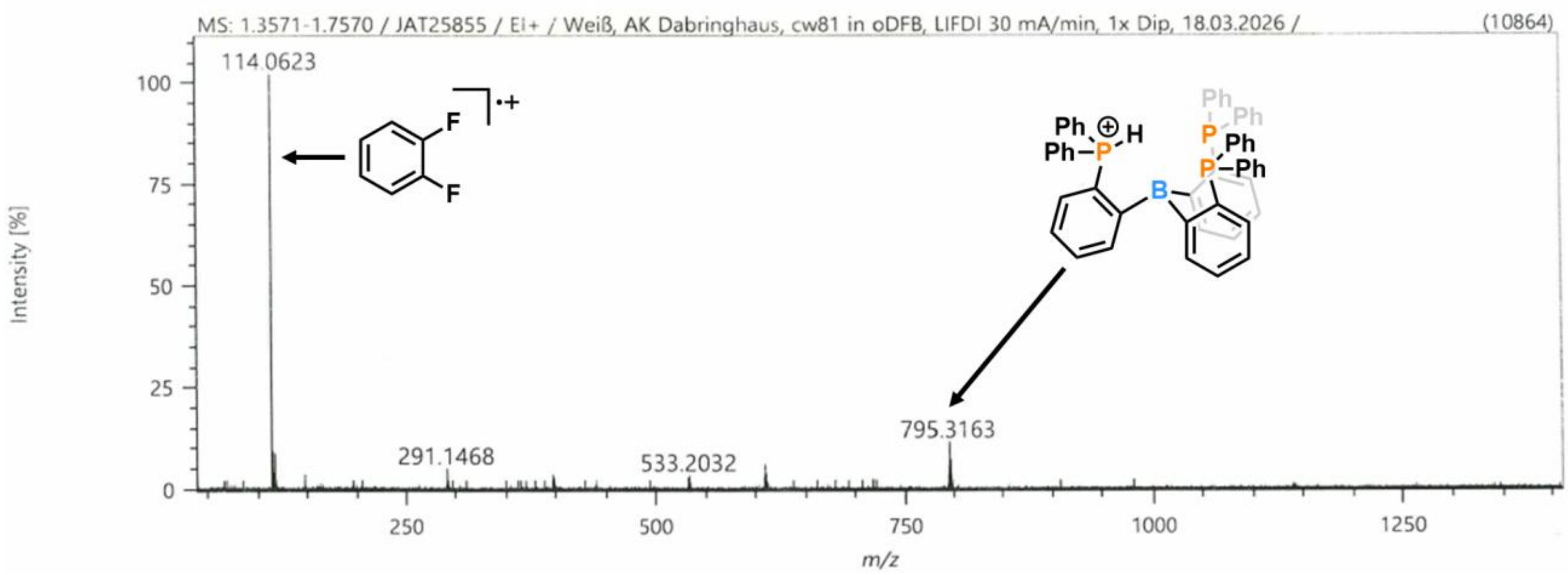

S-Figure 49: Mass spectrum (LIFDI, 1,2-DFB) of the isolated solid in the reaction of TPB with 3 equiv. $AlBr_3$.

S-1.12 Attempted isolation of $[(TPB)AlI]^{2+}([AlI_4]^-)_2$ 3

Procedure: TPB (10.0 mg, 12.6 µmol) and AlI_3 (15.4 mg, 37.8 µmol, 3.00 equiv.) were charged into a vial, into which 1,2-DFB (0.5 mL) was added. The mixture was stirred at room temperature for 15 min. The solvent was removed in vacuo, and the remaining solid was washed with pentane. The obtained yellow powder was analyzed by NMR and IR spectroscopy.

Analysis: ^{11}B, ^{27}Al, and ^{31}P NMR spectra of compound **3** were measured with a high scan number (**S-Figure 50**, **S-Figure 51**, **S-Figure 52**). The ^{11}B NMR reveals an additional signal at −8.3 ppm, the ^{27}Al NMR spectrum shows a signal at 117.1 ppm, and the ^{31}P NMR spectrum a new signal at −5.7 ppm, which were assigned to the proposed dication $[(TPB)AlI]^{2+}$ **3**. The presumed dicationic species is not stable in solution, as measurements with a high scan number show decomposition products in the NMR spectra.

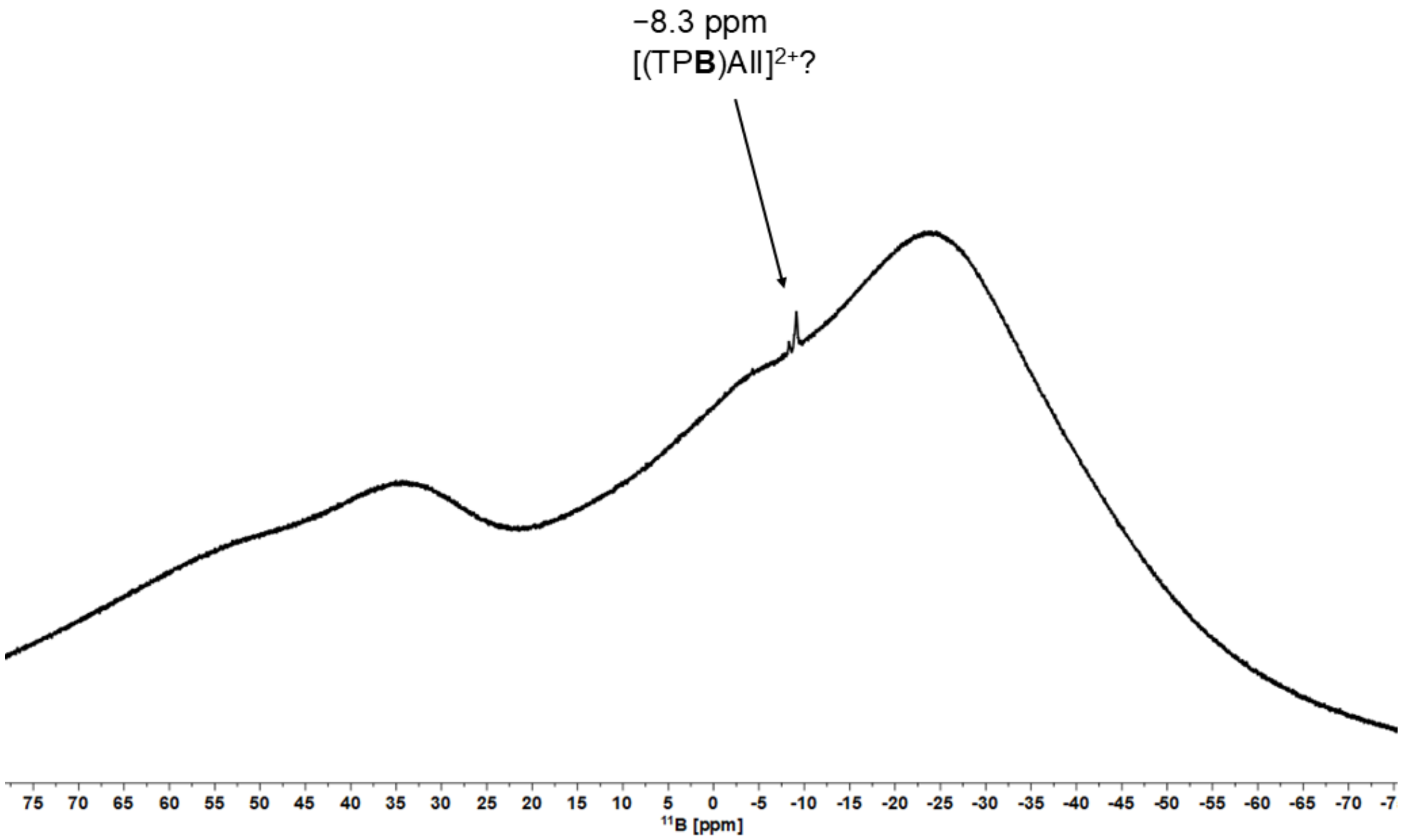

S-Figure 50: ^{11}B NMR spectrum (162.00 MHz, 1,2-DFB, 298 K, 3000 scans) of the isolated solid in the reaction of TPB with 3 equiv. AlI_3.

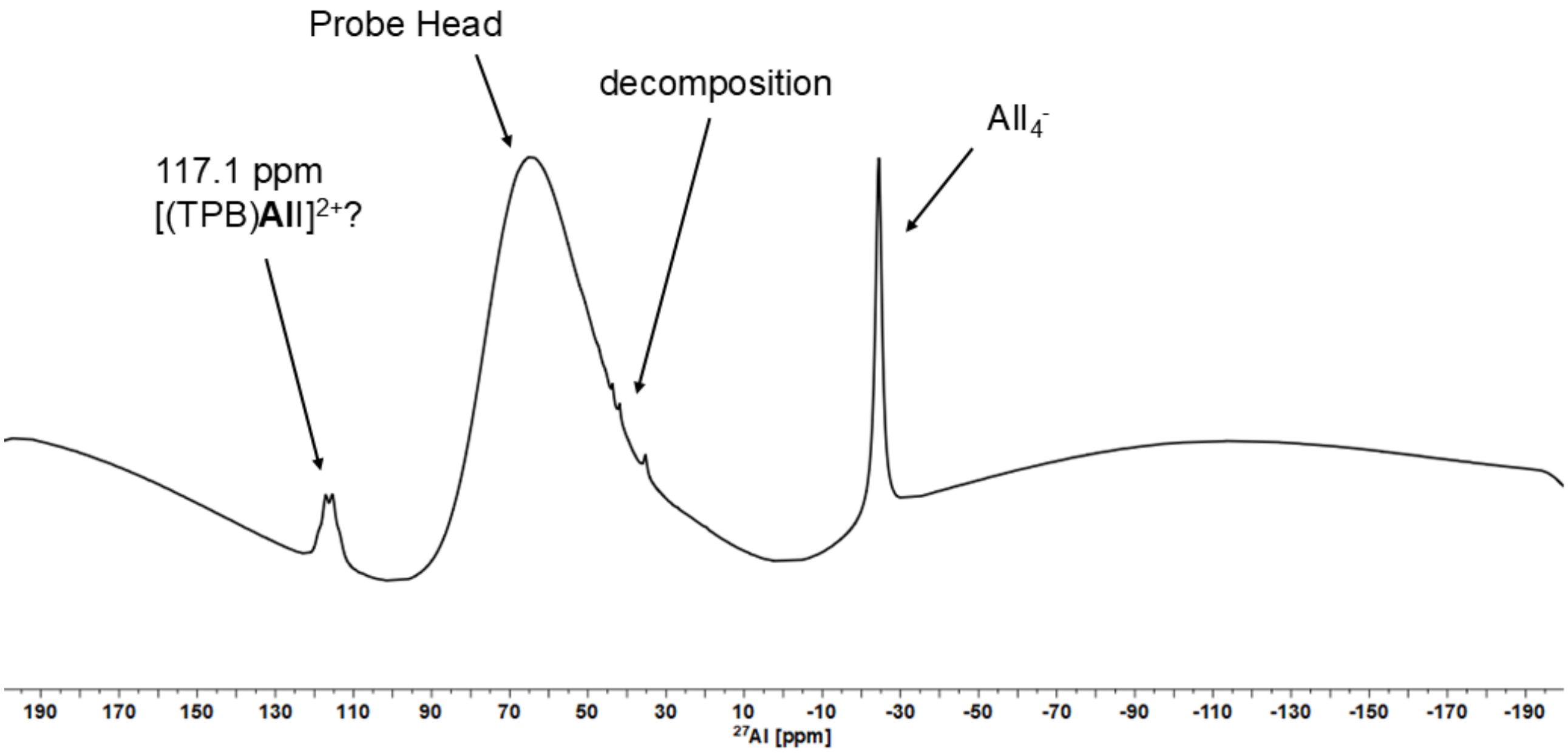

S-Figure 51: ^{27}Al NMR spectrum (104.28 MHz, 1,2-DFB, 298 K) of the isolated solid in the reaction of TPB with 3 equiv. AlI_3.

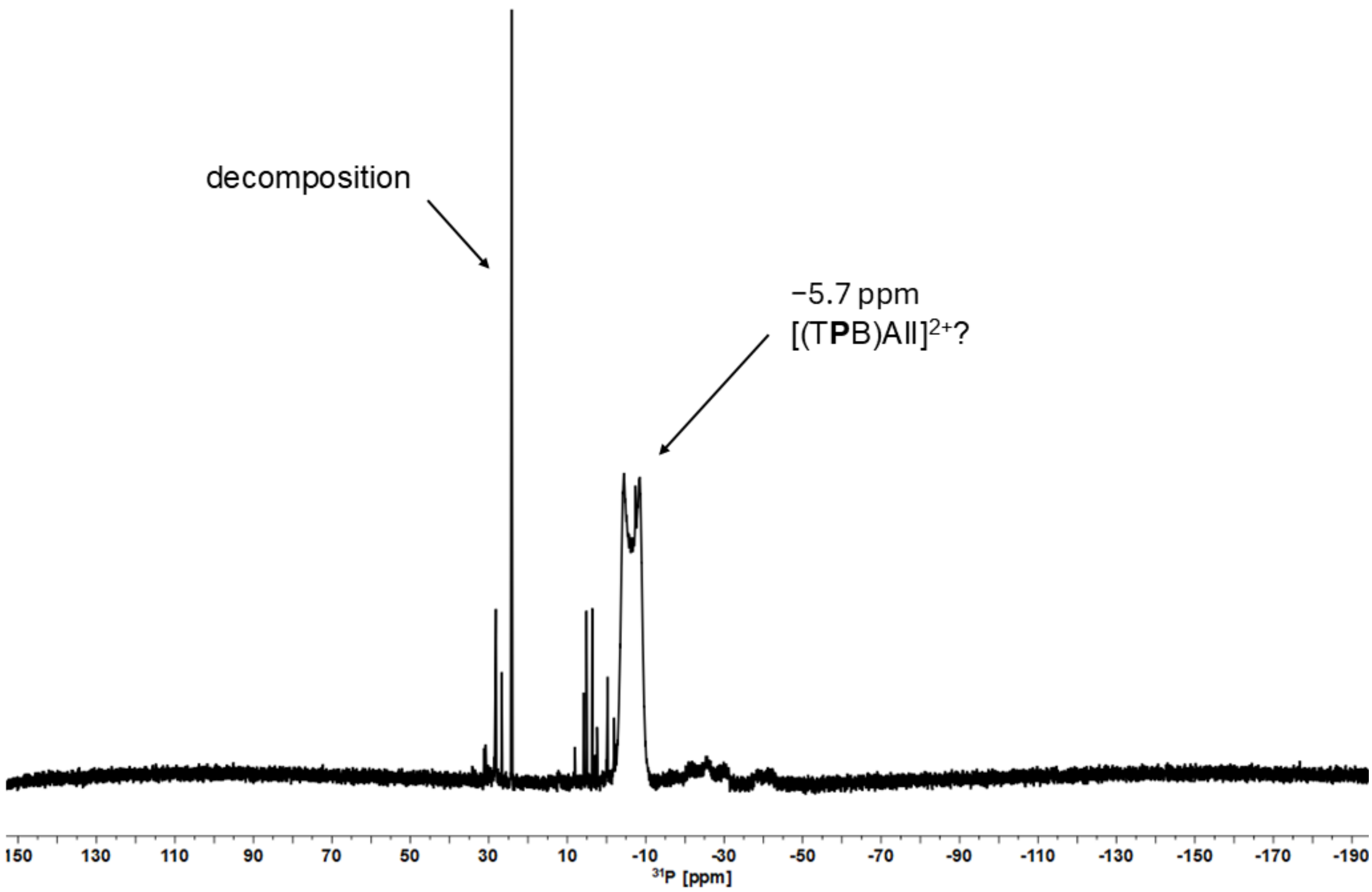

S-Figure 52: ^{31}P{^{1}H} NMR spectrum (162.00 MHz, 1,2-DFB, 298 K) of the isolated solid in the reaction of TPB with 3 equiv. AlI_3.

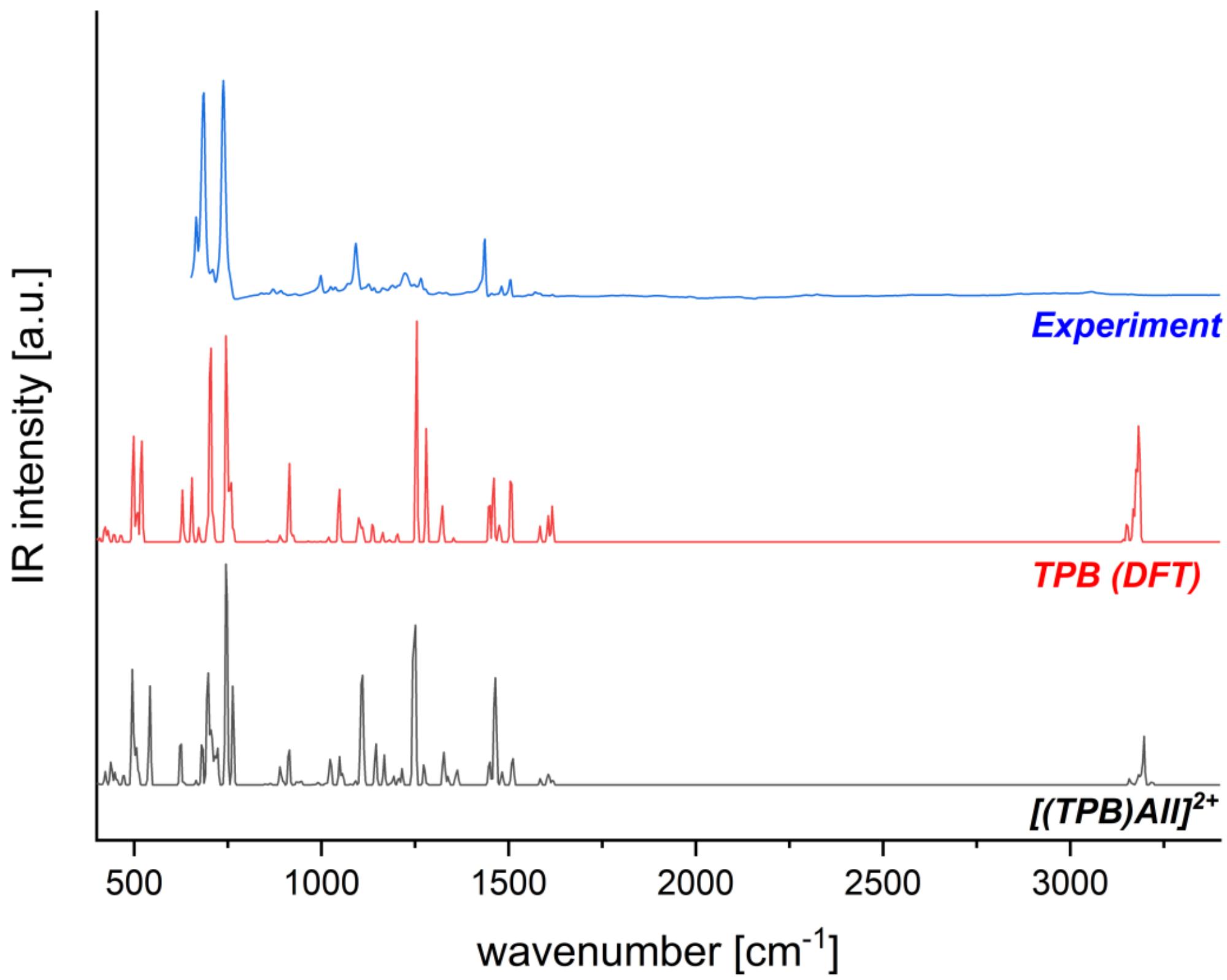

S-Figure 53: Comparison of the experimental IR spectrum (ATR, blue, top) of the isolated solid in the reaction of TPB with 3 equiv. AlI_3 with the computed IR spectra of the free TPB ligand (red, middle) and $[(TPB)AlI]^{2+}$ dication (black, bottom). Structures optimized at r^2SCAN-3c level of DFT with CPCM(fluorobenzene).

S-1.13 Preparation of [(TPB)AlH] 7

Procedure: [(TPB)AlBr] (50.0 mg, 55.5 µmol) and $LiAlH_4$ (2.1 mg, 56 µmol, 1.0 equiv.) were charged into a vial, into which Et_2O (0.5 mL) was added. The mixture was stirred for 10 min yielding a bright orange precipitate. Then, the solvent was removed in vacuo and the orange residue was dissolved in benzene (2 mL), filtered off and the solvent of the filtrate was removed in vacuo. The residual solid was washed with pentane (5 mL) and dried thoroughly in vacuo to yield compound **6** as orange powder (35.0 mg, 42.6 µmol, 77 %). Crystals were obtained by vapor diffusion of *n*-pentane onto the C_6D_6 solution.

^{1}H NMR spectrum (399.89 MHz, C_6D_6, 298 K): δ = 5.82 (q, 1H, $^{2}J_{H,P}$ = 50.7 Hz, Al−H), 6.74-6.79 (m, 4H, −P(C_6H_4)B), 6.81-6.98 (m, 13H, −PPh_2), 7.00-7.04 (m, 10H, −P(C_6H_4)B/−PPh_2), 7.18-7.21 (m, 12H, −PPh_2), 7.54-7.55 (m, 3H, −P(C_6H_4)B) ppm.

^{13}C NMR spectrum (100.56 MHz, C_6D_6, 298 K): δ = 123.7 (−P(C_6H_4)B), 128.3 (−P(C_6H_4)B), 128.8 (−PPh_2), 132.6 (−P(C_6H_4)B), 133.8 (−PPh_2), 136.3 (−P(C_6H_4)B) ppm.

^{11}B NMR spectrum (128.30 MHz, C_6D_6, 298 K): δ = −6.0 (q, $^{3}J_{B,P}$ = 30.4 Hz) ppm.

^{31}P{^{1}H} NMR spectrum (161.89, C_6D_6, 298 K): δ = −17.6 (q, $^{3}J_{B,P}$ = 28.1 Hz) ppm.

IR spectrum (128 scans, ATR, Diamond): ν = 658 (vw), 675 (s), 694 (vs), 739 (vs), 744 (s), 831 (vw), 843 (vw), 876 (vw), 916 (vw), 999 (vw), 1026 (vw), 1058 (vw), 1069 (vw), 1096 (w), 1118 (vw), 1155 (vw), 1183 (vw), 1245 (vw), 1264 (vw), 1306 (vw), 1327 (vw), 1421 (w), 1434 (m), 1479 (w), 1544 (vw), 1569 (vw), 1585 (vw), 1791 (w, Al−H), 3034 (vw), 3051 (vw) cm^{-1}.

UV-Vis (toluene, 500 µM, RT): λ_{max} = 364 (ε = 13852.2 $Lmol^{-1}cm^{-1}$) nm.

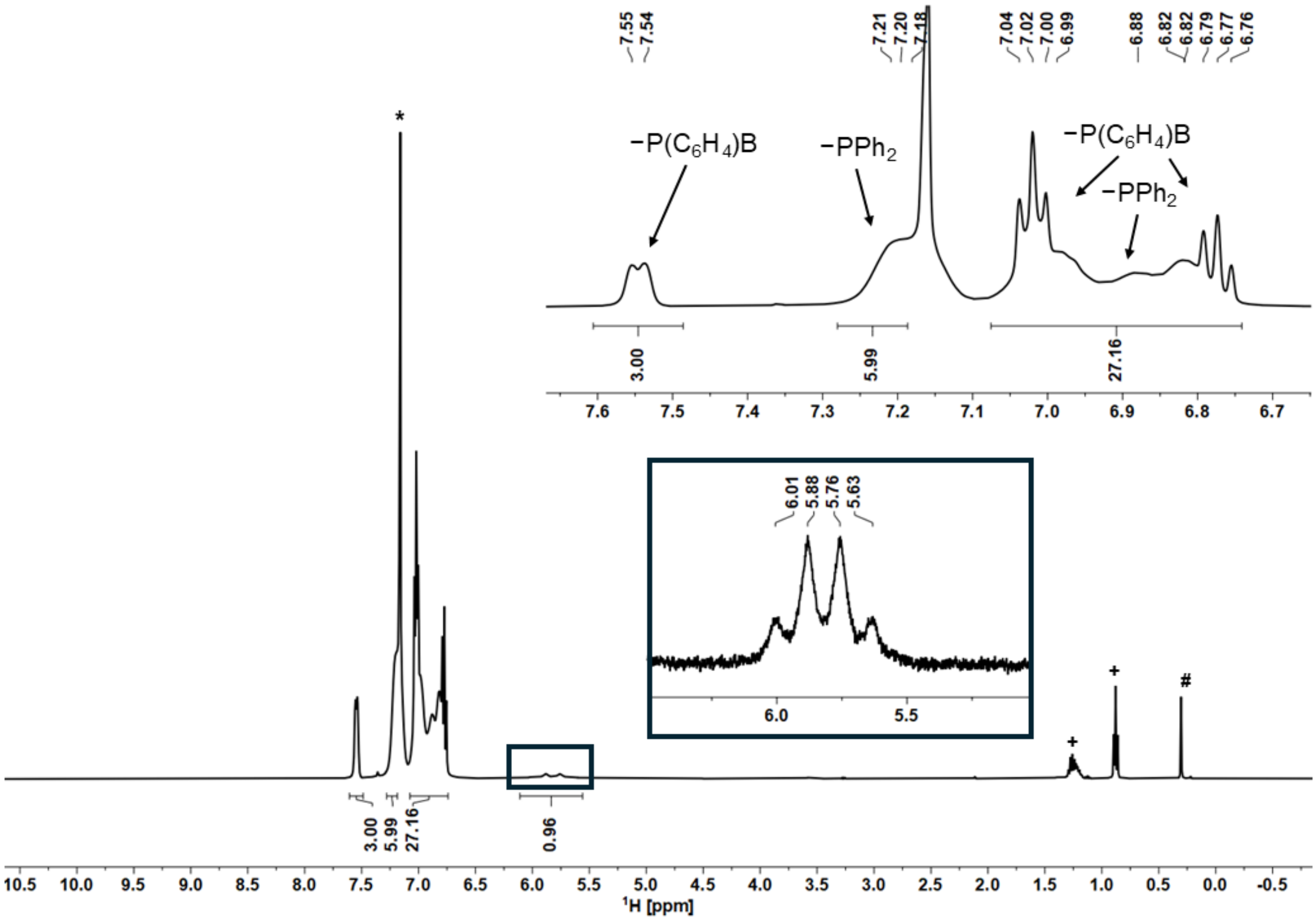

S-Figure 54: ^{1}H NMR spectrum (399.89 MHz, C_6D_6, 298 K) of [(TPB)AlH] **7**. C_6D_6 is marked with *, silicon grease with #, and residual pentane with +.

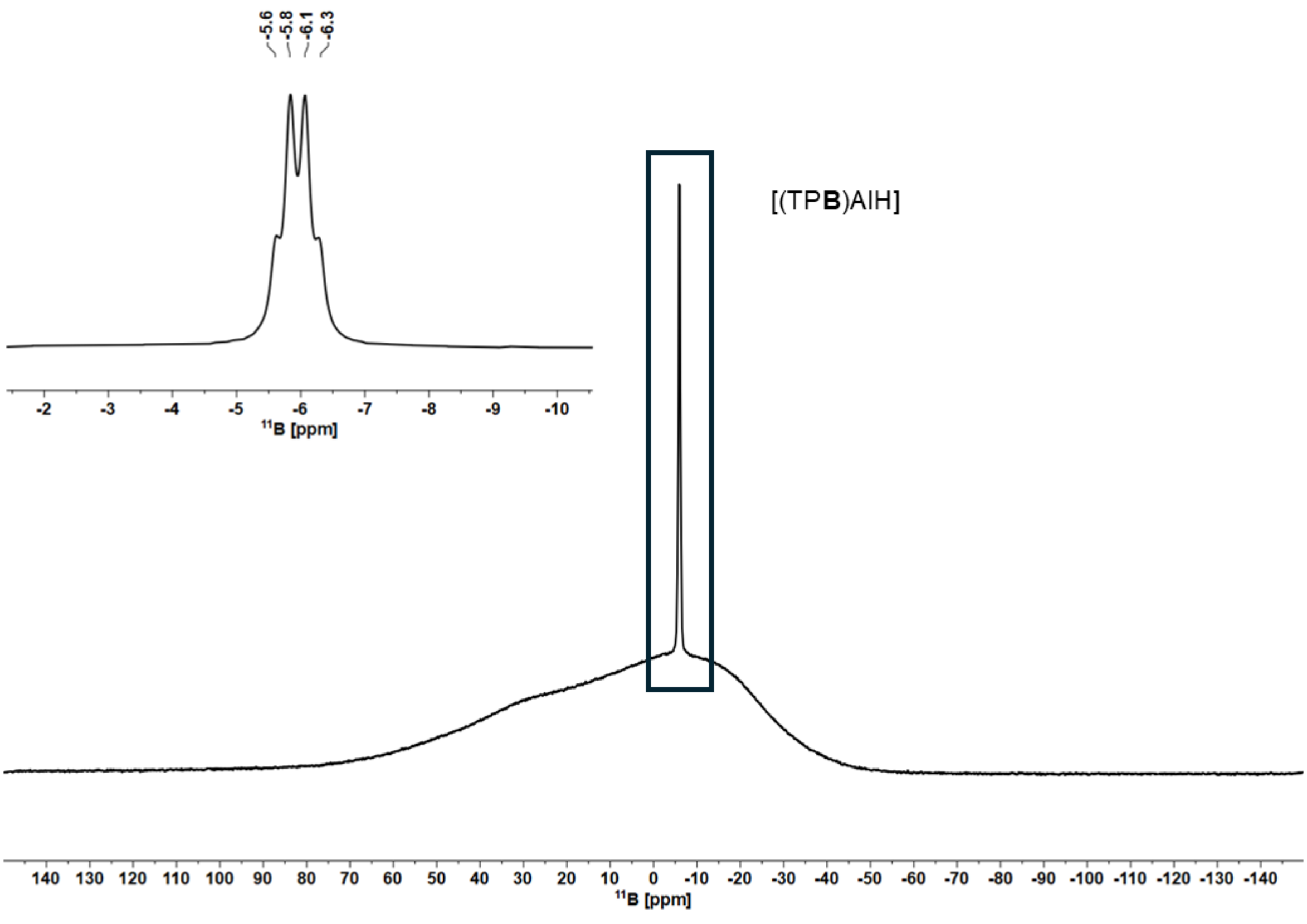

S-Figure 55: ^{11}B NMR spectrum (128.30 MHz, C_6D_6, 298 K) of [(TPB)AlH] **7**.

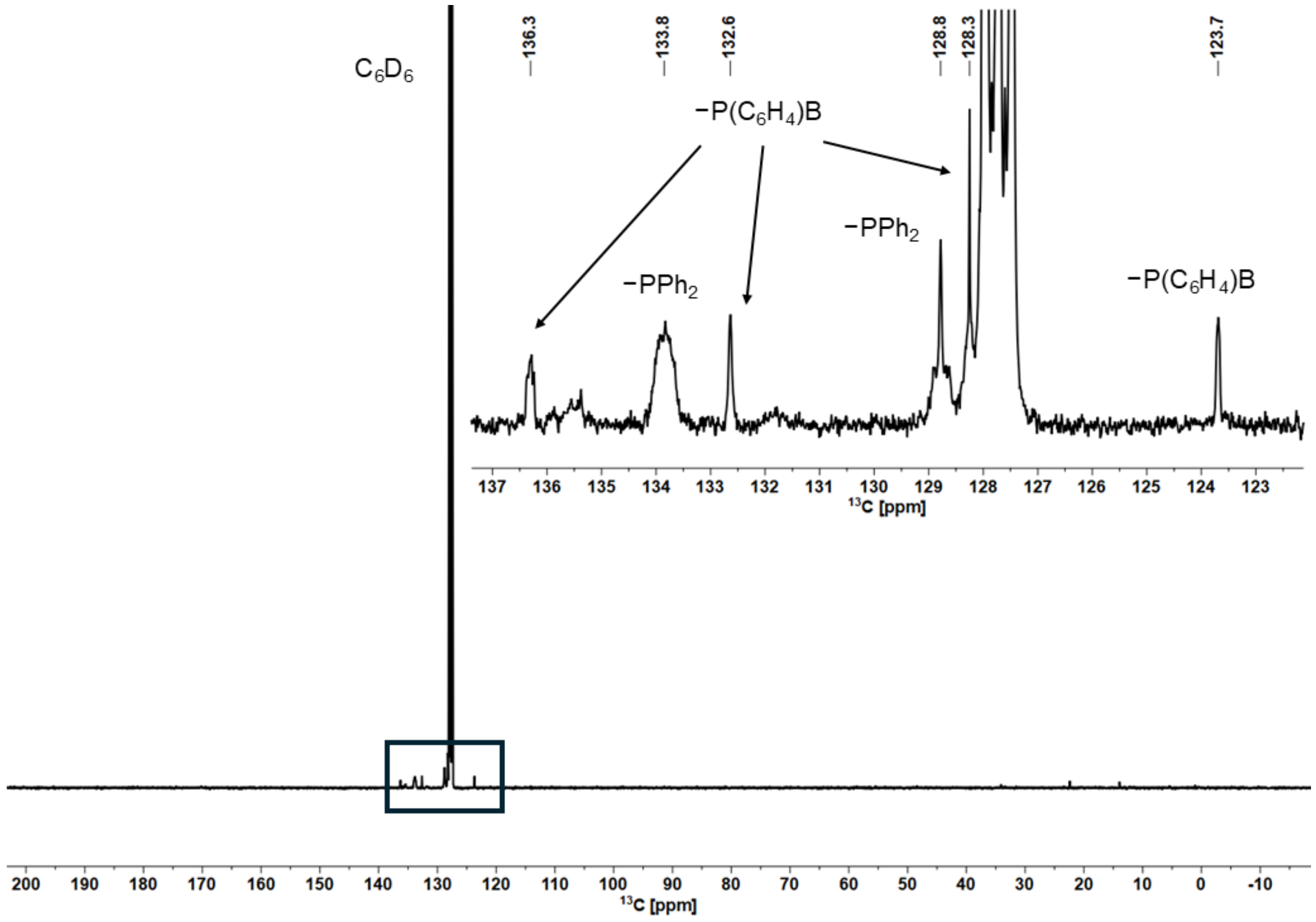

S-Figure 56: ^{13}C NMR spectrum (100.56 MHz, C_6D_6, 298 K) of [(TPB)AlH] **7**.

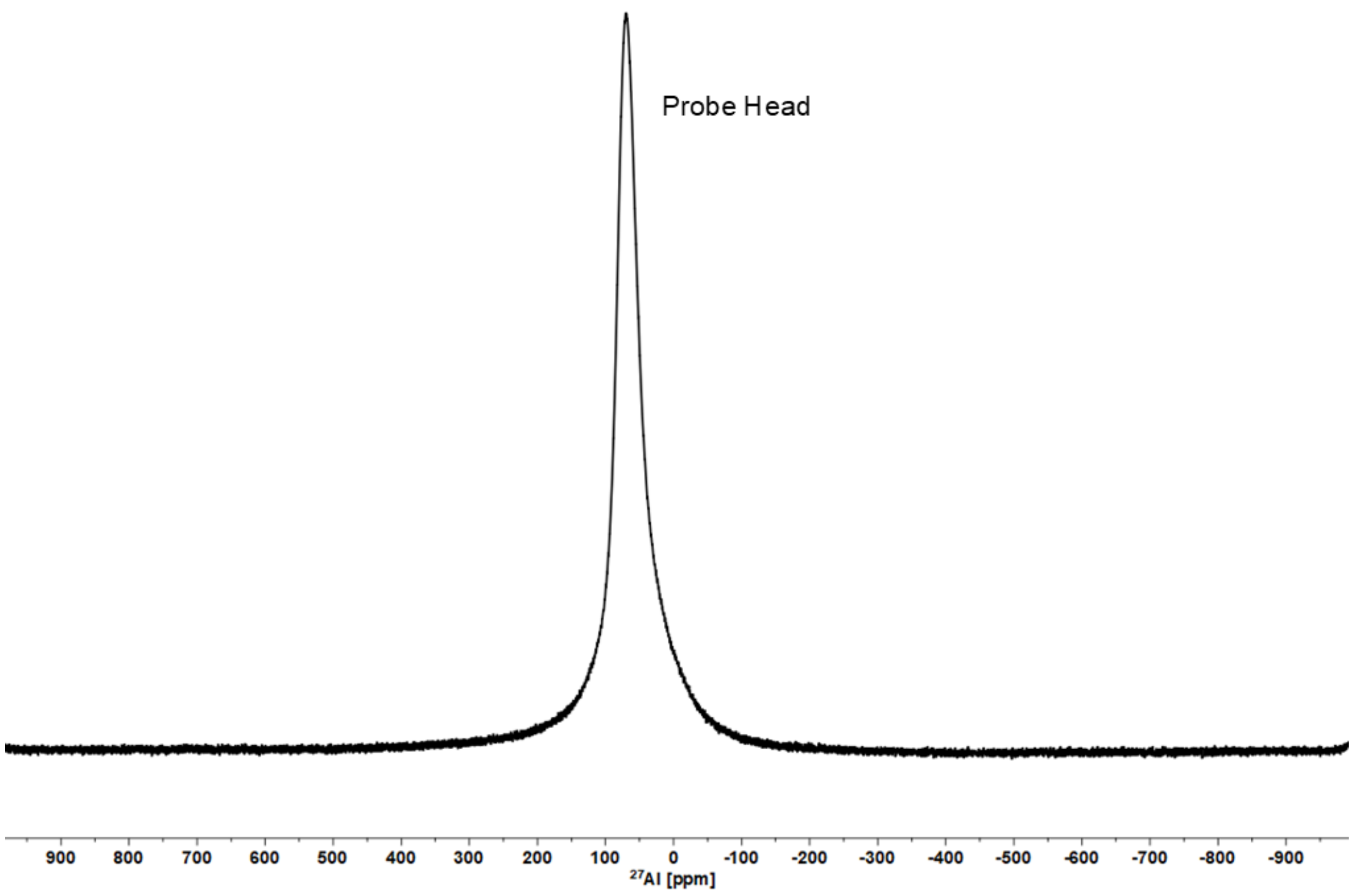

S-Figure 57: ^{27}Al NMR spectrum (104.20 MHz, C_6D_6, 298 K) of [(TPB)AlH] **7**.

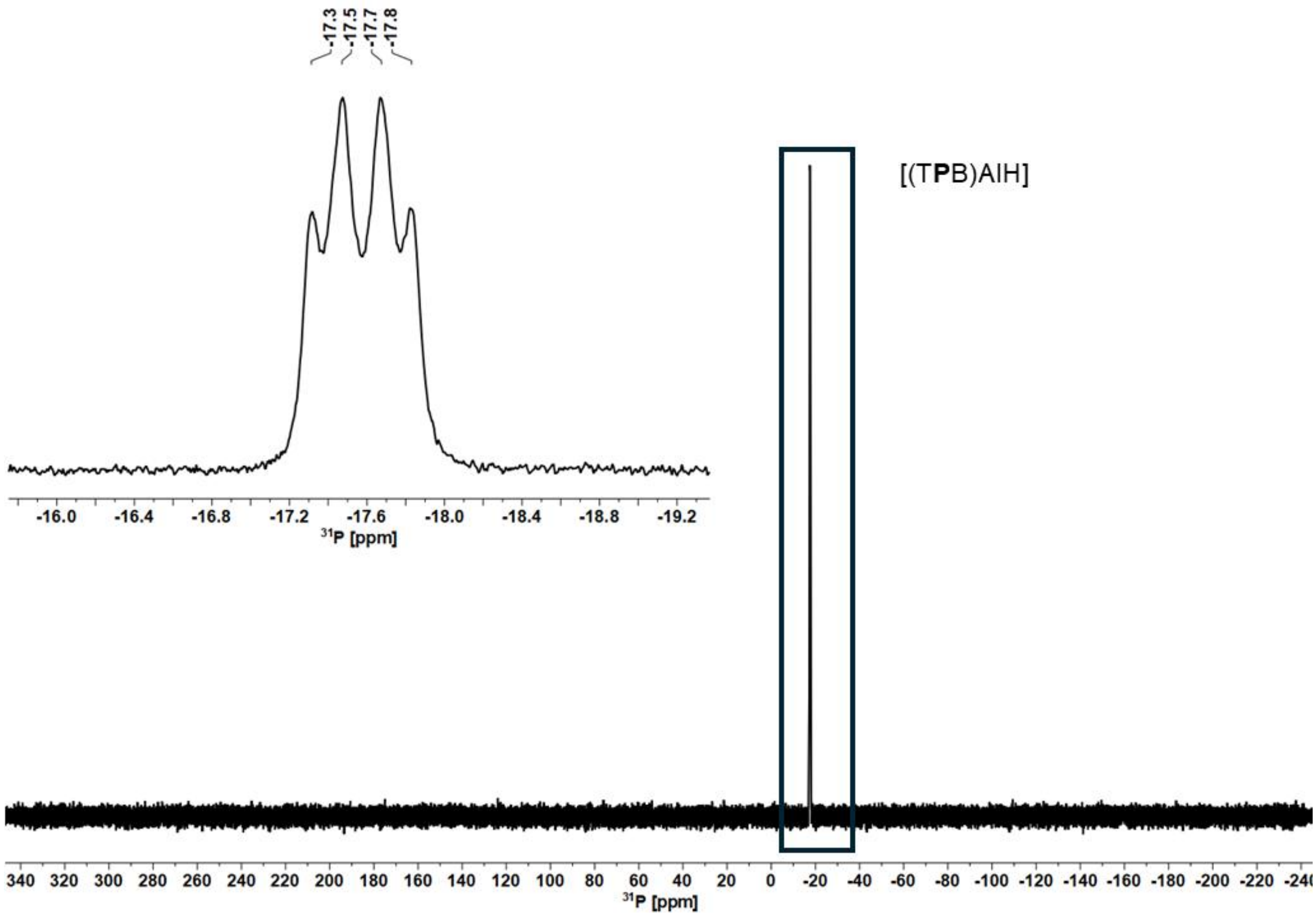

S-Figure 58: ^{31}P NMR spectrum (161.89 MHz, C_6D_6, 298 K) of [(TPB)AlH] **7**.

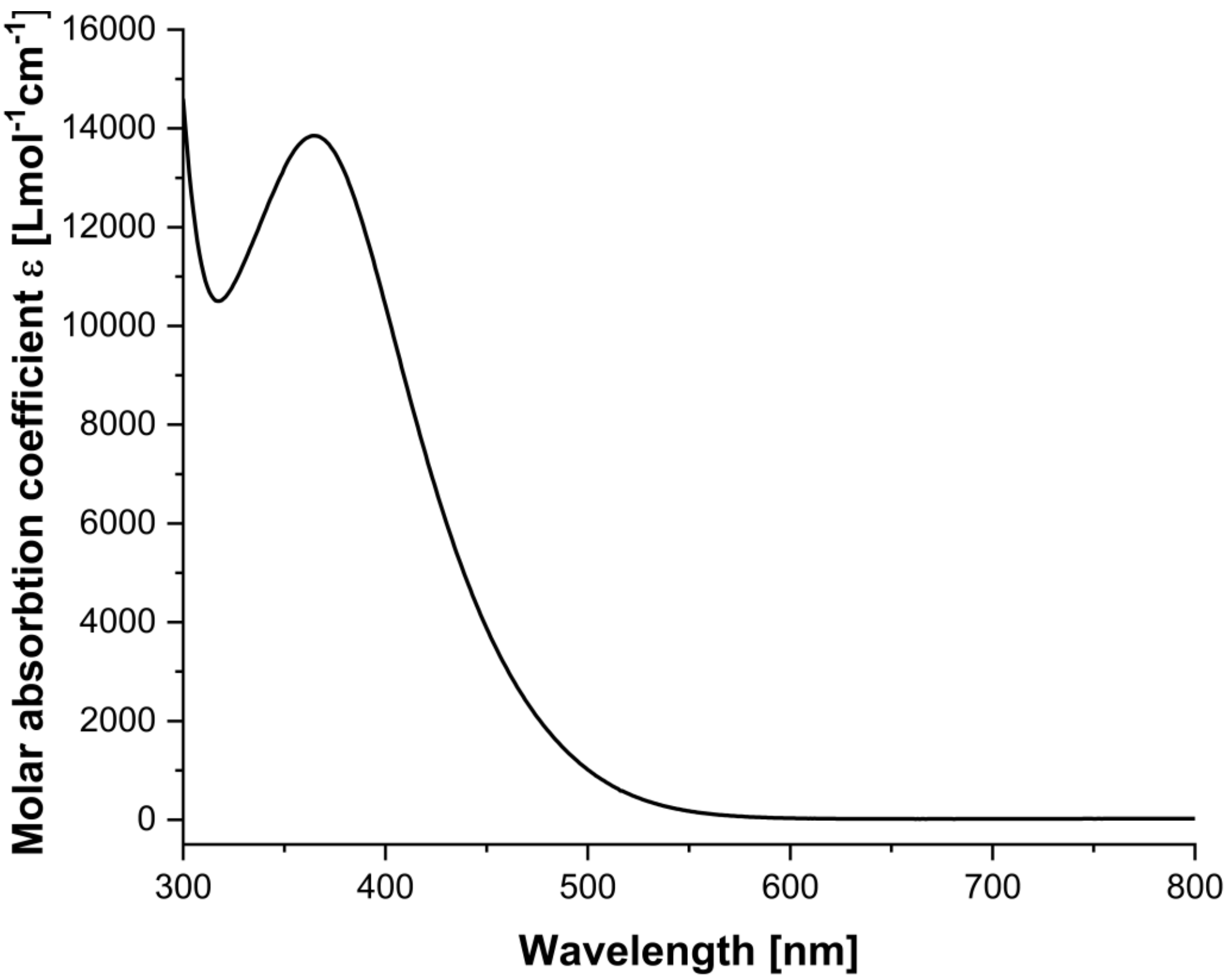

S-Figure 59: UV-Vis spectrum (toluene, 500 µM, RT) of [(TPB)AlH] **7**.

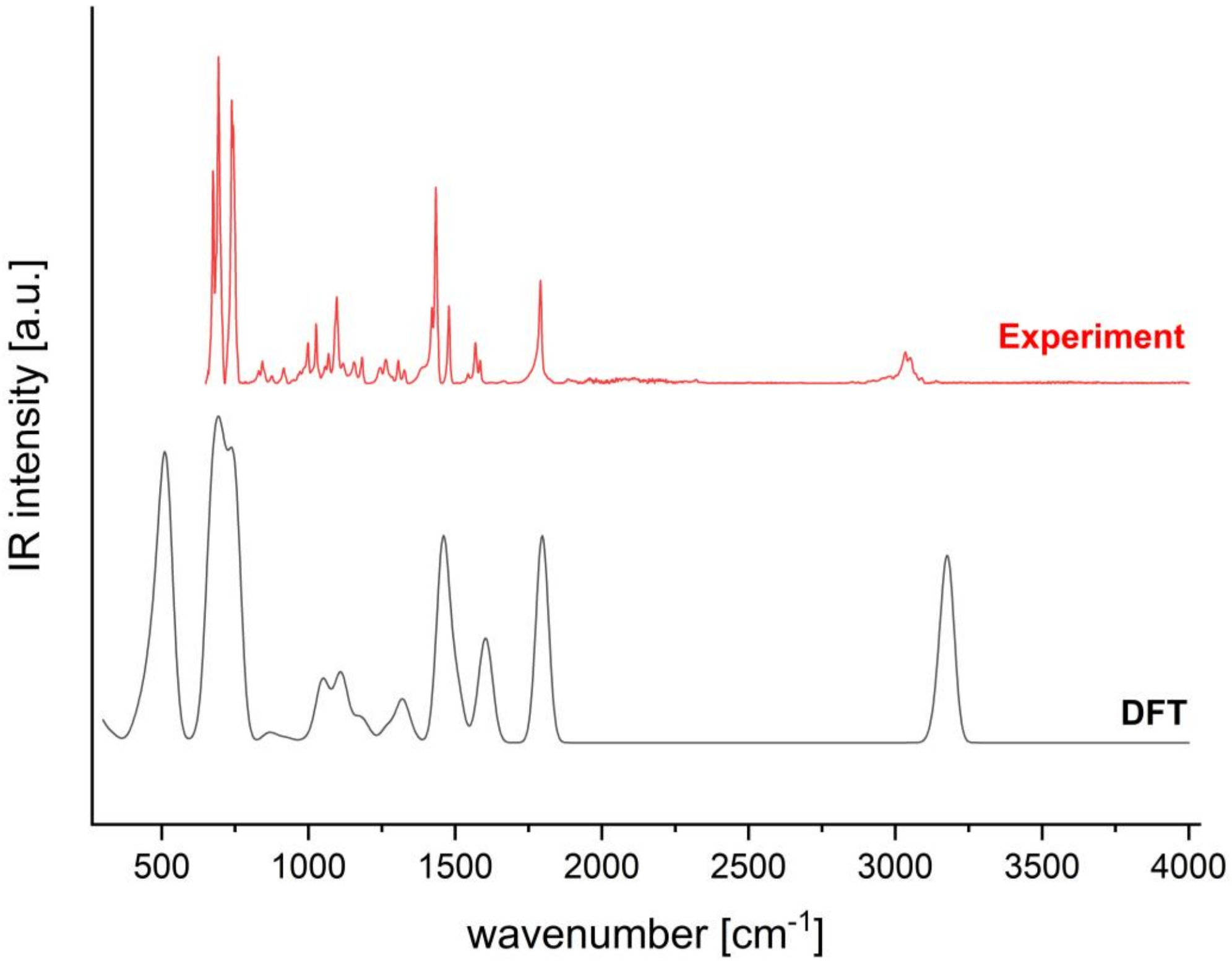

S-Figure 60: Comparison of the experimental IR spectrum (ATR, red, top) of [(TPB)AlH] **7** with the computed IR spectra of **7** (black, bottom). Structures optimized at r^{2}SCAN-3c level of DFT with CPCM(fluorobenzene).

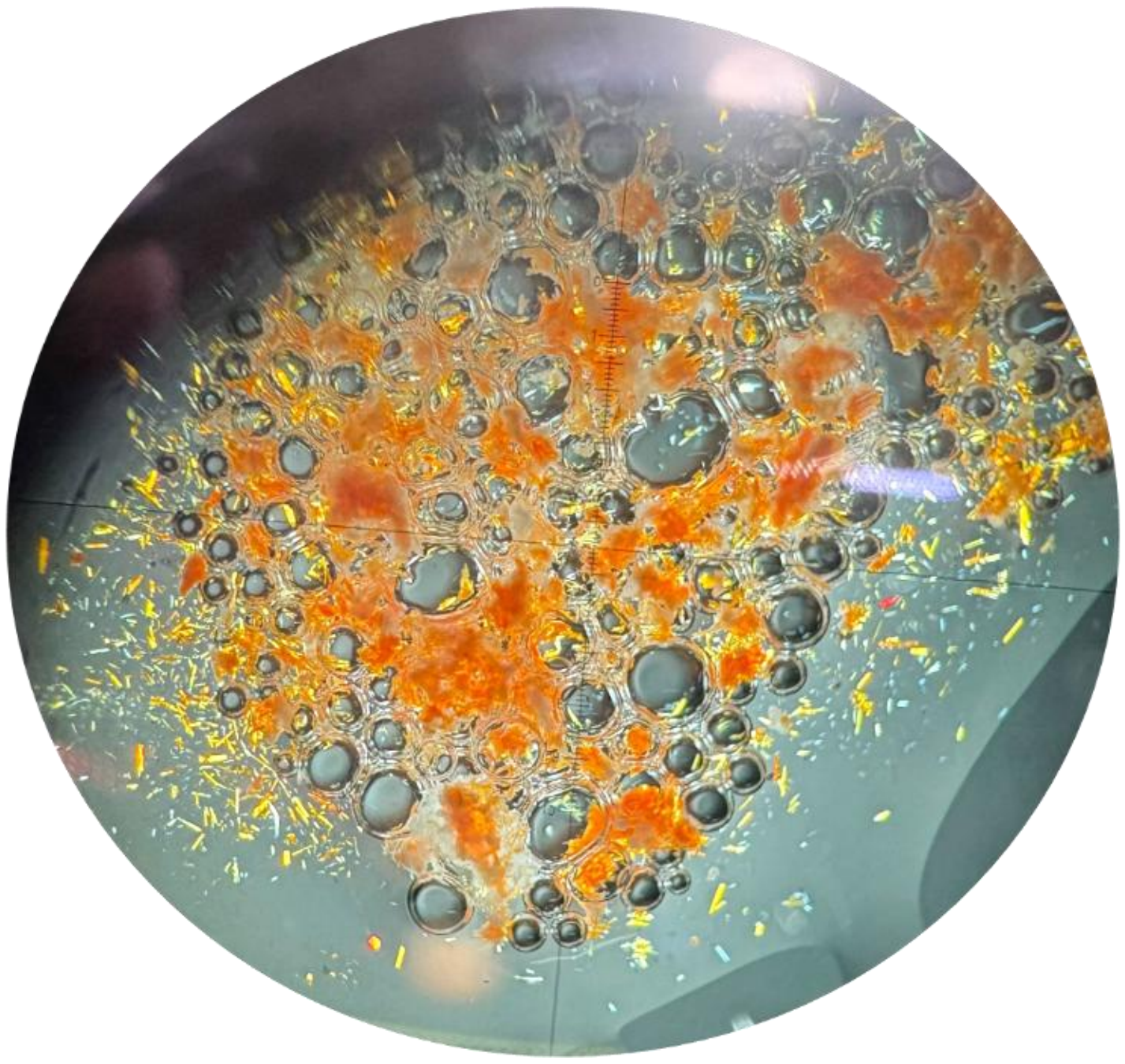

S-Figure 61: Picture of crystals of [(TPB)AlH] **7** obtained from crystallization of the reaction mixture.

S-1.14 Preparation of [(TPB)AlBr(DMAP)] 8

Procedure: [(TPB)AlBr] (10.0 mg, 11.1 µmol) was dissolved in 0.6 mL C_6D_6 and DMAP (1.4 mg, 11 µmol, 1.0 equiv.) was added yielding a bright yellow solution. The solution was layered with pentane. [(TPB)AlBr(DMAP)] **8** was isolated as yellow crystals (8.5 mg, 8.29 µmol, 75 %). scXRD-quality crystals were obtained by vapor diffusion of *n*-pentane onto a THF solution.

^{1}H NMR spectrum (600.15 MHz, THF-d_8, 298 K): δ = 2.87 (s, 6H, −CH_3), 5-95-8.28 (m) ppm.

^{11}B NMR spectrum (192.55 MHz, THF-d_8, 298 K): δ = −9.3 (q, $^{3}J_{B,P}$ = 27.0 Hz) ppm.

^{31}P{^{1}H} NMR spectrum (242.96, THF-d_8, 298 K): δ = −10.6 (s, −PPh_3), −24.9 (s, Al−PPh_3), −23.9 (s, Al−PPh_3) ppm.

Comment: Due to poor solubility of [(TPB)AlBr(DMAP)] **8** in THF-d_8 the concentration of the NMR samples was too low to identify ^{13}C NMR signals and assign the signals of the aromatic region of the ^{1}H NMR spectrum.

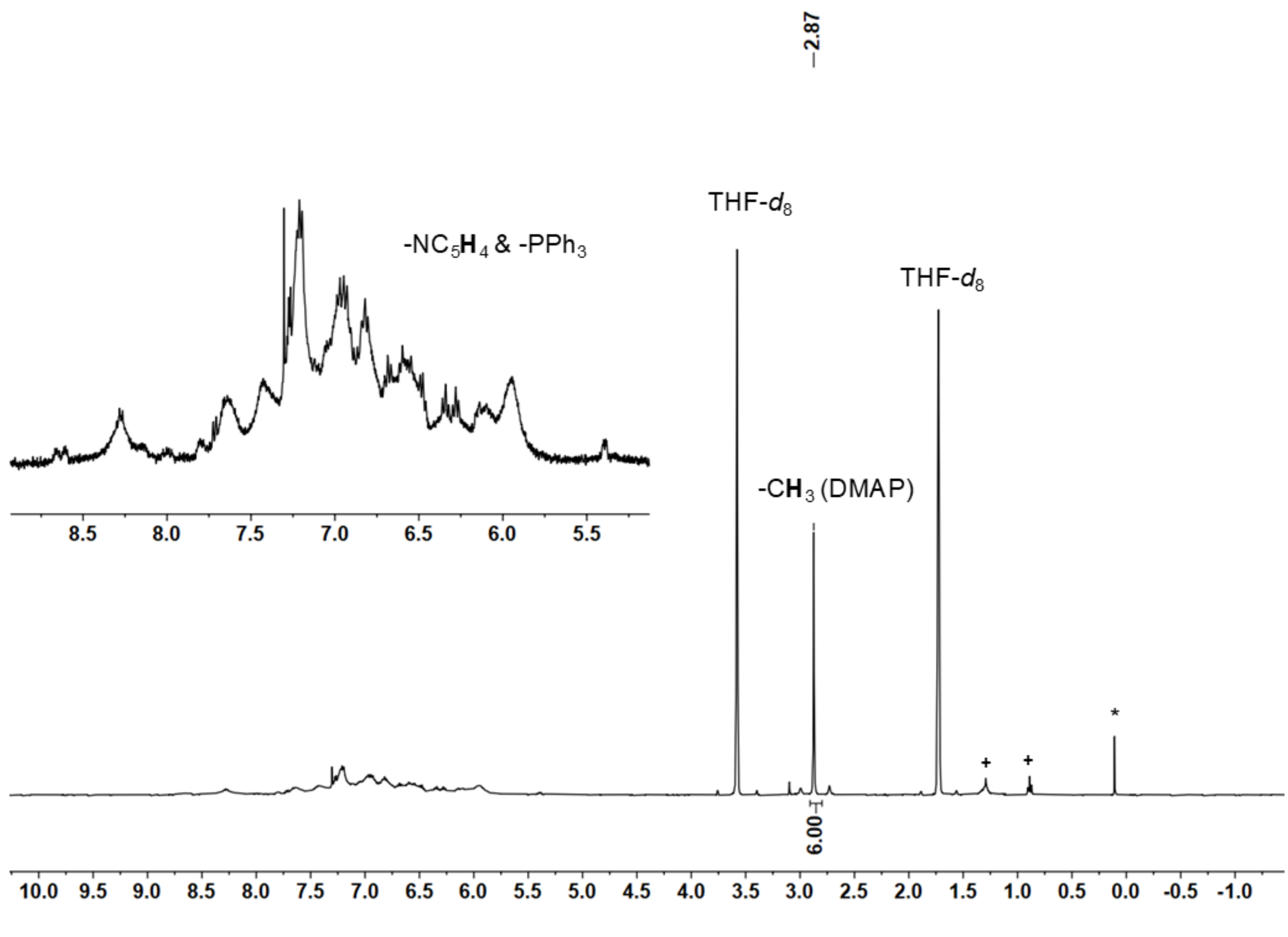

S-Figure 62: ^{1}H NMR spectrum (600.15 MHz, THF-d_8, 298 K) of [(TPB)Al(DMAP)] **8**. Silicon grease is marked with *, and residual pentane with +.

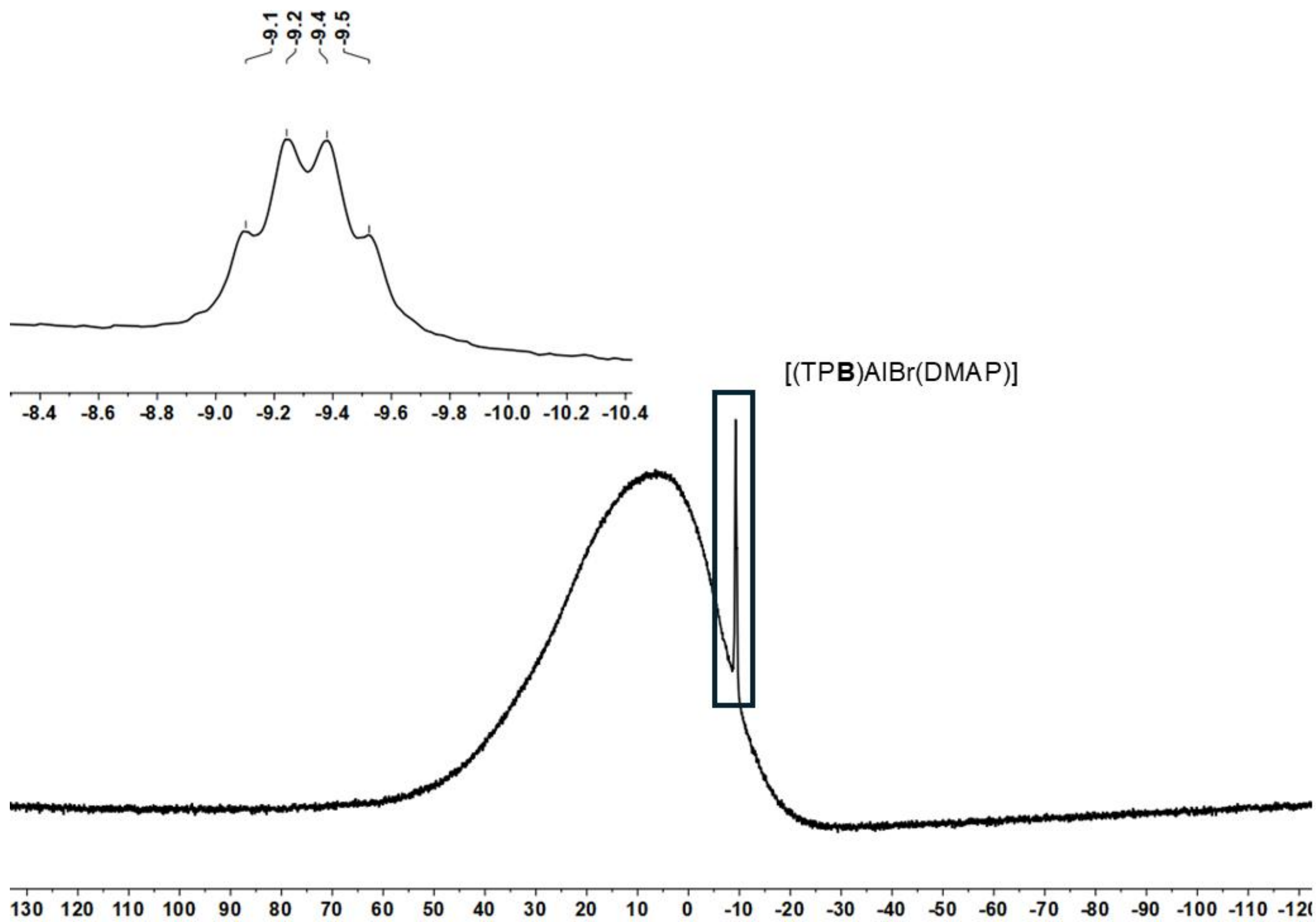

S-Figure 63: ^{11}B NMR spectrum (192.55 MHz, THF-d_8, 298 K) of [(TPB)AlBr(DMAP)] **8**.

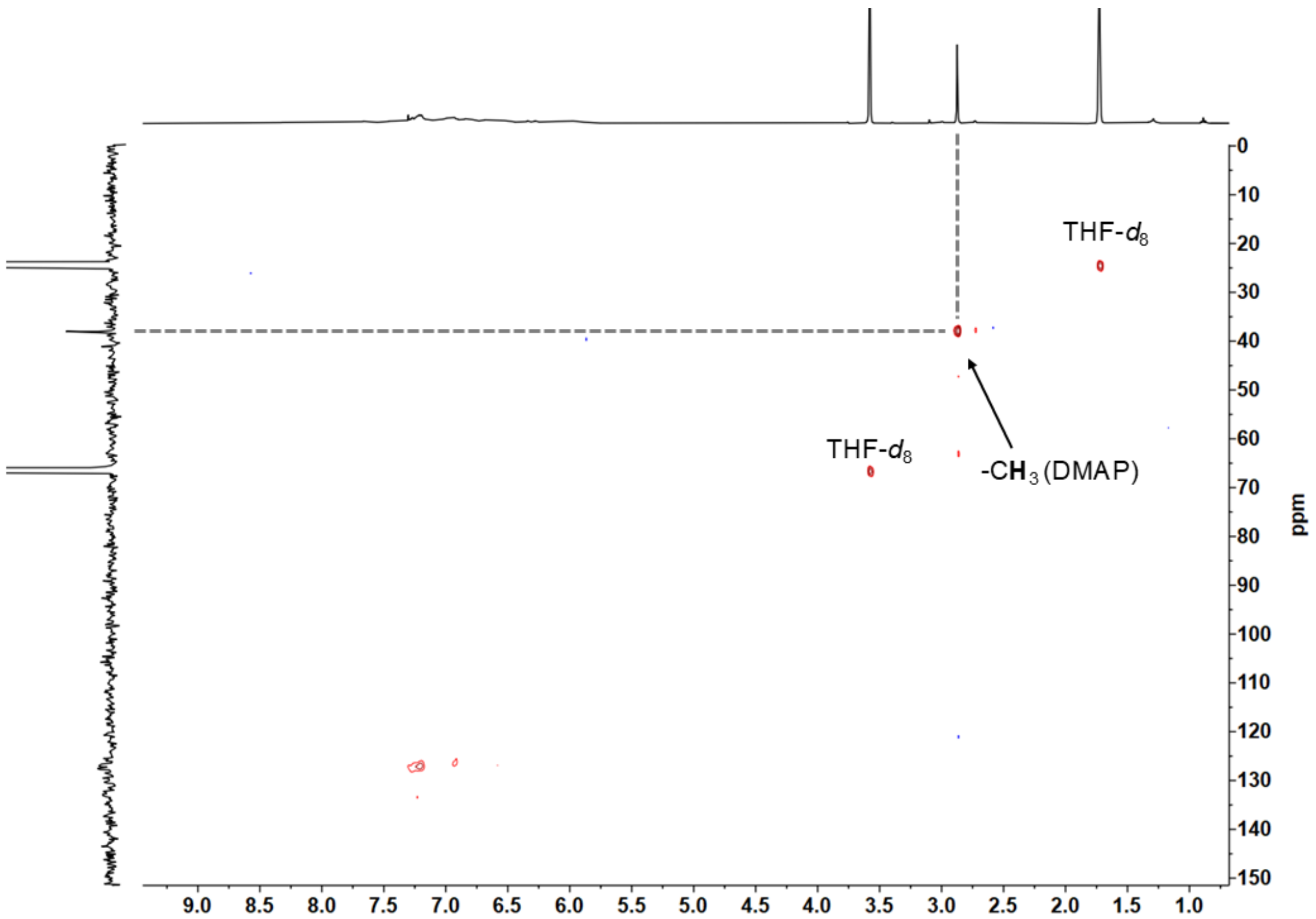

S-Figure 64: HSQC NMR spectrum (399.89/100.56 MHz, THF-d_8, 298 K) of [(TPB)AlBr(DMAP)] **8**.

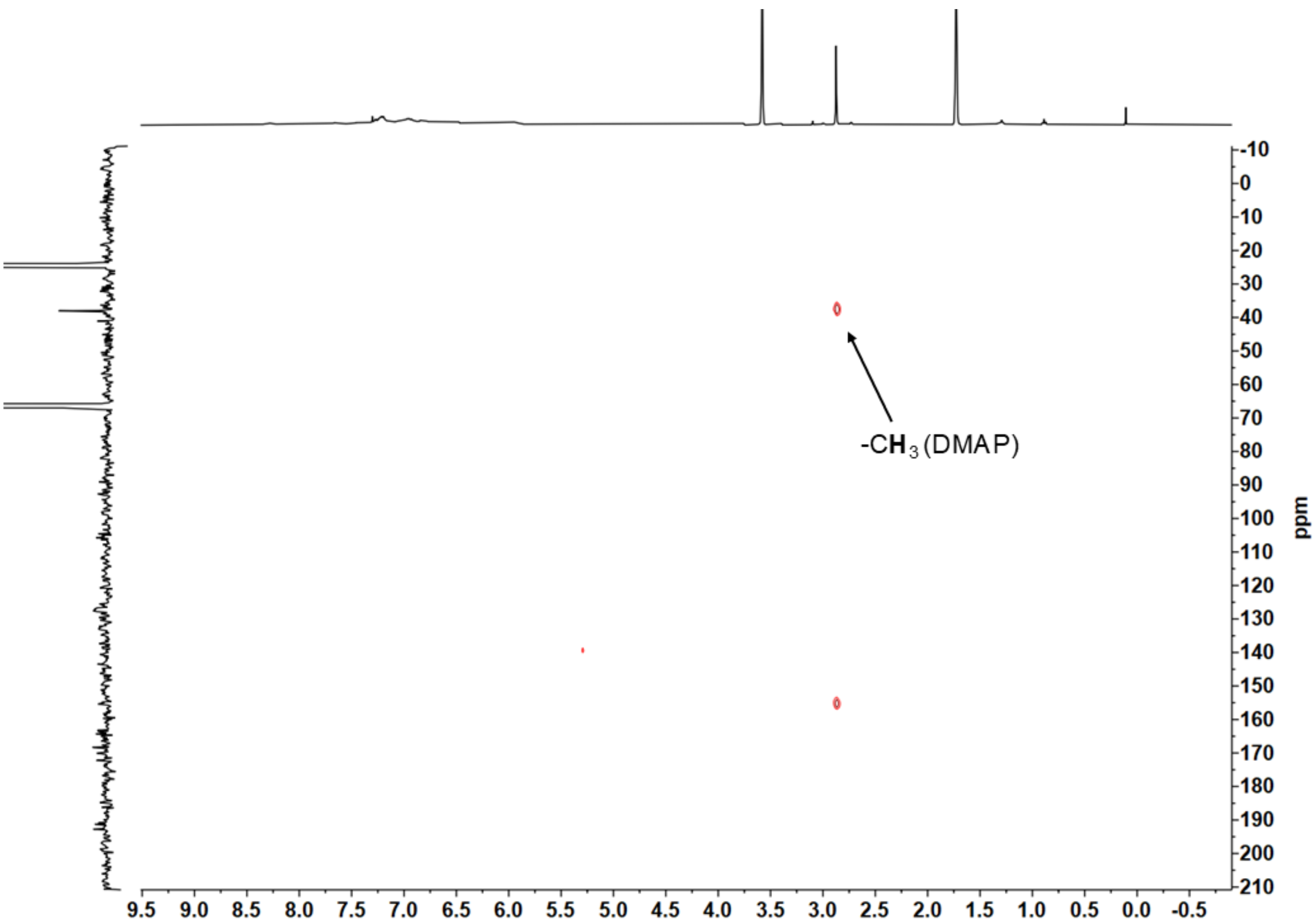

S-Figure 65: HMBC NMR spectrum (399.89/100.56 MHz, THF-d_8, 298 K) of [(TPB)AlBr(DMAP)] **8**.

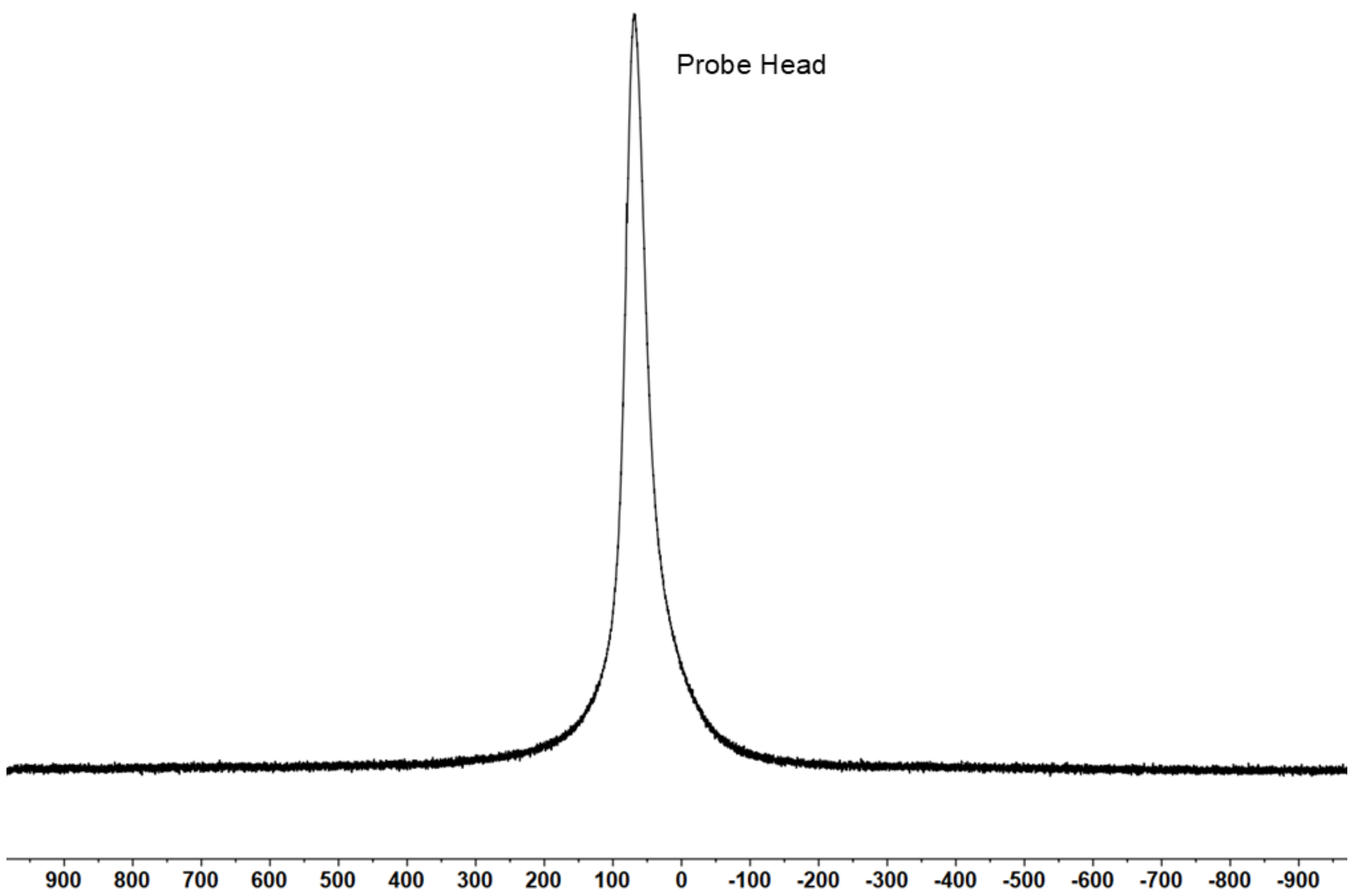

S-Figure 66: ^{27}Al NMR spectrum (104.20 MHz, THF-d_8, 298 K) of [(TPB)AlBr(DMAP)] **8**.

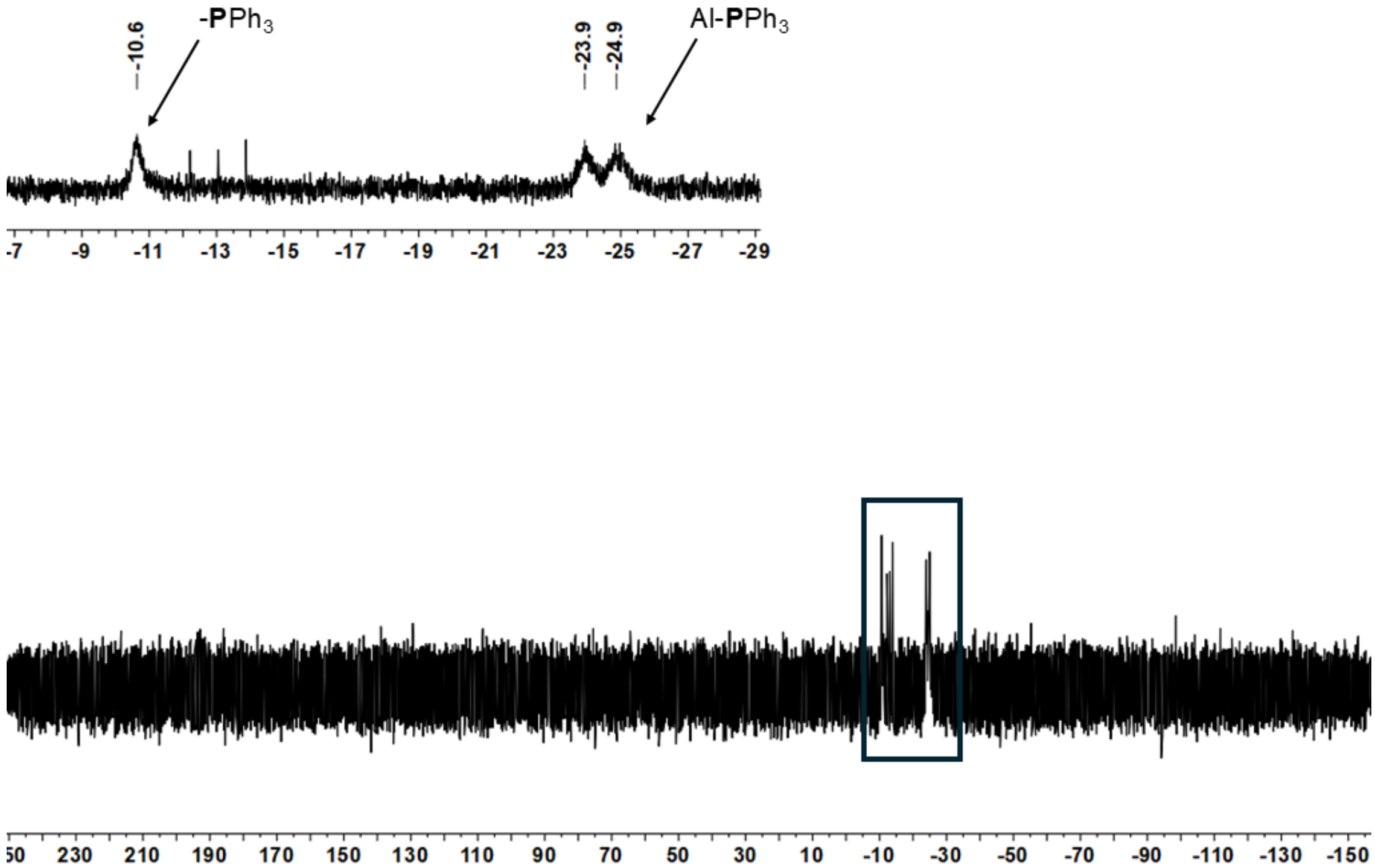

S-Figure 67: $^{31}P\{^{1}H\}$ NMR spectrum (242.96 MHz, THF-d_8, 298 K) of [(TPB)AlBr(DMAP)] **8**.

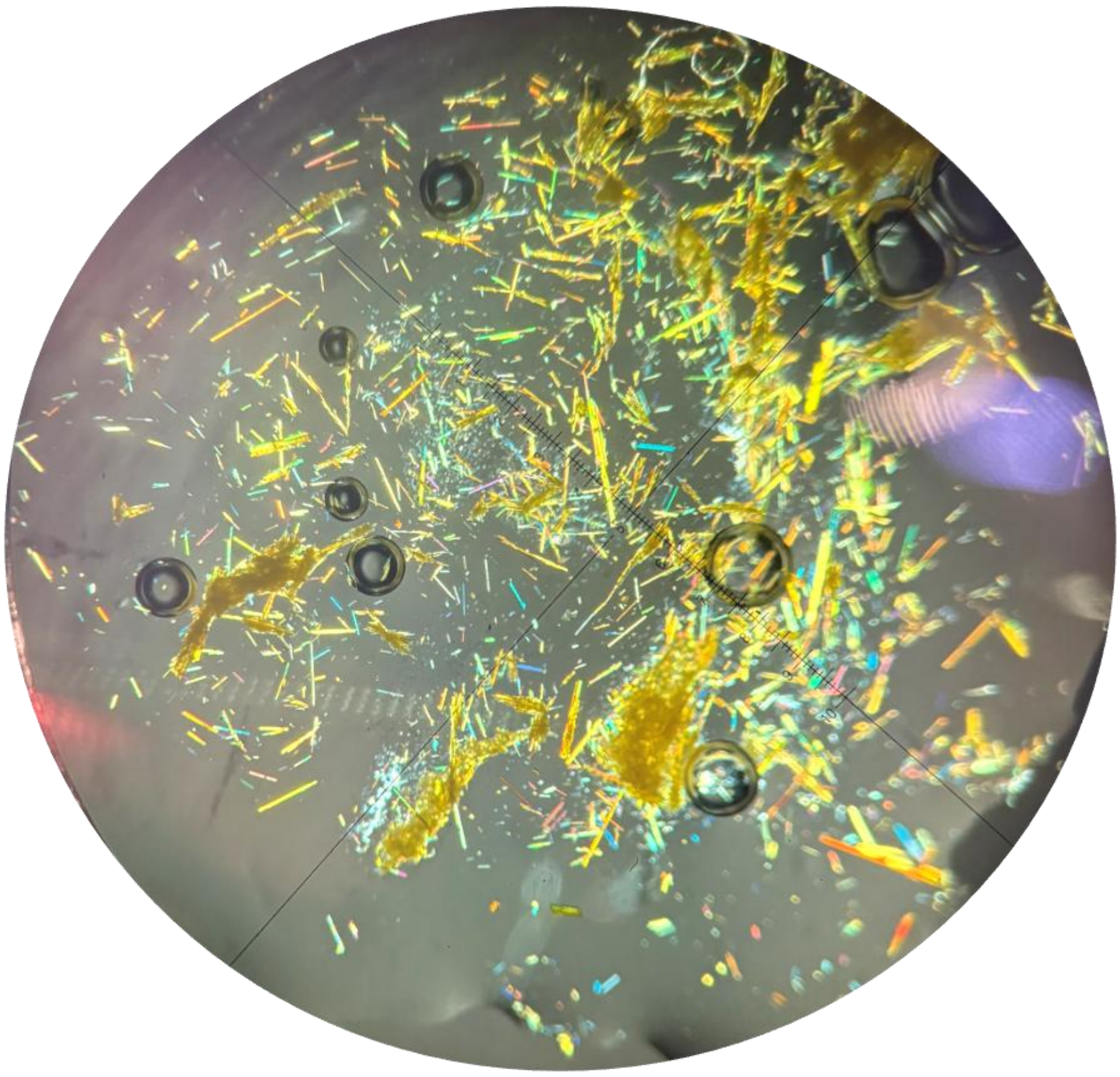

S-Figure 68: Picture of crystals of [(TPB)AlBr(DMAP)] **8** obtained from crystallization of the reaction mixture.

S-1.15 Preparation of [(TPB)AlBr(IMe$_4$)] 9

Procedure: [(TPB)AlBr] (30.0 mg, 33.3 µmol) was dissolved in 1.5 mL THF and IMe$_4$ (4.1 mg, 33 µmol, 1.0 equiv.) was added yielding a bright yellow solution. The solution was stirred for 10 min and then layered with 1.5 mL pentane. The yellow crystals were separated, washed with pentane, and dried in vacuo, affording [(TPB)AlBr(IMe$_4$)] as yellow powder (19.0 mg, 18.5 µmol, 56 %). scXRD-quality crystals were obtained by vapor diffusion of *n*-pentane onto a toluene solution.

^{1}H NMR spectrum (600.15MHz, THF-d_8, 298 K): δ = 1.62 (s, 6H, −CH$_3$), 2.59 (s, 6H, −CH$_3$), 6.88-7.96 (m) ppm.

^{11}B NMR spectrum (128.40 MHz, THF-d_8, 298 K): δ = −8.8 (q, $^{3}J_{B,P}$ = 25.7 Hz) ppm.

^{31}P{^{1}H} NMR spectrum (242.96 MHz, C_6D_6, 298 K): δ = −6.8 (s, −PPh$_3$), −27.8 (s, Al−PPh$_3$), −33.3 (s, Al−PPh$_3$) ppm.

Comment: Due to poor solubility of [(TPB)AlBr(IMe$_4$)] **9** in C_6D_6 and THF-d_8 the concentration of the NMR samples was too low to identify ^{13}C NMR signals and assign the signals of the aromatic region in the ^{1}H NMR spectrum. Signals in the ^{31}P NMR spectrum were detected with 640 scans.

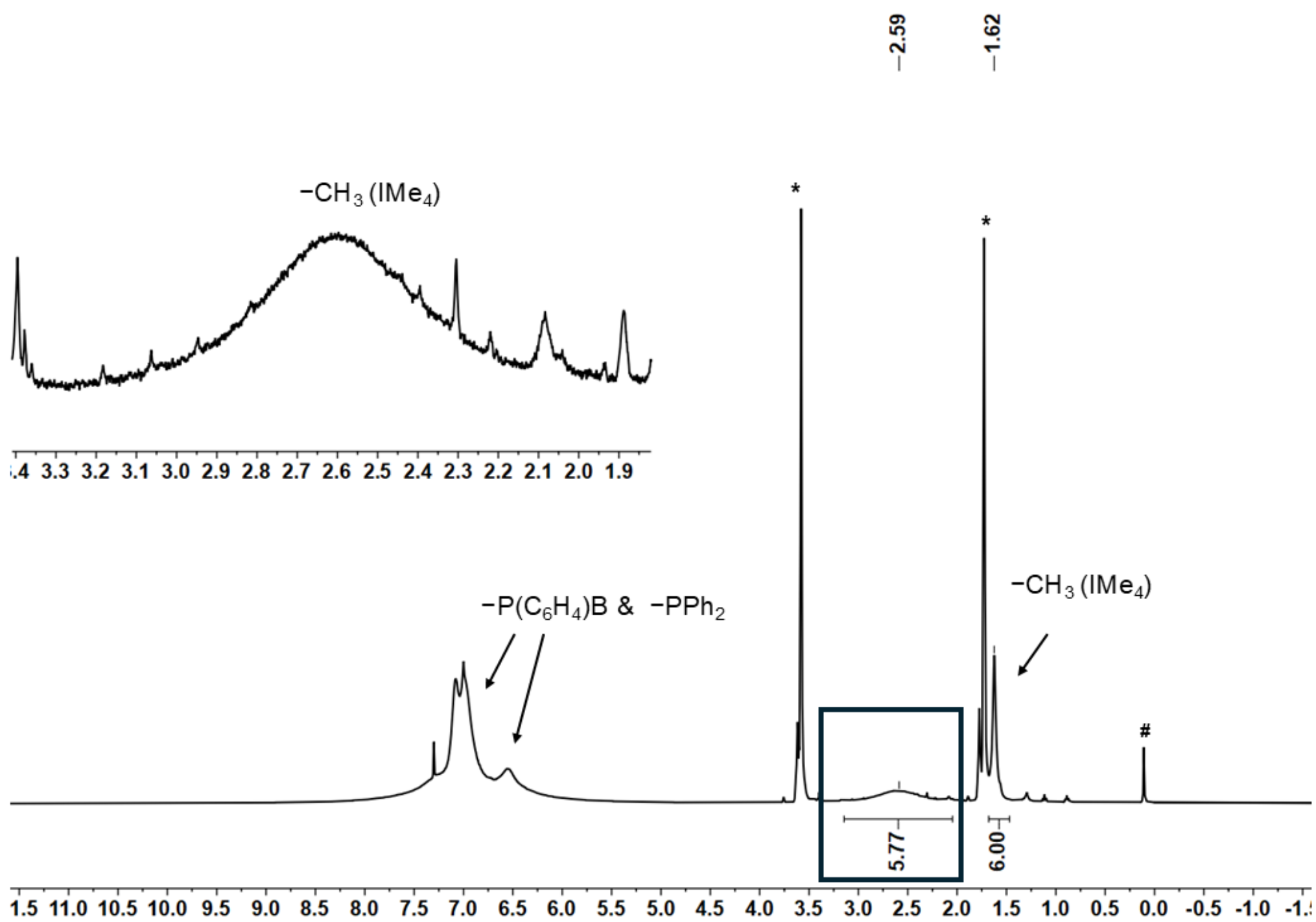

S-Figure 69: ^{1}H NMR spectrum (600.15 MHz, THF-d_8, 298 K) of [(TPB)AlBr(IMe$_4$)] **9**. THF-d_8 is marked with *, and silicon grease with #.

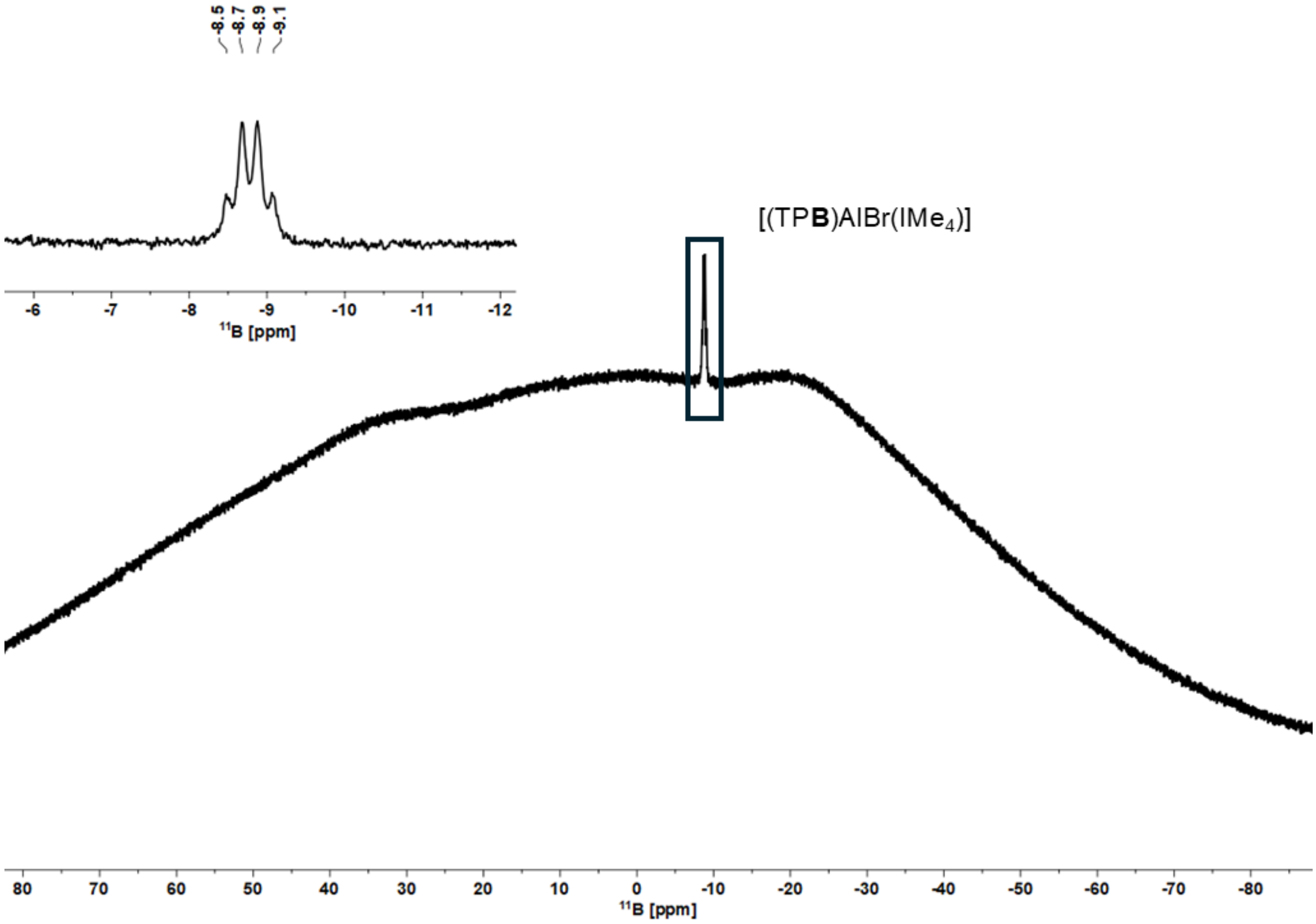

S-Figure 70: ^{11}B NMR spectrum (128.40 MHz, THF-d_8, 298 K) of [(TPB)AlBr(IMe₄)] **9**.

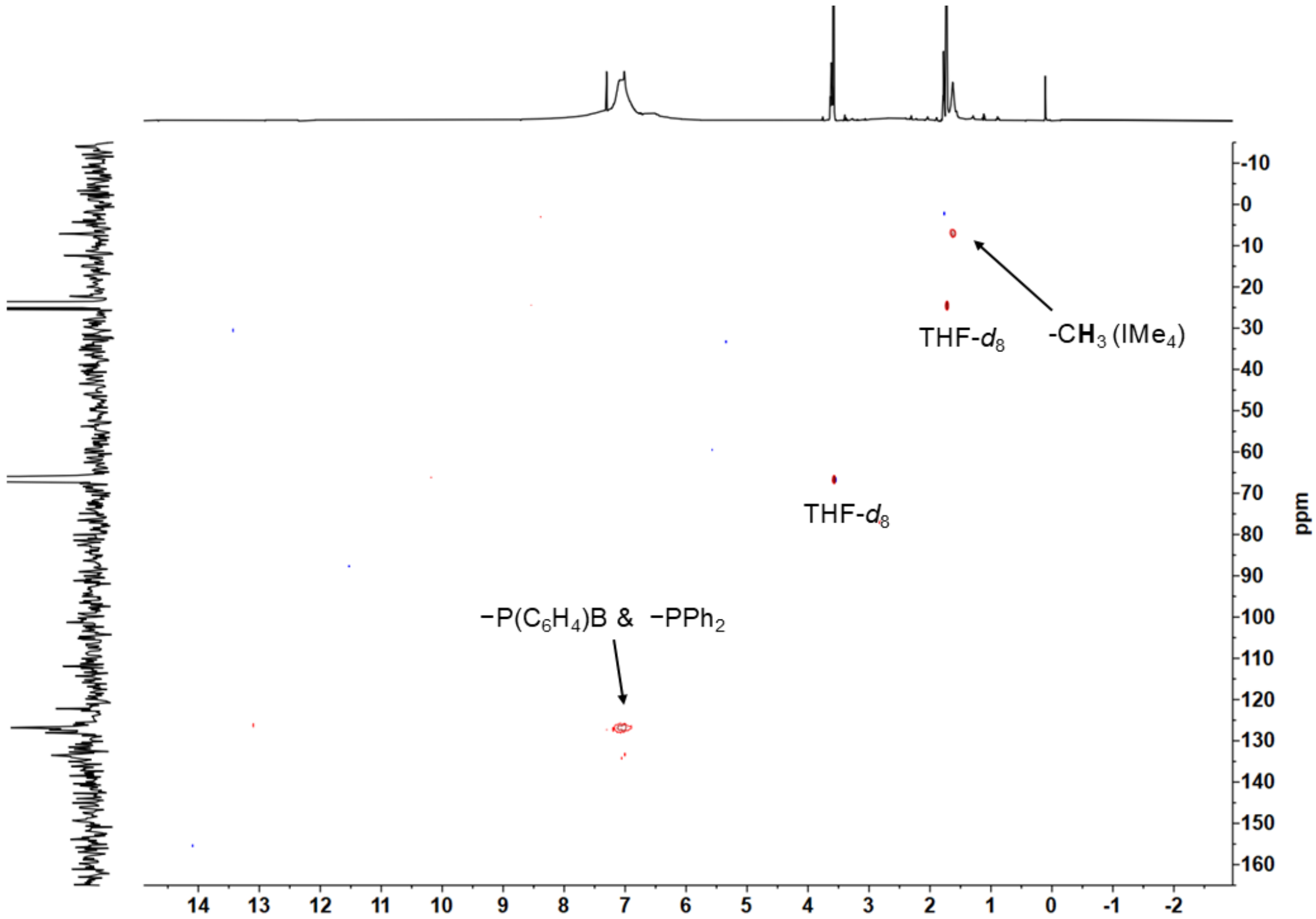

S-Figure 71: HSQC NMR spectrum (399.89/100.56 MHz, THF-d_8, 298 K) of [(TPB)AlBr(IMe$_4$)] **9**.

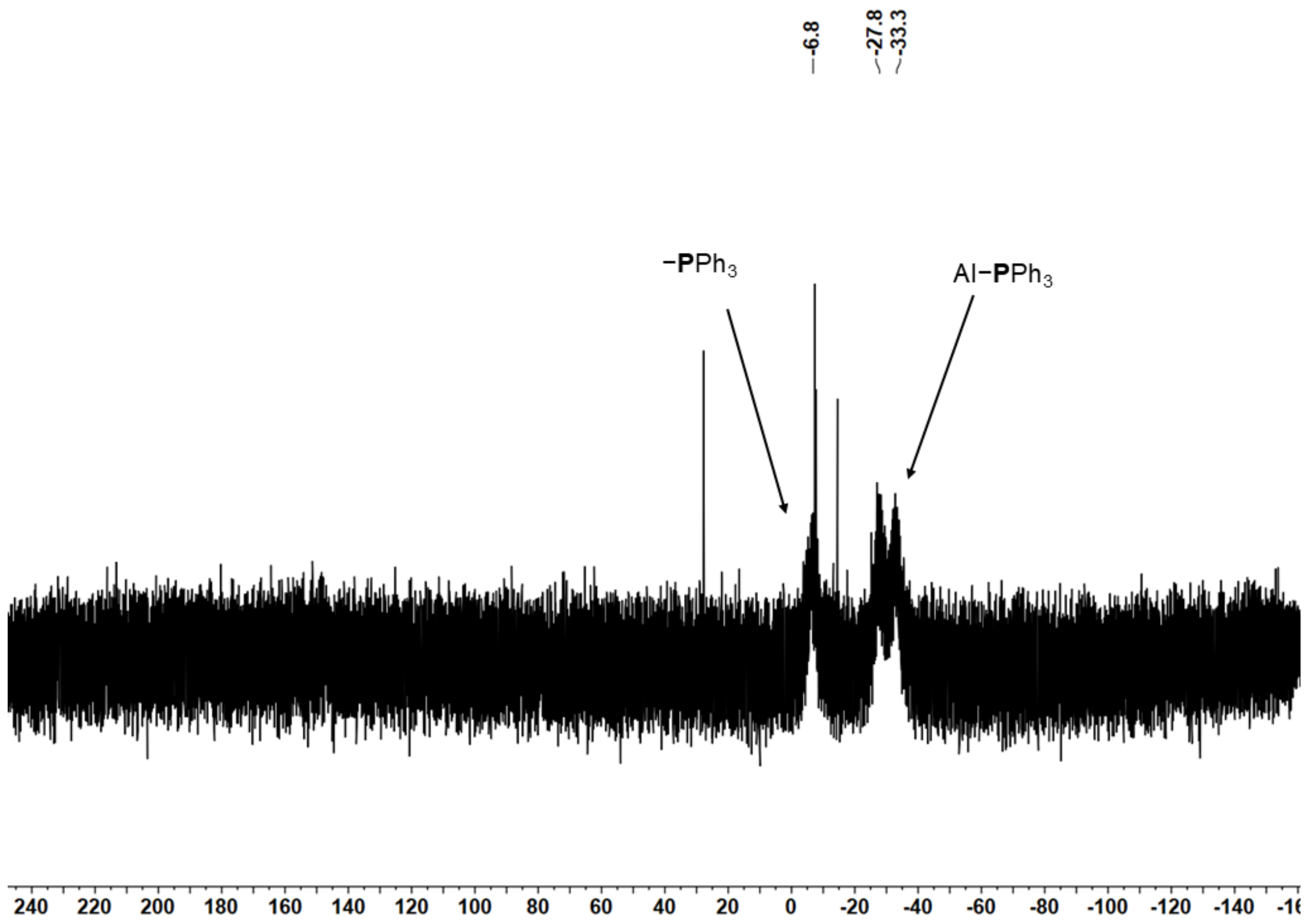

S-Figure 72: ^{31}P NMR spectrum (242.96 MHz, C_6D_6, 298 K) of [(TPB)AlBr(IMe$_4$)] **9** with 640 scans.

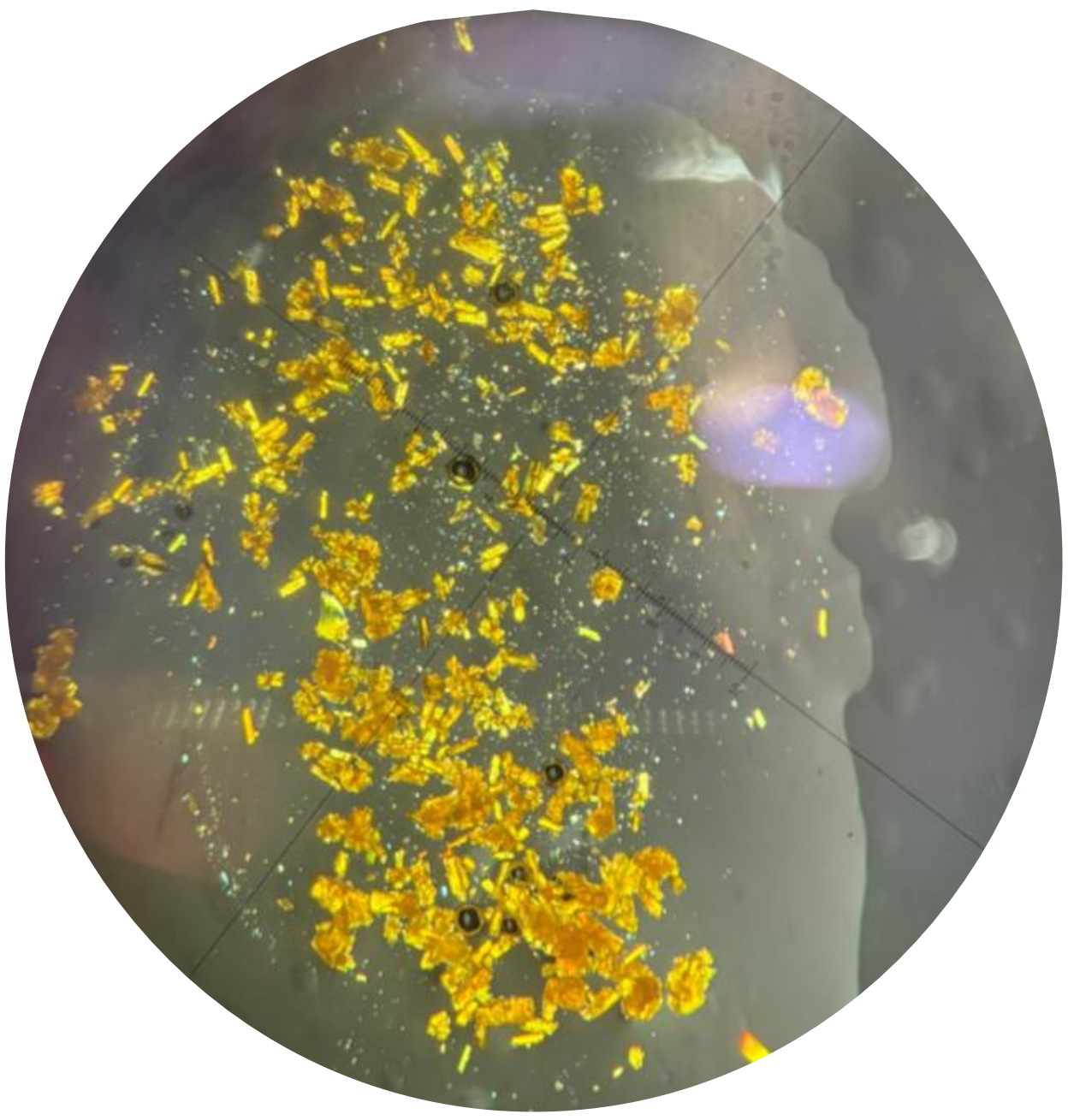

S-Figure 73: Picture of crystals of [(TPB)AlBr(IMe$_4$)] **9** obtained from crystallization of the reaction mixture.

S-1.16 Preparation of $[(TPB)Al(DMAP)]^+$ 10

Procedure: [(TPB)AlBr(DMAP)] (6.0 mg, 5.9 µmol) was dissolved in 0.6 mL C_6D_6 and charged into an NMR tube. $Na[B(C_6F_5)_4]$ (4.1 mg, 5.9 µmol, 1.0 equiv.) was added yielding a red solution. scXRD-quality crystals were obtained by vapor diffusion of *n*-pentane onto the C_6D_6 solution (6.4 mg, 3.9 µmol, 67%).

^{1}H NMR spectrum (600.15 MHz, C_6D_6, 298 K): δ = 1.86 (s, 6H, $-CH_3$), 5.86 (d, 2H, J = 6.9 Hz, $-NC_5H_4$), 6.73-6.85 (m, 20H $-PPh_2$/$-P(C_6H_4)B$), 6.96-7.02 (m, 12H, $-PPh_2$), 7.05-7.10 (m, 3H, $-PPh_2$), 7.11-7.13 (m, 4H, $-P(C_6H_4)B$), 7.52-7.53 (m, 3H, $-P(C_6H_4)B$), 8.34 (d, 2H, J = 6.8 Hz, $-NC_5H_4$) ppm.

^{13}C NMR spectrum (150.92 MHz, C_6D_6, 298 K): δ = 37,4 ($-CH_3$), 106.7 ($-NC_5H_4$), 124.5 ($-P(C_6H_4)B$), 128.5 ($-PPh_2$), 129.8 ($-P(C_6H_4)B$), 132.8 ($-PPh_2$), 135.6 ($-NC_5H_4$) ppm.

^{11}B NMR spectrum (192.55 MHz, C_6D_6, 298 K): δ = −7.6 (q, $^3J_{B,P}$ = 42.8 Hz) ppm.

^{31}P{^{1}H} NMR spectrum (242.96 MHz, C_6D_6, 298 K): δ = −23.4 (fq, $^3J_{B,P}$ = 35.6 Hz) ppm.

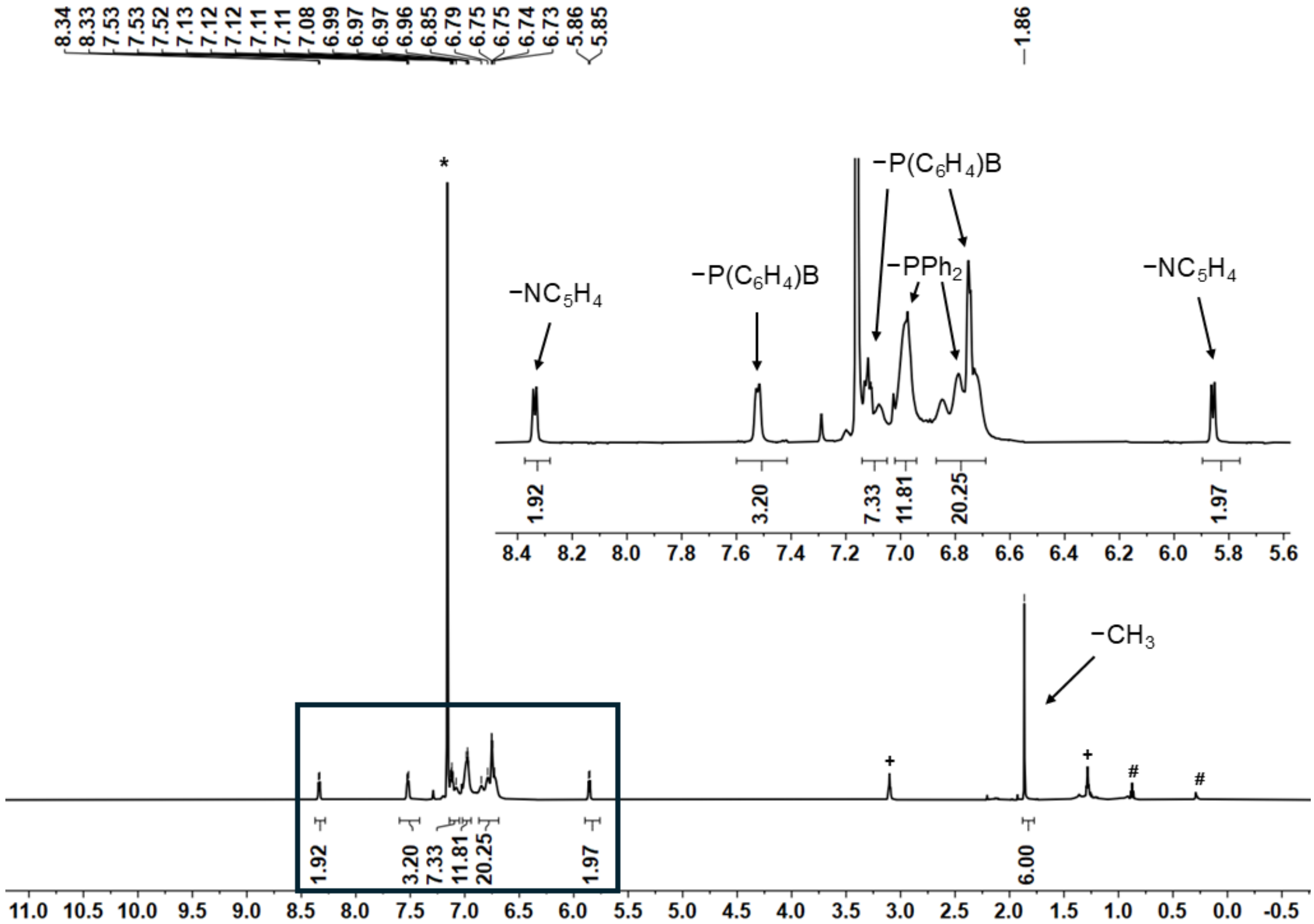

S-Figure 74: ^{1}H NMR spectrum (600.15 MHz, C_6D_6, 298 K) of $[(TPB)Al(DMAP)]^+$ **10**. C_6D_6 is marked with *, THF with +, and silicon grease with #

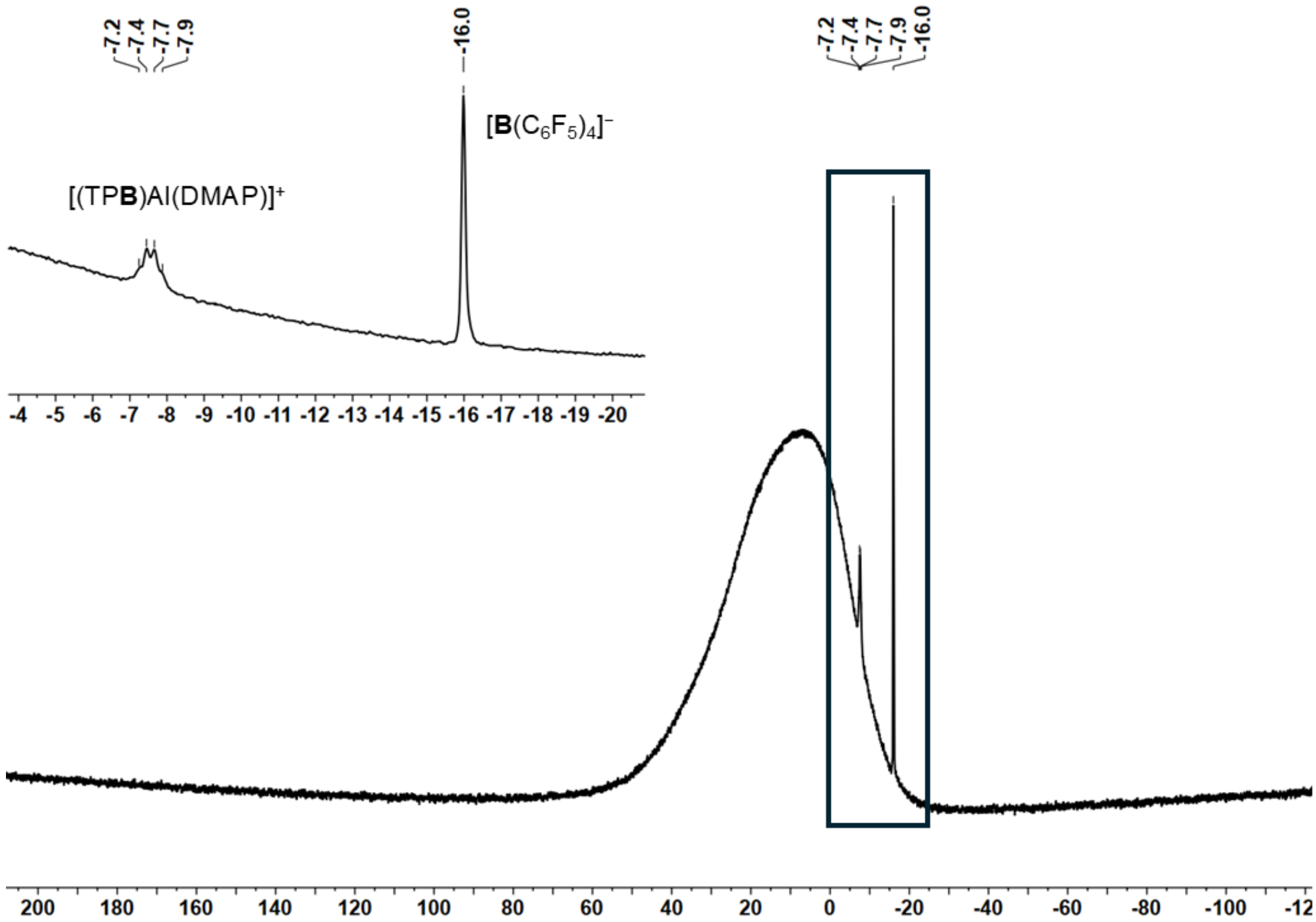

S-Figure 75: ^{11}B NMR spectrum (192.55 MHz, C_6D_6, 298 K) of $[(TPB)Al(DMAP)]^+$ **10**.

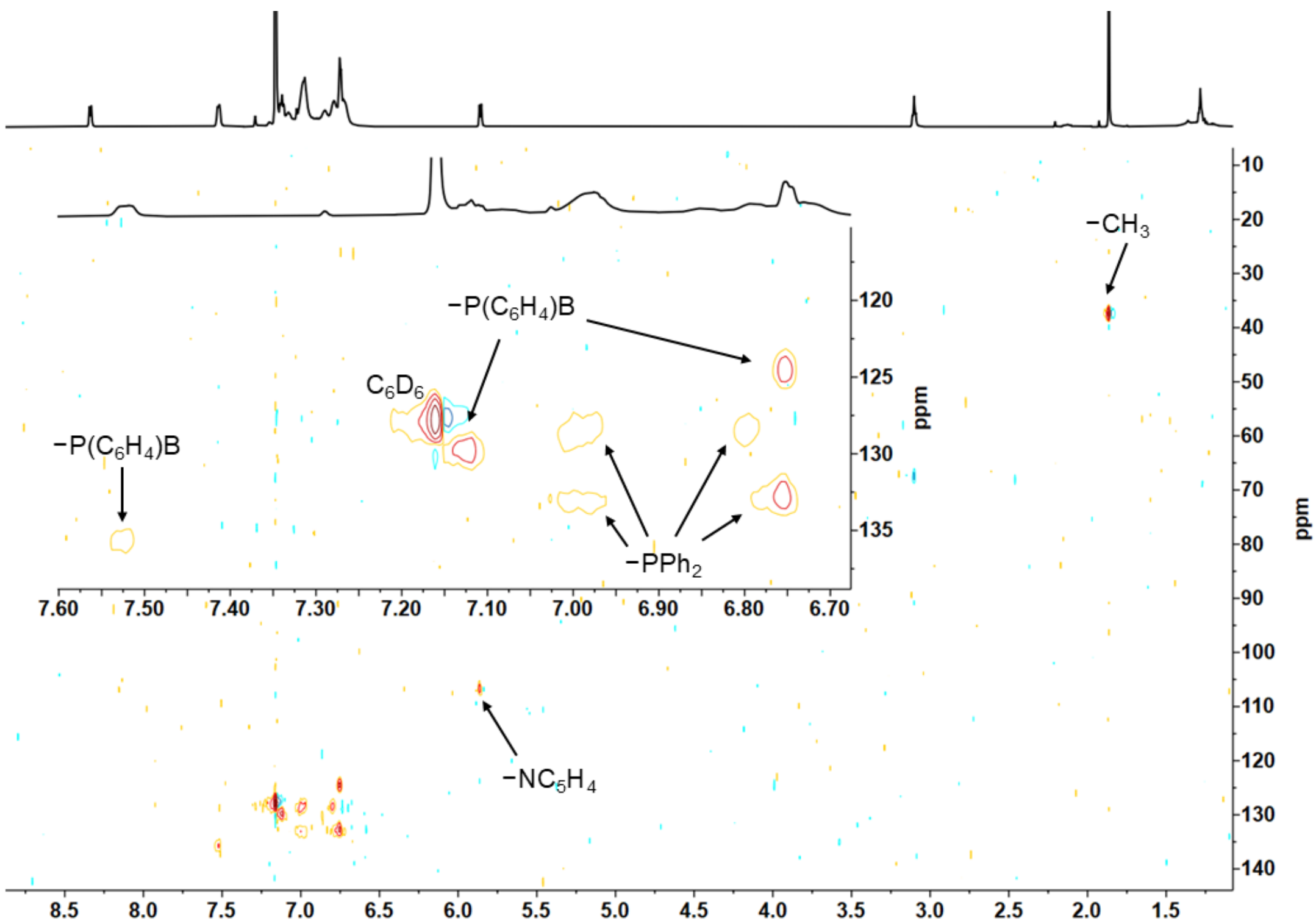

S-Figure 76: HSQC NMR spectrum (600.15/150.92 MHz, C_6D_6, 298 K) of $[(TPB)Al(DMAP)]^+$ **10**.

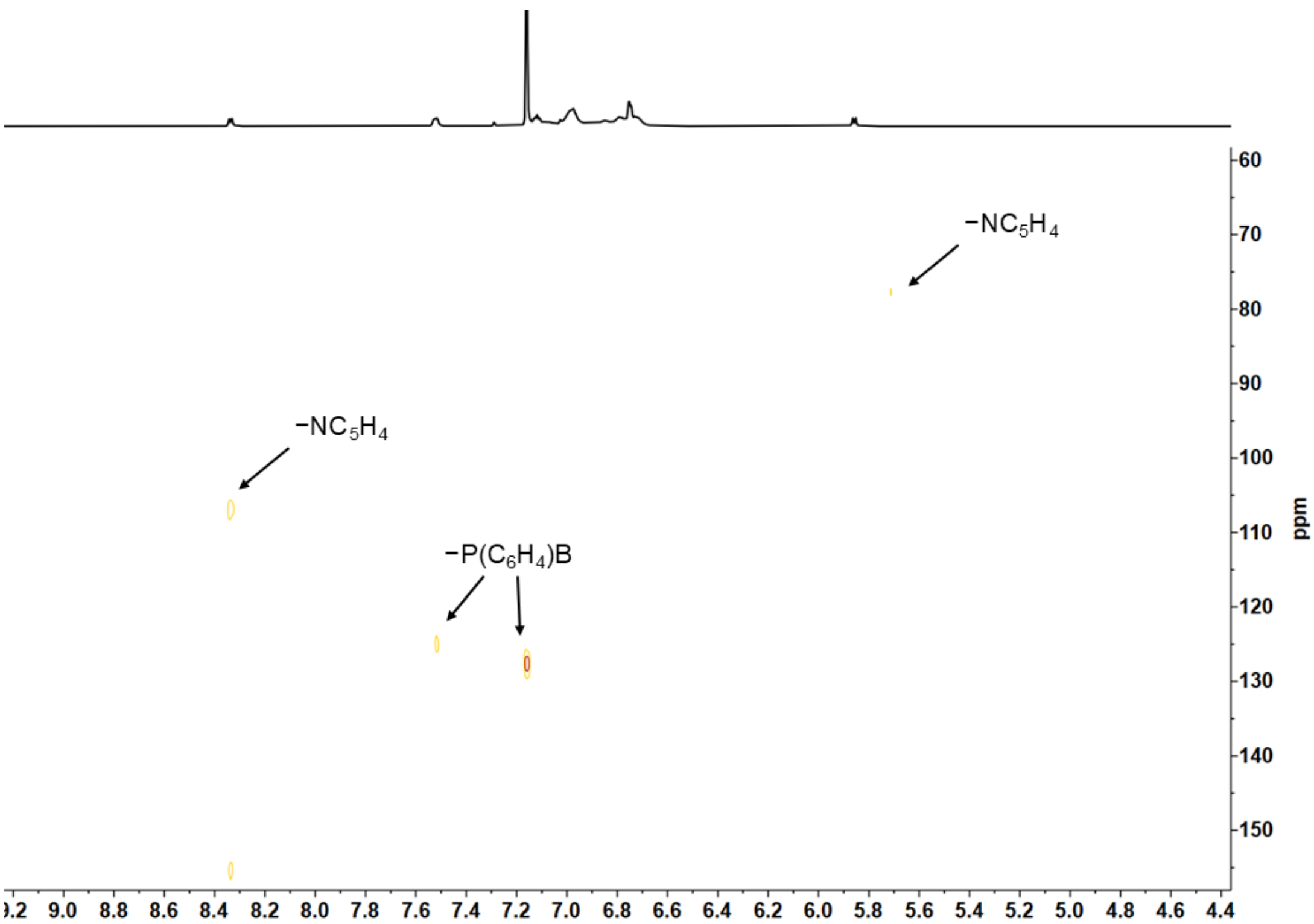

S-Figure 77: HMBC NMR spectrum (600.15/150.92 MHz, C_6D_6, 298 K) of $[(TPB)Al(DMAP)]^+$ **10**.

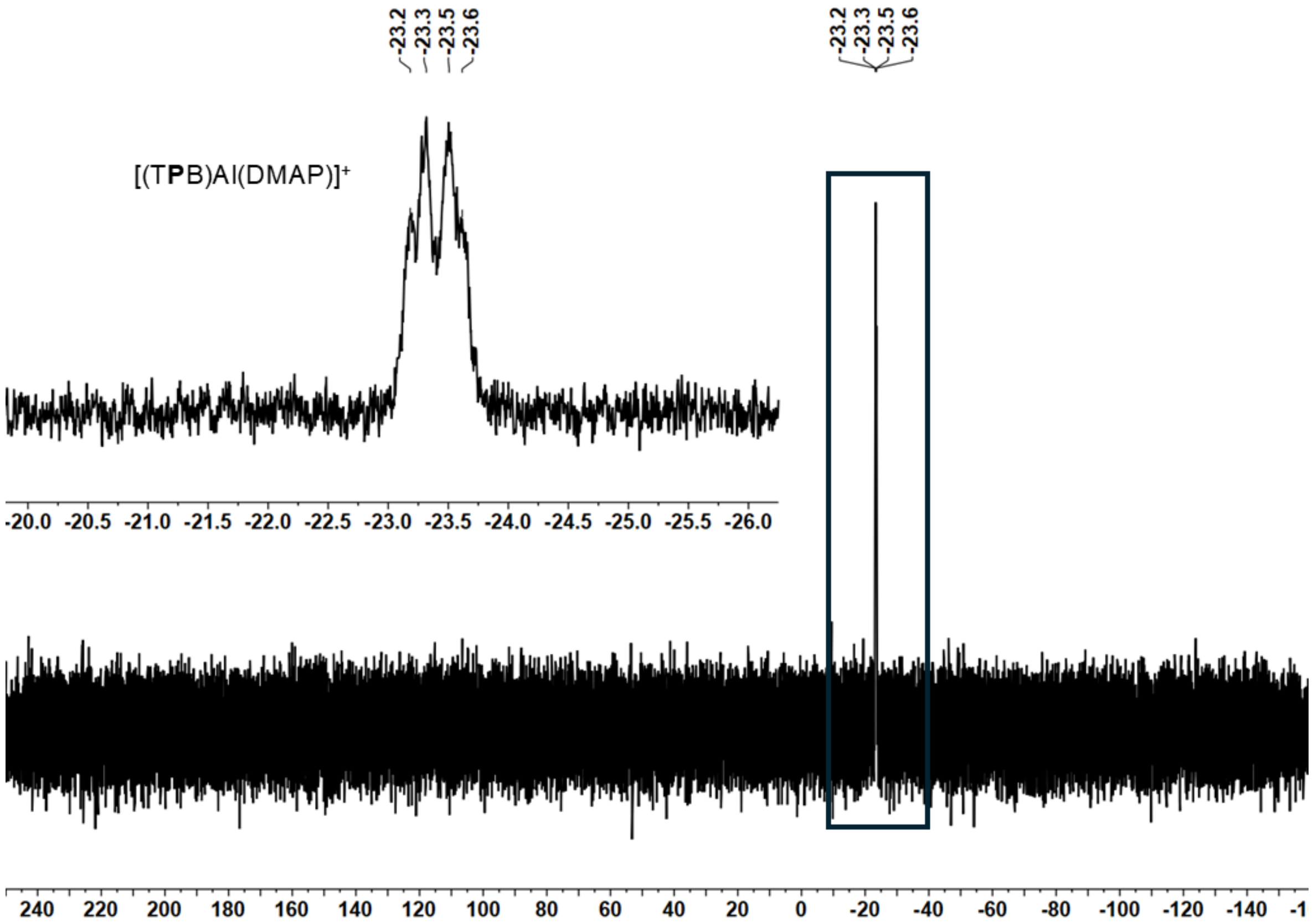

S-Figure 78: ^{31}P NMR spectrum (242.96 MHz, C_6D_6, 298 K) of $[(TPB)Al(DMAP)]^+$ **10**.

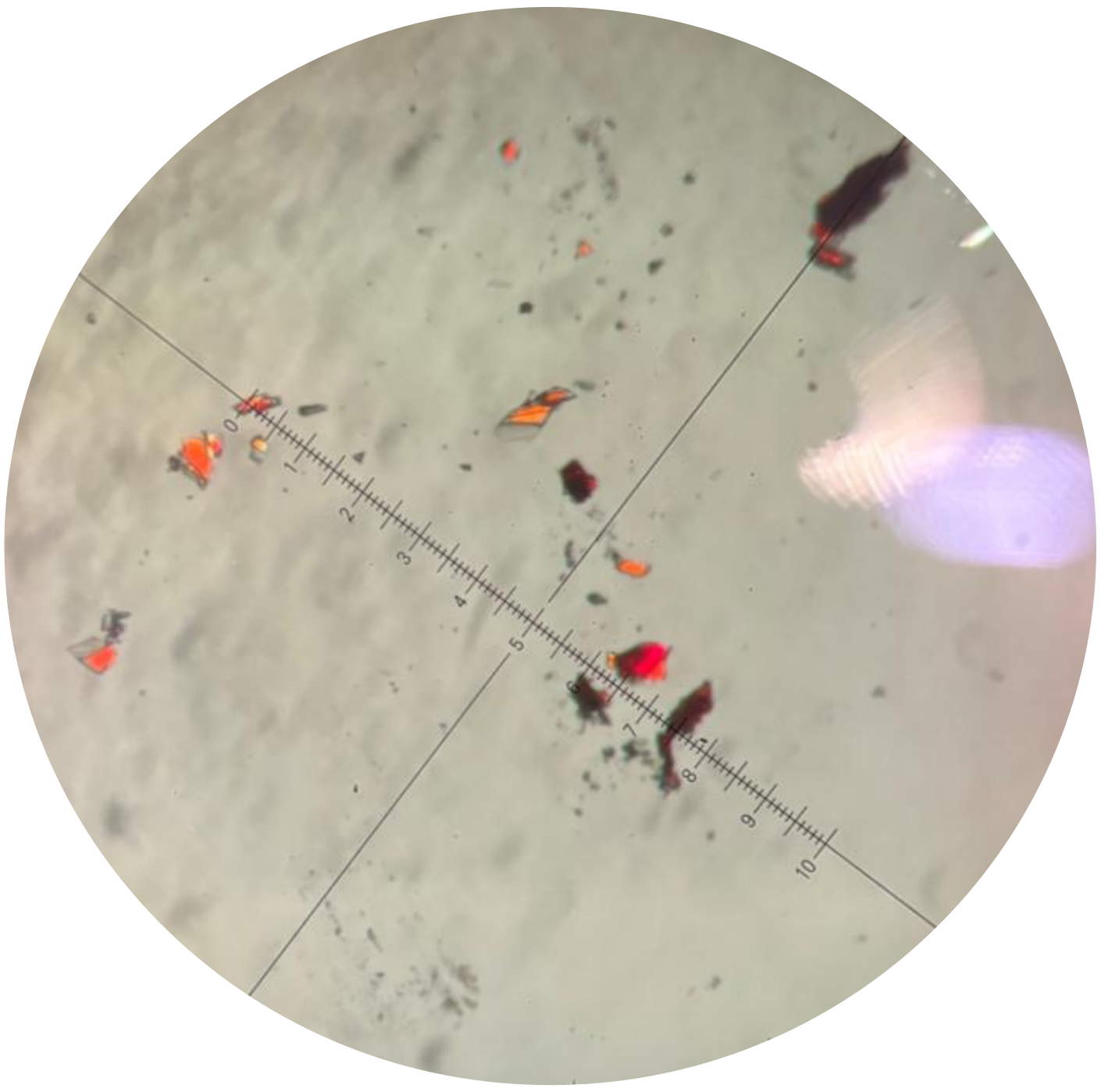

S-Figure 79: Picture of crystals of [(TPB)Al(DMAP)]$^+$ **10** obtained from crystallization of the reaction mixture.

S-1.17 NMR reaction: Preparation of $[(TPB)Al(IMe_4)]^+[B(C_6F_5)_4]^-$ 11 via $[(TPB)AlBr(IMe_4)]$ 9

Procedure: [(TPB)AlBr] (15.0 mg, 16.6 µmol) was dissolved in 0.6 mL toluene. A solution of IMe_4 (2.1 mg, 17 µmol, 1.0 equiv.) in 0.2 mL toluene was added. The bright yellow solution was investigated by NMR spectroscopy confirming the formation of $[(TPB)AlBr(IMe_4)]$ **9**. Then, $Na[B(C_6F_5)_4]$ (11.7 mg, 16.6 µmol, 1.00 equiv.) was added to the NMR tube and the mixture was investigated by NMR spectroscopy. The solvent was evaporated, yielding a yellow oil. Crystals were obtained by vapor diffusion of *n*-pentane onto a solution of Et_2O.

Analysis: The ^{11}B NMR spectrum shows a new signal at −6.4 ppm, and the ^{31}P NMR spectrum a new signal at −12.3 ppm which were assigned to the cation $[(TPB)Al(IMe_4)]^+[B(C_6F_5)_4]^-$ **11**. The structure was confirmed by X-ray crystallography.

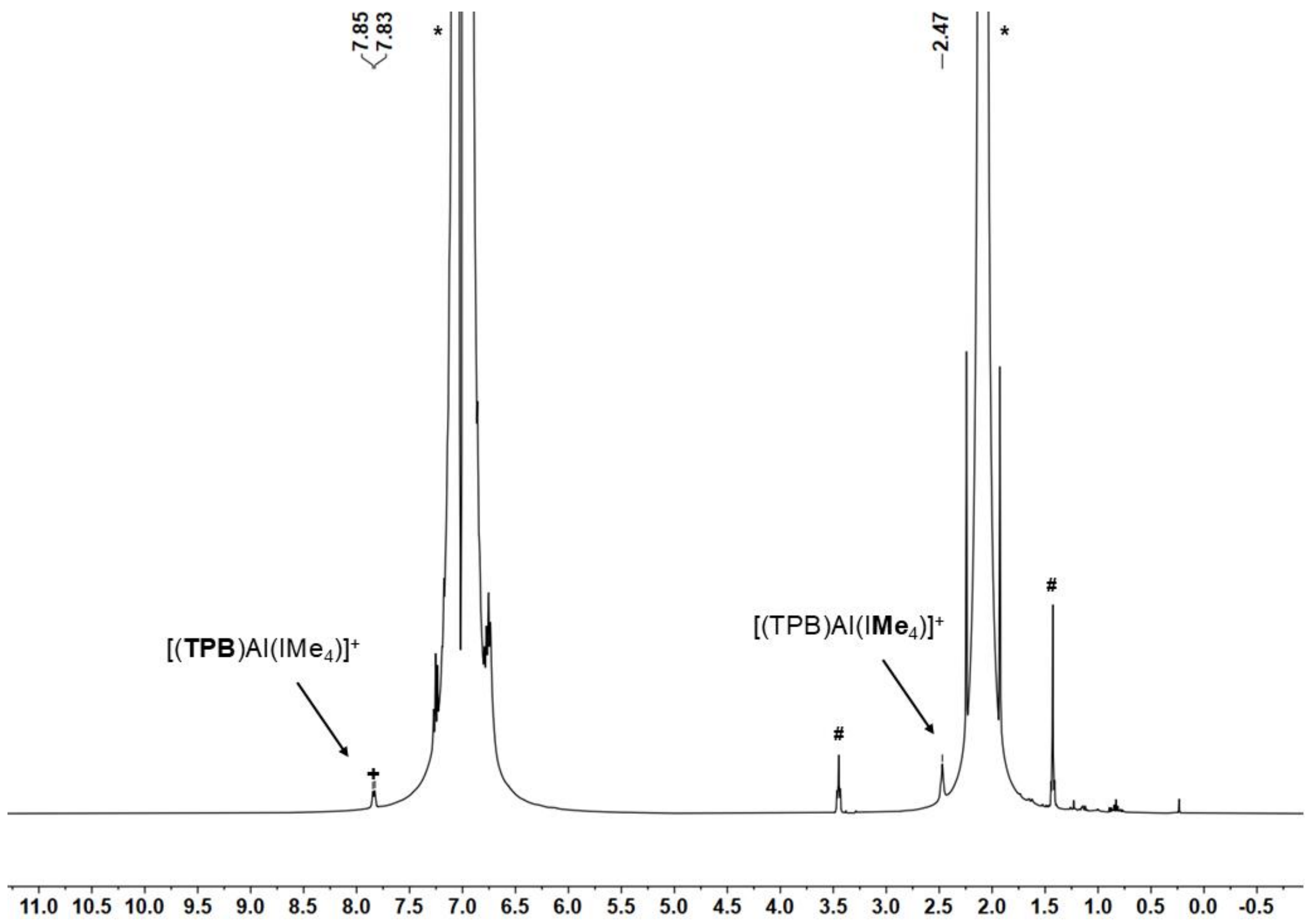

S-Figure 80: ^1H NMR spectrum (400.20 MHz, toluene, 298 K) of the NMR reaction mixture of [(TPB)AlBr], IMe_4 and $Na[B(C_6F_5)_4]$.

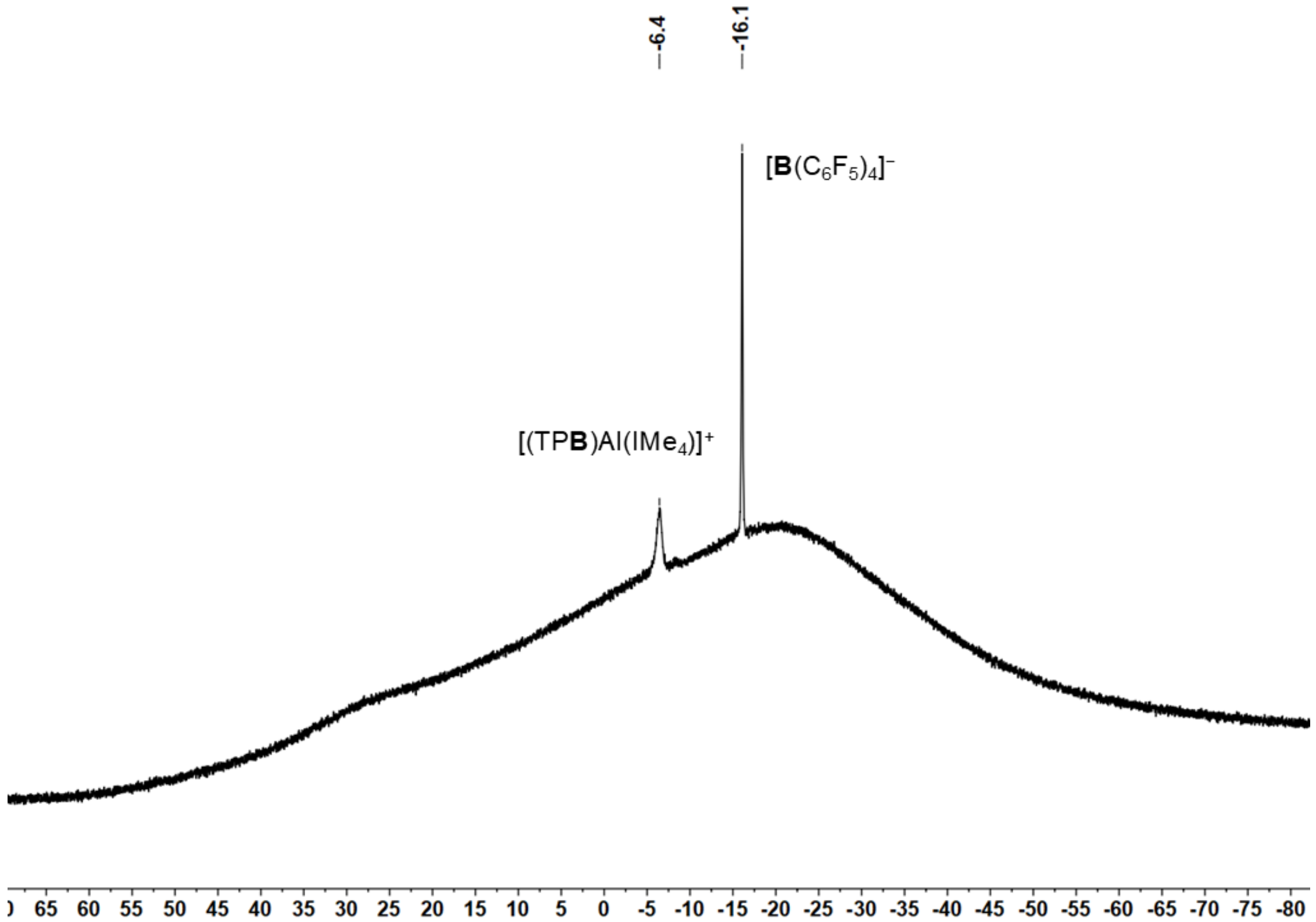

S-Figure 81: ^{11}B NMR spectrum (128.40 MHz, toluene, 298 K) of the NMR reaction mixture of [(TPB)AlBr], IMe_4 and $Na[B(C_6F_5)_4]$.

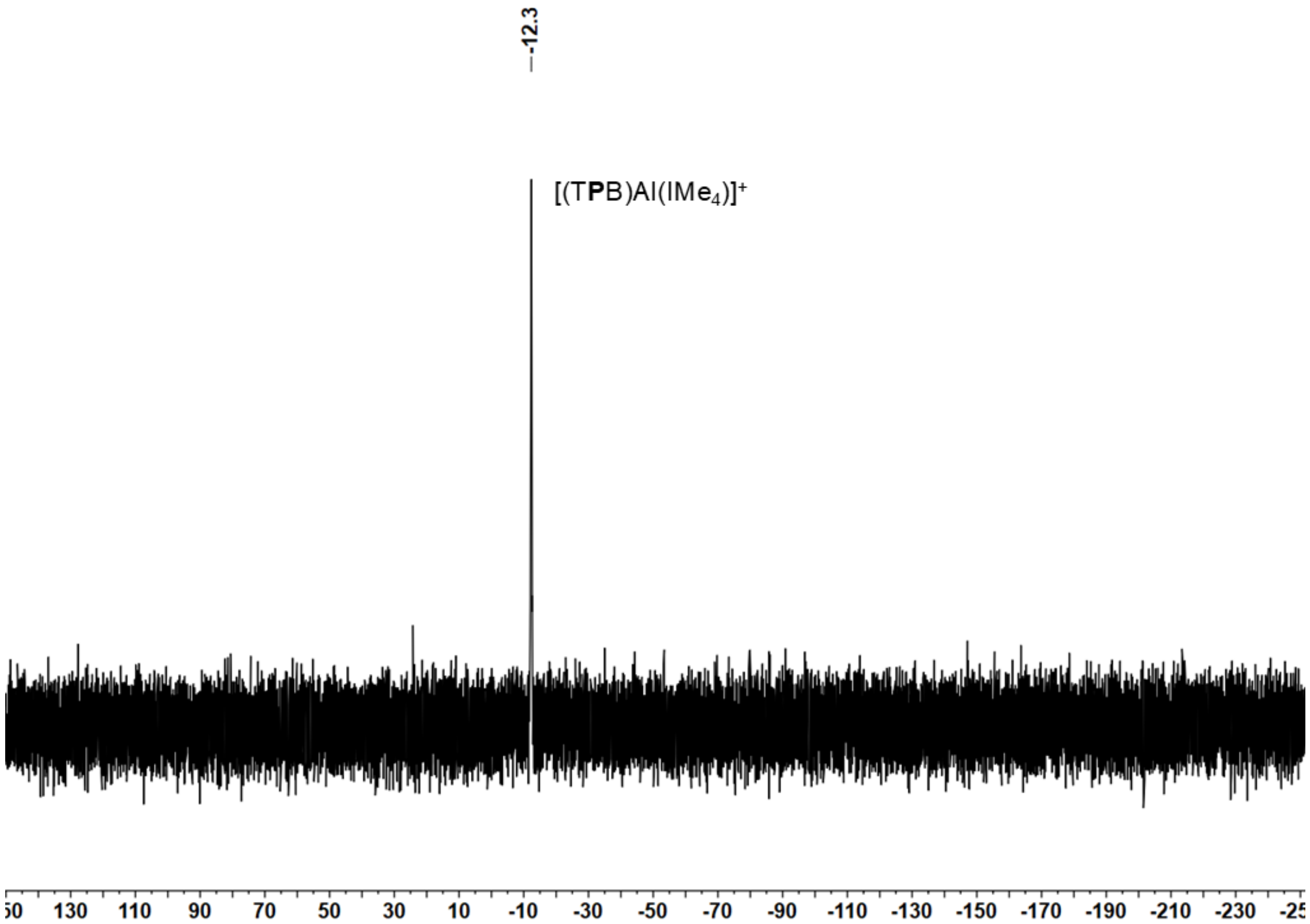

S-Figure 82: $^{31}P\{^{1}H\}$ NMR spectrum (162.00 MHz, toluene, 298 K) of the NMR reaction mixture of [(TPB)AlBr], IMe_4 and $Na[B(C_6F_5)_4]$.

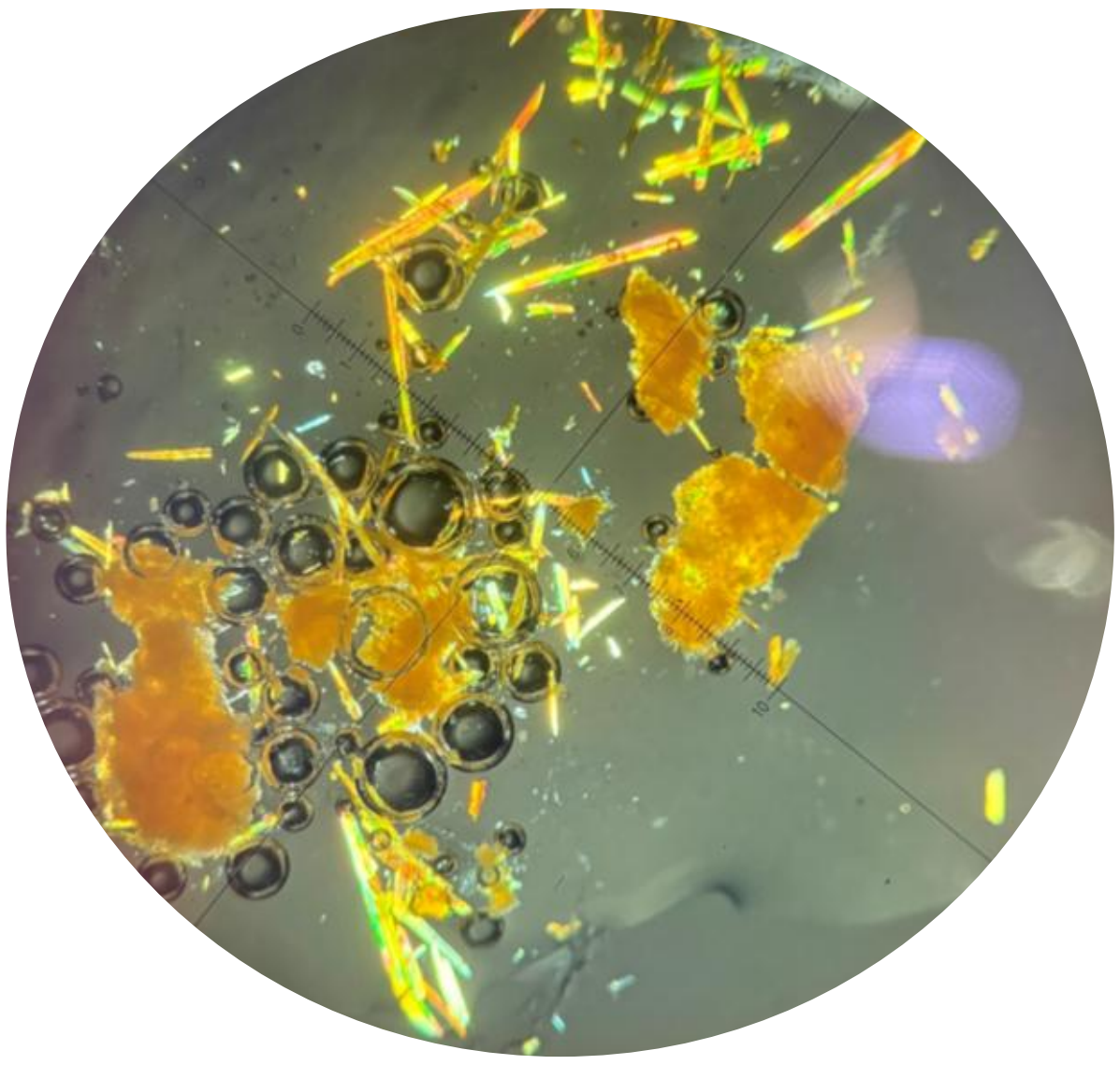

S-Figure 83: Picture of crystals of $[(TPB)Al(IMe_4)]^+$ **11** obtained from crystallization of the reaction mixture.

S-1.18 Preparation of $[(TPB)Al(IMe_4)]^+$ 11

Procedure: $[(TPB)AlBr(IMe_4)]$ **9** (7.0 mg, 6.8 µmol) and $Na[B(C_6F_5)_4]$ (4.8 mg, 6.8 µmol, 1.0 equiv.) were dissolved in C_6H_6 and stirred for 5 min. The mixture was filtered and the solvent of the filtrate was removed in vacuo affording $[(TPB)Al(IMe_4)]^+$ **11** as yellow solid (8.1 mg, 5.0 µmol, 73 %).

^{1}H NMR spectrum (600.15 MHz, C_6D_6, 298 K): δ = 1.39 (s, 6H, $-CH_3$), 2.47 (s, 6H, $-CH_3$), 6.76-6.79 (m, 25H, $-P(C_6H_4)B$ & $-PPh_2$), 6.90-6.95 (m, 6H, $-PPh_2$), 7.00-7.01 (m, 4H, $-PPh_2$), 7.07-7.09 (m, 4H, $-P(C_6H_4)B$), 7.92-9.93 (m, 3H, $-P(C_6H_4)B$) ppm.

^{13}C NMR spectrum (150.92 MHz, C_6D_6, 298 K): δ = 125.1 ($-P(C_6H_4)B$), 130.0 ($-PPh_2$), 132.2 ($-P(C_6H_4)B$), 133.6 ($-PPh_2$ & $-P(C_6H_4)B$) ppm.

^{11}B NMR spectrum (192.55 MHz, C_6D_6, 298 K): δ = −6.4 (s, $[(TP\mathbf{B})Al(IMe_4)]^+$), −15.9 (s,

$[\mathbf{B}(C_6F_5)_4]^-$) ppm.

^{31}P NMR spectrum (242.96, C_6D_6, 298 K): δ = −12.2 (s, $[(T\mathbf{P}B)Al(IMe_4)]^+$) ppm.

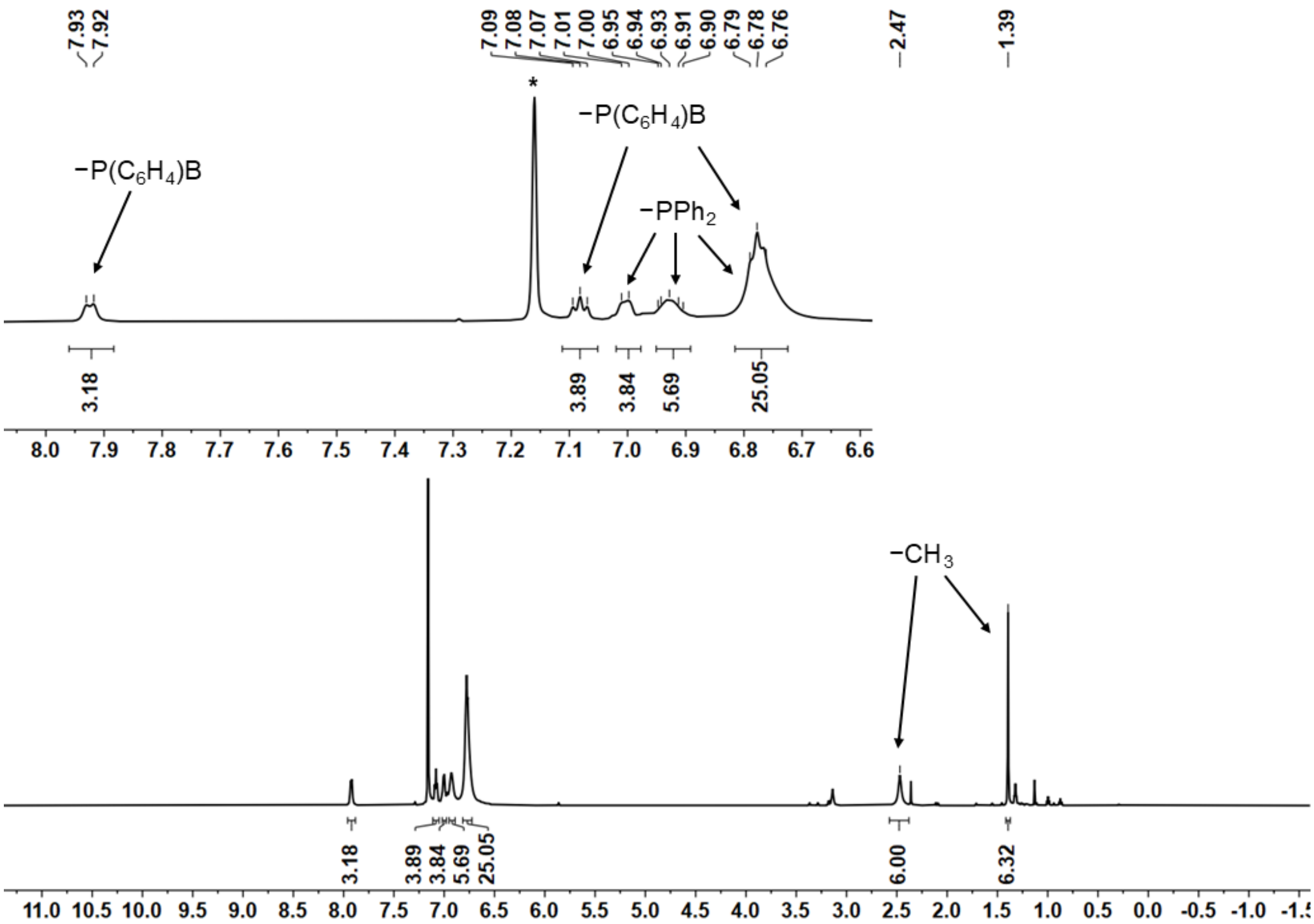

S-Figure 84: ^{1}H NMR spectrum (600.15 MHz, C_6D_6, 298 K) of $[(TPB)Al(IMe_4)]^+$ **11**. C_6D_6 is marked with *.

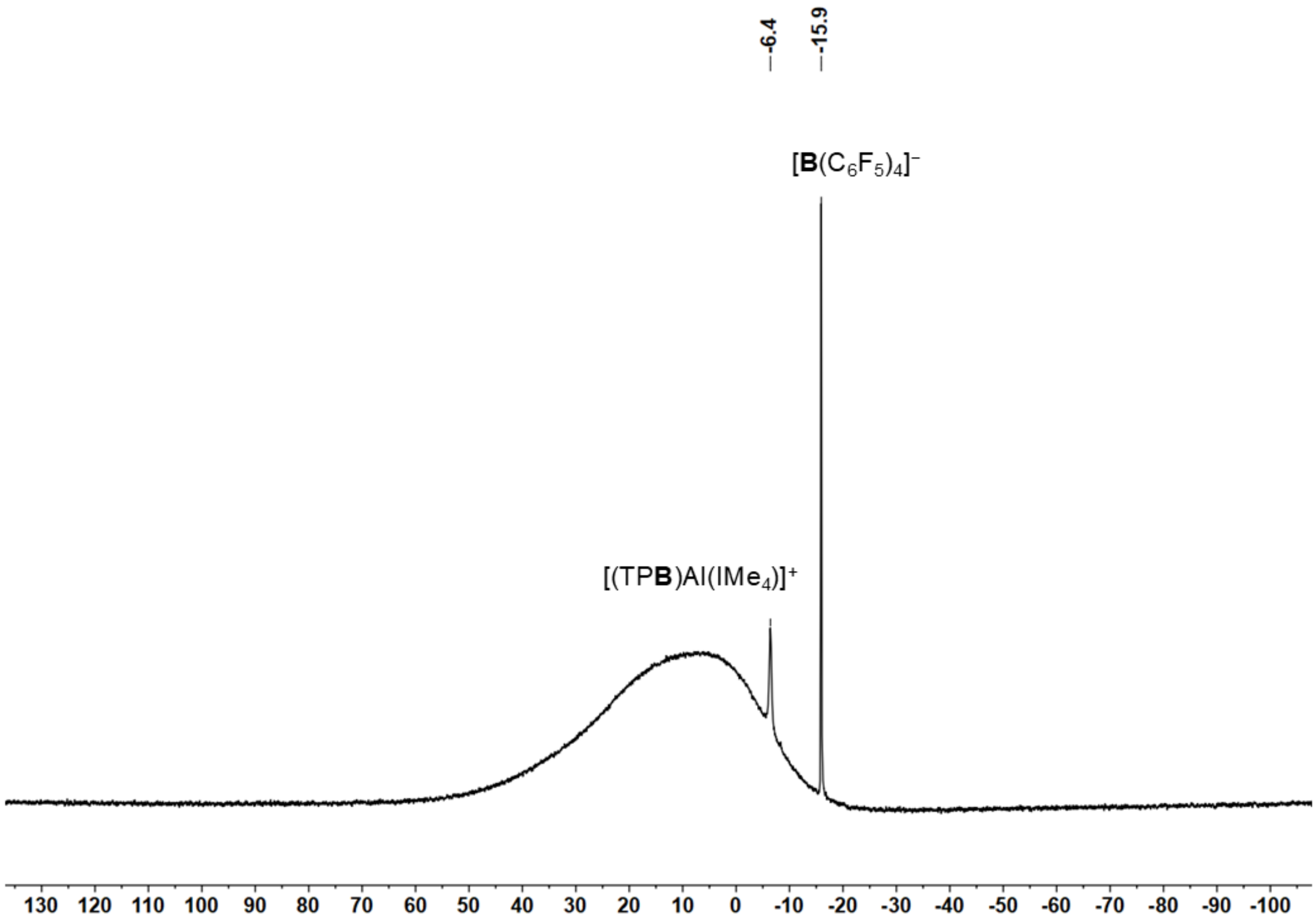

S-Figure 85: ^{11}B NMR spectrum (192.55 MHz, C_6D_6, 298 K) of $[(TPB)Al(IMe_4)]^+$ **11**.

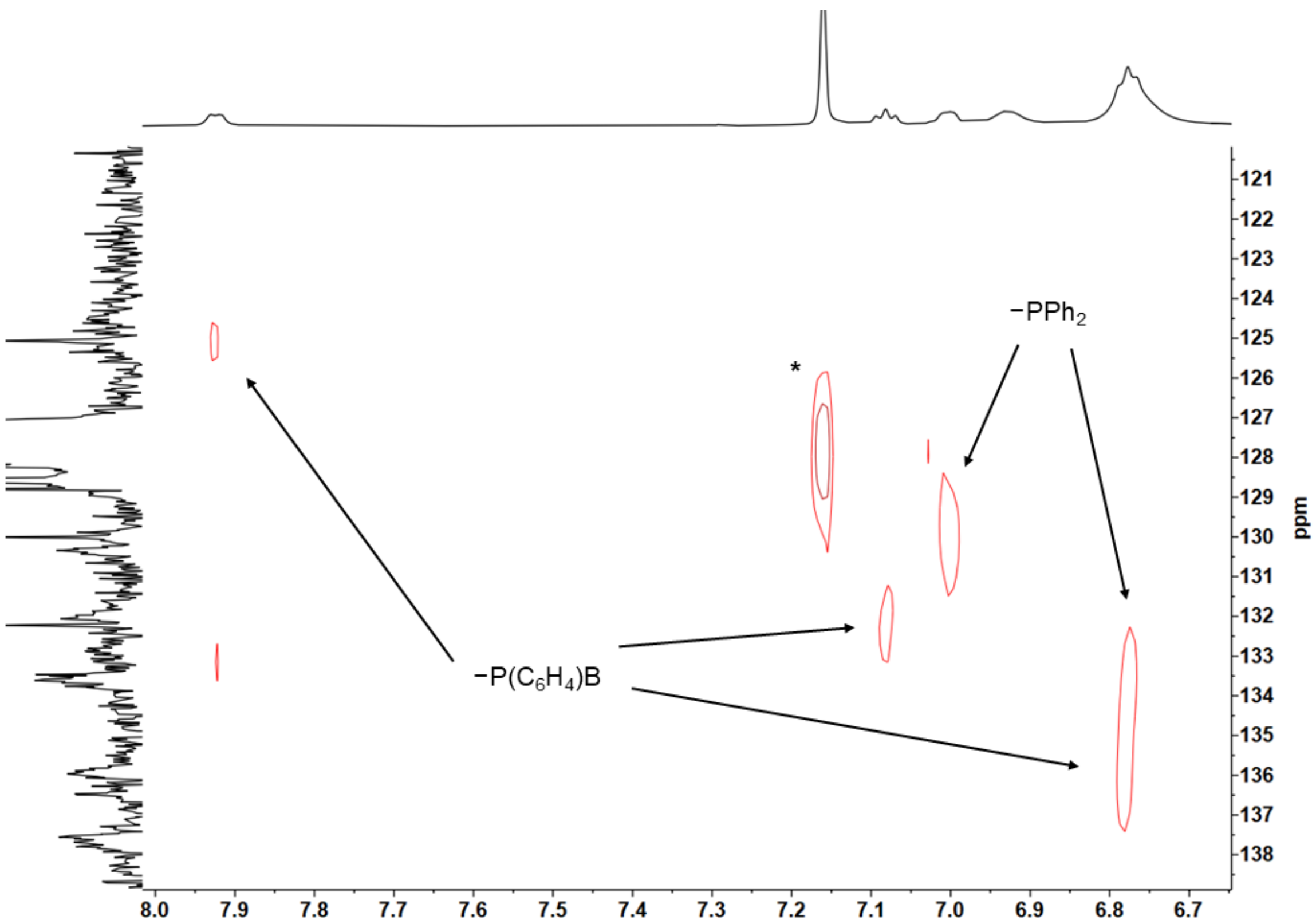

S-Figure 86: HMBC NMR spectrum (600.15/150.92 MHz, C_6D_6, 298 K) of $[(TPB)Al(IMe_4)]^+$ **11**. C_6D_6 is marked with *.

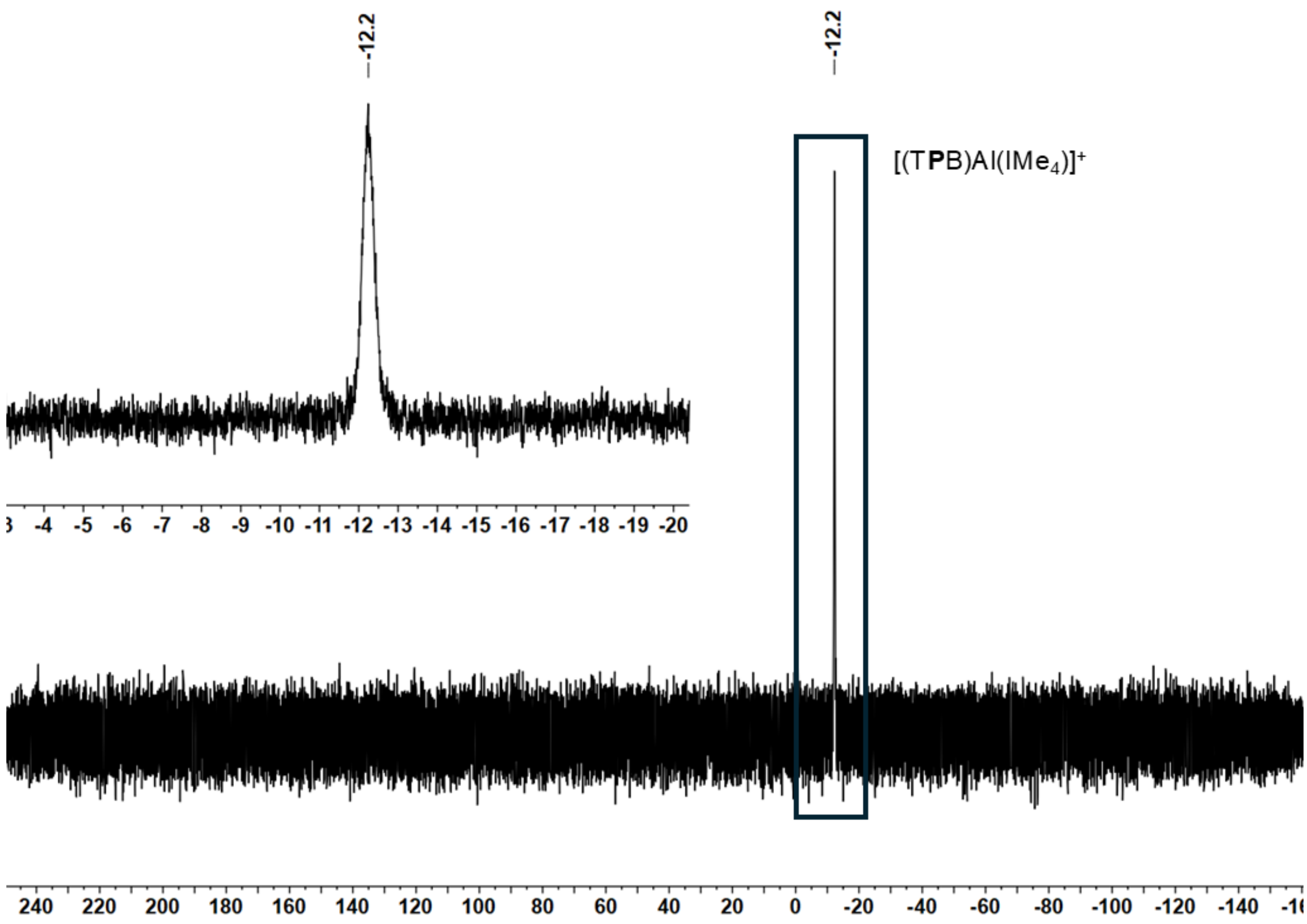

S-Figure 87: ^{31}P NMR spectrum (242.96 MHz, C_6D_6, 298 K) of $[(TPB)Al(IMe_4)]^+$ **11**.

S-1.19 Preparation of [Al(bipy)$_3$]Br 12

Procedure: [(TPB)AlBr] (30.7 mg, 34.1 µmol) was dissolved in 1 mL C_6H_6 and 2,2'-bipyridine (16.0 mg, 102 µmol, 3.00 equiv.) was added yielding a dark brown solution. The mixture was stirred for 10 min, and then the solvent was removed in vacuo. The residual solid was washed with pentane and dried thoroughly affording [Al(bipy)$_3$]Br and free TPB ligand. Crystals of the complex were obtained by layering a solution of [Al(bipy)$_3$]Br in 1,2-DFB with pentane.

Analysis: The ^{1}H NMR spectrum shows signals assigned to the free TPB ligand and the free bipy ligand. The ^{11}B NMR spectrum shows no signal, and the ^{31}P NMR spectrum shows a signal at -9.6 ppm, which is assigned to the free TPB ligand. No signals corresponding to the starting material [(TPB)AlBr] were observed. The EPR spectrum (1,2-DFB, 100 K) shows a single signal with *g* = 2.00342.

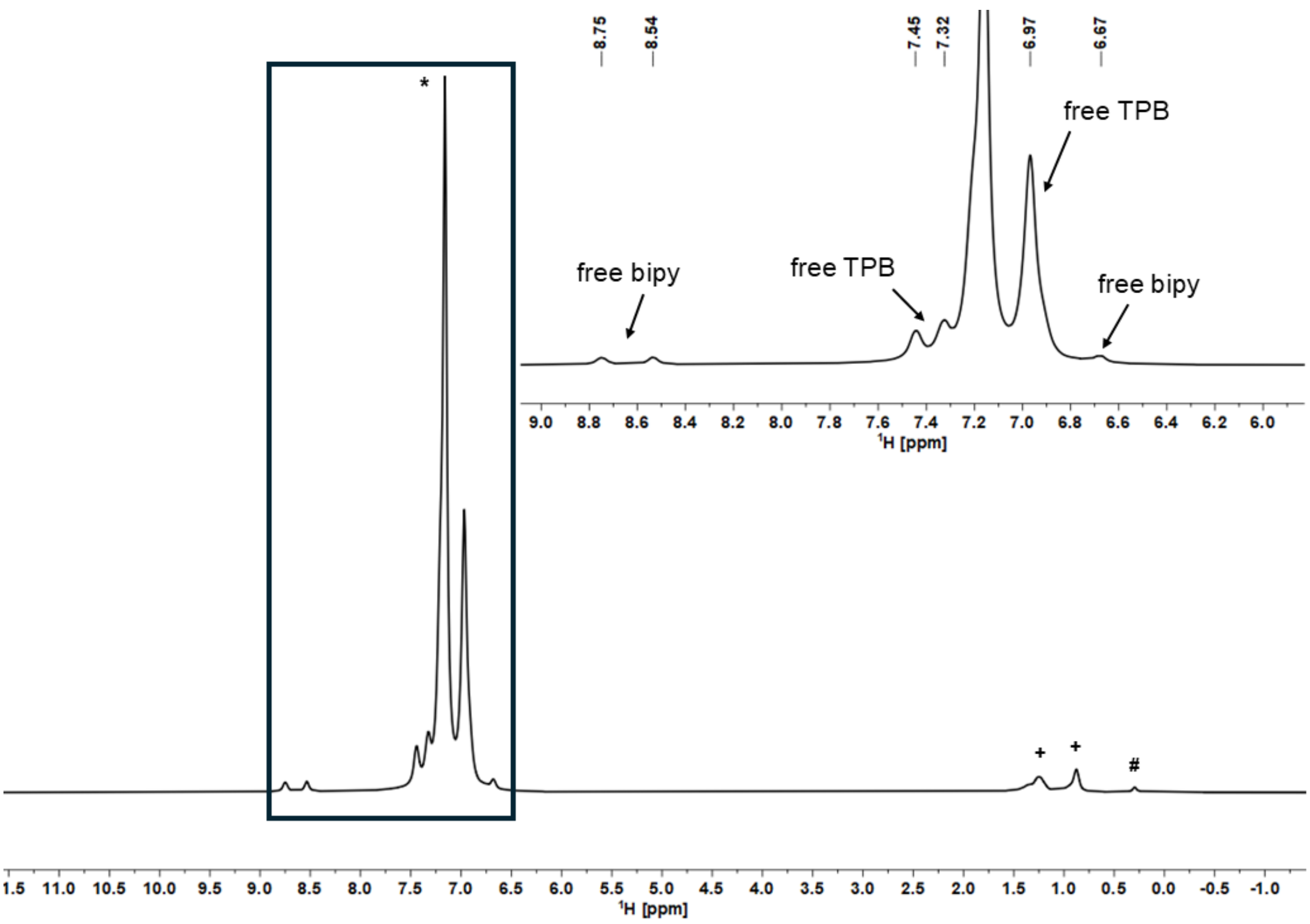

S-Figure 88: ^{1}H NMR spectrum (400.20 MHz, C_6D_6, 298 K) of [Al(bipy)$_3$]Br **12**. Signals of the free TPB and bipy ligand are assigned. C_6D_6 is marked with *, silicon grease with #, and residual pentane with +.

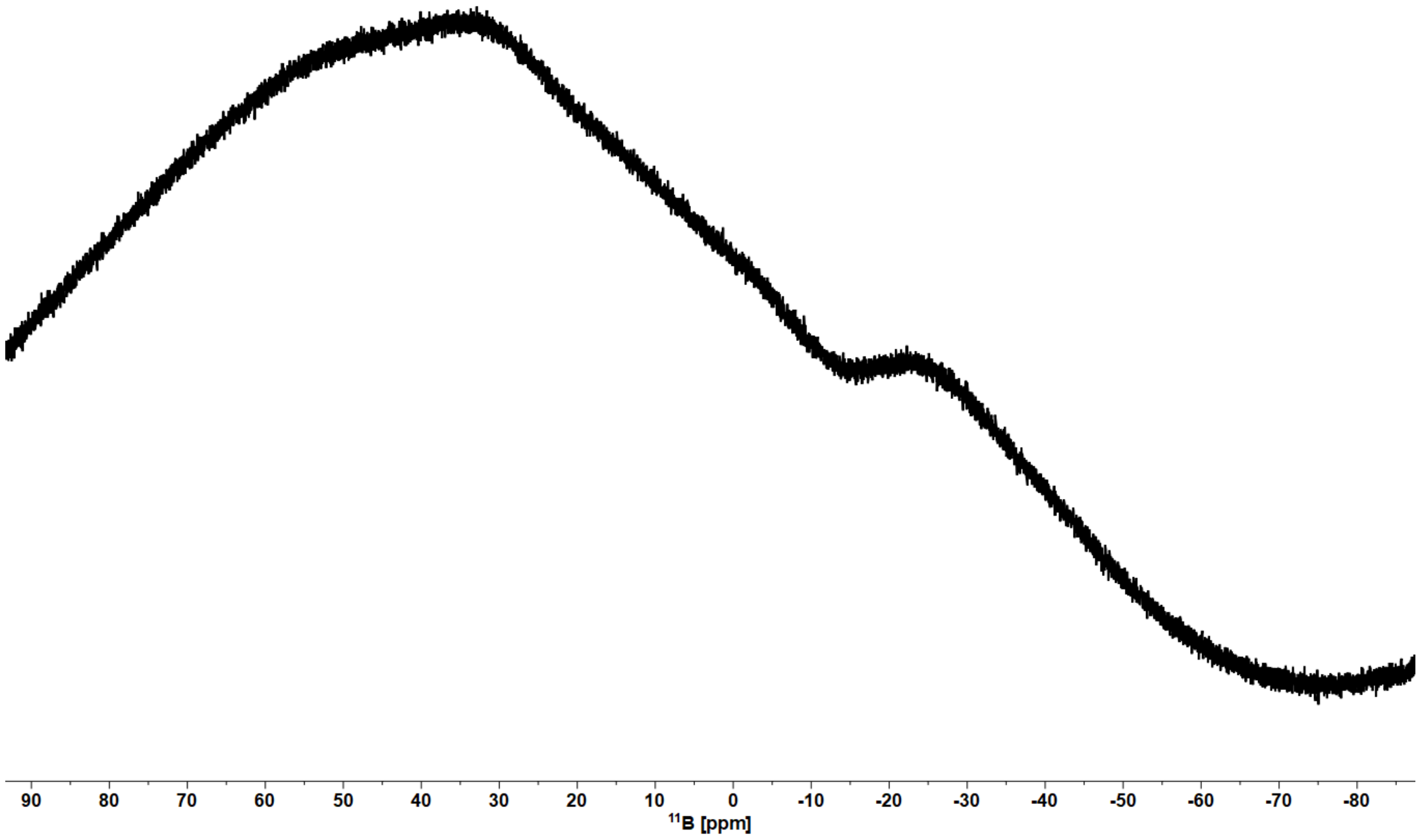

S-Figure 89: ^{11}B NMR spectrum (128.20 MHz, C_6D_6, 298 K) of [Al(bipy)$_3$]Br **12**.

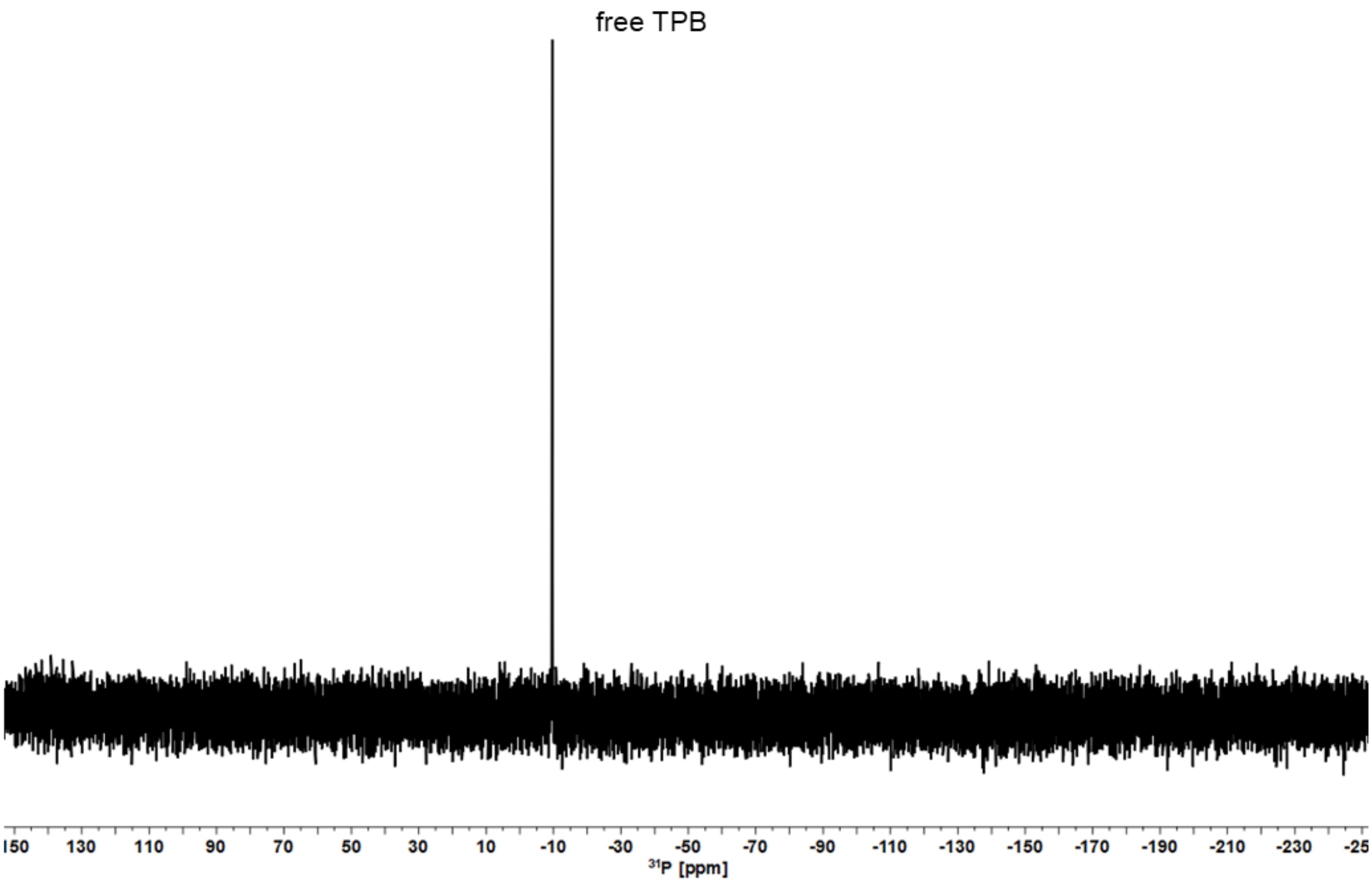

S-Figure 90: ^{31}P NMR spectrum (162.00 MHz, C_6D_6, 298 K) of [Al(bipy)$_3$]Br **12**.

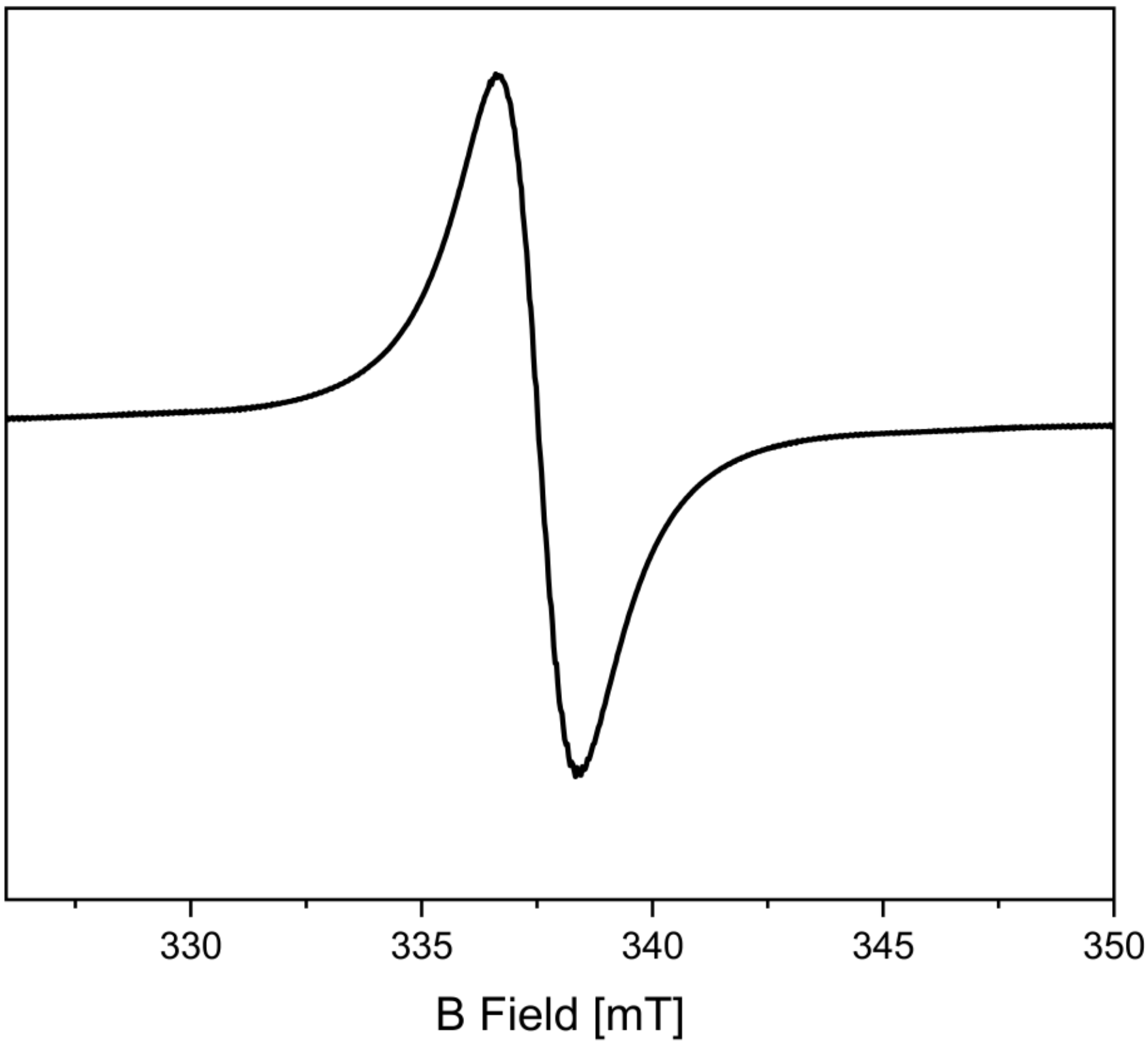

S-Figure 91: EPR spectrum (1,2-DFB, 100 K) of $[Al(bipy)_3]Br$ **12**.

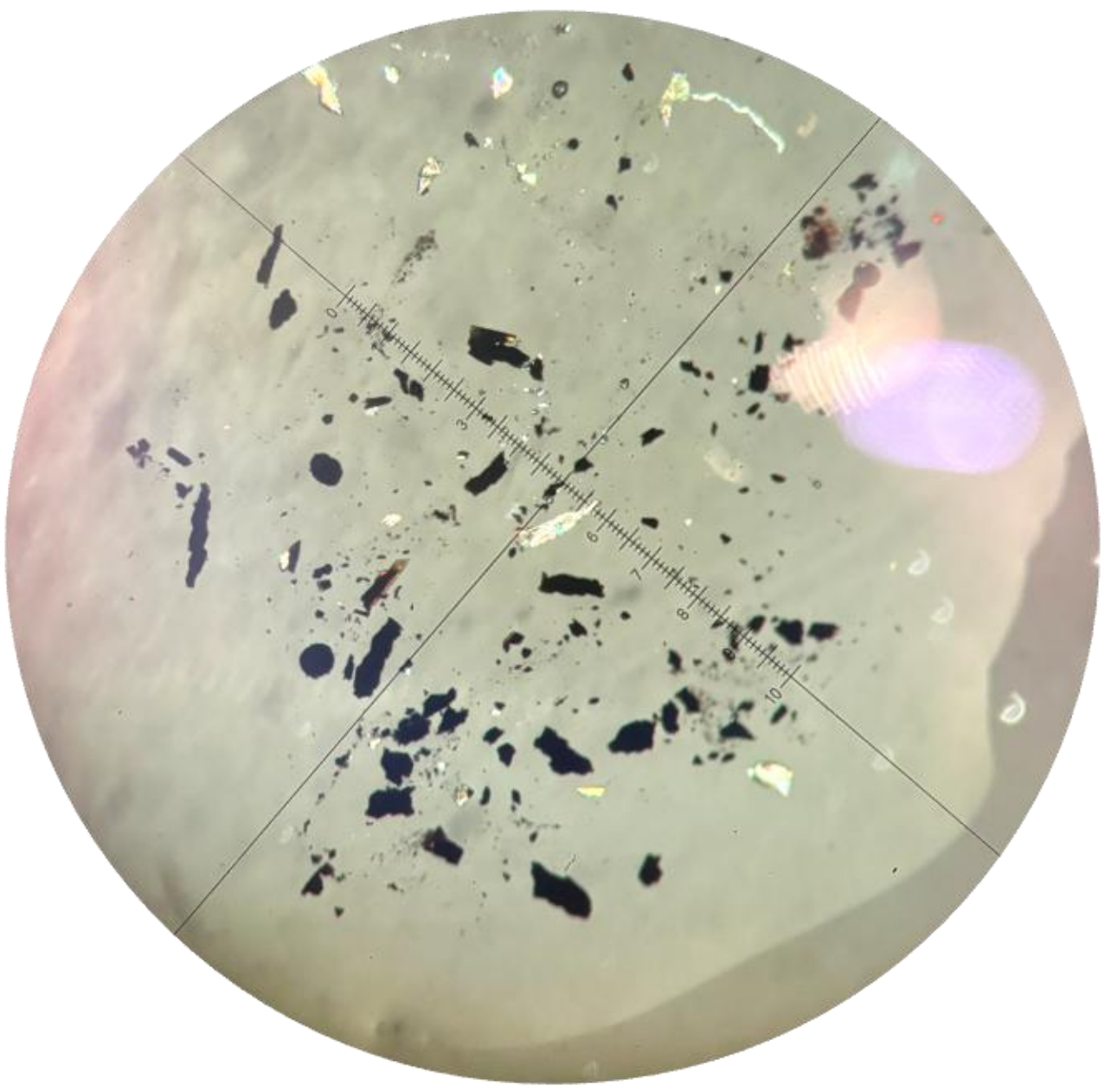

S-Figure 92: Picture of crystals of $[Al(bipy)_3]Br$ **12** obtained from crystallization of the reaction mixture.

S-2 Supporting quantum-chemical calculations

S-2.1 Computational details

Geometry optimizations were performed with the Orca (v6.0.1)[15–17] using DFT functional r^2-SCAN-3c[18] with the def2-mTZVPP basis set[18] (with D4 dispersion correction,[19,20] with RI-J approximation,[18] with geometrical counterpose correction gCP[21]). All structures were checked for imaginary frequencies. Thermal and entropic contributions to the Gibbs energy were calculated at the r^{2}Scan-3c(D4)/def2-mTZVPP level of DFT. Single-point calculations on the pre-optimized structures were performed at B3LYP[22–24](D4)/def2-TZVP[25] level of theory (tightSCF, with RIJCOSX approximation[26,27]) and the obtained SCF energies were used for reaction thermodynamics. Gibbs free energies of solvation were calculated with the CPCM model as implemented in Orca (v6.0.1).[28] QTAIM calculations were performed with the MultiWFN program package[29] and AIMAll Studio. The wfn-file for [(TPB)AlI] was obtained based on a single-point calculation on PBE0(D4)/ZORA-def2-TZVP//r^{2}SCAN-3c level of theory, since ECPs are incompatible with the QTAIM analysis. TDDFT computation was performed on pre-optimized structures at CAM-B3LYP[30](D3BJ)/def2-TZVP level of TDDFT.

EDA-NOCV analysis[31] of the compounds was performed as implemented in the ADF program package (2024.101)[32]. All EDA-NOCV calculations were performed on BP86[33,34](D3BJ)[35,36]/TZ2P[26]//BP86-D3BJ/def2-SVP level of DFT (numerical quality verygood, nosym).

S-2.2 RMSD analysis of experimental and optimized structures

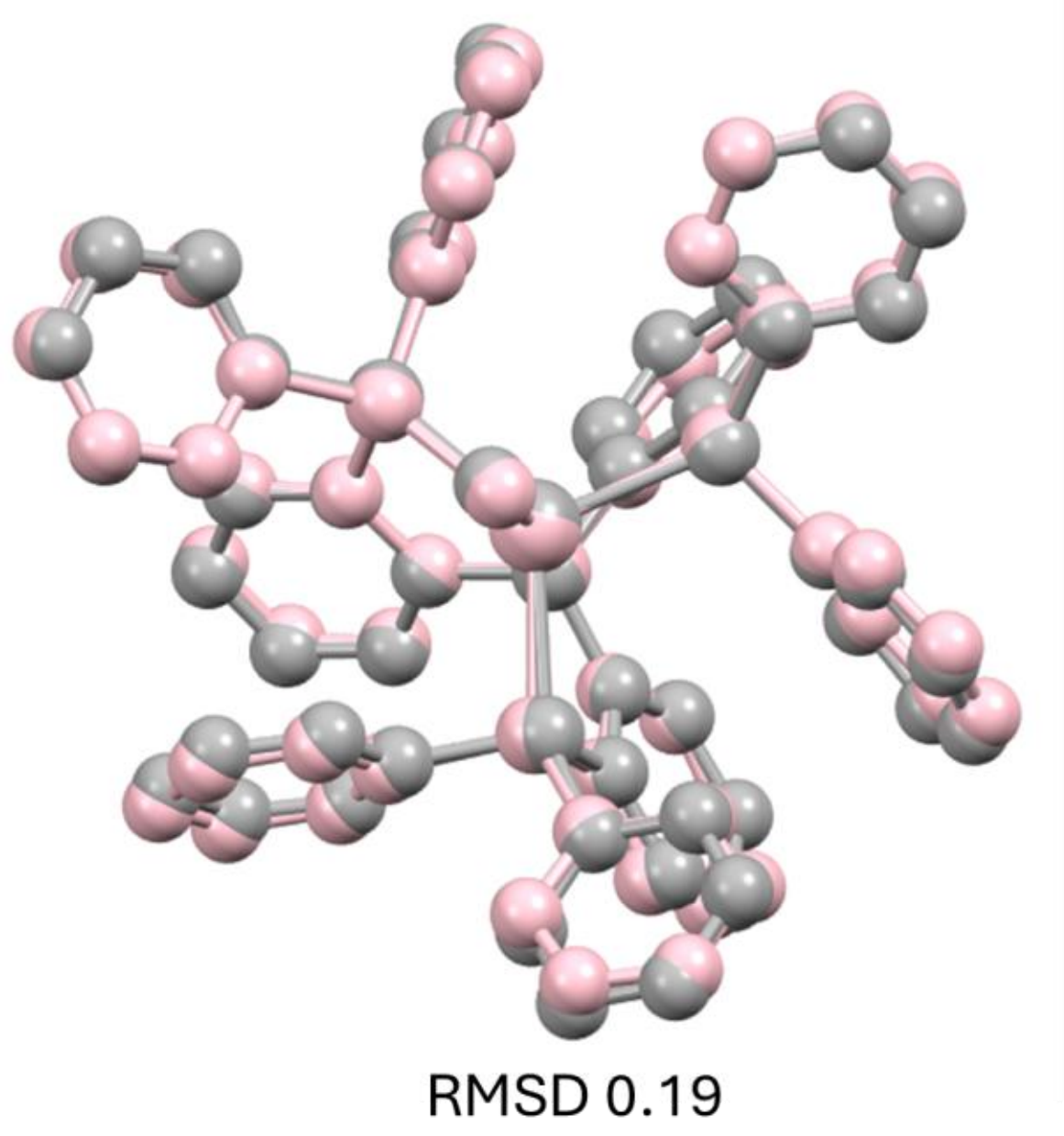

Bond	*d*(Exp.) [Å]	*d*(Comp.) [Å]
Al–Cl	2.1913(4)	2.2356
Al–B	2.211(1)	2.222
Al–P1	2.4457(4)	2.454
Al–P2	2.5418(6)	2.5188
Al–P3	2.5864(6)	2.5373

S-Figure 93: RMSD analysis of experimental and optimized structures for [(TPB)AlCl] **4** as well as the comparison of selected bonds. Structures optimized at the r^2SCAN-3c level of DFT with CPCM(Fluorobenzene).

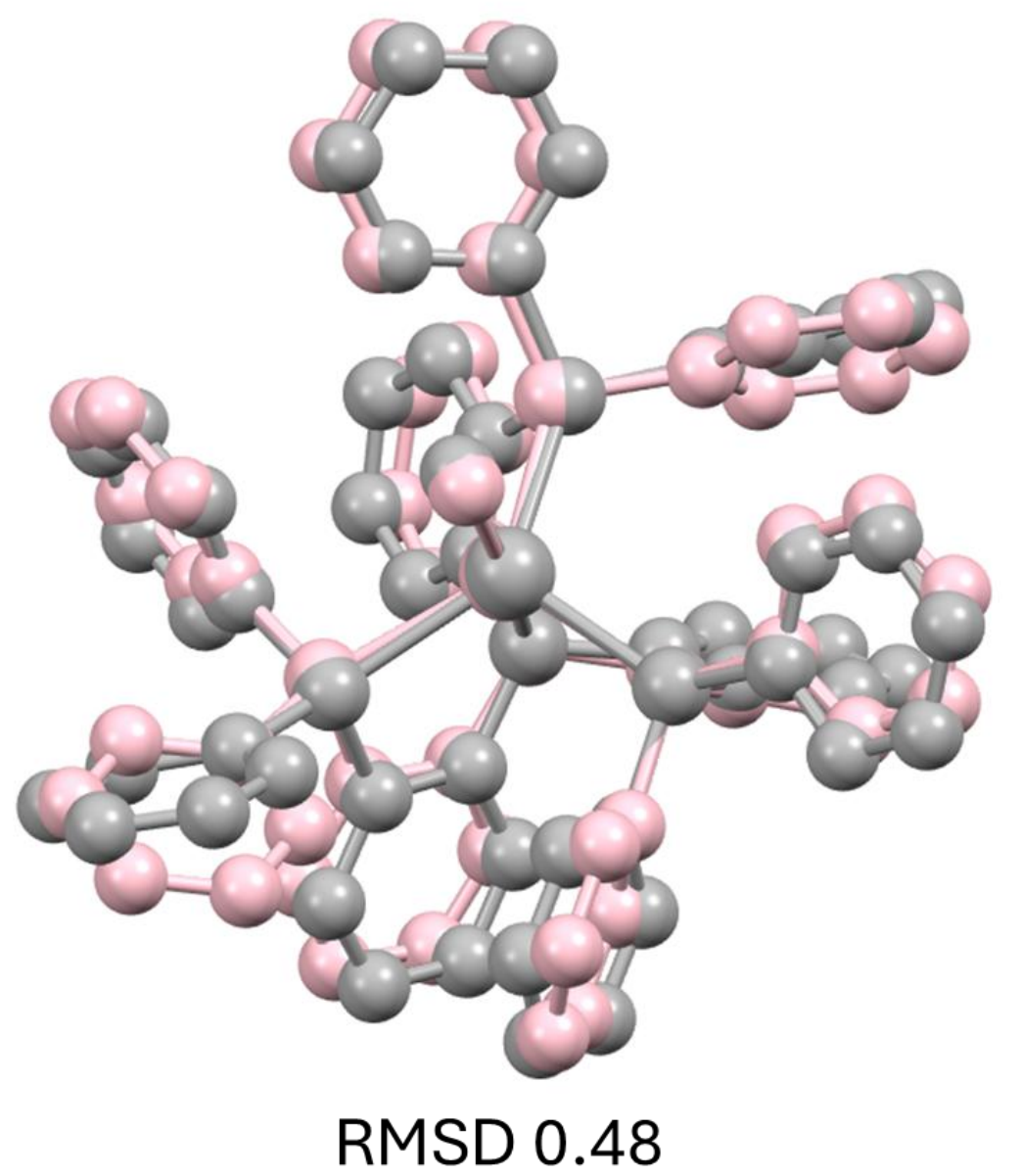

Bond	*d*(Exp.) [Å]	*d*(Comp.) [Å]
Al–Br	2.3500(4)	2.4229
Al–B	2.190(1)	2.220
Al–P1	2.4433(5)	2.4608
Al–P2	2.6004(5)	2.5245
Al–P3	2.5603(5)	2.5131

S-Figure 94: RMSD analysis of experimental and optimized structures for [(TPB)AlBr] **5** as well as the comparison of selected bonds. Structures optimized at the r^2SCAN-3c level of DFT with CPCM(Fluorobenzene).

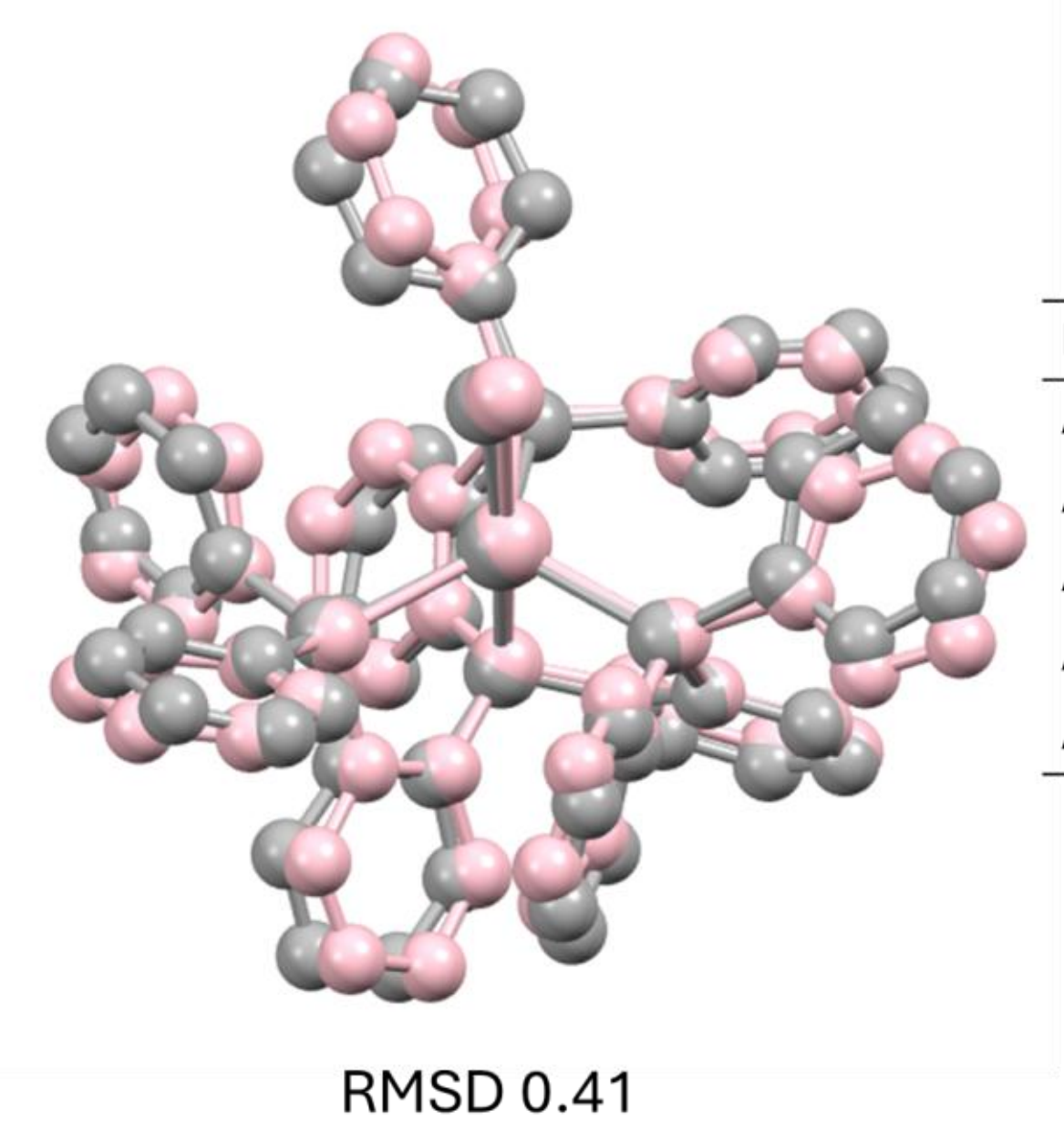

Bond	*d*(Exp.) [Å]	*d*(Comp.) [Å]
Al–I	2.623(2)	2.6821
Al–B	2.213(7)	2.216
Al–P1	2.484(2)	2.474
Al–P2	2.529(2)	2.507
Al–P3	2.532(2)	2.511

S-Figure 95: RMSD analysis of experimental and optimized structures for [(TPB)AlI] **6** as well as the comparison of selected bonds. Structures optimized at the r^{2}SCAN-3c level of DFT with CPCM(Fluorobenzene).

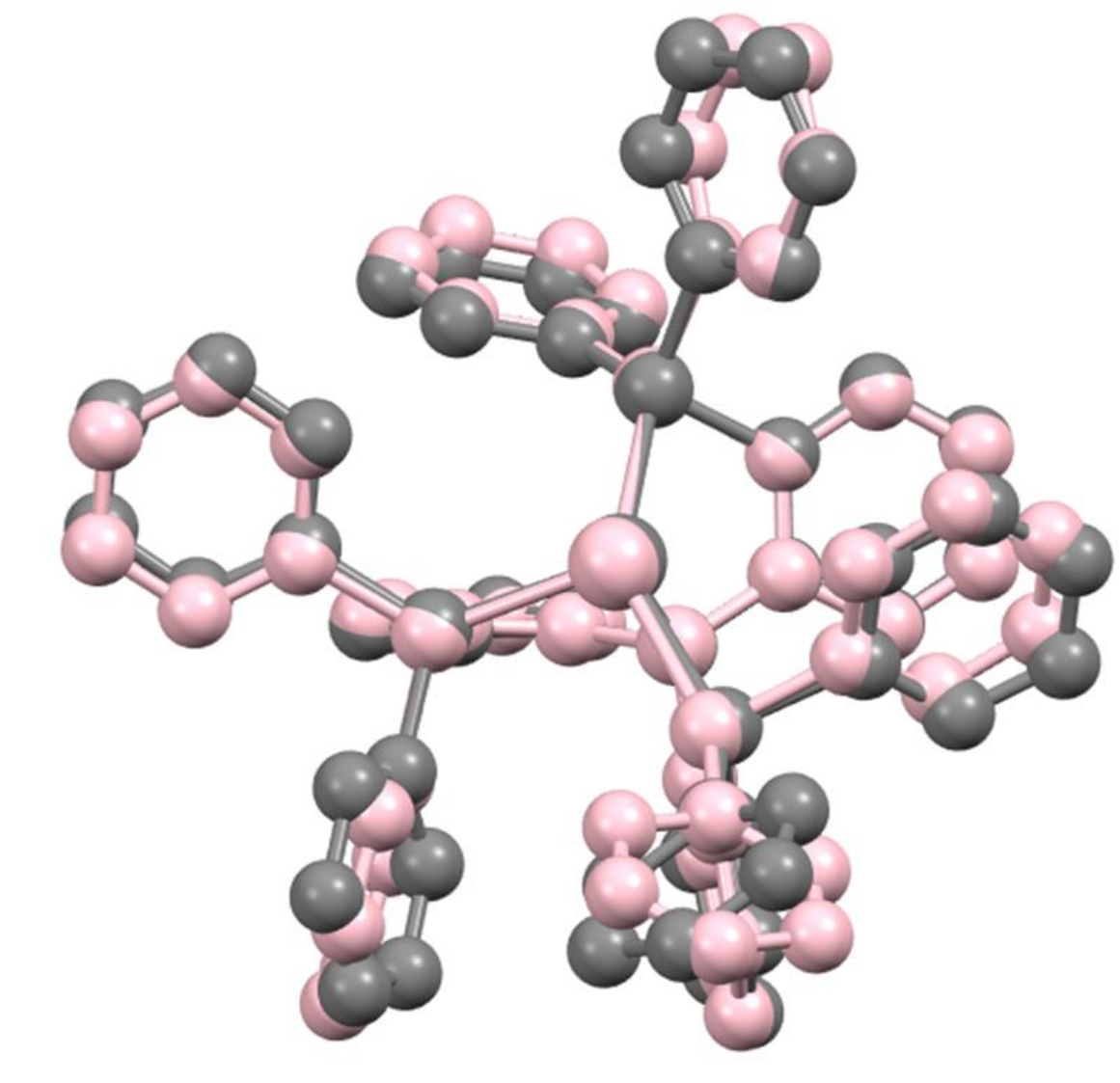

Bond	*d*(Exp.) [Å]	*d*(Comp.) [Å]
Al–H	1.73(2)	1.607
Al–B	2.183(3)	2.202
Al–P1	2.523(1)	2.549
Al–P2	2.436(1)	2.426
Al–P3	2.8164(9)	2.666

S-Figure 96: RMSD analysis of experimental and optimized structures for [(TPB)AlH] **7** as well as the comparison of selected bonds. Structures optimized at the r^{2}SCAN-3c level of DFT with CPCM(Fluorobenzene).

S-2.3 Frontier Orbitals of [(TPB)AlX] complexes

Frontier orbitals of compounds **4**-**6** are presented in the following. Here, the HOMO−1 and HOMO−2 display antibonding interactions between the lone pairs at the halides and the P→Al donor-acceptor bonds. This unfavorable interaction is minimized in **4** and **6** by distortion of the symmetry at aluminium. This distortion is reflected in an increase of the energy difference between HOMO−1 and HOMO−2 in the order of **6** < **5** < **4**.

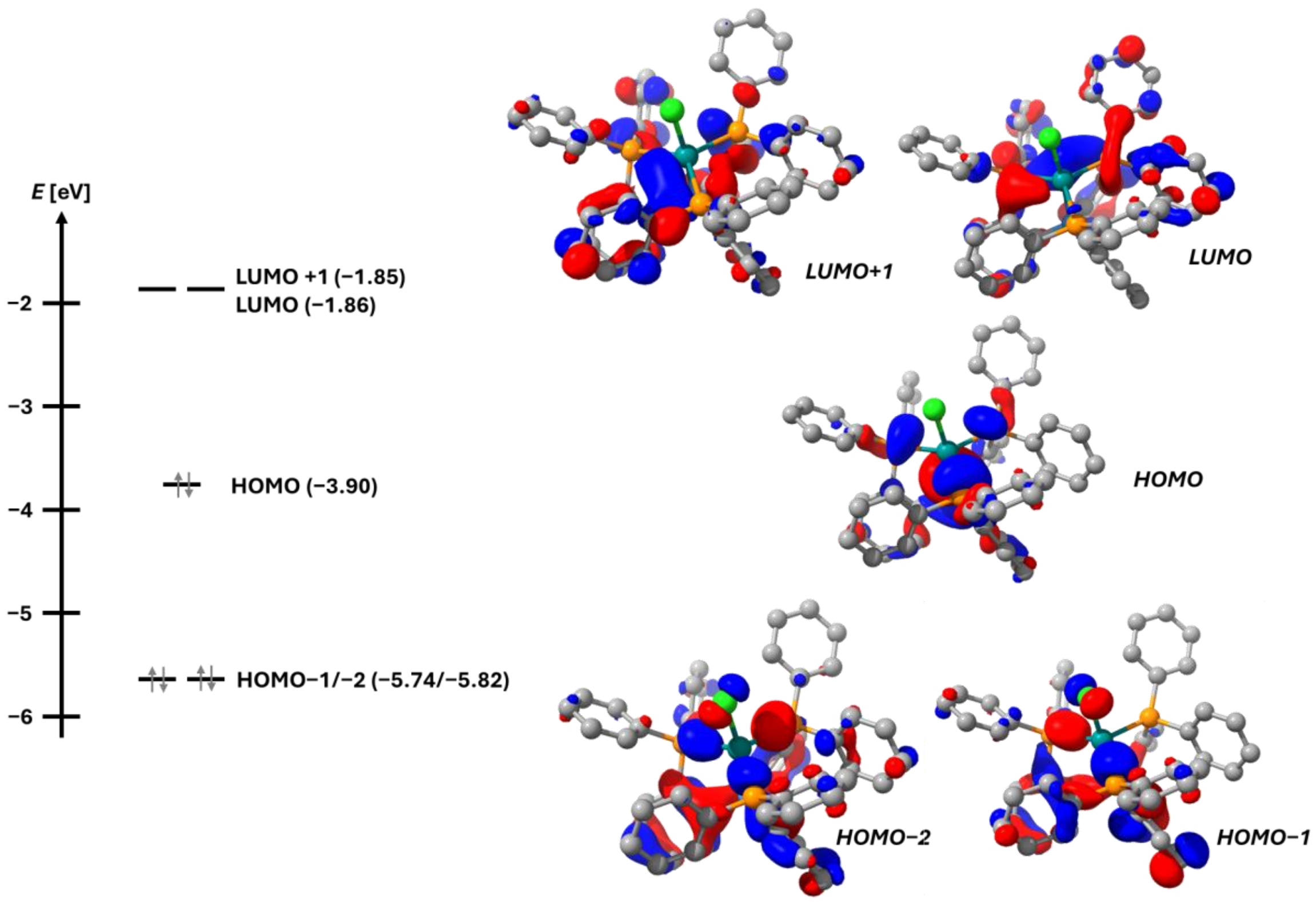

S-Figure 97: Frontier orbitals of **4** (isovalue 0.03).

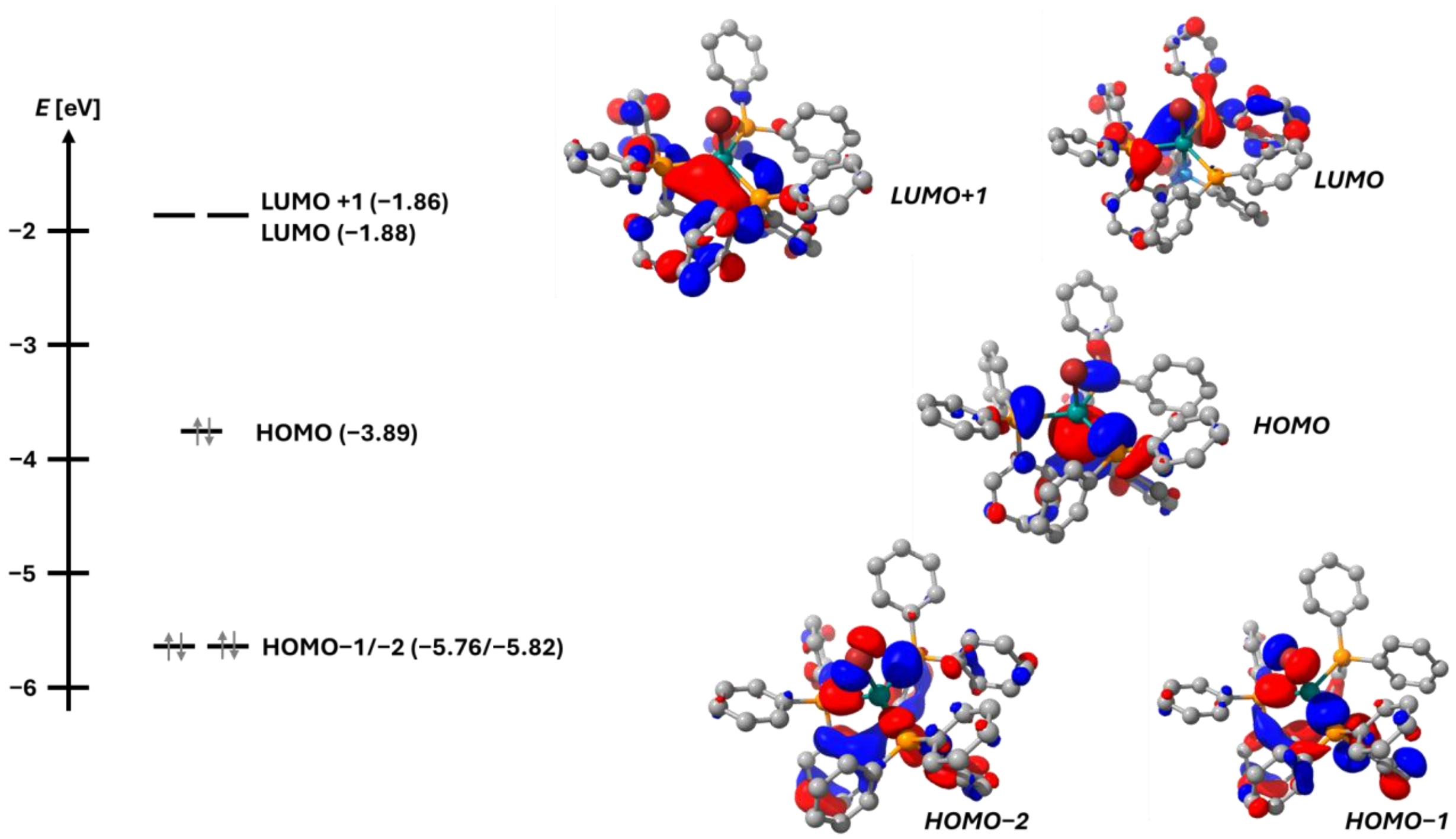

S-Figure 98: Frontier orbitals of **5** (isovalue 0.03).

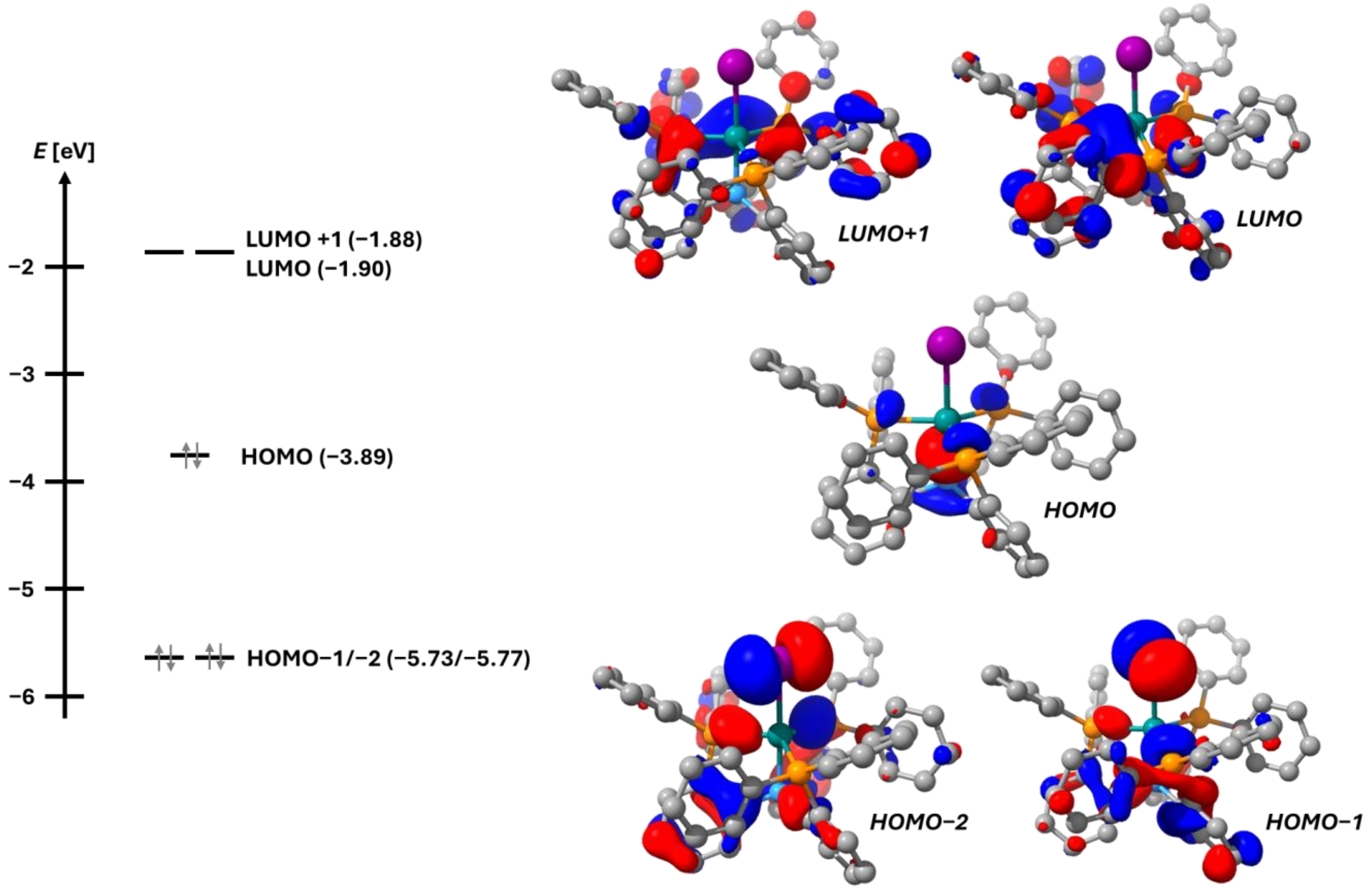

S-Figure 99: Frontier orbitals of **6** (isovalue 0.03).

S-2.4 QTAIM analysis of [(TPB)AlX] complexes

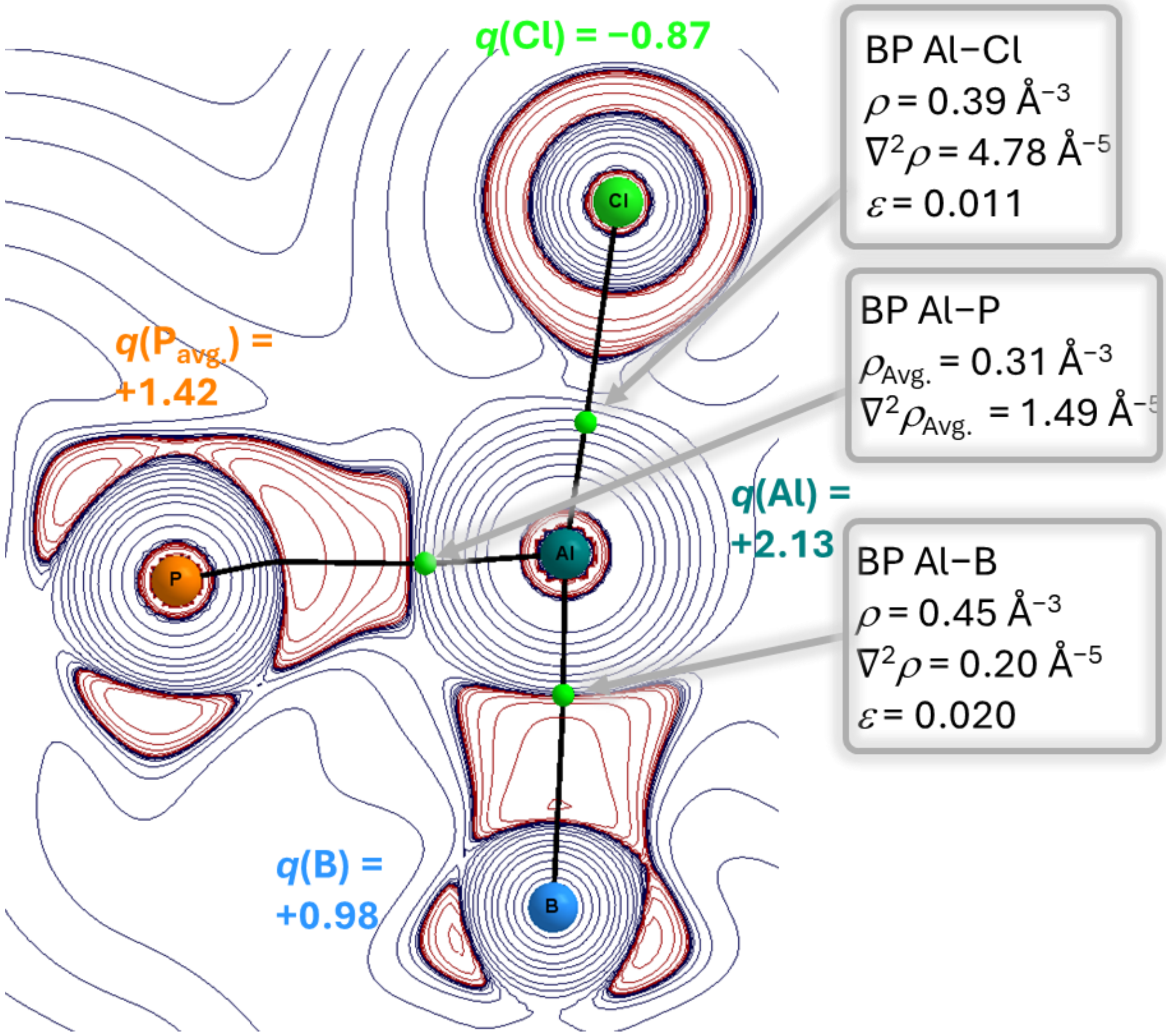

S-Figure 100: Results of QTAIM analysis for **4** (DFT-Structure). ρ = electron density; $\nabla^2\rho$ = Laplacian of electron density; ε = ellipticity; q = atomic charge. Plane defined by P, Al and B atoms.

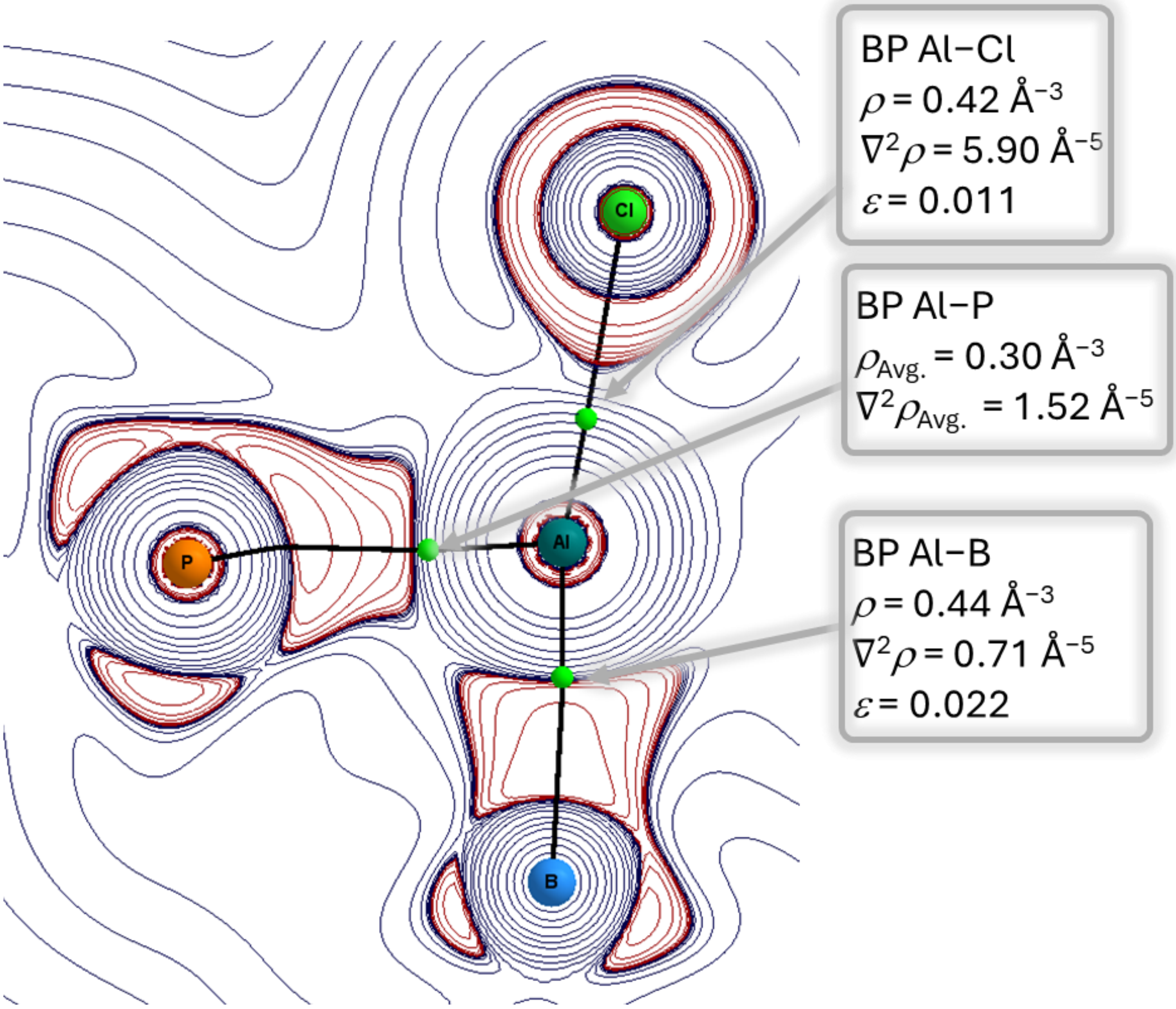

S-Figure 101: Results of QTAIM analysis for **4** (scXRD-Structure from HAR refinement). ρ = electron density; $\nabla^2\rho$ = Laplacian of electron density; ε = ellipticity; q = atomic charge. Plane defined by P, Al and B atoms.

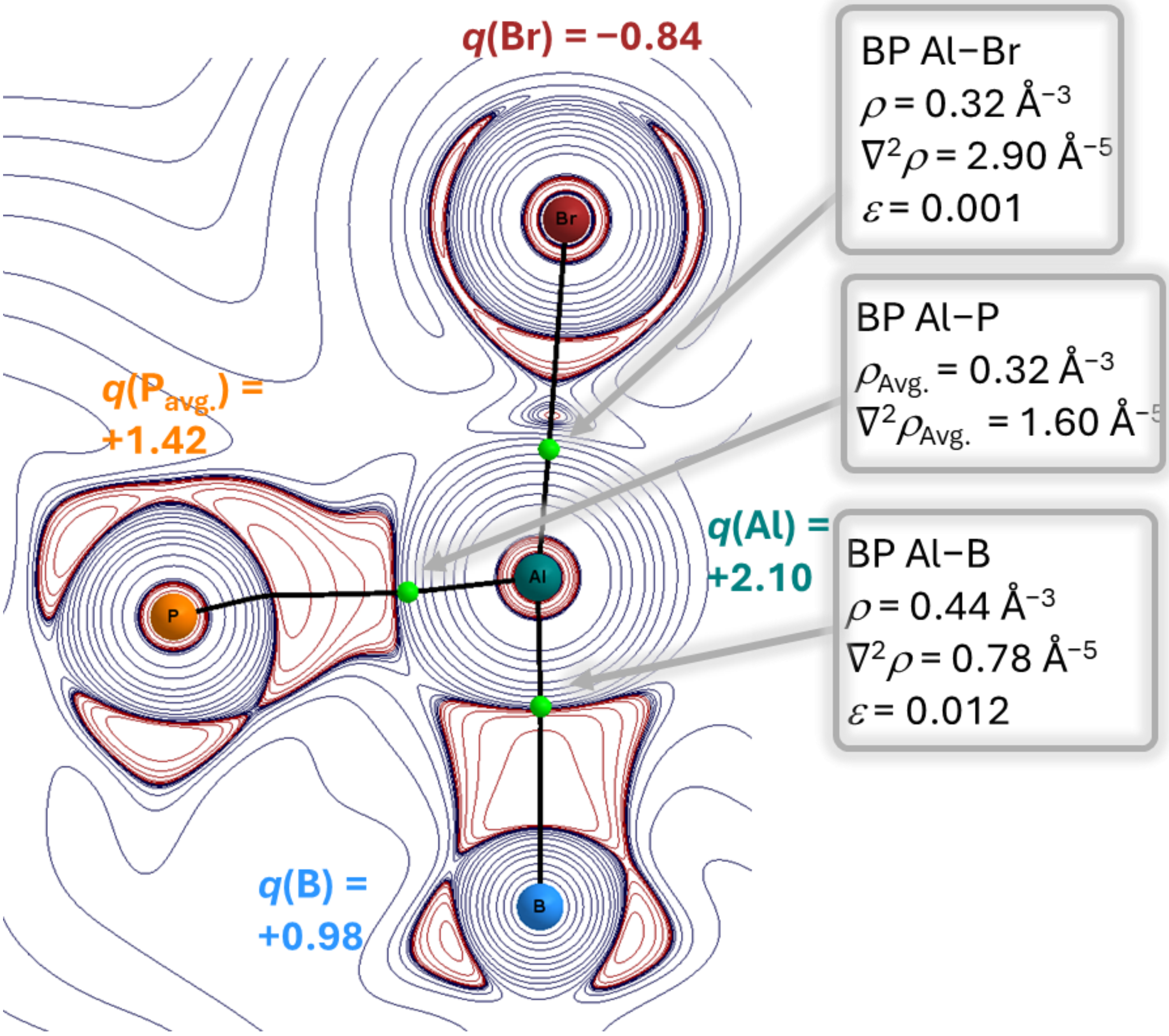

S-Figure 102: Results of QTAIM analysis for **5** (DFT-Structure) . ρ = electron density; $\nabla^2\rho$ = Laplacian of electron density; ε = ellipticity; q = atomic charge. Plane defined by P, Al and B atoms.

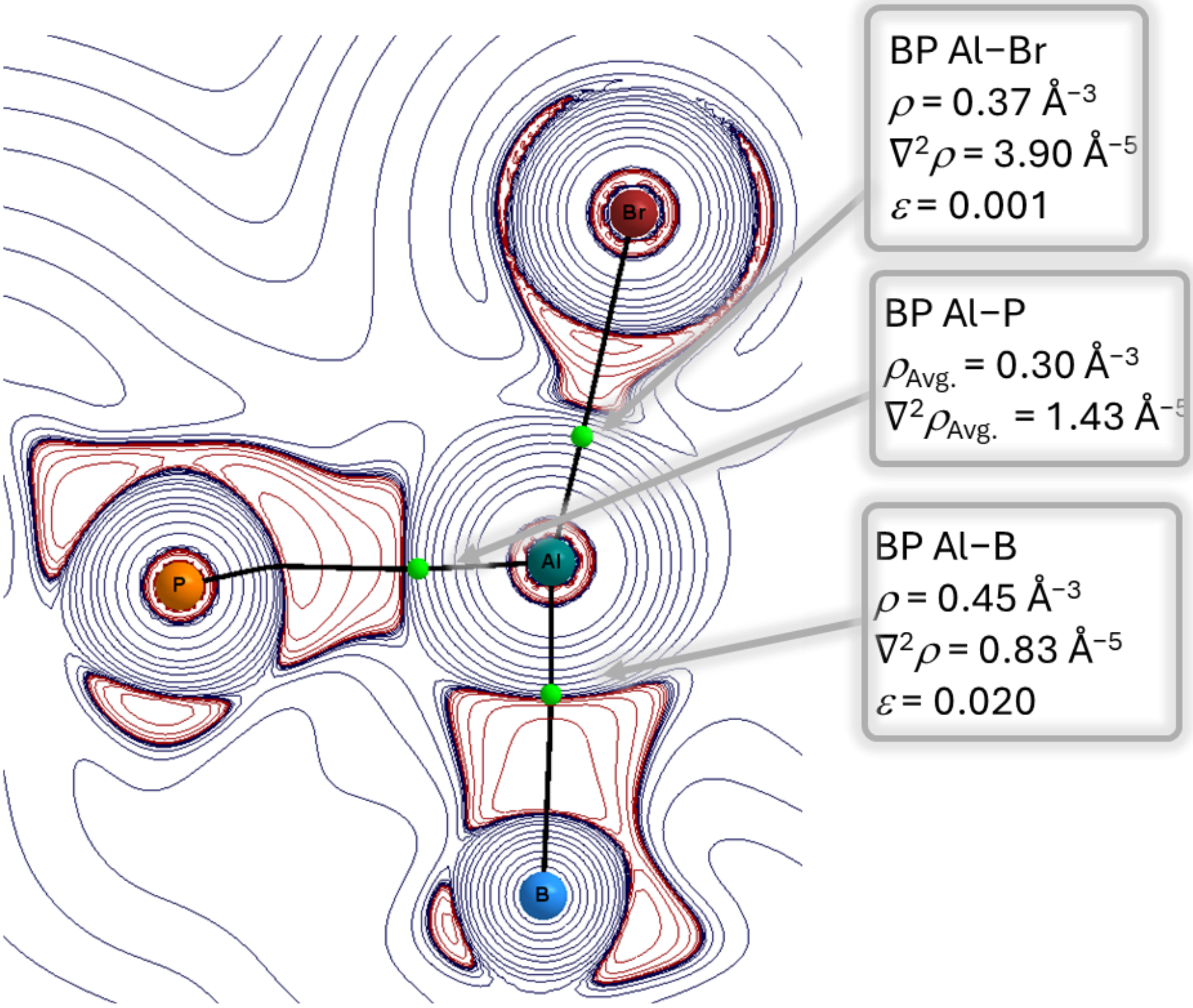

S-Figure 103: Results of QTAIM analysis for **5** (scXRD-Structure from HAR refinement). ρ = electron density; $\nabla^2\rho$ = Laplacian of electron density; ε = ellipticity; q = atomic charge. Plane defined by P, Al and B atoms.

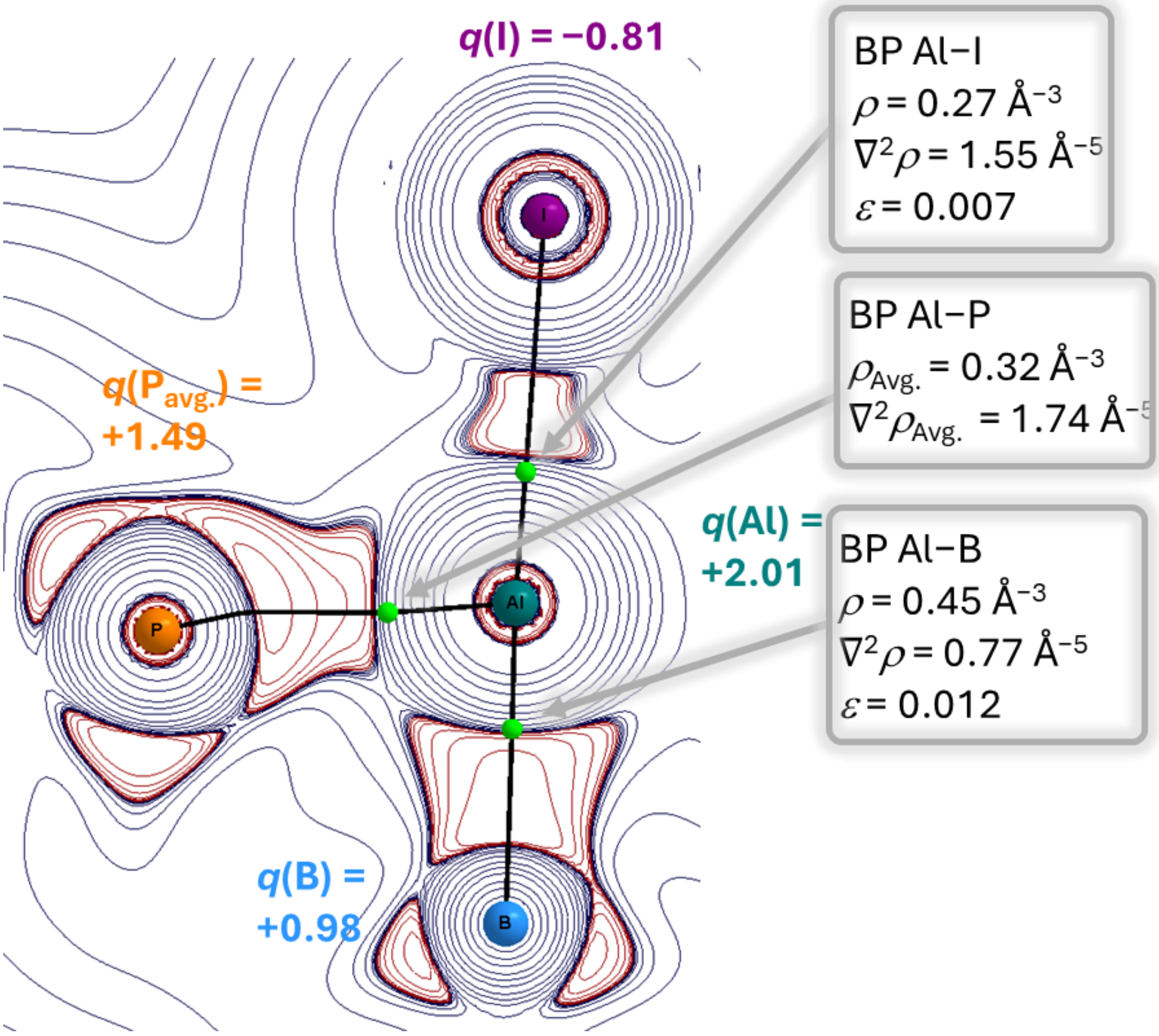

S-Figure 104: Results of QTAIM analysis for **6** (DFT-Structure). ρ = electron density; $\nabla^2\rho$ = Laplacian of electron density; ε = ellipticity; q = atomic charge. Plane defined by P, Al and B atoms.

S-2.5 QTAIM analysis of [(TPB)AlH] complexes

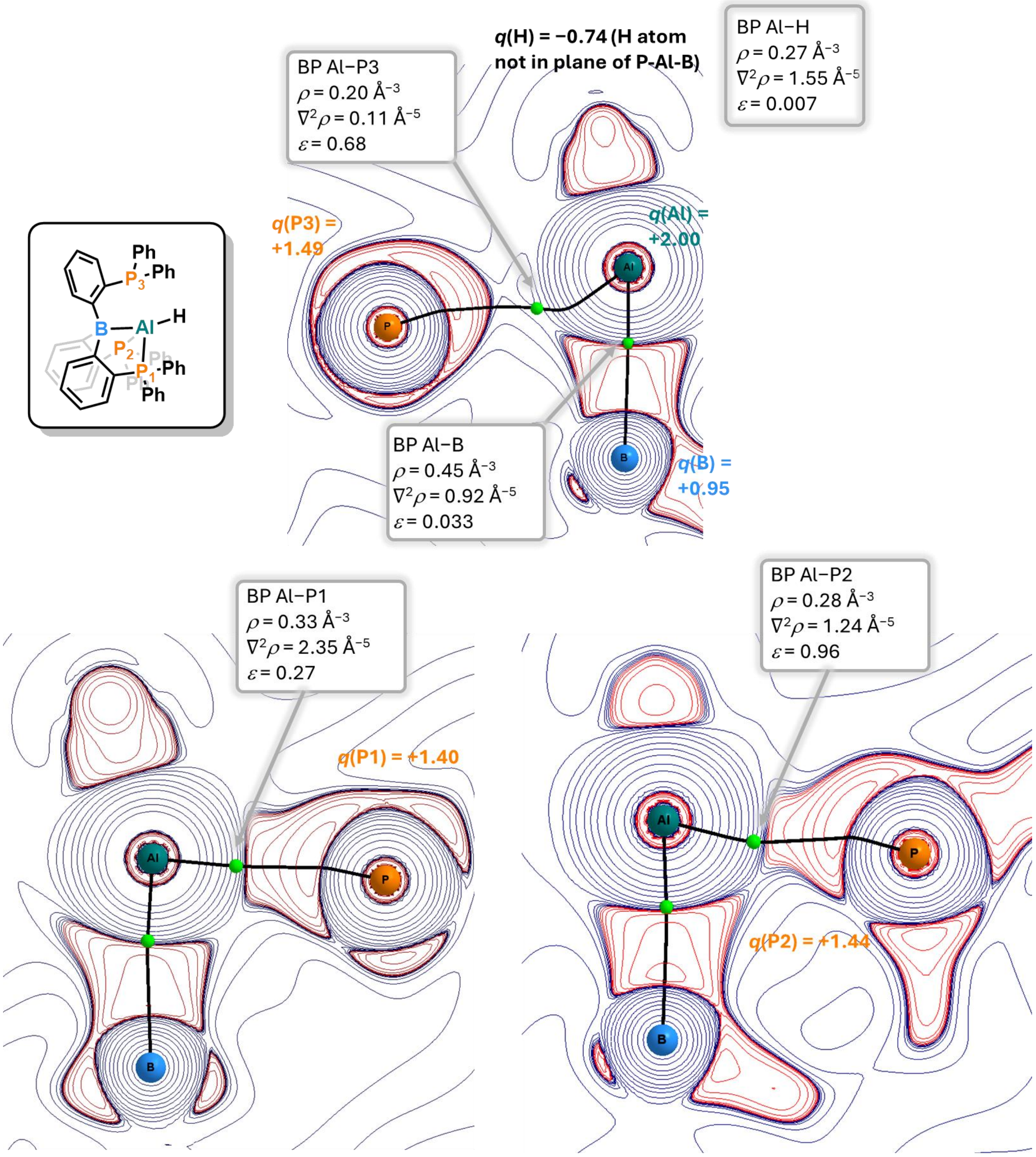

S-Figure 105: Results of QTAIM analysis for **7** (DFT-Structure). ρ = electron density; $\nabla^2\rho$ = Laplacian of electron density; ε = ellipticity; q = atomic charge. Planes defined by P, Al and B atoms.

S-2.6 NBO analysis of [(TPB)AlX] complexes

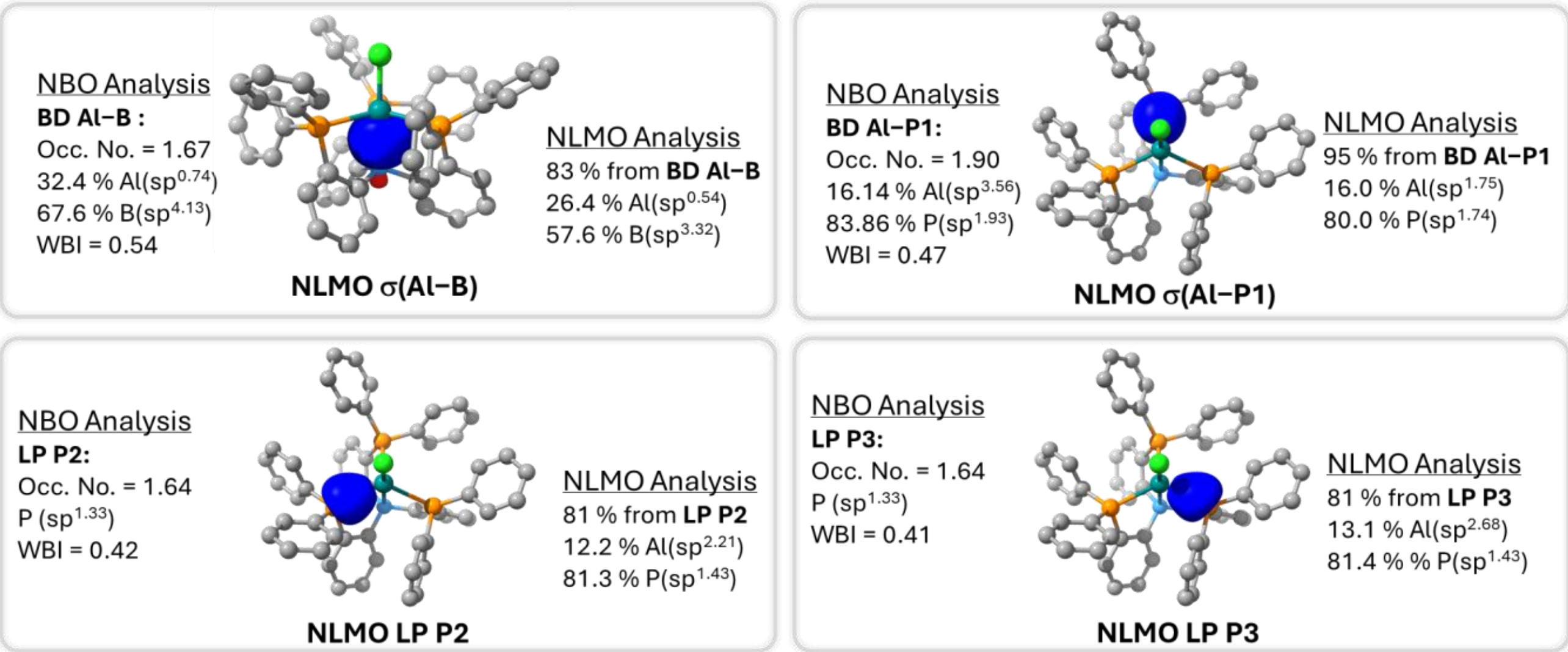

S-Figure 106: Results of NBO analysis for **4** (isovalue 0.07).

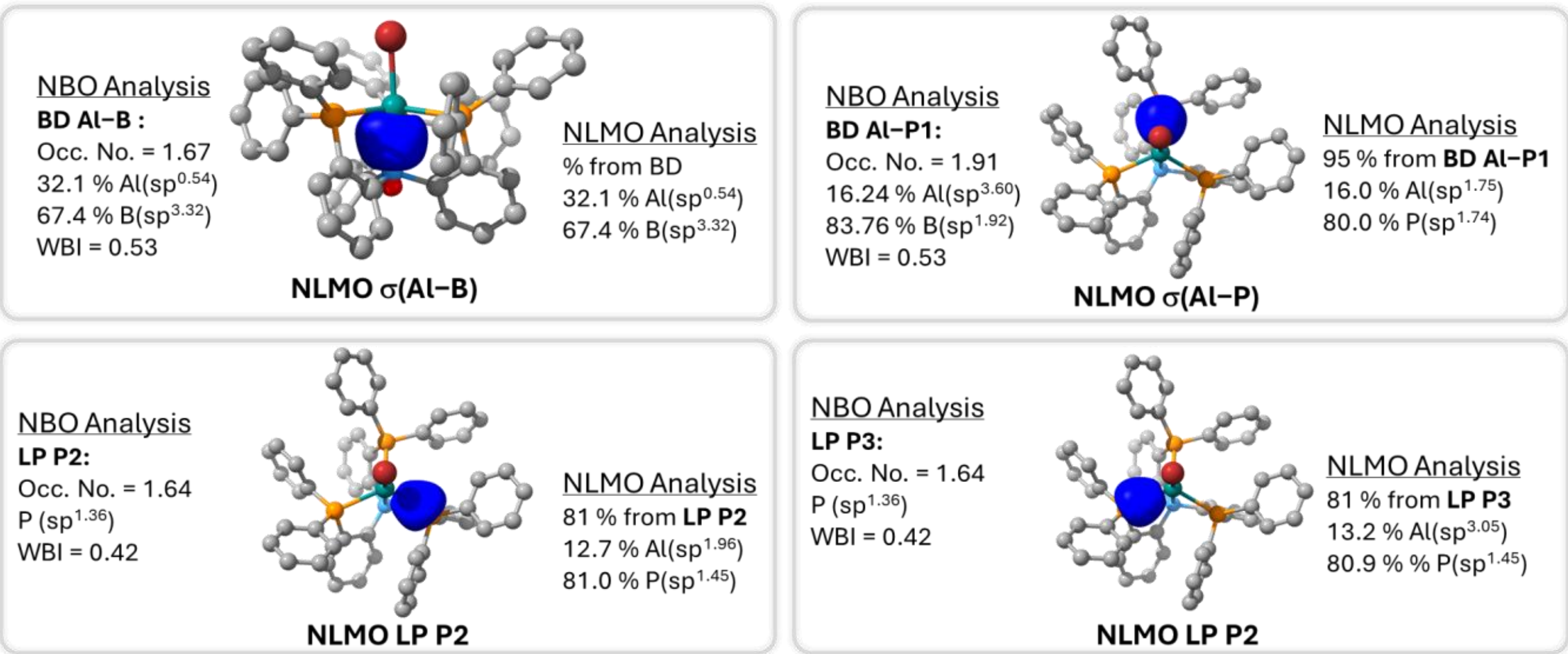

S-Figure 107: Results of NBO analysis for **5** (isovalue 0.07).

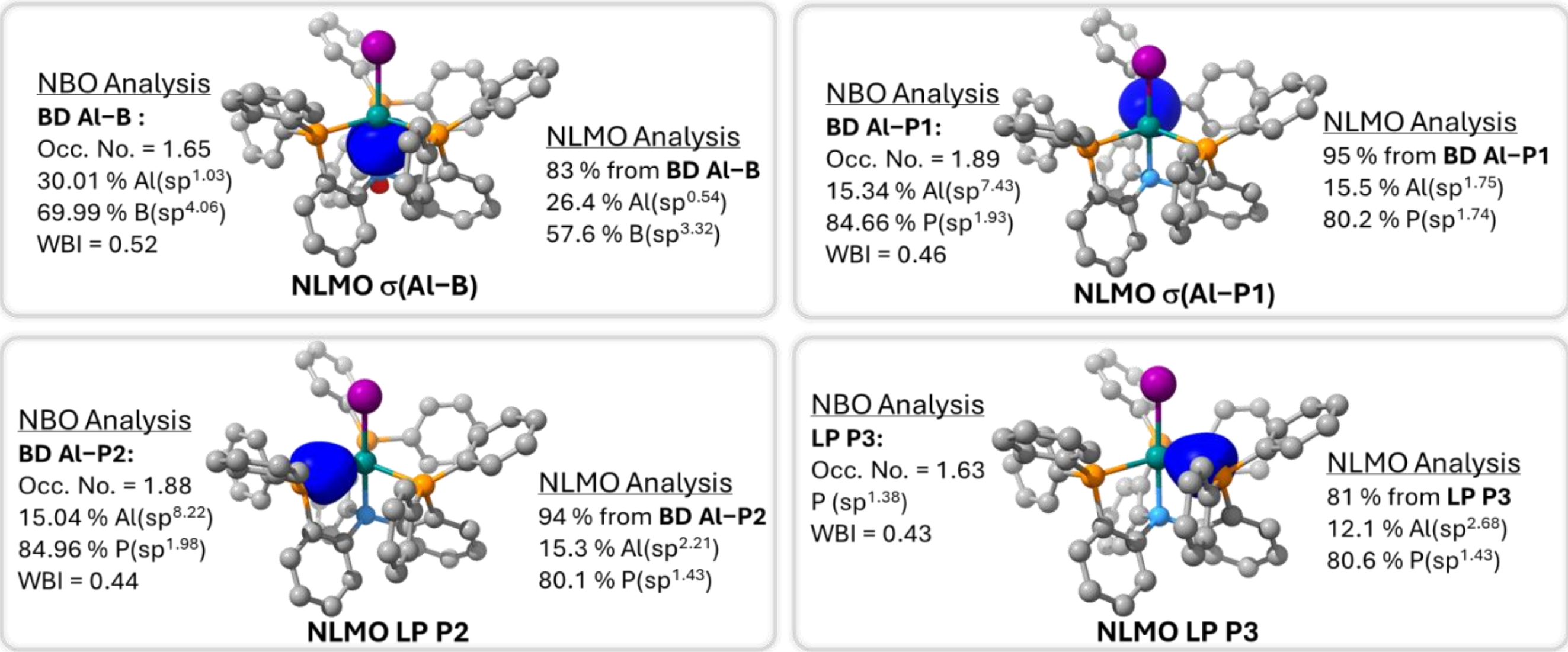

S-Figure 108: Results of NBO analysis for **6** (isovalue 0.07).

S-Table 1: E(2) Values obtained by the Second Order Perturbation Theory Analysis of Fock Matrix in NBO Basis for **5**. Values in kcal mol^{-1}

**Donor NBO**	**Acceptor NBO**	**E(2)**
BD (1)Al 1- B 6	LV (1)Al 1	2.00
BD (1)Al 1- B 6	LV (1) C 83	17.15
BD (1)Al 1- B 6	BD*(1)Al 1- P 3	3.60
BD (1)Al 1- B 6	BD*(1)Al 1- B 6	0.81
BD (1)Al 1- B 6	BD*(1) P 3- C 7	4.40
BD (1)Al 1- B 6	BD*(1) P 3- C 74	1.89
BD (1)Al 1- B 6	BD*(1) P 4- C 29	0.84
BD (1)Al 1- B 6	BD*(1) P 4- C 40	3.59
BD (1)Al 1- B 6	BD*(1) P 4- C 92	2.03
BD (1)Al 1- B 6	BD*(1) P 5- C 62	7.82
BD (1)Al 1- B 6	BD*(1) P 5- C 93	1.29
BD (1)Al 1- B 6	BD*(2) C 73- C 74	2.60
BD (1)Al 1- B 6	BD*(1) C 73- C 81	6.93
BD (1)Al 1- B 6	BD*(1) C 83- C 84	7.07
BD (1)Al 1- B 6	BD*(1) C 83- C 92	0.52
BD (1)Al 1- B 6	BD*(1) C 93- C102	0.72
BD (1)Al 1- B 6	BD*(2) C 93- C102	2.85
BD (1)Al 1- B 6	BD*(1) C100- C102	6.91
BD (1)Al 1- B 6	RY (10) B 6	0.57
BD (1)Al 1- B 6	RY (2) C 51	0.51
BD (1)Al 1- B 6	RY (2) C 73	2.16
BD (1)Al 1- B 6	RY (2) C 83	2.45
BD (1)Al 1- B 6−93.51	RY (2) C102	2.57

S-2.7 EDA-NOCV analysis of [(TPB)AlX] complexes

S-Table 2: EDA-NOCV results (kcal mol^{-1}) for **4** at the BP86-D3(BJ)/TZVP//r^2SCAN-3c level of theory.

**EDA-NOCV**	**Dative** **AlCl (s) + TPB (s)**	**Electron-sharing** **$AlCl^+$ (d) + TPB^- (d)**	**Electron-sharing** **AlCl (t) + TPB (t)**
ΔE_{int}	−93.51	−275.32	−200.69
ΔE_{Pauli}	439.59	258.88	372.19
ΔE_{Elstat}	−221.28	−239.83	−253.80
ΔE_{Disp}	−25.27	−25.27	−25.27
ΔE_{Orb}	−286.55	**−269.11**	−293.81
ΔE_{Orb1} **σ (B−Al)**	−205.92	−113.27	
ΔE_{Orb2} **σ (P−Al)**	−31.47	−47.51	
ΔE_{Orb3} **σ (P−Al)**	−29.89	−45.49	
ΔE_{Orb} **Rest**	−19.27	−62.84	

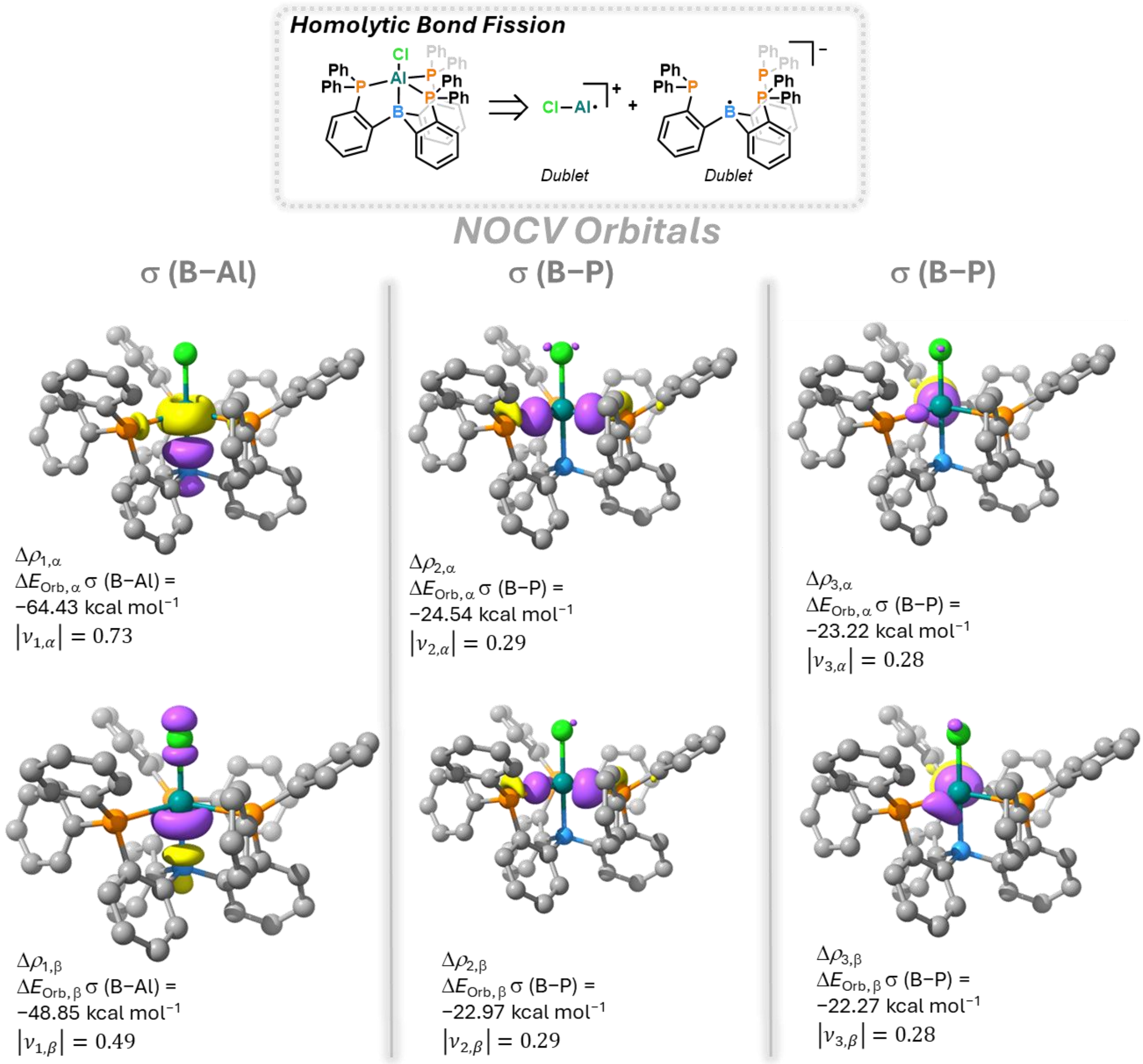

S-Figure 109: Plots of deformation densities $\Delta\rho_{(1)\text{-}(3)}$ of the pairwise orbital interaction for homolytic fragmentation of **4** in $AlCl^{+}$ and TPB^{-} (isovalue 0.004 for B−Al bond, 0.002 for Al−P bonds) associated with energy terms $\Delta E_{Orb(1)\text{-}Orb(3)}$ (values in kcal/mol). Charge flows from yellow to purple.

S-Table 3: EDA-NOCV results (kcal mol^{-1}) for **5** at the BP86-D3(BJ)/TZVP//r^{2}SCAN-3c level of theory.

**EDA-NOCV**	**Dative Al→B**<br>**AlBr (s) + TPB (s)**	**Electron-sharing**<br>**$AlBr^+$ (d) + TPB^- (d)**	**Electron-sharing**<br>**AlBr (t) + TPB (t)**	**Dative B→Al**<br>**$AlBr^{2+}$ (s) + TPB^{2-} (s)**
ΔE_{int}	−96.21	−273.48	−210.77	−832.86
ΔE_{Pauli}	440.09	273.34	371.66	364.80
ΔE_{Elstat}	−221.89	−243.19	−263.04	−432.62
ΔE_{Disp}	−27.52	−27.52	−27.52	−27.52
ΔE_{Orb}	−286.90	**−276.12**	−291.88	−726.51
ΔE_{Orb1} σ (B−Al)	−201.70	−116.12		
ΔE_{Orb2} σ (P−Al)	−32.92	−48.33		
ΔE_{Orb3} σ (P−Al)	−31.41	−46.14		
ΔE_{Orb} Rest	−20.87	−65.53		

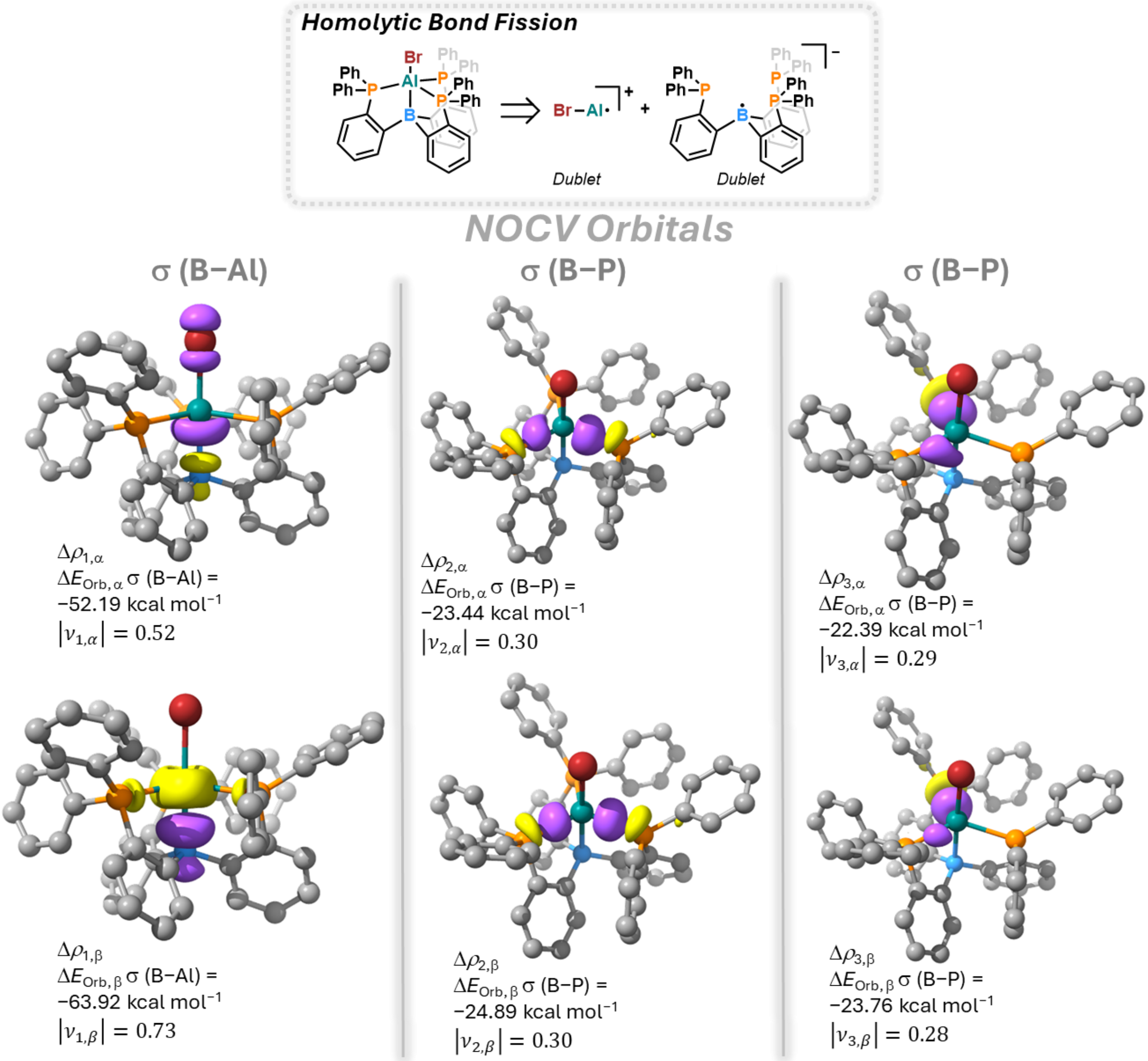

S-Figure 110: Plots of deformation densities $\Delta\rho_{(1)\text{-}(3)}$ of the pairwise orbital interaction for homolytic fragmentation of **5** in $AlBr^{+}$ and TPB^{-} (isovalue 0.004 for B−Al bond, 0.002 for Al−P bonds) associated with energy terms $\Delta E_{Orb(1)\text{-}Orb(3)}$ (values in kcal/mol). Charge flows from yellow to purple.

S-Table 4: EDA-NOCV results (kcal mol^{-1}) for **6** at the BP86-D3(BJ)/TZVP//r^{2}SCAN-3c level of theory.

**EDA-NOCV**	**Dative**<br>**AlI (s) + TPB (s)**	**Electron-sharing**<br>**AlI$^+$ (d) + TPB$^-$ (d)**	**Electron-sharing**<br>**AlI (t) + TPB (t)**
ΔE_{int}	−99.25	−268.95	−208.25
ΔE_{Pauli}	440.21	293.27	418.97
ΔE_{Elstat}	−223.32	−247.98	−261.84
ΔE_{Disp}	−30.58	−30.58	−30.58
ΔE_{Orb}	−285.57	**−283.67**	−334.81
ΔE_{Orb1} σ (B−Al)	−195.64	−120.39	
ΔE_{Orb2} σ (P−Al)	−34.45	−48.78	
ΔE_{Orb3} σ (P−Al)	−33.23	−47.02	
ΔE_{Orb} Rest	−22.25	−67.28	

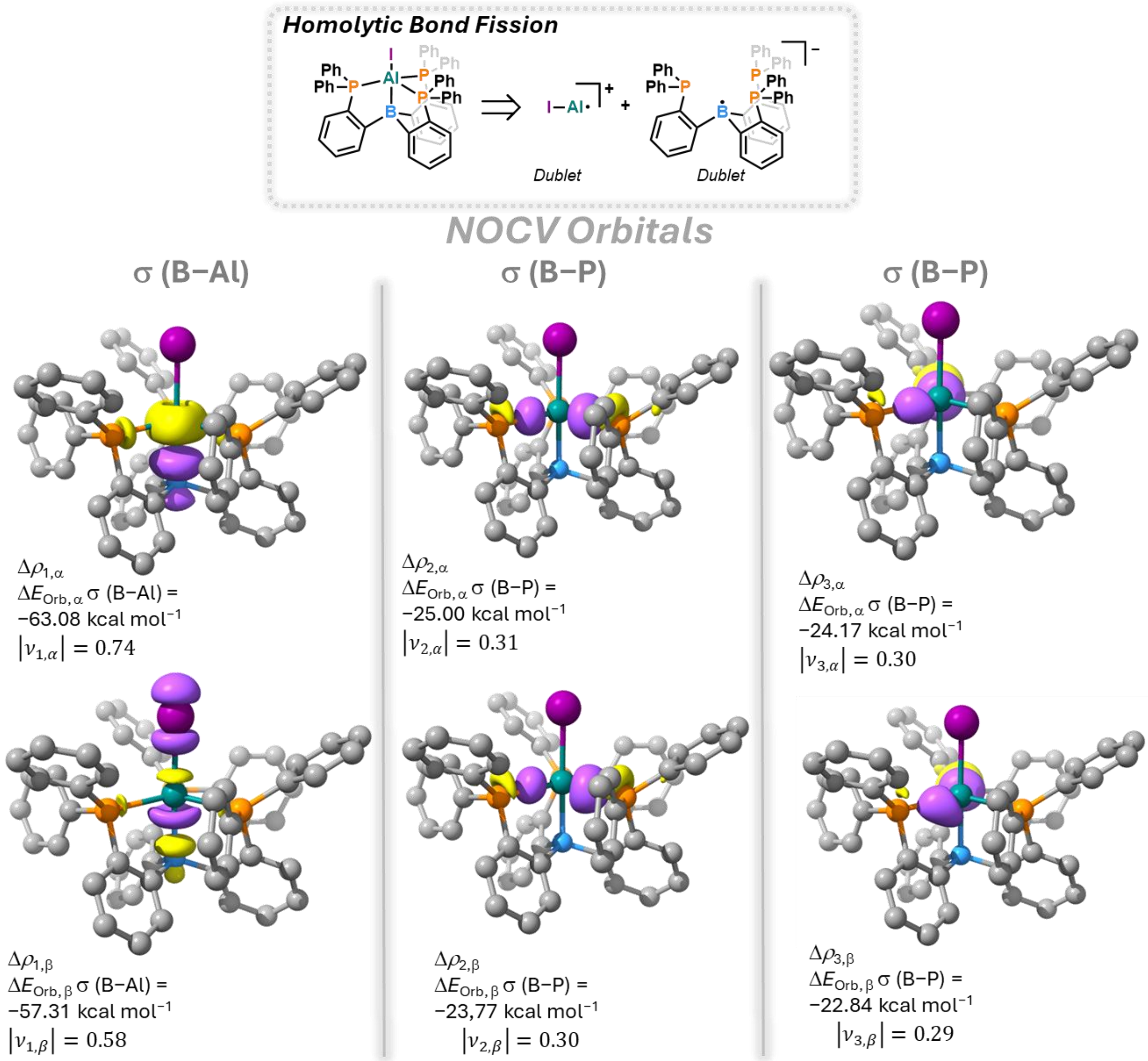

S-Figure 111: Plots of deformation densities $\Delta\rho_{(1)\text{-}(3)}$ of the pairwise orbital interaction for homolytic fragmentation of **6** in AlI^+ and TPB^- (isovalue 0.004 for B−Al bond, 0.002 for Al−P bonds) associated with energy terms $\Delta E_{Orb(1)\text{-}Orb(3)}$ (values in kcal/mol). Charge flows from yellow to purple.

S-2.8 EDA-NOCV analysis of [(TPB)AlH]

S-Table 5: EDA-NOCV results (kcal mol^{-1}) for **7** at the BP86-D3(BJ)/TZVP//r^{2}SCAN-3c level of theory.

**EDA-NOCV**	**Dative**<br>**AlH (s) + TPB (s)**	**Electron-sharing**<br>**AlH^+ (d) + TPB^- (d)**	**Electron-sharing**<br>**AlH (t) + TPB (t)**
ΔE_{int}	−99.83	−256.22	−181.18
ΔE_{Pauli}	399.33	233.91	364.59
ΔE_{Elstat}	−215.67	−228.07	−230.60
ΔE_{Disp}	−18.91	−18.91	−18.91
ΔE_{Orb}	−264.59	**−243.25**	−296.27
ΔE_{Orb1} **σ (B−Al)**	−202.28	–	
ΔE_{Orb2} **σ (P−Al)**	−24.98	–	
ΔE_{Orb3} **σ (P−Al)**	−21.21	–	
ΔE_{Orb} **Rest**	−16.12	–	

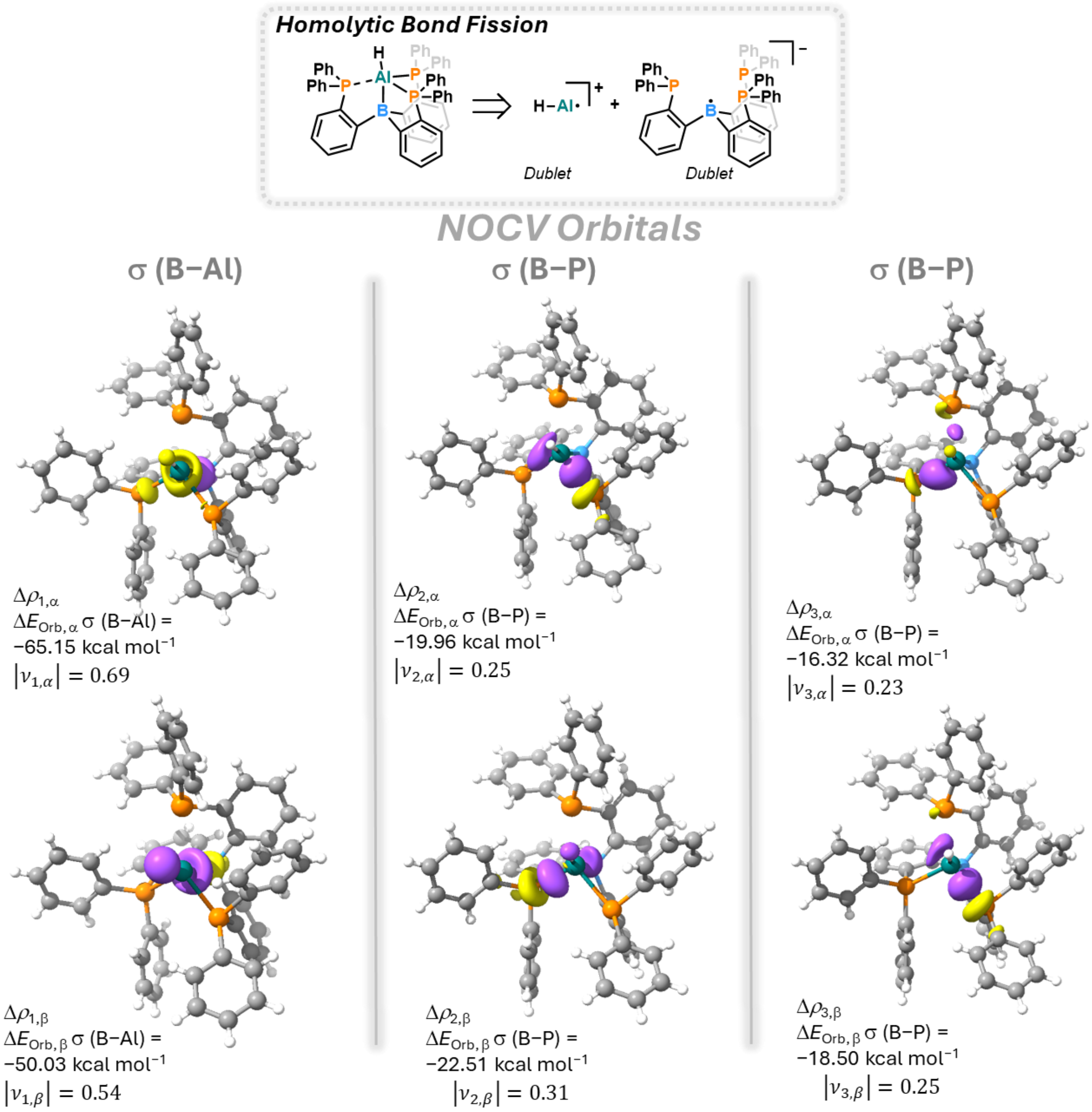

S-Figure 112: Plots of deformation densities $\Delta\rho_{(1)\text{-}(3)}$ of the pairwise orbital interaction for homolytic fragmentation of **7** in AlH^+ and TPB^- (isovalue 0.004 for B−Al bond, 0.002 for Al−P bonds) associated with energy terms $\Delta E_{Orb(1)\text{-}Orb(3)}$ (values in kcal/mol). Charge flows from yellow to purple.

S-2.9 EDA-NOCV analysis for hypothetical Ph_3B-AlBr

The geometry of Ph_3B-AlBr was obtained by restraining the B-Al bond and Br-Al-B bond angle to values computed for [(TPB)AlBr] **5**.

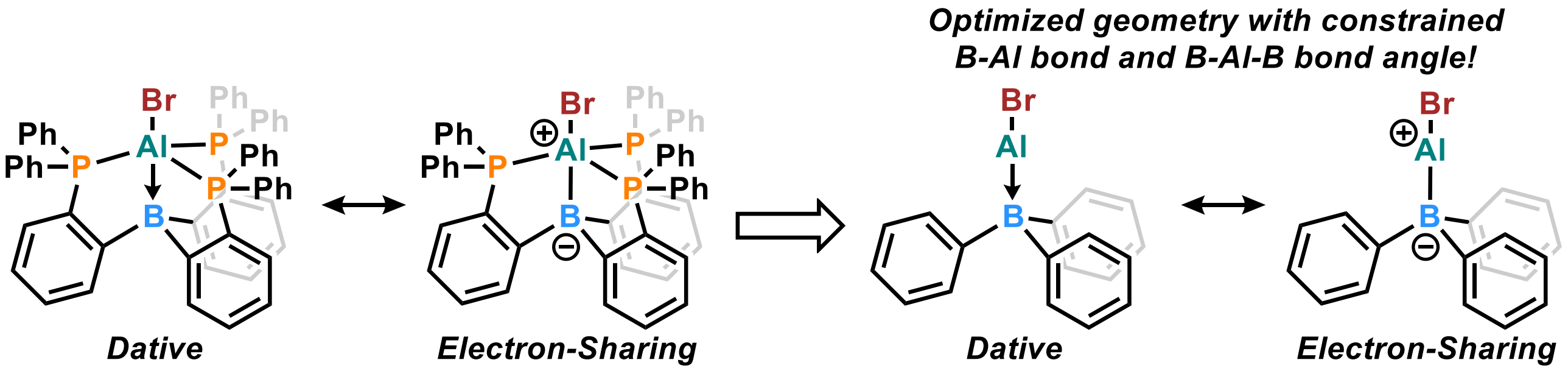

S-Table 6: EDA-NOCV results (kcal mol^{-1}) for Ph_3B-AlBr at the BP86-D3(BJ)/TZVP//r^{2}SCAN-3c level of theory.

EDA-NOCV	Electron-sharing<br>$BrAl^+$ (d) + BPh_3^- (d)	Dative<br>BrAl: (s) + BPh_3 (s)
ΔE_{int}	−15.80	−15.80
ΔE_{Pauli}	74.51	113.90
ΔE_{Elstat}	−137.69	−56.83
ΔE_{Disp}	−9.92	−9.92
ΔE_{Orb}	−126.82	**−62.94**
ΔE_{Orb1} σ (B−Al)	−87.13	−45.70
ΔE_{Orb} Rest	−39.69	−17.24

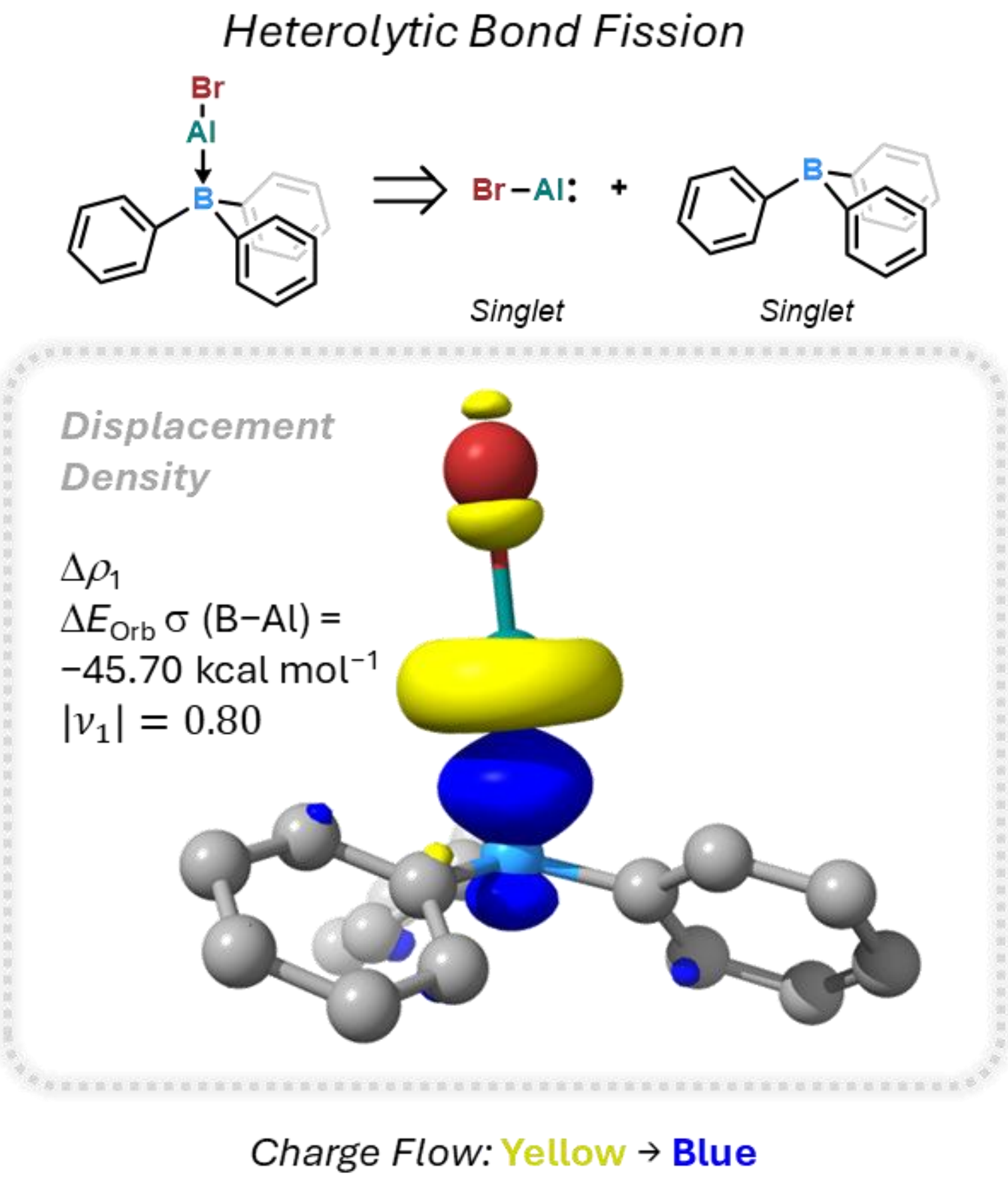

S-Figure 113: Plot of deformation densities $\Delta\rho_{(1)}$ of the pairwise orbital interaction for heterolytic fragmentation of Ph_3B-AlBr in Ph_3B and AlBr (isovalue 0.004 for B−Al bond) associated with energy terms $\Delta E_{Orb(1)}$ (values in kcal/mol). Charge flows from yellow to blue.

S-2.10 QTAIM, NBO and EDA-NOCV analysis for Cp*Al−B(C_6F_5)$_3$ I

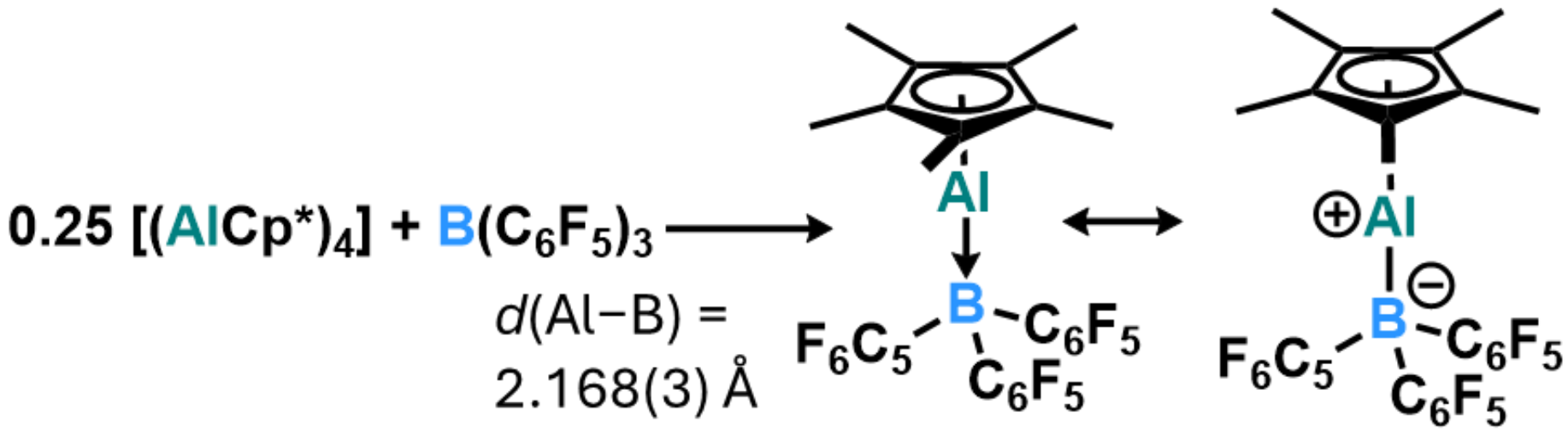

S-Figure 114: Structure of literature known **I**.[37]

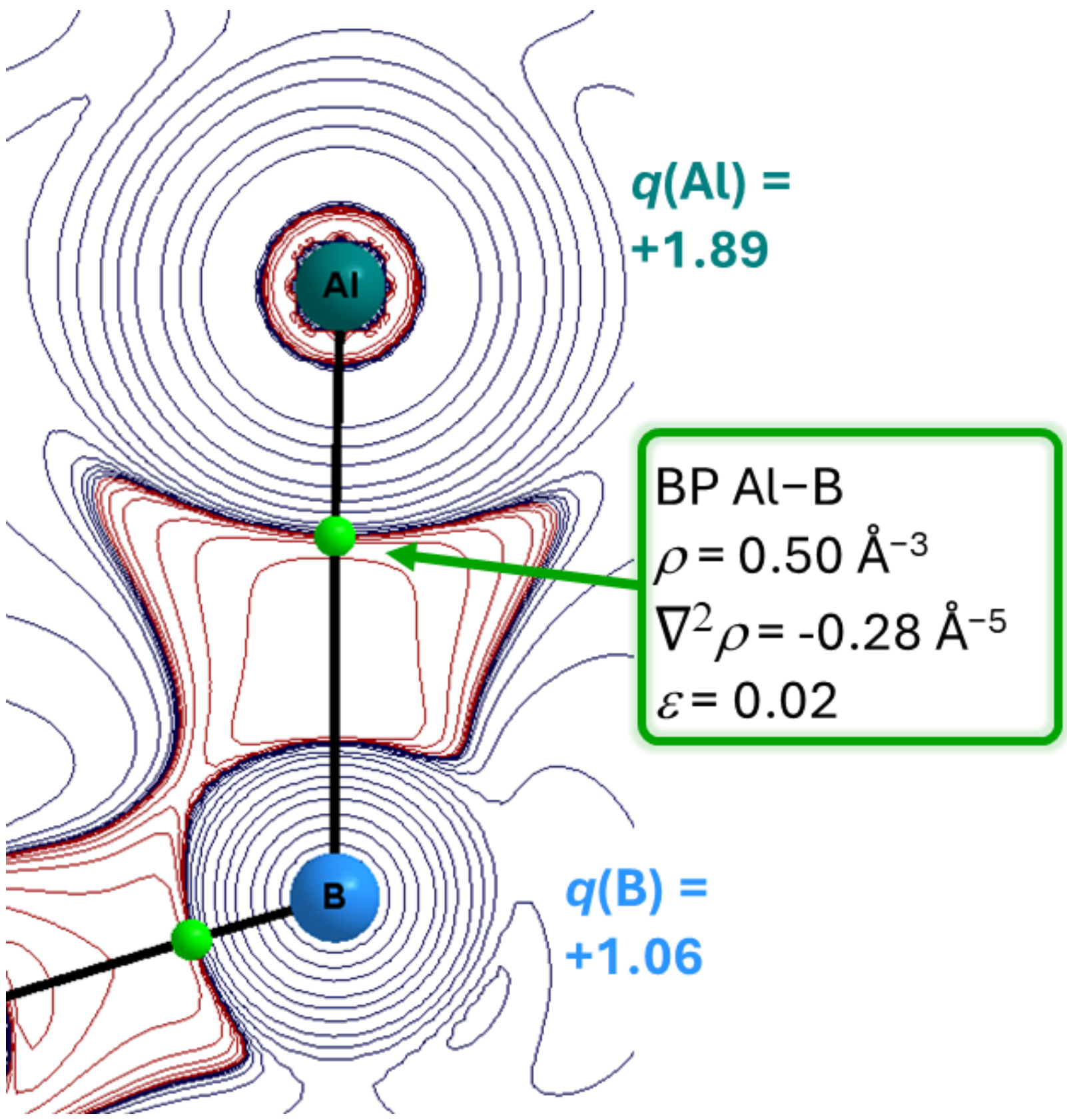

S-Figure 115: Results of QTAIM analysis for **I**. ρ = electron density; $\nabla^2\rho$ = Laplacian of electron density; ε = ellipticity; q = atomic charge. Plane defined by Al and B atoms as well as a carbon atom bonded to boron.

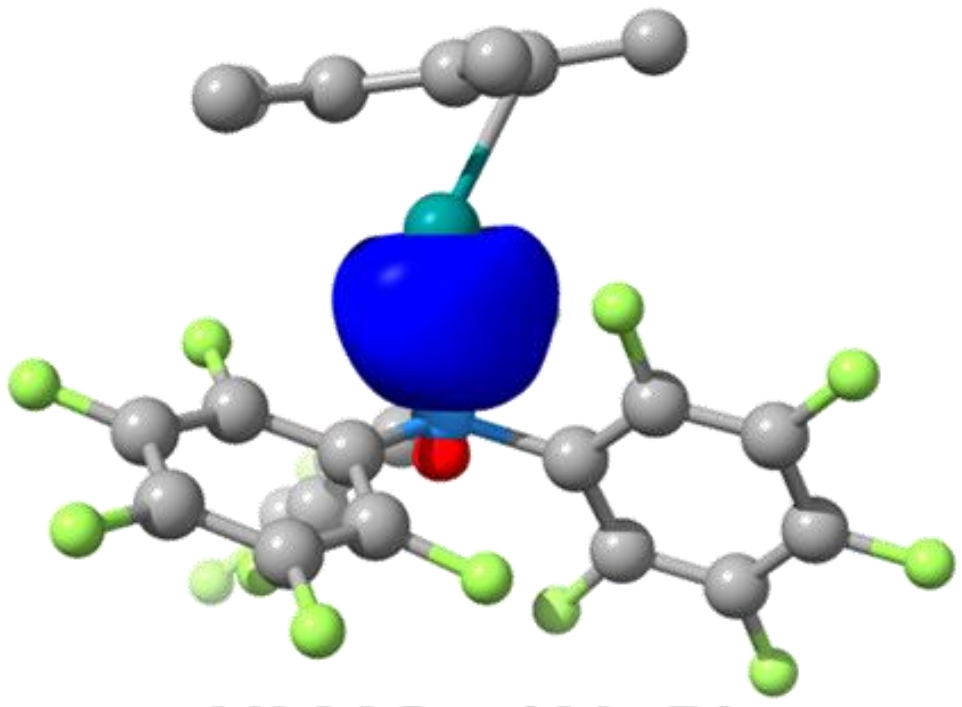

NLMO σ(Al−B)

NBO Analysis

→ *Covalent Al−B Bond:*

Occ. No. = 1.87

38.7 % Al($sp^{0.05}$)

61.3 % B($sp^{4.45}$)

WBI = 0.79

Natural population analysis:

Al +1.74, B −0.28

S-Figure 116: Results of NBO analysis for **I** (isovalue 0.07).

S-Table 7: EDA-NOCV results (kcal mol^{-1}) for **III** at the BP86-D3(BJ)/TZVP//r^2SCAN-3c level of theory.

**EDA-NOCV**	**Dative**<br>**AlCp* (s) + BCF (s)**	**Electron-sharing**<br>**$AlCp*^{+}$ (d) + BCF^{-} (d)**
**ΔE_{int}**	−54.80	−149.25
**ΔE_{Pauli}**	184.82	107.82
**ΔE_{Elstat}**	−104.62	−142.70
**ΔE_{Disp}**	−18.47	−18.47
**ΔE_{Orb}**	−116.53	**−95.91**
**ΔE_{Orb1} σ (B−Al)**	−102.95	−70.10
**ΔE_{Orb} Rest**	−13.58	−25.81

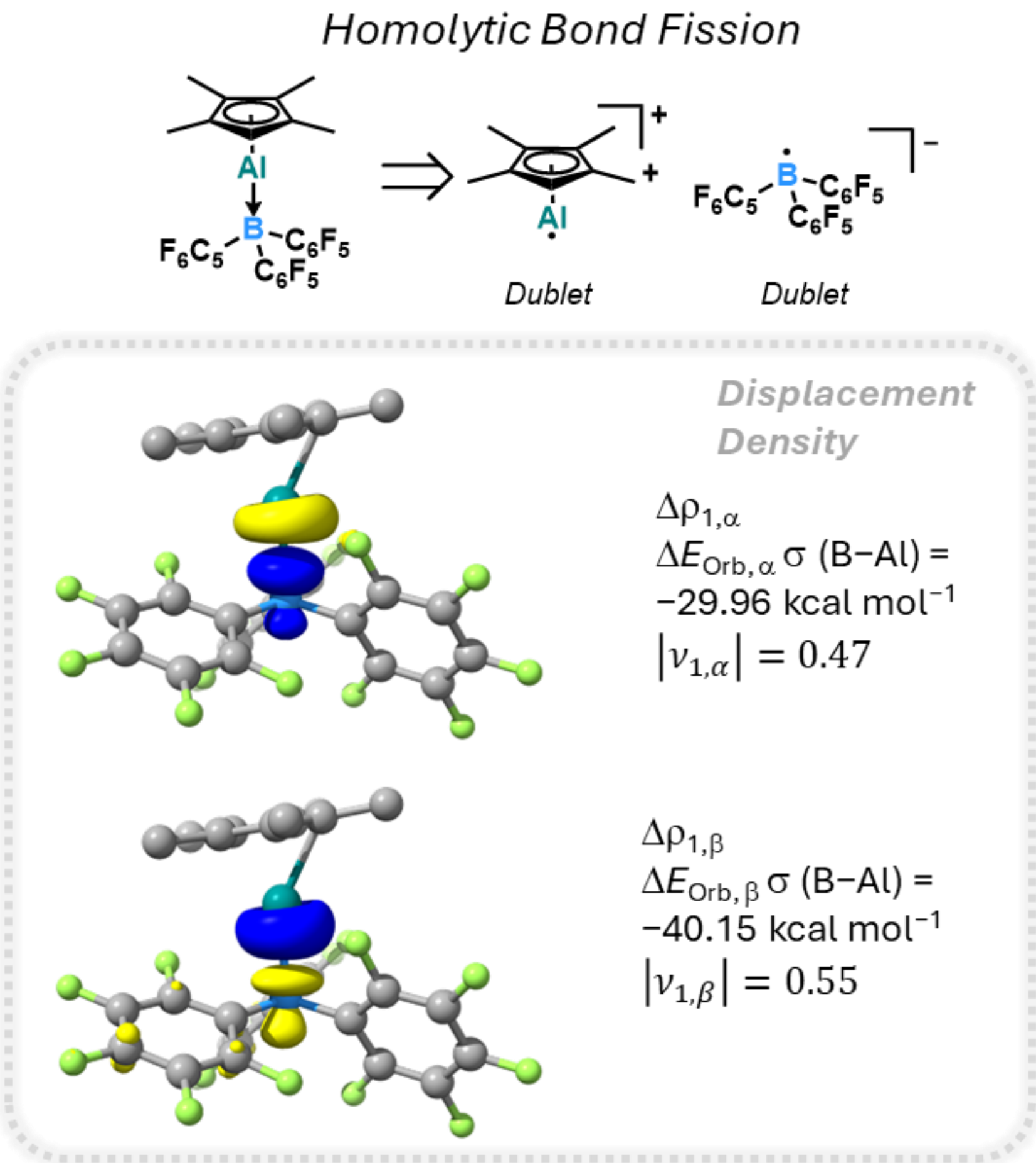

S-Figure 117: Plot of deformation densities $\Delta\rho_{(1)}$ of the pairwise orbital interaction for homolytic fragmentation of **I** in $Cp^{*}Al^{+}$ and BCF^{-} (isovalue 0.004 for B−Al bond) associated with energy terms $\Delta E_{Orb(1)}$ (values in kcal/mol). Charge flows from yellow to blue.

S-2.11 QTAIM, NBO and EDA-NOCV analysis for Cp*Al−BFPh II

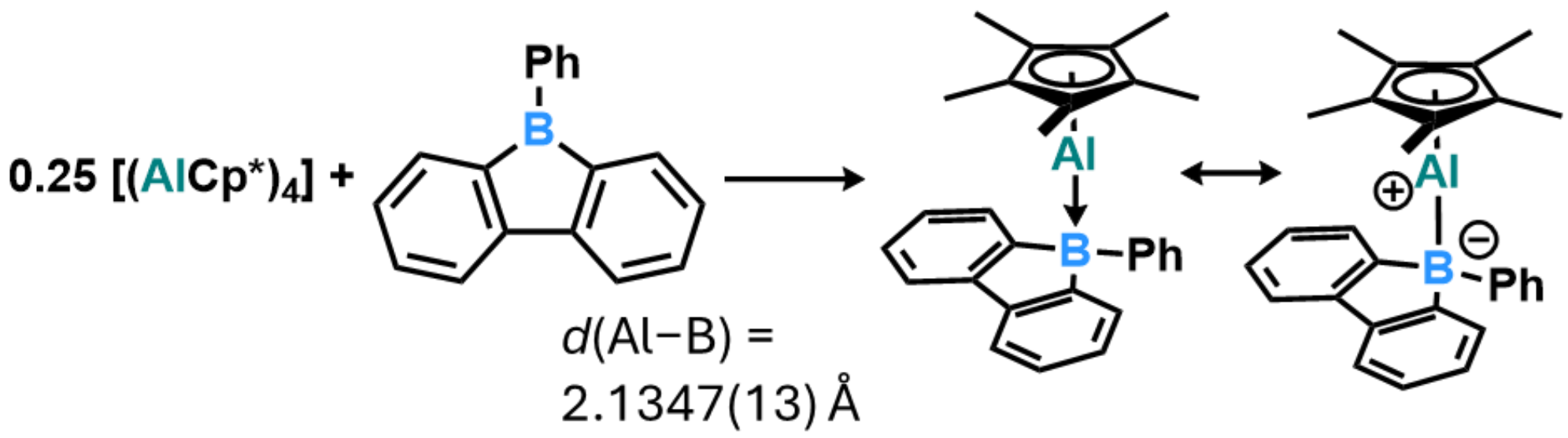

S-Figure 118: Structure of literature known **II**.[38]

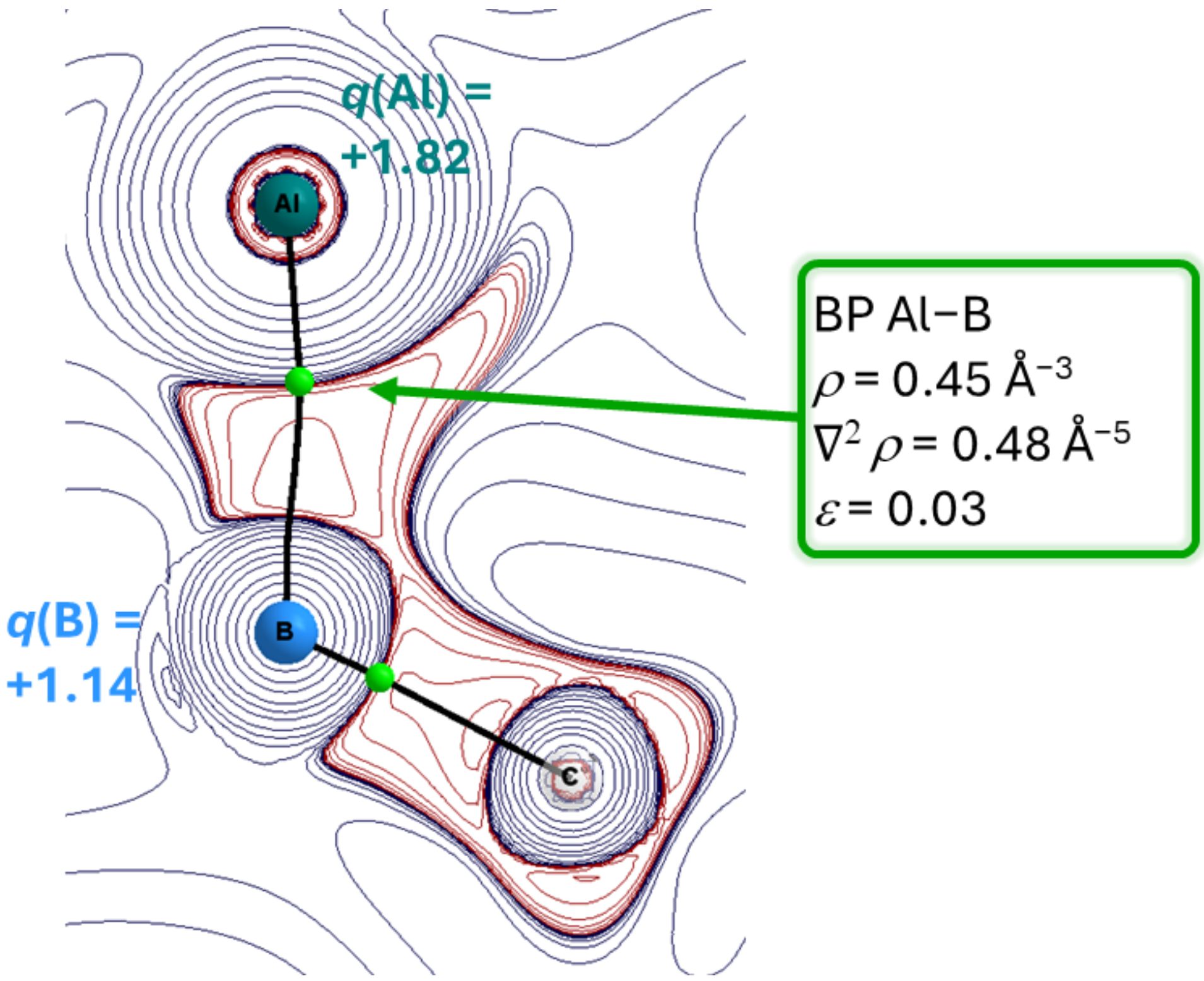

S-Figure 119: Results of QTAIM analysis for **II**. ρ = electron density; $\nabla^2\rho$ = Laplacian of electron density; ε = ellipticity; q = atomic charge. Plane defined by Al and B atoms as well as a carbon atom bonded to boron.

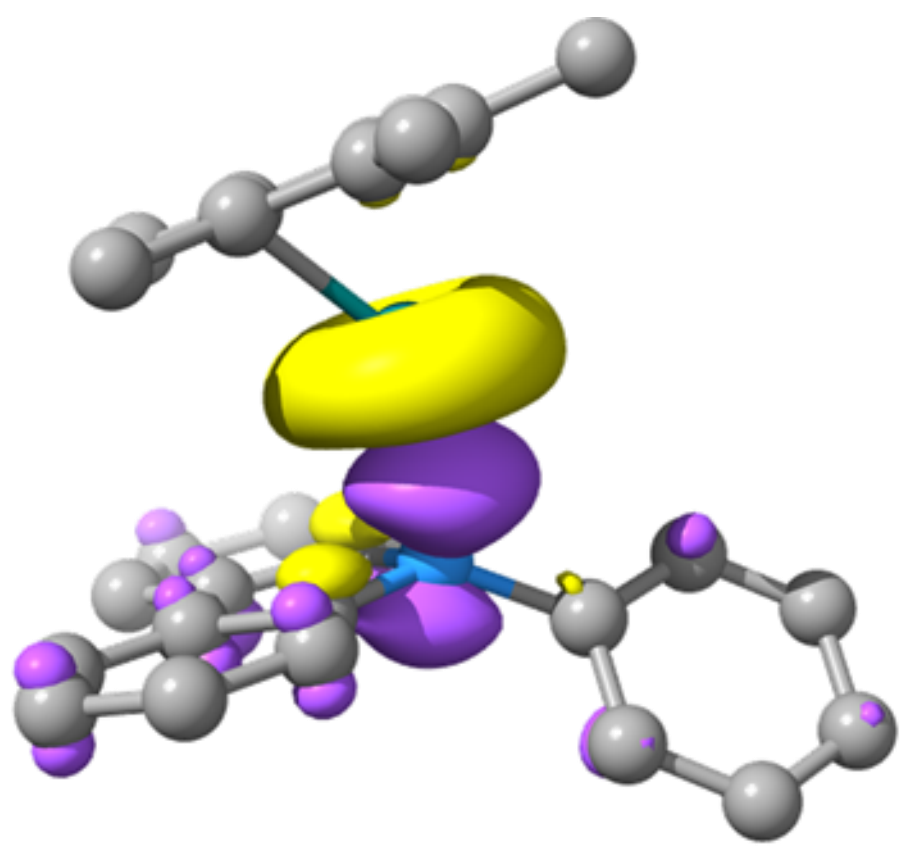

S-Figure 120: Results of NBO analysis for **II** (isovalue 0.07).

S-Table 8: EDA-NOCV results (kcal mol^{-1}) for **II** at the BP86-D3(BJ)/TZVP//r^{2}SCAN-3c level of theory.

**EDA-NOCV**	**Dative**<br>**AlCp* (s) + PhBF (s)**	**Electron-sharing**<br>**AlCp*$^+$ (d) + PhBF$^-$ (d)**
ΔE_{int}	−41.75	−164.56
ΔE_{Pauli}	164.30	104.07
ΔE_{Elstat}	−90.50	−147.09
ΔE_{Disp}	−18.41	−18.41
ΔE_{Orb}	**−97.14**	−103.14
ΔE_{Orb1} σ (B−Al)	−80.40	−70.74
ΔE_{Orb} Rest	−16.74	−32.40

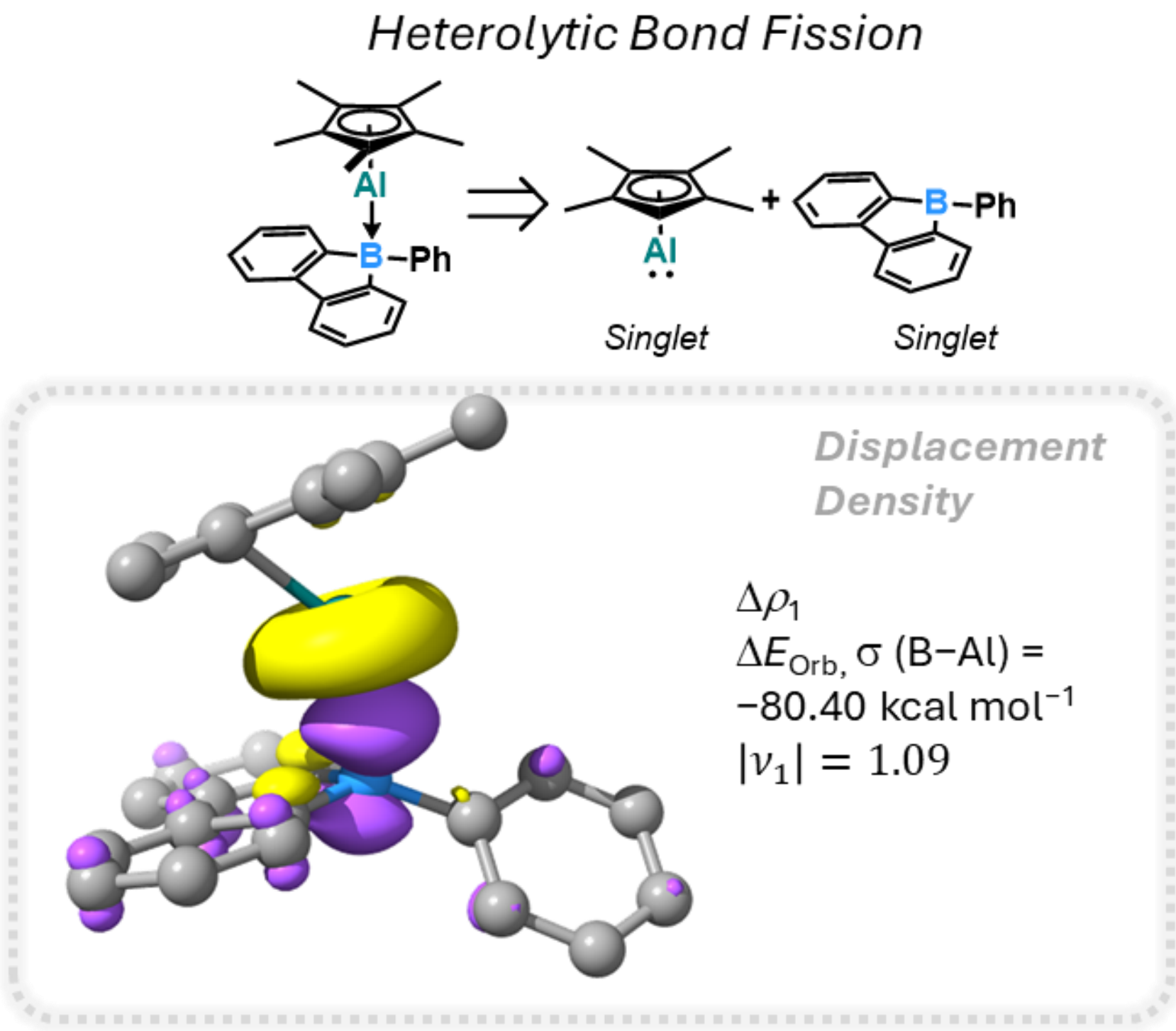

S-Figure 121: Plot of deformation densities $\Delta\rho_{(1)}$ of the pairwise orbital interaction for heterolytic fragmentation of **II** in Cp*Al and BFPh (isovalue 0.004 for B−Al bond) associated with energy terms $\Delta E_{Orb(1)}$ (values in kcal/mol). Charge flows from yellow to blue.

S-2.12 QTAIM, NBO and EDA-NOCV analyses of literature known $R_2B{-}AlR_2$ III

d(Al−B) = 2.191(2) Å

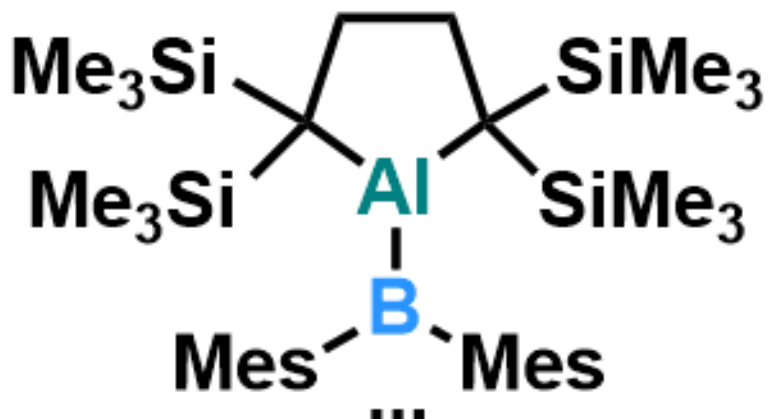

III

S-Figure 122: Structure of literature known aluminaborane **III**.[39]

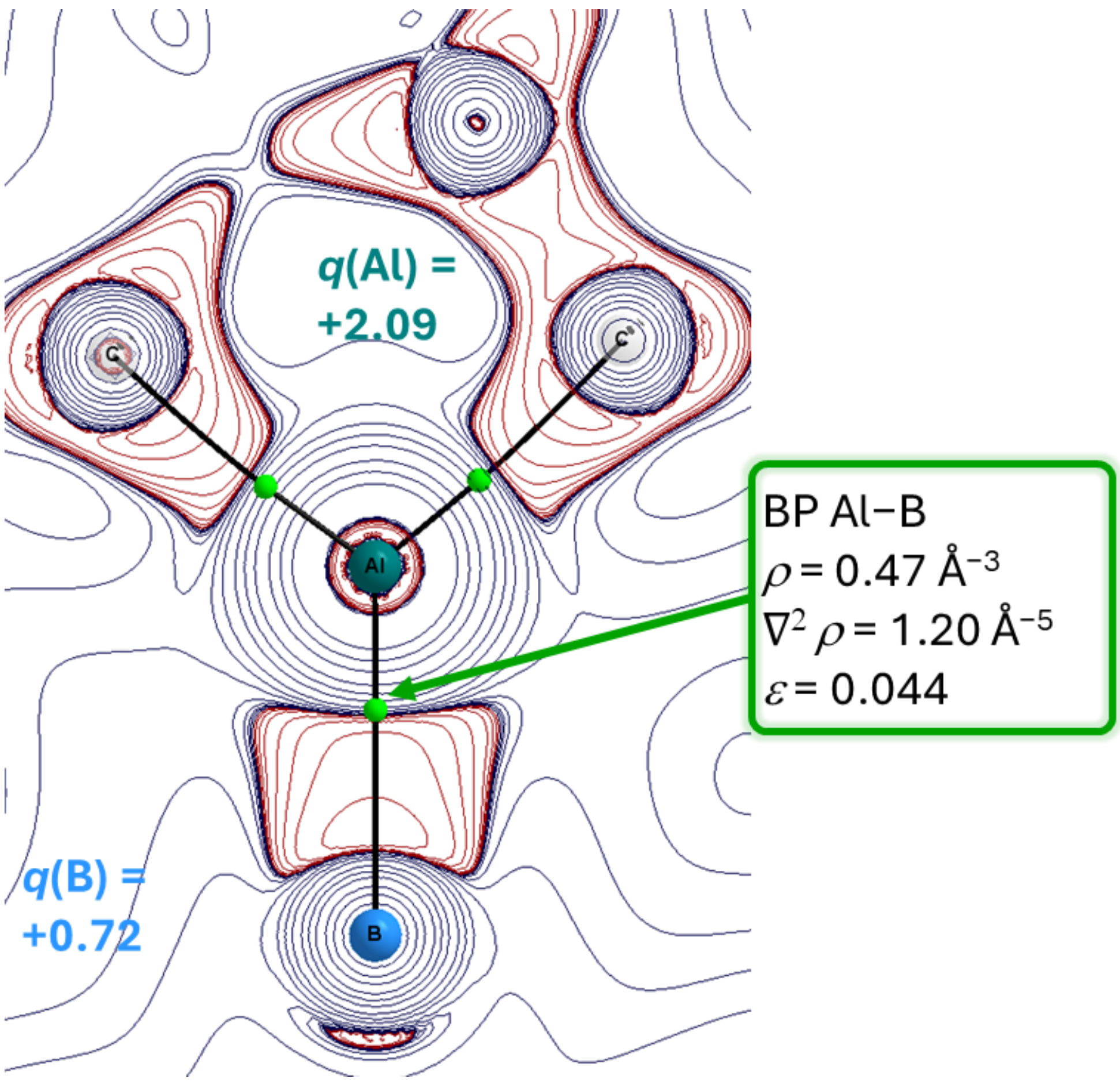

S-Figure 123: Results of QTAIM analysis for **III**. ρ = electron density; $\nabla^2\rho$ = Laplacian of electron density; ε = ellipticity; q = atomic charge. Plane defined by Al and B atoms as well as a carbon atom bonded to aluminum.

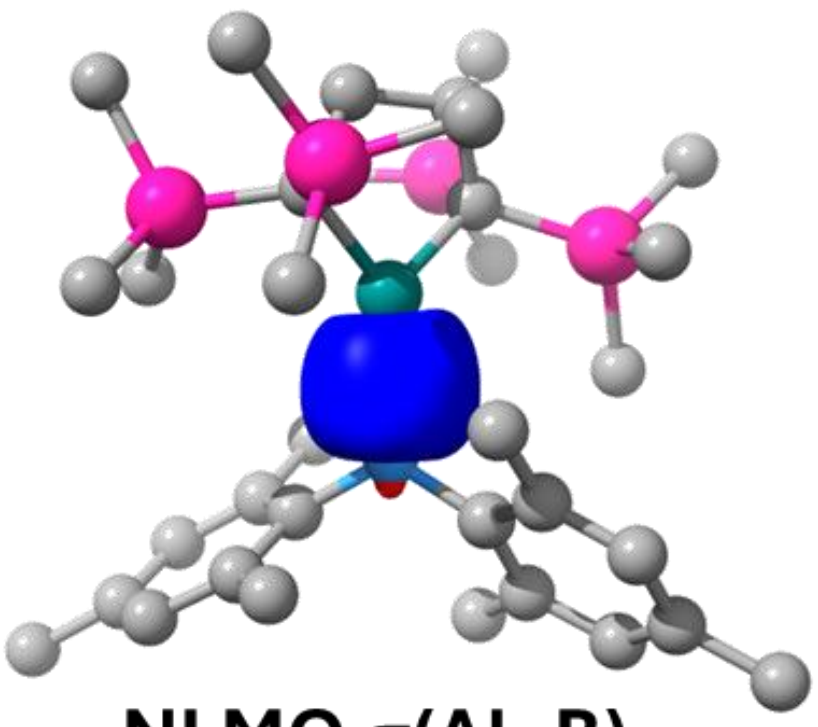

NLMO σ(Al−B)

NBO Analysis
Occ. No. = 1.87
28.0 % Al($sp^{0.25}$)
72.0 % B($sp^{1.64}$)
WBI = 0.62
Natural population analysis:
Al +1.86, B +0.32

S-Figure 124: Results of NBO analysis for **III** (isovalue 0.07).

S-Table 9: EDA-NOCV results (kcal mol^{-1}) for **III** at the BP86-D3(BJ)/TZVP//r^2SCAN-3c level of theory.

**EDA-NOCV**	**Electron-sharing** R_2**Al (d) + BMes**$_2$ **(d)**	**Dative** R_2Al^- **(s) + BMes**$_2^+$ **(s)**	**Dative** R_2Al^+ **(s) + BMes**$_2^-$ **(s)**
ΔE_{int}	−83.96	−200.09	−212.74
ΔE_{Pauli}	131.65	233.75	155.69
ΔE_{Elstat}	−104.12	−204.65	−190.53
ΔE_{Disp}	−29.14	−29.14	−29.14
ΔE_{Orb}	**−82.35**	−200.04	−148.76
ΔE_{Orb1} **σ (B−Al)**	−67.28	−172.88	−109.21
ΔE_{Orb} **Rest**	−25.81	−27.24	−39.55

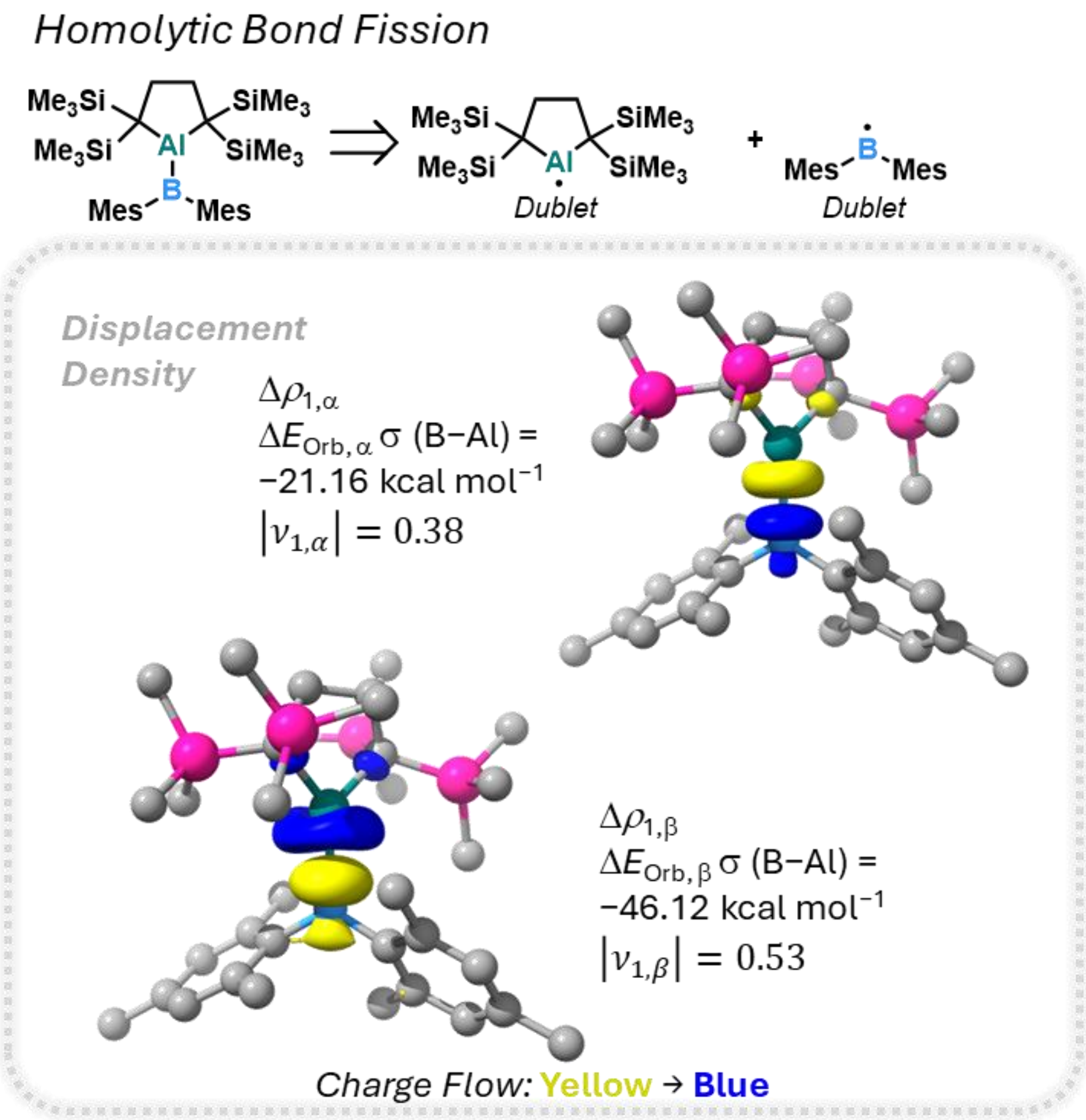

S-Figure 125: Plot of deformation densities $\Delta\rho_{(1)}$ of the pairwise orbital interaction for heteroyltic fragmentation of **III** in Ph_3B and AlBr (isovalue 0.004 for B−Al bond, 0.002 for Al−P bonds) associated with energy terms $\Delta E_{Orb(1)}$ (values in kcal/mol). Charge flows from yellow to blue.

S-3 scXRD Tables

S-Table 10: Summary of crystallographic data for the molecular structures.

	**[(TPB)AlCl] 4**	**[(TPB)AlBr] 5**	**[(TPB)AlI] 6**
CCDC number	2538245	2538245	2538244
Empirical formula	$C_{54}H_{42}AlBClP_3$	$C_{61}H_{50}AlBBrP_3$	$C_{54}H_{42}AlBIP_3$
Formula weight	857.115	993.707	948.47
Temperature [K]	100.00	100.00	100(2)
Crystal system	triclinic	triclinic	monoclinic
Space group (number)	$P\bar{1}$ (2)	$P\bar{1}$ (2)	$P2_1/c$ (14)
a [Å]	12.4831(5)	13.2889(4)	13.3336(4)
b [Å]	13.2327(5)	13.8207(4)	14.5177(5)
c [Å]	14.7608(6)	14.0045(5)	22.4484(7)
α [°]	83.727(1)	76.287(1)	90
β [°]	88.019(1)	85.566(1)	90.655(2)
γ [°]	67.161(1)	88.538(1)	90
Volume [$Å^3$]	2233.57(15)	2491.23(14)	4345.1(2)
Z	2	2	4
ρ_{calc} [gcm^{-3}]	1.274	1.325	1.450
μ [mm^{-1}]	0.250	0.982	7.317
F(000)	893.431	1028.802	1928
Crystal size [mm^3]	0.142×0.156×0.285	0.09×0.133×0.219	0.094×0.133×0.205
Crystal colour	red	red	red
Crystal shape	block	block	block
Radiation	Mo K_α (λ=0.71073 Å)	Mo K_α (λ=0.71073 Å)	CuK_α (λ=1.54178 Å)
2θ range [°]	4.52 to 59.32 (0.72 Å)	4.14 to 66.28 (0.65 Å)	7.25 to 140.33 (0.82 Å)
Index ranges	−17 ≤ h ≤ 17 −18 ≤ k ≤ 18 −20 ≤ l ≤ 20	−20 ≤ h ≤ 21 −21 ≤ k ≤ 21 −20 ≤ l ≤ 23	−16 ≤ h ≤ 16 0 ≤ k ≤ 17 0 ≤ l ≤ 27
Reflections collected	78866	116637	8215
Independent reflections	12138 R_{int} = 0.0610 R_{sigma} = 0.0449	18864 R_{int} = 0.0790 R_{sigma} = 0.0644	8215 R_{int} = 0.1531 R_{sigma} = 0.0761
Completeness to θ = 25.242°	96.1 %	99.2 %	99.5 %
Data / Restraints / Parameters	12138 / 426 / 541	18864 / 1034 / 725	8215 / 762 / 541
Absorption correction T_{min}/T_{max} (method)	0.6915 / 0.7459 (none)	0.6957 / 0.7470 (none)	0.2285 / 0.4314 (multi-scan)
Goodness-of-fit on F^2	1.0201	1.0158	1.028
Final *R* indexes [$I \geq 2\sigma(I)$]	R_1 = 0.0330 wR_2 = 0.0640	R_1 = 0.0383 wR_2 = 0.0699	R_1 = 0.0706 wR_2 = 0.1728
Final *R* indexes [all data]	R_1 = 0.0527 wR_2 = 0.0718	R_1 = 0.0630 wR_2 = 0.0784	R_1 = 0.0916 wR_2 = 0.1860
Largest peak/hole [$eÅ^{-3}$]	0.38/-0.33	1.24/-1.72	3.22/-0.51

S-Table 11: Summary of crystallographic data for the molecular structures.

	**[(TPB)AlH] 7**	**[(TPB)AlBr(dmap)] 8**	**[(TPB)AlBr(IMe$_4$)] 9**
CCDC number	2562648	2562651	2562650
Empirical formula	$C_{60}H_{49}AlBP_3$	$C_{65}H_{60}AlBBrN_2OP_3$	$C_{70.83}H_{67.34}AlBBrN_2P_3$
Formula weight	900.69	1095.76	1157.15
Temperature [K]	100(2)	100(2)	100(2)
Crystal system	monoclinic	triclinic	triclinic
Space group (number)	$P2_1/c$ (14)	$P\bar{1}$ (2)	$P\bar{1}$ (2)
a [Å]	13.5594(11)	13.2518(7)	13.5546(11)
b [Å]	31.751(2)	13.7718(7)	14.1089(9)
c [Å]	12.3508(11)	15.8022(8)	16.7942(14)
α [°]	90	106.216(2)	73.481(3)
β [°]	114.883(3)	97.361(2)	73.713(3)
γ [°]	90	92.284(2)	76.667(3)
Volume [Å^3]	4823.7(7)	2737.7(2)	2915.6(4)
Z	4	2	2
ρ_{calc} [gcm^{-3}]	1.240	1.329	1.318
μ [mm^{-1}]	0.181	0.900	0.848
F(000)	1888	1140	1209
Crystal size [mm^3]	0.066×0.131×0.297	0.126×0.135×0.227	0.110×0.166×0.357
Crystal colour	yellow	yellow	yellow
Crystal shape	plate	block	block
Radiation	MoK_α (λ=0.71073 Å)	MoK_α (λ=0.71073 Å)	MoK_α (λ=0.71073 Å)
2θ range [°]	3.85 to 54.39 (0.78 Å)	4.21 to 63.22 (0.68 Å)	3.93 to 61.23 (0.70 Å)
Index ranges	−17 ≤ h ≤ 17 −38 ≤ k ≤ 40 −15 ≤ l ≤ 15	−19 ≤ h ≤ 19 −20 ≤ k ≤ 20 −23 ≤ l ≤ 23	−19 ≤ h ≤ 19 −20 ≤ k ≤ 19 −24 ≤ l ≤ 24
Reflections collected	71994	157938	115314
Independent reflections	10697 R_{int} = 0.1041 R_{sigma} = 0.0695	18360 R_{int} = 0.0694 R_{sigma} = 0.0372	17932 R_{int} = 0.1060 R_{sigma} = 0.0684
Completeness to θ = 25.242°	99.9 %	99.7 %	99.7 %
Data / Restraints / Parameters	10697 / 4290 / 590	18360 / 66 / 669	17932 / 4008 / 802
Absorption correction T_{min}/T_{max} (method)	0.5894 / 0.7455 (multi-scan)	0.6309 / 0.7463 (multi-scan)	0.6108 / 0.7461 (multi-scan)
Goodness-of-fit on F^2	1.031	1.041	1.041
Final *R* indexes [I≥2σ(I)]	R_1 = 0.0594 wR_2 = 0.1380	R_1 = 0.0364 wR_2 = 0.0888	R_1 = 0.0477 wR_2 = 0.1031
Final *R* indexes [all data]	R_1 = 0.0841 wR_2 = 0.1520	R_1 = 0.0467 wR_2 = 0.0945	R_1 = 0.0641 wR_2 = 0.1128
Largest peak/hole [eÅ^{-3}]	0.55/−0.42	0.59/-0.52	0.66/-0.57

S-Table 12: Summary of crystallographic data for the molecular structures.

	**[(TPB)Al(IMe$_4$)]$^+$[B(C$_6$F$_5$)$_4$]$^-$ 11**	**[Al(bipy)$_3$]$^+$Br$^-$ 12**	**[(TPB)Al(dmap)]$^+$[B(C$_6$F$_5$)$_4$]$^-$ 10**
CCDC number	2562652	2562647	2564596
Empirical formula	$C_{44.50}H_{32}Al_{0.50}BF_{10}NO_{0.50}P_{1.50}$	$C_{36}H_{28}AlBrF_2N_6$	$C_{88}H_{55}AlB_2F_{20}N_2P_3$
Formula weight	849.46	689.546	1661.85
Temperature [K]	100(2)	100.00	100(2)
Crystal system	monoclinic	monoclinic	triclinic
Space group (number)	$P2_1/c$ (14)	$P2_1/c$ (14)	$P\bar{1}$ (2)
a [Å]	14.3036(7)	9.0658(5)	18.9722(11)
b [Å]	14.1079(7)	16.6194(9)	20.0492(12)
c [Å]	38.9711(18)	20.7084(11)	20.2900(11)
α [°]	90	90	79.882(2)
β [°]	93.731(2)	90.608(2)	80.745(2)
γ [°]	90	90	86.635(2)
Volume [Å^3]	7847.5(7)	3119.9(3)	7495.2(7)
Z	8	4	4
ρ_{calc} [gcm^{-3}]	1.438	1.468	1.473
μ [mm^{-1}]	0.185	1.399	0.191
F(000)	3472	1408.298	3380
Crystal size [mm^3]	0.088×0.117×0.830	0.047×0.154×0.278	0.059×0.100×0.368
Crystal colour	yellow	black	red
Crystal shape	plate	needle	plate
Radiation	MoK_α (λ=0.71073 Å)	Mo K_α (λ=0.71073 Å)	MoK_α (λ=0.71073 Å)
2θ range [°]	4.06 to 54.31 (0.78 Å)	3.94 to 59.28 (0.72 Å)	3.69 to 54.40 (0.78 Å)
Index ranges	−18 ≤ h ≤ 18 −18 ≤ k ≤ 18 −50 ≤ l ≤ 42	−12 ≤ h ≤ 12 −23 ≤ k ≤ 23 −28 ≤ l ≤ 27	−24 ≤ h ≤ 24 −25 ≤ k ≤ 25 −26 ≤ l ≤ 26
Reflections collected	86597	59979	423714
Independent reflections	17270 R_{int} = 0.0587 R_{sigma} = 0.0492	8782 R_{int} = 0.0642 R_{sigma} = 0.0406	33303 R_{int} = 0.1179 R_{sigma} = 0.0509
Completeness to θ = 25.242°	99.2 %	99.8 %	99.7 %
Data / Restraints / Parameters	17270 / 2448 / 1124	8782 / 395 / 415	33303 / 14912 / 2150
Absorption correction T_{min}/T_{max} (method)	0.5799 / 0.7455 (multi-scan)	0.6075 / 0.7459 (multi-scan)	0.6639 / 0.7455 (multi-scan)
Goodness-of-fit on F^2	1.060	1.0143	1.018
Final *R* indexes [$I \geq 2\sigma(I)$]	R_1 = 0.0790 wR_2 = 0.1835	R_1 = 0.0358 wR_2 = 0.0672	R_1 = 0.0483 wR_2 = 0.1131
Final *R* indexes [all data]	R_1 = 0.1033 wR_2 = 0.1963	R_1 = 0.0538 wR_2 = 0.0733	R_1 = 0.0790 wR_2 = 0.1326
Largest peak/hole [eÅ^{-3}]	1.15/-0.70	0.60/-0.46	0.36/-0.36

S-Table 13: Summary of crystallographic data for the molecular structures.

	**TPB V**
CCDC number	2562649
Empirical formula	$C_{36}H_{28}AlBrF_2N_6$
Formula weight	689.546
Temperature [K]	100.00
Crystal system	monoclinic
Space group (number)	$P2_1/c$ (14)
a [Å]	9.0658(5)
b [Å]	16.6194(9)
c [Å]	20.7084(11)
α [°]	90
β [°]	90.608(2)
γ [°]	90
Volume [$Å^3$]	3119.9(3)
Z	4
ρ_{calc} [gcm^{-3}]	1.468
μ [mm^{-1}]	1.399
F(000)	1408.298
Crystal size [mm^3]	0.047×0.154×0.278
Crystal colour	black
Crystal shape	needle
Radiation	Mo K_α (λ=0.71073 Å)
2θ range [°]	3.94 to 59.28 (0.72 Å)
Index ranges	$-12 \le h \le 12$ $-23 \le k \le 23$ $-28 \le l \le 27$
Reflections collected	59979
Independent reflections	8782 R_{int} = 0.0642 R_{sigma} = 0.0406
Completeness to θ = 25.242°	99.8 %
Data / Restraints / Parameters	8782 / 395 / 415
Absorption correction T_{min}/T_{max} (method)	0.6075 / 0.7459 (multi-scan)
Goodness-of-fit on F^2	1.0143
Final *R* indexes [$I \geq 2\sigma(I)$]	R_1 = 0.0358 wR_2 = 0.0672
Final *R* indexes [all data]	R_1 = 0.0538 wR_2 = 0.0733
Largest peak/hole [$eÅ^{-3}$]	0.60/-0.46

S-4 Literature

[1] T. M. Maier, P. Coburger, N. P. van Leest, E. Hey-Hawkins and R. Wolf, "Direct synthesis of an anionic 13-vertex closo-cobaltacarborane cluster," *Dalton Transactions* 48, no. 42 (2019): 15772. https://doi.org/10.1039/c9dt03111a.

[2] L. Omann and M. Oestreich, "Catalytic Access to Indole-Fused Benzosiloles by 2-Fold Electrophilic C–H Silylation with Dihydrosilanes," *Organometallics* 36, no. 4 (2017): 767. https://doi.org/10.1021/acs.organomet.6b00801.

[3] A. S. Chang, K. E. Kawamura and H. S. Henness et al., "(NHC)Ni(0)-Catalyzed Branched-Selective Alkene Hydrosilylation with Secondary and Tertiary Silanes," *ACS Catalysis* 12, no. 18 (2022): 11002. https://doi.org/10.1021/acscatal.2c03580.

[4] J. Cosier and A. M. Glazer, "A nitrogen-gas-stream cryostat for general X-ray diffraction studies," *Journal of applied crystallography* 19, no. 2 (1986): 105. https://doi.org/10.1107/S0021889886089835.

[5] L. Krause, R. Herbst-Irmer, G. M. Sheldrick and D. Stalke, "Comparison of silver and molybdenum microfocus X-ray sources for single-crystal structure determination," *Journal of applied crystallography* 48, no. Pt 1 (2015): 3. https://doi.org/10.1107/S1600576714022985.

[6] G. M. Sheldrick, "SHELXT - integrated space-group and crystal-structure determination," *Acta crystallographica. Section A, Foundations and advances* 71, no. Pt 1 (2015): 3. https://doi.org/10.1107/S2053273314026370.

[7] G. M. Sheldrick, "Crystal structure refinement with SHELXL," *Acta crystallographica. Section C, Structural chemistry* 71, no. Pt 1 (2015): 3. https://doi.org/10.1107/S2053229614024218.

[8] C. B. Hübschle, G. M. Sheldrick and B. Dittrich, "ShelXle: a Qt graphical user interface for SHELXL," *Journal of applied crystallography* 44, no. Pt 6 (2011): 1281. https://doi.org/10.1107/S0021889811043202.

[9] G. M. Sheldrick, "A short history of SHELX," *Acta Crystallogr. A* 64, no. Pt 1 (2008): 112. https://doi.org/10.1107/S0108767307043930.

[10] D. Kratzert, J. J. Holstein and I. Krossing, "DSR: enhanced modelling and refinement of disordered structures with SHELXL," *Journal of applied crystallography* 48, no. Pt 3 (2015): 933. https://doi.org/10.1107/S1600576715005580.

[11] Daniel Kratzert, can be found under https://www.xs3.uni-freiburg.de/research/finalcif.

[12] F. Kleemiss, O. V. Dolomanov and M. Bodensteiner et al., "Accurate crystal structures and chemical properties from NoSpherA2," *Chemical Science* 12, no. 5 (2020): 1675. https://doi.org/10.1039/d0sc05526c.

[13] L. Midgley, L. J. Bourhis and O. V. Dolomanov et al., "Vanishing of the atomic form factor derivatives in non-spherical structural refinement - a key approximation scrutinized in the case of Hirshfeld atom refinement," *Acta crystallographica. Section A, Foundations and advances* 77, no. Pt 6 (2021): 519. https://doi.org/10.1107/S2053273321009086.

[14] H. Kameo, Y. Hashimoto and H. Nakazawa, "Synthesis of Iridaboratranes Bearing Phosphine-Tethered Borane: Reversible CO/PR 3 (R = Me, OMe, OEt) Substitution Reactions Induced by a σ-Electron-Acceptor Borane Ligand," *Organometallics* 31, no. 11 (2012): 4251. https://doi.org/10.1021/om300216g.

[15] F. Neese, "Software update: The ORCA program system—Version 5.0," *WIREs Computational Molecular Science* 12, no. 5 (2022). https://doi.org/10.1002/wcms.1606.

[16] F. Neese, F. Wennmohs, U. Becker and C. Riplinger, "The ORCA quantum chemistry program package," *J. Chem. Phys.* 152, no. 22 (2020): 224108. https://doi.org/10.1063/5.0004608.

[17] F. Neese, "The ORCA program system," *WIREs Computational Molecular Science* 2, no. 1 (2012): 73. https://doi.org/10.1002/wcms.81.

[18] S. Grimme, A. Hansen, S. Ehlert and J.-M. Mewes, "r2SCAN-3c: A "Swiss army knife" composite electronic-structure method," *J. Chem. Phys.* 154, no. 6 (2021): 64103. https://doi.org/10.1063/5.0040021.

[19] E. Caldeweyher, C. Bannwarth and S. Grimme, "Extension of the D3 dispersion coefficient model," *J. Chem. Phys.* 147, no. 3 (2017): 34112. https://doi.org/10.1063/1.4993215.

[20] E. Caldeweyher, S. Ehlert and A. Hansen et al., "A generally applicable atomic-charge dependent London dispersion correction," *J. Chem. Phys.* 150, no. 15 (2019): 154122. https://doi.org/10.1063/1.5090222.

[21] H. Kruse and S. Grimme, "A geometrical correction for the inter- and intra-molecular basis set superposition error in Hartree-Fock and density functional

theory calculations for large systems," *J. Chem. Phys.* 136, no. 15 (2012): 154101. https://doi.org/10.1063/1.3700154.

[22] A. D. Becke, "Density-functional thermochemistry. III. The role of exact exchange," *J. Chem. Phys.* 98, no. 7 (1993): 5648. https://doi.org/10.1063/1.464913.

[23] Lee, Yang and Parr, "Development of the Colle-Salvetti correlation-energy formula into a functional of the electron density," *Phys. Rev. B Condens. Matter* 37, no. 2 (1988): 785. https://doi.org/10.1103/physrevb.37.785.

[24] S. H. Vosko, L. Wilk and M. Nusair, "Accurate spin-dependent electron liquid correlation energies for local spin density calculations: a critical analysis," *Canadian Journal of Physics* 58, no. 8 (1980): 1200. https://doi.org/10.1139/p80-159.

[25] F. Weigend and R. Ahlrichs, "Balanced basis sets of split valence, triple zeta valence and quadruple zeta valence quality for H to Rn: Design and assessment of accuracy," *Physical chemistry chemical physics : PCCP* 7, no. 18 (2005): 3297. https://doi.org/10.1039/B508541A.

[26] F. Weigend, "Accurate Coulomb-fitting basis sets for H to Rn," *Physical chemistry chemical physics : PCCP* 8, no. 9 (2006): 1057. https://doi.org/10.1039/B515623H.

[27] S. Kossmann and F. Neese, "Efficient Structure Optimization with Second-Order Many-Body Perturbation Theory: The RIJCOSX-MP2 Method," *Journal of chemical theory and computation* 6, no. 8 (2010): 2325. https://doi.org/10.1021/ct100199k.

[28] V. Barone and M. Cossi, "Quantum Calculation of Molecular Energies and Energy Gradients in Solution by a Conductor Solvent Model," *The journal of physical chemistry. A* 102, no. 11 (1998): 1995. https://doi.org/10.1021/jp9716997.

[29] T. Lu and F. Chen, "Multiwfn: a multifunctional wavefunction analyzer," *Journal of Computational Chemistry* 33, no. 5 (2012): 580. https://doi.org/10.1002/jcc.22885.

[30] T. Yanai, D. P. Tew and N. C. Handy, "A new hybrid exchange–correlation functional using the Coulomb-attenuating method (CAM-B3LYP)," 393, no. 1-3 (2004): 51. https://doi.org/10.1016/j.cplett.2004.06.011.

[31] M. P. Mitoraj, A. Michalak and T. Ziegler, "A Combined Charge and Energy Decomposition Scheme for Bond Analysis," *Journal of chemical theory and computation* 5, no. 4 (2009): 962. https://doi.org/10.1021/ct800503d.

[32] G. te Velde, F. M. Bickelhaupt and E. J. Baerends et al., "Chemistry with ADF," *Journal of Computational Chemistry* 22, no. 9 (2001): 931. https://doi.org/10.1002/jcc.1056.

[33] Perdew, "Density-functional approximation for the correlation energy of the inhomogeneous electron gas," *Phys. Rev. B Condens. Matter* 33, no. 12 (1986): 8822. https://doi.org/10.1103/PhysRevB.33.8822.

[34] J. P. Perdew, "Erratum: Density-functional approximation for the correlation energy of the inhomogeneous electron gas," *Phys. Rev. B Condens. Matter* 34, no. 10 (1986): 7406. https://doi.org/10.1103/PhysRevB.34.7406.

[35] S. Grimme, J. Antony, S. Ehrlich and H. Krieg, "A consistent and accurate ab initio parametrization of density functional dispersion correction (DFT-D) for the 94 elements H-Pu," *J. Chem. Phys.* 132, no. 15 (2010): 154104. https://doi.org/10.1063/1.3382344.

[36] S. Grimme, S. Ehrlich and L. Goerigk, "Effect of the damping function in dispersion corrected density functional theory," *Journal of Computational Chemistry* 32, no. 7 (2011): 1456. https://doi.org/10.1002/jcc.21759.

[37] J. D. Gorden, A. Voigt, C. L. B. Macdonald, J. S. Silverman and A. H. Cowley, "A Lewis Acid Adduct of an Alanediyl: An Aluminum(I)−Boron Donor−Acceptor Bond," *Journal of the American Chemical Society* 122, no. 5 (2000): 950. https://doi.org/10.1021/ja993537p.

[38] P. E. Romero, W. E. Piers, S. A. Decker, D. Chau, T. K. Woo and M. Parvez, "η 1 versus η 5 Bonding Modes in Cp*Al(I) Adducts of 9-Borafluorenes," *Organometallics* 22, no. 6 (2003): 1266. https://doi.org/10.1021/om0209935.

[39] S. Kurumada and M. Yamashita, "A Tetraorganyl-Alumaborane with An Al-B σ-Bond and Two Adjacent Lewis-Acidic Centers," *Journal of the American Chemical Society* 144, no. 10 (2022): 4327. https://doi.org/10.1021/jacs.2c01580.
